# Global, regional, and national causes of under-5 mortality in 2000–15: an updated systematic analysis with implications for the Sustainable Development Goals

**DOI:** 10.1016/S0140-6736(16)31593-8

**Published:** 2016-12-17

**Authors:** Li Liu, Shefali Oza, Dan Hogan, Yue Chu, Jamie Perin, Jun Zhu, Joy E Lawn, Simon Cousens, Colin Mathers, Robert E Black

**Affiliations:** a Department of Population Family and Reproductive Health, Johns Hopkins Bloomberg School of Public Health, Baltimore, MD, USA; b The Institute for International Programs, Department of International Health, Johns Hopkins Bloomberg School of Public Health, Baltimore, MD, USA; c London School of Hygiene and Tropical Medicine, London, UK; d Department of Health Statistics and Informatics, World Health Organization, Geneva, Switzerland; e National Office of Maternal and Child Health Surveillance of China, Department of Pediatrics, West China Second University Hospital, Sichuan University, Chengdu, China; f Key Laboratory of Birth Defects and Related Diseases of Women and Children (Sichuan University), Ministry of Education, Chengdu, Sichuan, China

## Abstract

**Background:**

Despite remarkable progress in the improvement of child survival between 1990 and 2015, the Millennium Development Goal (MDG) 4 target of a two-thirds reduction of under-5 mortality rate (U5MR) was not achieved globally. In this paper, we updated our annual estimates of child mortality by cause to 2000–15 to reflect on progress toward the MDG 4 and consider implications for the Sustainable Development Goals (SDG) target for child survival.

**Methods:**

We increased the estimation input data for causes of deaths by 43% among neonates and 23% among 1–59-month-olds, respectively. We used adequate vital registration (VR) data where available, and modelled cause-specific mortality fractions applying multinomial logistic regressions using adequate VR for low U5MR countries and verbal autopsy data for high U5MR countries. We updated the estimation to use *Plasmodium falciparum* parasite rate in place of malaria index in the modelling of malaria deaths; to use adjusted empirical estimates instead of modelled estimates for China; and to consider the effects of pneumococcal conjugate vaccine and rotavirus vaccine in the estimation.

**Findings:**

In 2015, among the 5·9 million under-5 deaths, 2·7 million occurred in the neonatal period. The leading under-5 causes were preterm birth complications (1·055 million [95% uncertainty range (UR) 0·935–1·179]), pneumonia (0·921 million [0·812 −1·117]), and intrapartum-related events (0·691 million [0·598 −0·778]). In the two MDG regions with the most under-5 deaths, the leading cause was pneumonia in sub-Saharan Africa and preterm birth complications in southern Asia. Reductions in mortality rates for pneumonia, diarrhoea, neonatal intrapartum-related events, malaria, and measles were responsible for 61% of the total reduction of 35 per 1000 livebirths in U5MR in 2000–15. Stratified by U5MR, pneumonia was the leading cause in countries with very high U5MR. Preterm birth complications and pneumonia were both important in high, medium high, and medium child mortality countries; whereas congenital abnormalities was the most important cause in countries with low and very low U5MR.

**Interpretation:**

In the SDG era, countries are advised to prioritise child survival policy and programmes based on their child cause-of-death composition. Continued and enhanced efforts to scale up proven life-saving interventions are needed to achieve the SDG child survival target.

**Funding:**

Bill & Melinda Gates Foundation, WHO.

## Introduction

The year 2015 marks the end of the Millennium Development Goals (MDGs) era, during which the under-5 mortality rate (U5MR) reduced by an impressive 53% globally, although still falling short of the MDG 4 target of a two-thirds reduction from 1990 to 2015.^1^, ^2^

Year 2016 marks the beginning of the implementation of the Sustainable Development Goals (SDGs).^3^ The SDGs target an U5MR of no more than 25 per 1000 livebirths in every country of the world in 2030.^4^ To plan how to eliminate preventable child deaths, information is needed about the current distribution of causes of child deaths and this has changed in recent decades. In this paper, we update our annual estimates of child mortality by cause to 2000–15; reflect on progress toward the MDG 4; and consider the implications for national and global priorities if the SDG target for child survival is to be achieved.

## Methods

### General estimation approaches

We estimated the number of child deaths by cause for each of the 194 WHO member states for each year in 2000–15. This was done separately for neonates and children aged 1–59 months. The number of child deaths by cause was estimated as the product of the number of age-specific deaths due to all causes and age-specific and cause-specific mortality fractions. The age-specific all-cause death estimates were derived from age-specific child mortality estimates produced by the UN Inter-Agency Group for Child Mortality Estimation (UN-IGME).^5^ The livebirth estimates were produced by the UN Population Division.^6^

To generate cause-specific mortality fractions (CSMFs) for neonates and 1-59-month–olds, we applied our estimation framework with updates.^7^ The estimation framework comprises three components. Component one covers countries with adequate vital registration (VR) (67 for neonates, 69 for 1–59-month-olds) for which we used CSMFs derived from the country-specific VR data as is or with minor adjustments.^8^ Component two covers countries with inadequate VR and low U5MR (<35 per 1000 livebirths in 2000–15; 47 for neonates and 44 for 1–59-month-olds). For these countries, we modelled CSMFs using a multinomial logistic regression (MLR) applied to input CSMFs calculated from number of deaths by cause from VR countries (ie, component one) and their distal (eg, socioeconomic indicators) and proximate (eg, childhood life-saving intervention coverage values) determinates of child survival as model inputs.^9^ Component two is referred to as a VR based multicause model or VRMCM. Component three is for countries with inadequate VR and high U5MR (≥35 per 1000 livebirths in 2000–15; 80 for neonates and 81 for 1–59-month-olds). For these countries, we modelled CSMFs using MLR with empirical input CSMFs calculated from number of deaths by cause extracted from verbal autopsy (VA) studies and primarily proximate determinants as model inputs. Component three is referred to as a VA based multicause model or VAMCM. Details of the estimation framework including the use of MLR and information on the standard International Classification of Diseases codes by cause have been published elsewhere.^7^, ^8^ The distal and proximate determinants used as model inputs are listed in the appendix (p 5).

**Research in context**

**Evidence before this study**

Our study group, formerly referred to as the WHO and UNICEF's Child Health Epidemiology Reference Group (CHERG), has systematically reviewed, estimated, and published a series of child mortality by cause estimates since 2003, with the last publication presenting estimates for years 2000–13. To collect data published since 2013, we did an updated systematic review to identify quality child cause-of-death studies published between Jan 1, 2013, and Feb 12, 2015 in the following databases: PubMed, Embase, ISIS Web of Knowledge, Medline BIOSIS, Popline, WHOLIS (via Global Health Library, including the regional specific databases of LILACs, African Index Medicus, WPRIM, IMEMR, and PAHO) and IndMed without language limitation. Search strategies, search terms, and study inclusion and exclusion criteria were consistent with our previous studies. Other investigators have estimated distribution of mortality by cause among all age groups.

**Added value of this study**

In this paper, we updated the estimates from years 2000–13 to 2000–15 to reflect on the progress toward the MDG 4 and draw implications for the SDG child survival target. Our updates are based on substantially more input data and several important methodological advances, including using adjusted empirical instead of modelled child cause-of-death estimates for China for the first time.

**Implications of all the available evidence**

Estimates presented here are the most up-to-date, and likely thus far the most valid ones of child mortality by cause at the global, regional, and national levels. Such information can and should be used to inform child survival policy making and resource allocation. Future research should further consider how to best incorporate increasing national empirical estimates and balance between empirical and modelled estimates. Continued investment in child cause-of-death data collection and estimation applying innovative approaches will further improve validity of such important information.

We updated our database to include new VR data reported to WHO up to July 30, 2015, and new VA data identified through an updated systematic review of literature published between Jan 1, 2013, and Feb 12, 2015 (table 1; appendix p 3). Despite completing the systematic review in 2015, our estimates for year 2015 were intended to cover the entire year. In total, the input number of deaths increased by 43·0% for neonates (from 2·629 million to 3·760 million) and 22·7% for children aged 1–59 months (from 3·312 million to 4·063 million). For China, we replaced modelled estimates^10^ with adjusted empirical estimates from the China Maternal and Child Health Surveillance System (MCHSS), the details of which can be found elsewhere.^11^ A summary map of input data and estimation methods is presented in the appendix (p 3).

### Updates on estimation methods

A few methodological updates are shown here. First, we used the *Plasmodium falciparum* parasite rate (PfPR)^12^ in place of the more subjective malaria index^8^ as one of the candidate covariates to model the fraction of deaths due to malaria in countries with high transmission intensity. PfPR is the proportion of the population carrying asexual blood-stage parasites and is considered as an indicator of malaria transmission intensity (appendix p 4). In the VAMCM, we used post-hoc adjustment to consider the effects of recently scaled up interventions, which previously included insecticide treated bed nets (ITN). Since PfPR reflects the impact of ITN,^12^ we dropped ITN from the post-hoc adjustment. Second, we considered the impacts of pneumococcal conjugate vaccine^13^, ^14^ and rotavirus vaccine,^15^ in addition to the previously included *Haemophilus influenzae* type b (Hib) vaccine in the post-hoc adjustment. Specifically, we calculated pneumonia-specific, meningitis-specific, and diarrhoea-specific deaths averted due to each of these vaccines in the post-hoc adjustment and redistributed the cause-specific deaths averted to the remaining causes pro rata. The cause-specific deaths averted were calculated as the product of the following four quantities: 1) cause-specific deaths estimated by VAMCM before post-hoc adjustment, 2) the fractions due to vaccine-specific serotypes, 3) vaccine coverage, and 4) vaccine effectiveness, where available, or efficacy. Details of the parameters used in the post-hoc adjustment are available in the appendix (p 7). Lastly, a 7-year moving-average smoother was applied to the national-level prediction covariates used in the VAMCM to attenuate implausible spikes due to systematic errors in measurement or inconsistencies in covariate definitions.

### Model selection and uncertainty estimation

We selected the final model by cross validation. Specifically, we selected 10% of the observed cause-of-death data points, and fit each of the candidate models using the remaining 90%. We then predicted the CSMFs in the withheld selection, and determined the difference between the observed and predicted estimates. We repeated this process with 500 random subsets. We selected as final the model with the smallest average out of sample prediction error.^13^, ^16^

We estimated uncertainty in model coefficients by bootstrap resampling of input data sets from all estimation components and their respective distributions. This uncertainty was propagated through to the model predictions.^13^, ^17^ Uncertainty in the estimates of under-5 and neonatal deaths was also included using the UN-IGME methodology.^1^ We also accounted for potential variability due to post-hoc adjustment and the modelled number of deaths due to measles, pertussis, HIV, and malaria outside of sub-Saharan Africa. The 2·5 and 97·5 percentiles were taken as the lower and upper ranges of the uncertainty.

### Estimates reporting

Estimates of deaths in the 1–59 month period due to preterm birth complications, intrapartum-related events, and congenital abnormalities were produced previously, but were collapsed into the category of other. They are reported separately here. Notably, the number of deaths due to, for example, congenital abnormalities among under-5 is then the sum of the numbers of deaths due to congenital abnormalities among neonates and 1–59-month-olds. Other conditions among children aged 1–59 months include causes originated during the perinatal period, cancer, severe malnutrition, and other specified causes.

Since we estimated child mortality by cause for 2000–15 but not for 1990–99, we were not able to assess cause-specific progress toward the MDG 4 for the entire period of 1990–2015. However, we can benchmark cause-specific progress in 2000–15 with the 4·4% average annual rate of reduction (ARR) required to achieve the MDG 4.^8^, ^18^ We also present the aggregated cause-of-death profile by six U5MR strata using the 2015 estimates with the cutoffs of 10, 25, 50, 75, and 100 per 1000 livebirths, referred to as very low, low, medium, medium high, high, and very high mortality strata, respectively.^1^

To promote transparency and replicability of global health estimates, we have also included the GATHER reporting checklist in the appendix (p 9).^19^ Additional details of the input data, estimation methodology including statistical codes, and estimates are online and publicly available through the Maternal and Child Epidemiology Estimation's website.

### Role of the funding source

The funder of the study had no role in the study design, data collection, data analysis, data interpretation, or writing of the report. All authors had full access to all the data in the study and the corresponding author had final responsibility for the decision to submit for publication.

## Results

**Figure 1:**
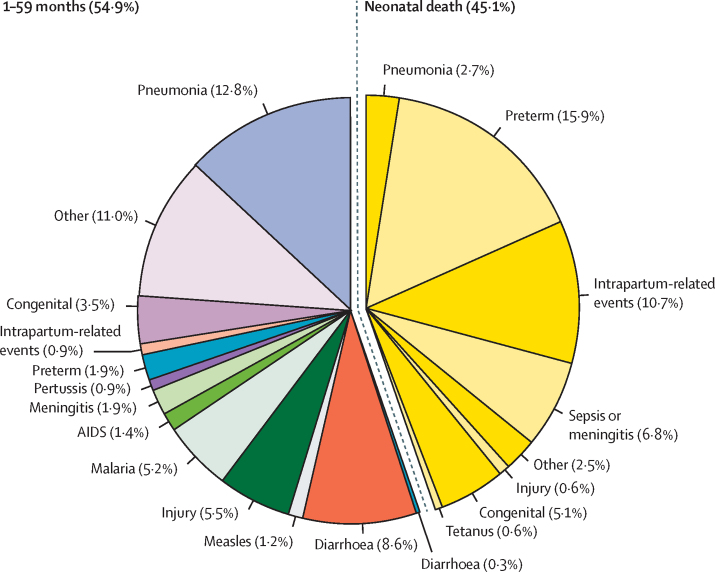
Global causes of under-5 deaths in 2015

In 2015, among the 5·941 million children who did not live to age 5 years, 2·681 million (45·1%) died in the neonatal period (figure 1). The leading causes of deaths in children under 5 were preterm birth complications (1·055 million [95% UR 0·935–1·179]; 17·8% [UR 15·4–19·0]), pneumonia (0·921 million [0·812–1·117], 15·5% [13·9–17·6]), and intrapartum-related events (0·691 million [0·598–0·778], 11·6% [9·9–12·7]; table 2). Among neonates, the leading causes were preterm birth complications (0·944 million [UR 0·832–1·066], 15·9% [UR 13·8–17·3]), intrapartum related events (0·637 million [0·550–0·723], 10·7% [9·2–11·8]), and sepsis or meningitis (0·401 million [0·280–0·522], 6·8% [4·7–8·6]). Among children who died in the 1-59-month period, the leading causes were pneumonia (0·762 million [UR 0·651–0·943], 12·8% [UR 11·5–14·6]), diarrhoea (0·509 million [0·401–0·661], 8·6% [7·0–10·2]), and injuries (0·327 million [0·272–0·410], 5·5% [4·6–6·3]).

Sub-Saharan Africa and southern Asia remained the MDG regions with the highest numbers of under-5 deaths in 2015 (2·947 million and 1·891 million, respectively). The distribution of under-5 deaths by cause differed substantially by region (appendix p 11). For example, in sub-Saharan Africa, the leading causes of under-5 deaths were pneumonia (0·490 million [UR 0·417–0·631], 16·6% [UR 14·8–19·1]), preterm birth complications (0·356 million [0·283–0·433], 12·1% [9·3–13·6]), and intrapartum-related events (0·338 million [0·278–0·397], 11·5% [9·0–12·3]). Southern Asia, however, had a higher fraction of neonatal deaths (57·0%), with preterm birth complications being the leading cause (0·478 million [0·394–0·552], 25·3% [21·7–28·7]).

Among the 194 countries estimated, the number of under-5 deaths varied between 1 death and 1·201 million deaths in 2015. The ten countries with the highest number of under-5 deaths were collectively responsible for three-fifths (60·4%, 3·587 million) of the global under-5 deaths. Their cause composition is presented in the appendix (p 12). The share of neonatal deaths in these countries varied from 30·9% (0·094 of 0·305 million in DR Congo) to 62·3% (0·074 of 0·119 million in Bangladesh). The leading cause among under-5s was pneumonia in Angola (0·029 million [UR 0·011–0·064], 17·4% [UR 13·8–21·9]), DR Congo (0·046 million [0·026–0·074], 15·2% [12·8–17·9]), Ethiopia (0·031 million [0·012–0·060], 17·1% [14·2–20·7]), Nigeria (0·133 million [0·087–0·209], 17·8% [15·9–20·6]), and Tanzania (0·014 million [0·007–0·028], 14·6% [12·8–17·5]). All of these countries are in sub-Saharan Africa. The leading cause was preterm birth complications in Bangladesh (0·024 million [UR 0·018–0·031], 19·8% [UR 15·6–24·0]), Indonesia (0·028 million [0·023–0·038], 18·9% [16·3–23·3]), India (0·330 million [0·269–0·387], 27·5% [23·4–31·5]), and Pakistan (0·102 million [0·071–0·137], 23·6% [18·4–28·7]). Most of these countries are in southern Asia. The leading cause was congenital abnormalities in China (0·036 million [UR 0·034–0·039], 19·7% [UR 18·0–21·5]). Malaria was an important cause in DR Congo and Nigeria, responsible for 0·036 million ([UR 0·017–0·062; 11·9% [UR 8·2–16·5]) and 0·102 million ([0·056–0·186]; 13·6% [10·2–17·8]) deaths, respectively.

The risk of dying in the first 5 years, the U5MR, ranged between 1·9 and 155·1 per 1000 livebirths among the 194 countries in 2015. The ten countries with the highest U5MR are all in sub-Saharan Africa and had U5MRs above 90 per 1000 livebirths. Three of these ten countries (Angola, Nigeria, and DR Congo) are among the ten countries with the most under-5 deaths mentioned above. In the remaining seven countries, the leading cause among under-5s was pneumonia in Benin, Central African Republic, Equatorial Guinea, Somalia, and Chad, and malaria in Mali and Sierra Leone (appendix p 13). Additional estimates of country-specific and cause-specific numbers of deaths are available in the appendix (p 14).

**Figure 2:**
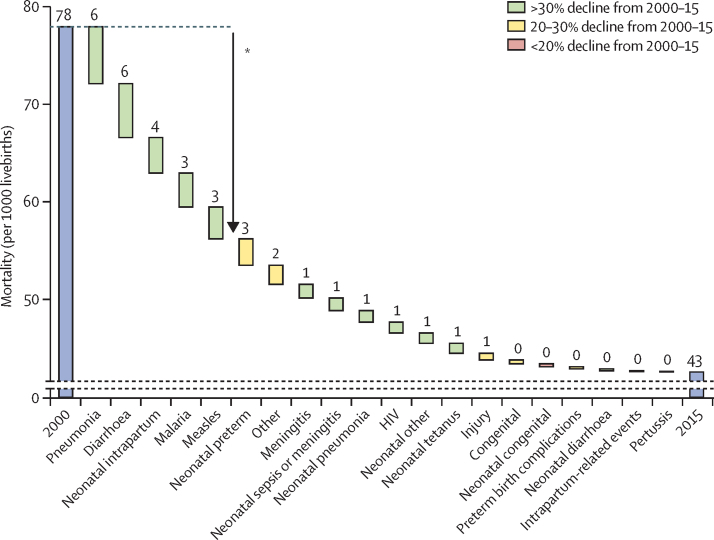
Global trends in cause-specific mortality rates in neonates and children aged 1–59 months, 2000–15 *About 61% of the reduction comes from pneumonia, diarrhoea, malaria, and measles among 1-59-month olds and neonatal intrapartum related events.

Globally, more than 4 million (4·020 million) fewer under-5 deaths occurred in 2015 compared with in 2000. During this period, causes of child deaths changed gradually at the global level (appendix p 45). Although pneumonia and preterm birth complications were also leading causes of under-5 deaths in 2000 (1·738 million [UR 1·654–1·997]; 17·4% [UR 16·0–19·3] and 1·339 [1·169–1·464]; 13·4% [11·6–14·2], respectively), diarrhoea was replaced as the third leading cause in 2000 (1·213 million [1·115–1·451], 12·2% [11·0–14·3]) by intrapartum-related events in 2015. U5MR declined from 77·8 to 42·5 per 1000 livebirths over this period. Mortality rates for pneumonia, diarrhoea, neonatal intrapartum related events, malaria, and measles all reduced by more than 30% (figure 2). Collectively, reductions in these causes (21·7 less deaths per 1000 livebirths) were responsible for 61·6% of the total reduction in U5MR (35·3 less deaths per 1000 livebirths) in 2000–15.

The global ARR of U5MR in 2000–15 was 4·0%, below the 4·4% required to achieve the MDG 4 in 1990–2015. All-cause neonatal mortality has been declining at a slower rate than that of children aged 1–59 months, at 3·1% versus 4·7% respectively. As a result, the fraction of neonatal deaths increased from 39·3% in 2000 to 45·1% in 2015. Nine causes achieved an ARR of at least 4·4% since 2000, with ARRs ranging from 13·1% [UR 6·8–16·1] for measles to 4·6% [3·4–5·4] for neonatal pneumonia (appendix p 46). By comparison, neonatal mortality due to congenital abnormalities only declined by 0·8% per year (UR 0·1–2·0).

Among the ten MDG regions, eastern Asia, which is composed mainly of China, saw the fastest reduction in U5MR in 2000–15 at an ARR of 8·2%. Mortality rates of almost all causes in this region have declined by at least 70% from 37·1 to 10·8 per 1000 livebirths (appendix p 49). The cause composition also changed, with congenital abnormalities (0·038 million [UR 0·036–0·041], 19·3% [UR 17·8–21·0]) and injuries (0·028 [0·027–0·030], 14·2% [13·0–15·3]) replacing pneumonia (0·024 [0·022–0·026], 20·1% [18·1–22·3]) and intrapartum-related events (0·028 million [0·023–0·030], 15·7% [14·2–17·3]) as the leading causes among under-5s in 2015 (appendix p 59). In sub-Saharan Africa, malaria, diarrhoea, and measles among children aged 1–59 months saw substantial reductions, contributing 18, 13, and 11 per 1000 livebirths to the reduction of U5MR, respectively (appendix p 55). The leading cause changed from malaria (0·699 million [UR 0·612–0·960], 16·4% [UR 13·5–20·7]%) in 2000 to pneumonia (0·490 million [0·417–0·631], 16·6% [14·8–19·1]%) in 2015 (appendix p 65). In southern Asia, U5MR due to preterm birth complications have only been declining at an ARR of 1·4% per annum (UR −0·3 to 2·2), a rate slower than the regional ARR of U5MR at 3·8%. As a result, the contribution of preterm birth complications to under-5 deaths has increased from 16·7% (UR [14·2–18·6], 0·598 million [0·497–0·664]) in 2000 to 25·3% ([21·7–28·7], 0·478 million [0·394–0·552]) in 2015. Despite a faster decline at an ARR of 5·4% [UR 3·7–7·3], pneumonia remained one of the leading killers of under-5 children in southern Asia, responsible for 0·687 million (0·626–0·771) deaths (19·2% [UR 17·7–21·6]) in 2000 and 0·285 million (0·215–0·372) deaths (15·1% [13·4–17·4]) in 2015. Additional regional and national trends can be viewed in the appendix (p 14).

**Figure 3:**
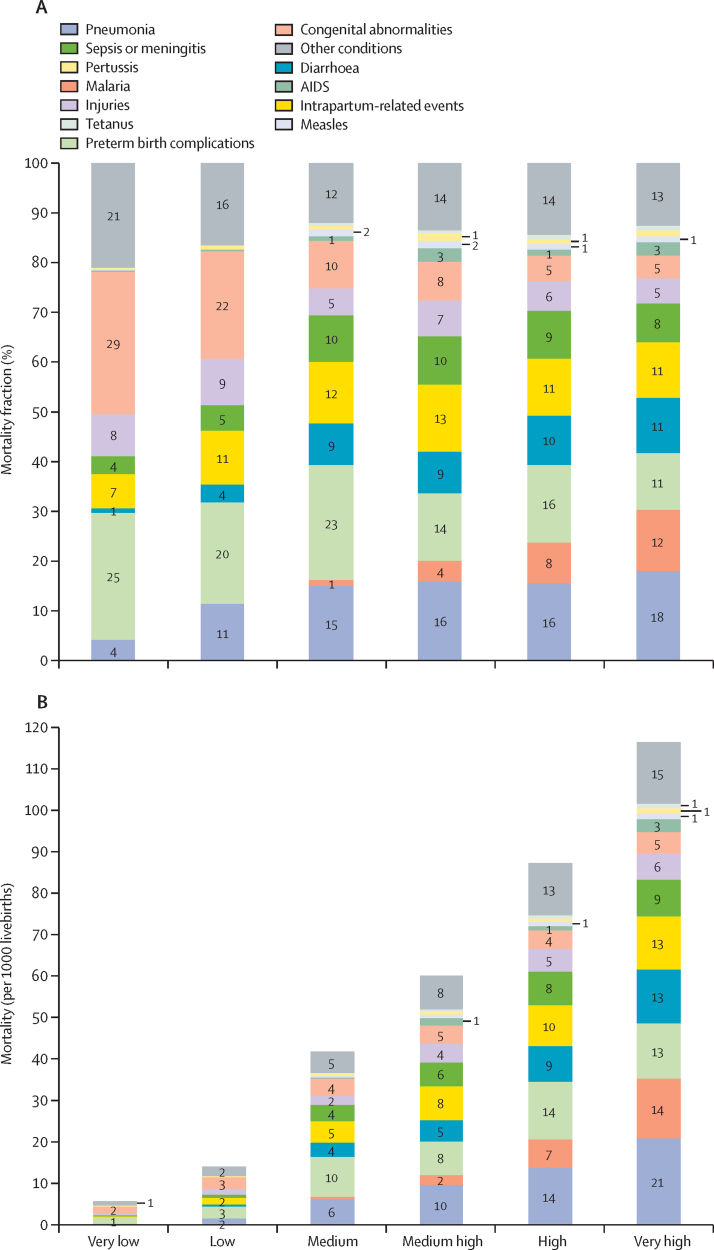
Cause-of-death mortality fractions (A) and cause-specific mortality rates (B) by U5MR strata, 2015 U5MR=under-5 mortality rate. U5MR strata are defined as very low (<10 per 1000 livebirths), low (10–<25 per 1000 livebirths), medium (25–<50 per 1000 livebirths), medium high (50–<75 per 1000 livebirths), high (75–<100 per 1000 livebirths), and very high (≥100 per 1000 livebirths). Values less than 1 are not labelled.

Figure 3 shows the CSMFs and cause-specific mortality rates by six U5MR strata in 2015. Seven (Angola, Central African Republic, Chad, Mali, Nigeria, Sierra Leone, and Somalia) of the 194 countries fell in the very high mortality stratum. Collectively, they were responsible for a fifth (20·1%, 1·193 million of 5·942 million) of global under-5 deaths in 2015. Pneumonia, malaria, and diarrhoea were the leading causes in this stratum. The high, medium high, and medium strata each included 17, 20, and 36 countries. They were responsible for about a quarter (23·7%), a tenth (11·0%), and a third (34·9%) of global under-5 deaths in 2015, respectively. The leading causes in these three strata were very similar, being preterm birth complications, pneumonia, and intrapartum-related events. 54 countries were in the low mortality stratum. They contributed to 9% of the world's under-5 deaths. Their leading causes were congenital abnormalities, preterm birth complications, pneumonia, and intrapartum-related events. Another 60 countries fell in the very low mortality strata, responsible for 2% of the global under-5 deaths. The leading causes in this stratum were congenital abnormalities, preterm birth complications, and injuries. When moving from the very low to the very high mortality strata, the fractions and mortality rates of pneumonia, diarrhoea, and malaria increase. By contrast, the fractions of congenital abnormalities decrease, although its mortality rate is still the lowest in the very low mortality stratum (figure 3B).

## Discussion

Child survival has improved substantially in the MDG era even though the targeted two-thirds reduction was not achieved.^1^, ^20^ This progress has been partly credited to the establishment of the MDGs, the ensuing increase in official development assistance, and the consequential scaling up of many life-saving interventions.^20^, ^21^, ^22^ However, the progress has been uneven and high levels of child mortality persist in many countries.

In regard to cause-specific pace of reduction in 2000–15, measles and neonatal tetanus have seen tremendous progress, as have major causes such as diarrhoea and pneumonia though to a lesser degree. Injuries, neonatal preterm birth complications, neonatal intrapartum-related events, neonatal congenital abnormalities, and neonatal sepsis or meningitis are major causes among those with insufficient decline (ARR <4·4%) in 2000–15. To achieve the SDG child survival target, substantial progress is needed for these causes.

Neonatal mortality has declined more slowly than that of the 1–59-month-olds. If neonatal causes had been declining at a rate achieved by the 1–59 month age group, the world would have attained the MDG 4 target ahead of time. Eastern Asia is an exception in that it managed to achieve faster decline among neonates than among older children. Case studies are valuable on how eastern Asia, primarily China, has achieved an impressive and balanced decline across age groups and causes.^11^ By contrast, sub-Saharan Africa had the largest disparity in progress between the two age groups, with neonatal survival only having been improving at an ARR of less than half of that of the 1–59 month age group. Five of the 12 post-neonatal causes achieved an ARR of at least 4·4%, yet only two of the eight neonatal causes did in this region.

Focusing on the high-burden regions, sub-Saharan African countries had a quarter of the world's livebirths in 2015.^6^ This figure is projected to increase to a third in 2030.^6^ Ensuring family planning needs of adolescent girls, women, and couples are satisfied with modern contraception is a key to reduce the number of child deaths in this region and globally in the next 15 years.^21^ Major infectious causes in sub-Saharan Africa had reductions in 2000–15, but infectious causes such as pneumonia, diarrhoea, malaria, and sepsis or meningitis remain important and should be a focus of child survival efforts going forward. Many neonatal causes and injuries have been declining more slowly in this region and interventions should be enhanced to address these conditions. In southern Asia, the contributions of preterm birth complications and congenital abnormalities to under-5 deaths have increased substantially in 2000–15. A major focus of child survival programmes in this region has to be on neonatal causes.

Out of equity considerations, the SDG child survival target calls on all countries to reduce U5MR to 25 per 1000 livebirths or below by 2030. Country strategy formulation should consider their current U5MR and cause-of-death profile. When prioritising by child mortality strata, for the very high mortality countries, the focus should still be on the leading infectious causes, such as pneumonia, malaria, and diarrhoea. All of these can be addressed by highly effective and low cost preventive and therapeutic interventions, such as breastfeeding promotion, and *Haemophilus influenzae* type b and pneumococcal vaccines for pneumonia,^23^ improved water and sanitation, rotavirus vaccine, zinc supplementation, oral rehydration solutions, and community case management for diarrhoea,^23^ and insecticide treated bed-nets, intermittent preventive treatment in pregnancy, and artemisinin-based combination therapy for malaria.^24^ In addition, recent approval of a malaria vaccine holds potential for further malaria reduction, although major challenges exist for vaccine schedules.^25^

Among countries with high, medium high, and medium child mortality, a clear child survival policy and programme focus should be to further invest in reducing deaths due to preterm birth complications, pneumonia, and intrapartum-related events. Relevant interventions for pneumonia are described above. Improved labour and delivery management is also important to reduce the causes of neonatal deaths.^26^ Antenatal corticosteroids and Kangaroo mother care are among the major recommended interventions to improve preterm birth outcomes.^27^ Neonatal resuscitation and comprehensive emergency obstetric care are among those recommended to reduce deaths due to intrapartum-related events.^28^, ^29^, ^30^

114 countries already have an U5MR of no more than 25 per 1000 livebirths in 2015.^1^ Effectively, they have achieved the SDG child survival target. However, this does not mean that they have homogeneous child cause-of-death profiles. Different from their higher mortality peers, countries in the low mortality stratum have congenital abnormalities as the leading cause. Clearly, congenital abnormalities should receive special attention in this stratum. Reducing the burden of congenital abnormalities will require better detection of some conditions and surgery for many. In addition to preterm birth complications and intrapartum-related events, pneumonia is still relatively important among low mortality countries compared with their very low mortality counterparts.

For countries in the very low mortality stratum, there is still room for improvement. Rapid reductions were seen between 2000 and 2015 for countries in this mortality stratum. For example, Portugal and Czech Republic had an U5MR around seven per 1000 livebirths in 2000. They both achieved an ARR of 4·5% for U5MR in 2000–15. Cause wise, they both had an accelerated decline, for example, in neonatal intrapartum-related events, which could be due to improved delivery care. These set examples for countries with very low child mortality to achieve rapid cause-specific reduction. In addition to congenital abnormalities, injuries also become increasingly important in this stratum. More rigorous research is needed to understand the epidemiology and effectiveness of injury interventions, such as barriers to prevent drowning, safer stoves to prevent burns, and car seats to prevent road traffic injury.^31^ Coordination and cooperation across sectors are essential. Injuries and other childhood conditions in settings of conflict and humanitarian crisis, such as Syria, should be a priority for global assistance.^32^

In addition to newly implemented vaccines, other new interventions could bear the potential to further improve child survival. New WHO guidelines on antibiotic management of neonatal infections have been released based on the results of the Simplified Antibiotic Therapy Trial.^33^, ^34^, ^35^, ^36^ These guidelines could further encourage community treatment and reduce mortality from neonatal infections. Based on preliminary results from the Strategic Timing of Antiretroviral Treatment study, which was done among adults,^37^ WHO revised its HIV treatment recommendation to “treat-all”, that is to treat anyone living with HIV with antiretroviral as soon as possible and to offer people at high risk with preventive antiretrovirals.^38^ This strategy could further avert under-5 HIV/AIDS deaths, although more evidence for children is needed. During the scale-up of antiretroviral treatment, precautions need be exercised to ensure that existing childhood interventions are not crowded out.^39^

We warn against attributing reductions of child cause-specific mortality rates to covariates used in our estimation due to circularity. For example, accelerated decline estimated in pneumonia, meningitis, and diarrhoea mortality, particularly in the past few years, is in part the result of directly taking account of the expected effect of *Haemophilus influenzae* type b vaccine, pneumococcal conjugate vaccine, and rotavirus virus vaccines in the post-hoc adjustment.

Despite an increasing number of VA study data points and two additional countries now included as adequate VR countries, the data gap remains large for high burden countries and regions where 90% of under-5 deaths still occur in countries estimated by VAMCM, yet only 3% occur in countries with adequate VR in 2015 (appendix p 3). To improve internal validity of VA, innovative approaches such as the minimally invasive tissue sampling (MITS) can be applied.^40^ The establishment of the Child Health and Mortality Prevention Surveillance Network^41^ applying the MITS technique is welcome, although understanding the external validity of these approaches and estimates is crucial. In high burden countries where systematic collection of cause of death information lacks yet resources and technical capacity could become sustainably available, sample registration system should be attempted. Initiatives, such as the Countrywide Mortality Surveillance for Action by the Bill and Melinda Gates Foundation, can be useful efforts.

Our uncertainty ranges do not fully capture all the associated uncertainties. For example, we have not taken into consideration uncertainty associated with model inputs or covariates. The estimates for 2015 are particularly uncertain, because they were prepared using model inputs available up to the end of August, 2015. At the time, the most recent empirical estimates of model inputs were only available for up to year 2014, and those for 2015 were either assumed the same as those in 2014 or modelled through simplistic approaches. This could overestimate the burden of causes for which effective interventions are being rapidly scaled up. For example, in countries where pneumococcal conjugate vaccine and rotavirus vaccine are being rapidly scaled up, we might have overestimated the burden of pneumonia, meningitis, and diarrhoea in 2015 by assuming that the coverage of pneumococcal conjugate vaccine and rotavirus vaccine was the same in 2015 as in 2014. On the other hand, applying effectiveness and sometimes efficacy parameters from trials could have overestimated the life-saving effects of interventions, resulting in underestimated pneumonia, meningitis, and diarrhoea deaths. To what extent the above two sources of biases cancel each other out is unknown. Additional uncertainty is likely to be associated with U5MR and number of all-cause death estimates in the past few years as fewer empirical data points are available compared to earlier periods.^42^ Therefore, continued evaluation and reflection on MDG 4 beyond 2015 are important.

In the next round, we plan to apply methods to better incorporate countries' transition from VAMCM to VRMCM, and those from VRMCM to VR. We will also investigate methods to better incorporate national empirical data recently collected from low-income and middle-income countries into our modelled estimates. Additional research is under way to better synthesise region-specific efficacy and effectiveness of pneumococcal conjugate vaccine as inputs to the post-hoc adjustment.

Much has been accomplished on child survival in 1990–2015, and particularly since 2000. However, accelerated investment in child survival is imperative post-2015 to achieve the SDG child survival target. US$25 billion have been pledged by governments over the next 5 years to improve the health of women, children, and adolescents.^43^ With the pledged resources and hopefully more to come, concerted efforts are needed across disease control and prevention programmes to maintain progress for countries which have accomplished rapid decline, and to accelerate progress for those that have had slower reductions in the past. Progress on child survival has benefited from a cohesive action plan in achieving the MDG 4 in the past two decades. United and continued actions are needed to achieve the SDG child survival target by 2030 and end preventable child deaths in a generation.^44^

For **Maternal and Child Epidemiology Estimations** see http://tinyurl.com/Hopkins-MNCH-cod-openaccess

## Figures and Tables

**Table 1:** New and total input data by estimation methods

	**New input data**			**Total input data**		
	Data points	Deaths	Countries	Data points	Deaths	Countries
**Neonates**						
VR	298	355 666	58	1628	1 621 273	66
VRMCM	674	772 904	64	2004	2 038 511	66
VAMCM	12	1897	5	124	100 119	37
**1–59-month-olds**						
VR	166	412 925	69	1104	2 043 763	69
VRMCM	147	288 535	58	1364	1 646 909	68
VAMCM	90	49 214	18	218	372 324	42

VR=vital registration. VRMCM=VR based multi-cause model. VAMCM=VA based multi-cause model.

**Table 2:** Estimated numbers of deaths by cause and cause-specific mortality rate in 2015

	**Estimated number (UR; millions)**	**Cause specific mortality rate (per 1000 livebirths)**
**Children aged 0–59 months**		
Preterm birth complications	1·055 (0·935–1·179)	7·556 (6·696–8·442)
Pneumonia	0·921 (0·812–1·117)	6·594 (5·815–7·996)
Intrapartum-related events	0·689 (0·598–0·778)	4·934 (4·283–5·569)
Diarrhoea	0·526 (0·418–0·691)	3·768 (2·992–4·950)
Sepsis/meningitis	0·517 (0·408–0·647)	3·699 (2·922–4·634)
Congenital abnormalities	0·512 (0·455–0·606)	3·666 (3·256–4·338)
Other conditions	0·841 (1·602–2·051)	6·020 (11·467–14·682)
**Neonates aged 0–27 days**		
Preterm birth complications	0·943 (0·832–1·066)	6·753 (5·959–7·632)
Intrapartum-related events	0·631 (0·550–0·723)	4·520 (3·937–5·177)
Sepsis/meningitis	0·402 (0·280–0·522)	2·875 (2·005–3·739)
Congenital abnormalities	0·305 (0·260–0·382)	2·183 (1·861–2·735)
Pneumonia	0·161 (0·111–0·239)	1·154 (0·793–1·713)
Tetanus	0·034 (0·018–0·084)	0·243 (0·129–0·600)
Diarrhoea	0·018 (0·010–0·070)	0·126 (0·072–0·504)
Other conditions	0·187 (0·142–0·240)	1·337 (1·017–1·719)
**Children aged 1–59 months**		
Pneumonia	0·760 (0·651–0·943)	5·441 (4·661–6·752)
Diarrhoea	0·509 (0·401–0·661)	3·642 (2·872–4·730)
Injuries	0·326 (0·272–0·410)	2·337 (1·944–2·938)
Malaria	0·306 (0·225–0·452)	2·193 (1·613–3·237)
Congenital abnormalities	0·207 (0·165–0·259)	1·482 (1·178–1·851)
Meningitis	0·115 (0·091–0·162)	0·824 (0·652–1·157)
Preterm birth complications	0·112 (0·061–0·168)	0·802 (0·436–1·202)
AIDS	0·086 (0·076–0·101)	0·614 (0·541–0·722)
Measles	0·074 (0·038–0·268)	0·529 (0·274–1·920)
Intrapartum-related events	0·058 (0·028–0·092)	0·414 (0·203–0·657)
Pertussis	0·054 (0·053–0·060)	0·387 (0·377–0·427)
Other conditions	0·654 (0·536–0·803)	4·683 (3·835–5·752)

Uncertainty range (UR) is defined as the 2·5–97·5 centile· Other conditions among children aged 1–59 months included causes originated during the perinatal period, cancer, severe malnutrition, and other specified causes. Intrapartum-related events were formerly referred to as “birth asphyxia”.
