# Department of Error

**DOI:** 10.1016/S0140-6736(17)31212-6

**Published:** 2017-05-13

*Liu L, Oza S, Hogan D, et al. Global, regional, and national causes of under-5 mortality in 2000–15: an updated systematic analysis with implications for the Sustainable Development Goals.* Lancet *2016;* **388:** *3027–35*—In the first sentence of the Results in the Article, the number of children who did not live to age 5 years should have been 5·942 million. Data in table 2 have been updated. These corrections have been made to the online version as of May 11, 2017.

