# Supplementary material for: Global, regional, and national causes of under-5 mortality in 2000–15: an updated systematic analysis with implications for the Sustainable Development Goals
Source: Lancet. 2016 Dec 17;388(10063):3027–35. doi: 10.1016/S0140-6736(16)31593-8 (PMC5161777; doi:10.1016/S0140-6736(16)31593-8)
Supplement: Supplementary appendix [file mmc1.pdf]

THE LANCET

Supplementary appendix

This appendix formed part of the original submission and has been peer reviewed. We post it as supplied by the authors.

Supplement to: Liu L, Oza S, Hogan D, et al. Global, regional, and national causes of under-5 mortality in 2000–15: an updated systematic analysis with implications for the Sustainable Development Goals. *Lancet* 2016; published online Nov 10. http://dx.doi.org/10.1016/S0140-6736(16)31593-8.

Global, regional and national causes of under-5 mortality in 2000-2015 – an updated systematic analysis with implications for the Sustainable Development Goals

Li Liu,[1,2] Shefali Oza,[3] Dan Hogan,[4] Yue Chu,[2] Jamie Perin,[2] Jun Zhu,[5,6] Joy Lawn,[3] Simon Cousens,[3] Colin Mathers,[4] Robert E. Black[2]

[1]Department of Population Family and Reproductive Health, Johns Hopkins Bloomberg School of Public Health, Baltimore, Maryland
[2]The Institute for International Programs, Department of International Health, Johns Hopkins Bloomberg School of Public Health, Baltimore, Maryland
[3]London School of Hygiene and Tropical Medicine, London, UK
[4]Department of Health Statistics and Informatics, World Health Organization, Geneva, Switzerland
[5]National Office of Maternal and Child Health Surveillance, Chengdu, China
[6]Key Laboratory of Birth Defects and Related Diseases of Women and Children (Sichuan University), Ministry of Education, Chengdu, Sichuan, China

Correspondence to: Dr. Li Liu, Department of Population, Family and Reproductive Health, and the Institute for International Programs, Department of International Health, Johns Hopkins Bloomberg School of Public Health, 615 N Wolfe Street, Baltimore, MD 21205, USA lliu26@jhu.edu

Supplementary webappendix

Webappendix 1. Details of updated input data, modeling methods, and posthoc adjustment

Webappendix 2. GATHER checklist

Webappendix 3. Cause-specific mortality fractions (CSMFs) by MDG regions in 2015

Webappendix 4. CSMFs of the top 10 countries with the highest number of under-five deaths in 2015

Webappendix 5. CSMFs of the 10 countries with the highest U5MR in 2015

Webappendix 6. Estimated numbers of deaths by cause in neonates, children aged 1-59 months at the global, regional and country levels in 2000-2015

Webappendix 7. Global CSMFs in 2000-2015

Webappendix 8. Global cause-specific average annual rate of reduction in 2000-2015

Webappendix 9. Trends in cause-specific mortality rates in neonates and children aged 1–59 months by the MDG regions, 2000–2015

Webappendix 10. CSMFs by the MDG regions, 2000-2015

Webappendix 1. Details of updated input data, modeling methods, and posthoc adjustment

Updated systematic review

Input verbal autopsy (VA) studies used for VAMCM were updated. We searched literature published between January 1, 2013 and February 3, 2015 for neonates and between May 28, 2013 and February 12, 2015 for the 1-59 months age group.

Similar search strategies and exclusion/inclusion criteria as the previous estimation were applied and details can be found elsewhere.[1,2] We used DistillerSR,[3] an online systematic review software by Evidence Partners LLC, for literature screening, data abstraction and adjudication for systematic review.

In addition to published studies identified through systematic review, we included three sets of recent and known VA studies. They are the International Network for the Demographic Evaluation of Populations and their Health (INDEPTH) studies,[4] the Verbal Autopsy Social Autopsy (VASA) study,[5] and the India Million Death Study (MDS) 2004-2006.[6] Figure S1.1 below shows the number of input data points by estimation methods.

Figure S1.1 Number of input data points by estimation methods

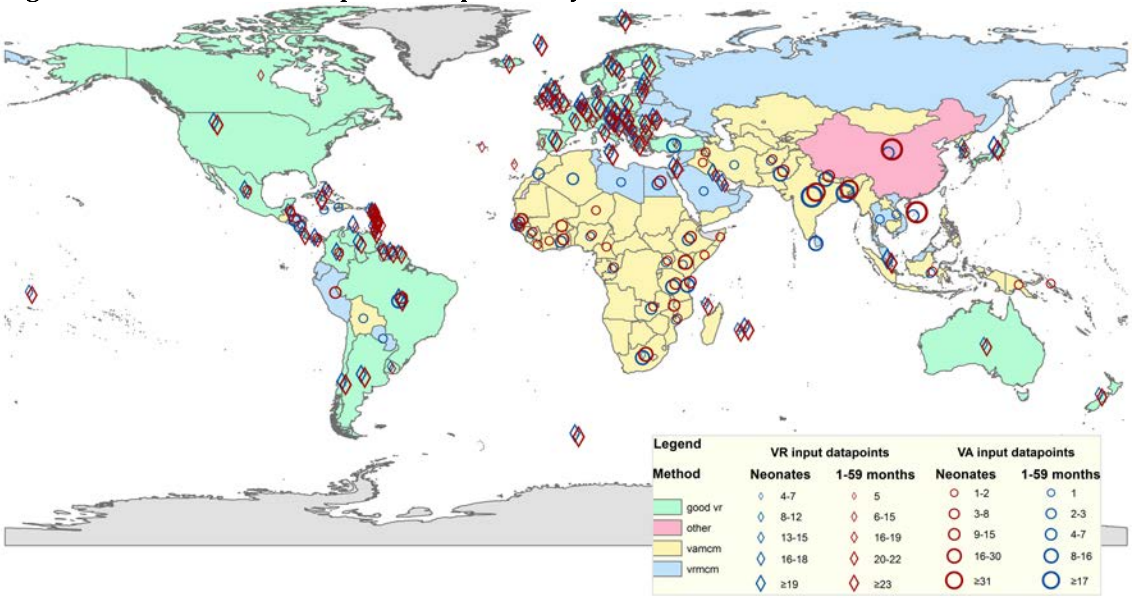

VR: vital registration; VA: verbal autopsy; VAMCM: verbal autopsy based multi-cause model; VRMCM: vital registration based multi-cause model

<u>INDEPTH</u>

INDEPTH published cause-of-death data from 21 Health and Demographic Surveillance Sites (HDSSs) across Africa and Asia from 1992 to 2012.[7] We used these data for modeling cause-specific mortality fractions (CSMFs) among children aged 1 to 59 months. The same exclusion criteria used in the systematic review were applied.[1,2] In addition, three sites (Purworejo in Indonesia, Farafenni in the

Gambia, and Vadu in India) were excluded due to quality concern.[4] To determine the final study points, we first collapsed years when less than 25 deaths were reported to adjacent years within the same sites. We further collapsed years within sites while considering the availability of covariates (e.g. those from the Demographic and Health Surveys or Multiple Indicator Cluster Surveys) and the reference periods of the corresponding surveys to ensure compatibility between cause-of-death study points and site-specific covariates to our best ability. At the end, 35 study points from 16 HDSSs across 9 Africa and Asia countries were included, representing 12,970 deaths among children aged 1-59 months.

Use *plasmodium falciparum* parasite rate in place of malaria index in the modeling of malaria

The estimates of *Plasmodium falciparum* parasite rate (PfPR) were derived from the most recent global map of *Plasmodium falciparum* malaria endemicity by the Malaria Atlas Project (MAP), using a model-based geostatistical prediction that created a continuous surface of PfPR for every 5 km by 5 km pixel within the stable limits of *Plasmodium falciparum* malaria transmission.[8-11]

At the study level, site-year-specific values were obtained from the MAP global surface data for study sites with the highest possible resolution. If the study site was larger than one 25 km^2, the mean from the entire study site was taken. If the study period was more than one year, we used average PfPR over time. Eight VA studies reported malaria deaths yet their corresponding PfPR was estimated to be zero. We used national PfPR values in this case to account for the possibility that those cases occurred among migrants lived in the study sites.

For national level PfPR used for the prediction of CSMFs, we smoothed PfPR using a 7-year-moving-average for countries in Africa with PfPR available from MAP. For countries without PfPR available from MAP, we used the most recent global surface from MAP and assumed constant value for 2000-2015. For countries with PfPR estimated to be zero, we adjusted cause-specific number of deaths during posthoc adjustment to ensure zero malaria deaths in these countries. Table S1.1 shows the set of covariates used in multinomial logistic regression by age group.

Table S1.1 Covariates used in multinomial logistic regression by age group

Cause	VRMCM	VAMCM
**Early neonatal**		
Preterm	(as base)	BCG; FemLit; GFR; LBW; SBA; PAB
Intrapartum	FemLit	(as base)
Congenital	IMR	Period; U5MR
Pneumonia	GINI; GNI; U5MR	Period; LBW
Sepsis	GINI; IMR	BCG; Period; LBW
Other	GINI; ANC; LBW; FemLit; IMR	BCG; LBW; Region
Injuries	GINI; GNI; U5MR; IMR	N/A
Diarrhoea	N/A	Period; NMR; Region; IMR; GFR
Tetanus	N/A	PAB; NMR; ANC; Period; BCG
**Late neonate**		
Preterm	(as base)	GFR; LBW; SBA; NMR; U5MR
Intrapartum	FemLit	(as base)
Congenital	FemLit; U5MR	Period; SBA; U5MR; Region
Pneumonia	ANC; U5MR; GFR; NMR	BCG; SBA
Sepsis	GINI; LBW; NMR	ANC; FemLit; Period; PAB
Other	ANC; LBW; FemLit; NMR	PAB; ANC; BCG; Region
Injuries	GINI; U5MR	N/A
Diarrhoea	N/A	LBW
Tetanus	N/A	Period; NMR; Region
**1-59 months**		
Pneumonia	(as base)	(as base)
Diarrhoea	Region; U5Pop; DPT3; GNI	Year; U5MR; Urban
Meningitis	Region; year; HDI; Hib3	GNI; MCV; Meningitis epidemic
Perinatal	Region; Hib3; DPT3; U5MR; SBA; Improved water source	Period; Underweight
Other I	Region; HDI; Hib3; DPT3; SBA	SBA
Other NCD	Region; HDI; U5MR; Urban; Education index; GNI	SBA
Congenital	Region; HDI; Hib3; U5MR; Urban; Education index	Period; Underweight
Injuries	Region; HDI; SBA; Education index; Improved water source; U5Pop	U5MR
Malaria	N/A	PFPR

Abbreviations: NCD: non-communicable diseases; N/A: not applicable; FemLit: female literacy; IMR: infant mortality rate; NMR: neonatal mortality rate; U5MR: under five mortality rate; GINI: Gini coefficient; GNI: gross national income per capita; ANC: antenatal care; LBW: low birth weight rate; GFR: gross fertility rate; SBA: deliveries assisted by skilled birth attendants; PAB: Neonates protected at birth against neonatal tetanus; BCG: Bacillus

Calmette–Guérin vaccine coverage; DPT3: Diphtheria-tetanus-pertussis vaccine third dose immunization coverage; U5Pop: under five population; HDI: human development index; HIB3: Haemophilus influenzae type b third dose immunization coverage; PFPR: Plasmodium falciparum parasite rate.
* Note: actual covariates used in the model could be transformed (e.g. log, square root, exponentiation etc.) or used as dummy variables (e.g. period, region etc.) for the best fit. Detailed model equations could be found in the open access file: http://tinyurl.com/Hopkins-MNCH-cause-of-death.

Updated posthoc adjustment

We performed post-hoc adjustment to further account for the effect of *Pneumococcal* vaccine (PCV) and *Haemophilus* influenza type B vaccine (Hib) on pneumonia and meningitis deaths, and the effect of rotavirus vaccine (Rota) on diarrhea deaths after modeling CSMFs for children aged 1-59 months using VAMCM.

There have been three types of PCV vaccine formulations, PCV7, PCV10 and PCV13. Different formulations cover different serotypes and different proportions of invasive pneumococcal disease (IPD) due to serotype variability across regions.[12] The actual coverage for PCV vaccine was calculated using the following equation:

$$\mathrm{C}_{pcv} = \sum (D_{formulation} \times C_{formulation})$$

where C_{pcv} denotes coverage for PCV based on vaccine formulation. $D_{formulation}$ represents region-specific percentage of IPD due to serotypes in each vaccine formulation. And $C_{formulation}$ is the country-and-formulation-specific PCV coverage.

We calculated the numbers of cause-specific deaths due to pneumonia, meningitis and diarrhea that could have been averted by PCV, Hib and Rota separately. The numbers of deaths averted were calculated using the following equation:

$$CSN_{averted} = CSN_{modeled} \times SSP_{serotype} \times \mathrm{C} \times \mathrm{Eff}$$

where $CSN_{averted}$ denotes the number of cause-specific deaths averted with vaccine use. $CSN_{modeled}$ represents VAMCM modeled cause-specific deaths. $SSP_{serotype}$ is the proportion of cause-specific deaths due to the vaccine-specific serotype if there were no vaccine coverage. *C* denotes vaccine coverage. And *Eff* is vaccine effectiveness (or efficacy if effectiveness is not available). Data sources for post-hoc adjustment parameters are presented in Table S1.2.

Table S1.2 Data sources of post-hoc adjustment parameters

**Parameters**	**Hib**		**PCV**		**Rota**
Variable as in WUENIC database	Hib3: 3 doses coverage		PCV3: 3 doses coverage		rota_last: last dose administrated
Diseases directly affected by vaccine use	Pneumonia	Meningitis	Pneumonia	Meningitis	Diarrhea
$SSP_{serotype}$	Watt, 2009[13]	Davis, 2013[14]	O'Brien, 2009[15]	Davis, 2013[14]	Lanata, 2013[16]
% of disease due to serotype in existing vaccine	Not applicable		Johnson, 2010[12]		Not applicable
C	WUENIC[17]		WUENIC[17]		WUENIC[17]
Vaccine formulation	Not applicable		WUENIC,[17] government estimates		Not applicable
Eff	Griffiths, 2012[18]	Davis, 2013[14]	Lucero, 2009[19]*	Davis, 2013[14]	Lamberti, 2015[20]*

WUENIC: WHO/UNICEF Estimates of National Immunization Coverage[17]
*vaccine efficacy

PCV and Hib were considered to have relatively independent effect thus we assumed their effects were additive. Averted number of deaths due to pneumonia, meningitis or diarrhea were re-distributed to the remaining causes pro rata. The order in which the three causes were adjusted was taken into consideration by taking the average of all the permutations of the adjusting order.

Vaccine effectiveness was used when available. If there was no systematic review or meta-analysis published to provide evidence-based vaccine effectiveness estimates, vaccine efficacy was used instead. Rota effectiveness/efficacy on preventing severe rotavirus diarrhea was shown to vary across regions and was not available in all regions.[20] For VAMCM countries/regions lacking Rota efficacy data, we borrowed values from regions with data available based on the following rules (Table S1.3):

- For regions with low gross national income (GNI) per capita (regional mean GNI per capita for 2013-2015 was less than 5,000 current international dollar) and low urban population (regional mean of percent urban population for 2013-2015 was less than 50%), the efficacy value of Sub-Saharan Africa was used
- For regions with moderate GNI per capita (regional mean GNI per capita for 2013-2015 was between 5,000 and 6,000 current international dollar) and low urban population (regional mean of percent urban population for 2013-2015 was less than 50%), the efficacy value of Southern Asia was used

- For regions with high GNI per capita (regional mean GNI per capita for 2013-2015 was greater than or equal to 6,000 current international dollar) and high urban population (regional mean of percent urban population for 2013-2015 was greater than or equal to 50%), the effectiveness value of Latin America & Caribbean was used.

Table S1.3 Rotavirus vaccine effectiveness/efficacy approximation

**Region**	**Effectiveness /efficacy**	**Approximation where needed**
Oceania	Efficacy	Sub-Saharan Africa
Sub-Saharan Africa	Efficacy	
South-eastern Asia	Efficacy	Southern Asia
Southern Asia	Efficacy	
Caucasus and Central Asia	Efficacy	Southern Asia
Eastern Asia	Efficacy	
Latin America & Caribbean	Effectiveness	
Western Asia	Effectiveness	Latin America & Caribbean
Northern Africa	Effectiveness	Latin America & Caribbean

Potential bias in input data

VAMCM models were built based on cause-of-deaths input data from verbal-autopsy studies. We used a systematic review approach to capture both published and major unpublished verbal-autopsy studies reporting causes of deaths information that met our inclusion/exclusion criteria. This could potentially suffer from reporting and/or publication bias, especially when some studies only reported leading causes of deaths. Many covariates used in the models like female literacy, vaccine coverage, low birth weight, and improved water source etc. came from large population surveys (e.g. Demographic and Health Surveys), which could be subject to underlying recall bias and other measurement errors.

Webappendix 2. GATHER checklist

Item	Checklist item*	Section(s) or sources providing information
Objectives and funding		
1	Estimated indicator and population	Methods
2	Funding sources	Funding section of summary
Data Inputs		
For all data inputs from multiple sources that are synthesized as part of the study:		
3	Data identification	Methods and appendix 1; Methods and appendix of previous publications[1,2,21]
4	Inclusion/exclusion criteria	Appendix 1; Methods and appendix of previous publications[1,2,21]
5	Included data sources and their main characteristics	Open access databases**
6	Potential important biases of input data	Discussion and appendix 1
For data inputs that contribute to the analysis but were not synthesized as part of the study:		
7	Source of other data inputs	Open access databases**
For all data inputs:		
8	Accessible input data files	Open access databases**
Data analysis		
9	Conceptual overview of the data analysis method	Methods and appendix of pervious publications. [1,21] List of model covariates inspired by Mosley-Chen framework of child survival. [22]
10	Description of all steps of the analysis	Methods and appendix of current and previous publications[1,2,21]
11	Model selection methods	Methods and appendix of current and previous publications[1,2,21]
12	Model performance and/or sensitivity analysis.	Methods
13	Uncertainty estimation methods	Methods and discussion; Methods and appendix of previous publications. [1,2,21]
14	Statistical code	Open access databases**
Results and discussion		
15	Accessible estimates data files	Open access databases**

16	Uncertainty of the estimates	Results and discussion
17	Results interpretation in light of existing evidence	Results and discussion
18	Limitations of the estimates	Discussion

*Detailed GATHER statement with explanation and elaboration of the items could be found on gather - statement.org

** Open access databases with input files and analytical code of the study could be accessed on the Maternal and Child Epidemiology Estimates project website: http://tinyurl.com/Hopkins-MNCH-cod-openaccess

Webappendix 3. CSMFs by MDG regions in 2015*

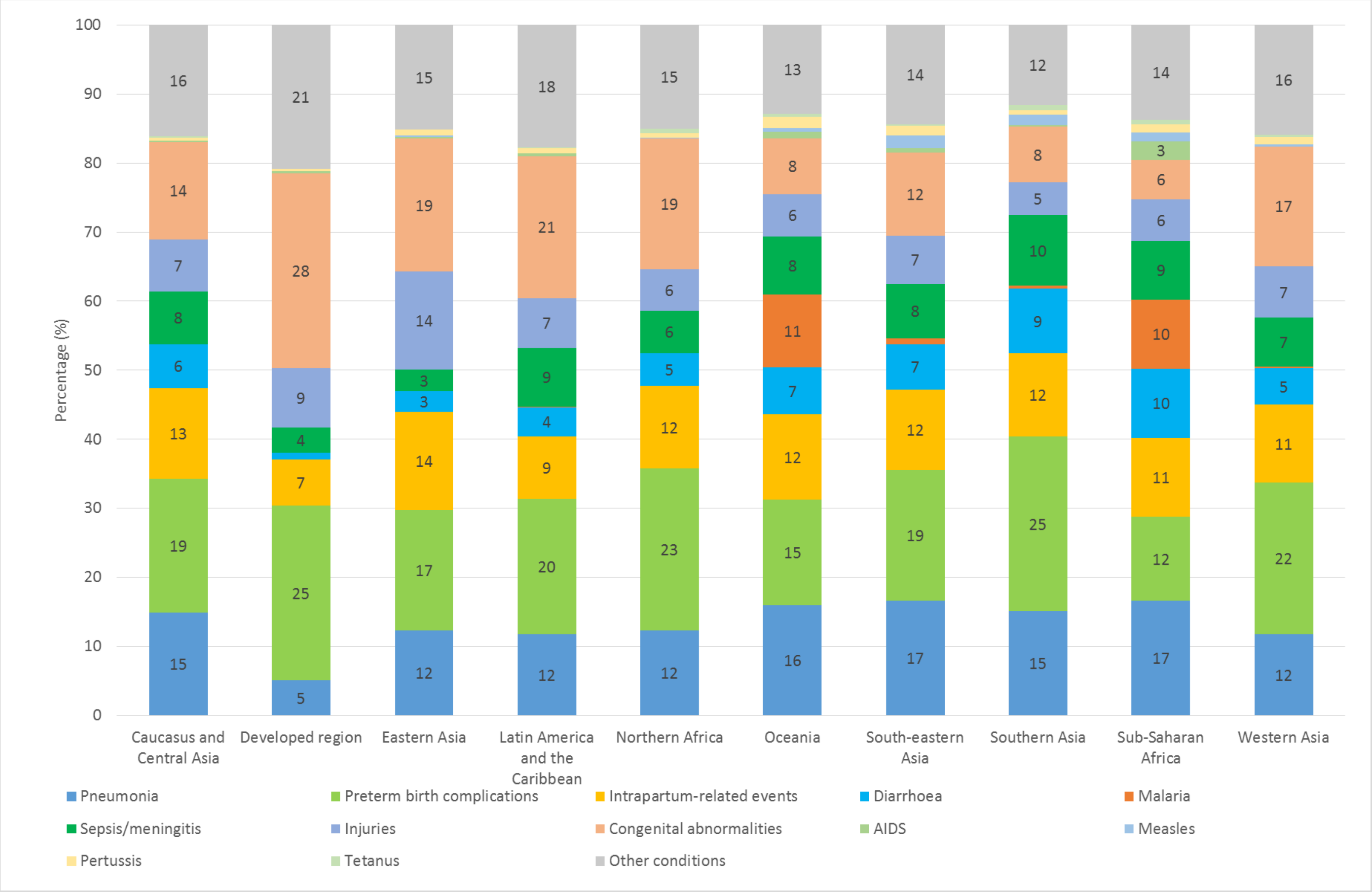

*CSMFs are not labeled if less than 2%

Webappendix 4. CSMFs of the top 10 countries with the highest number of under-five deaths in 2015

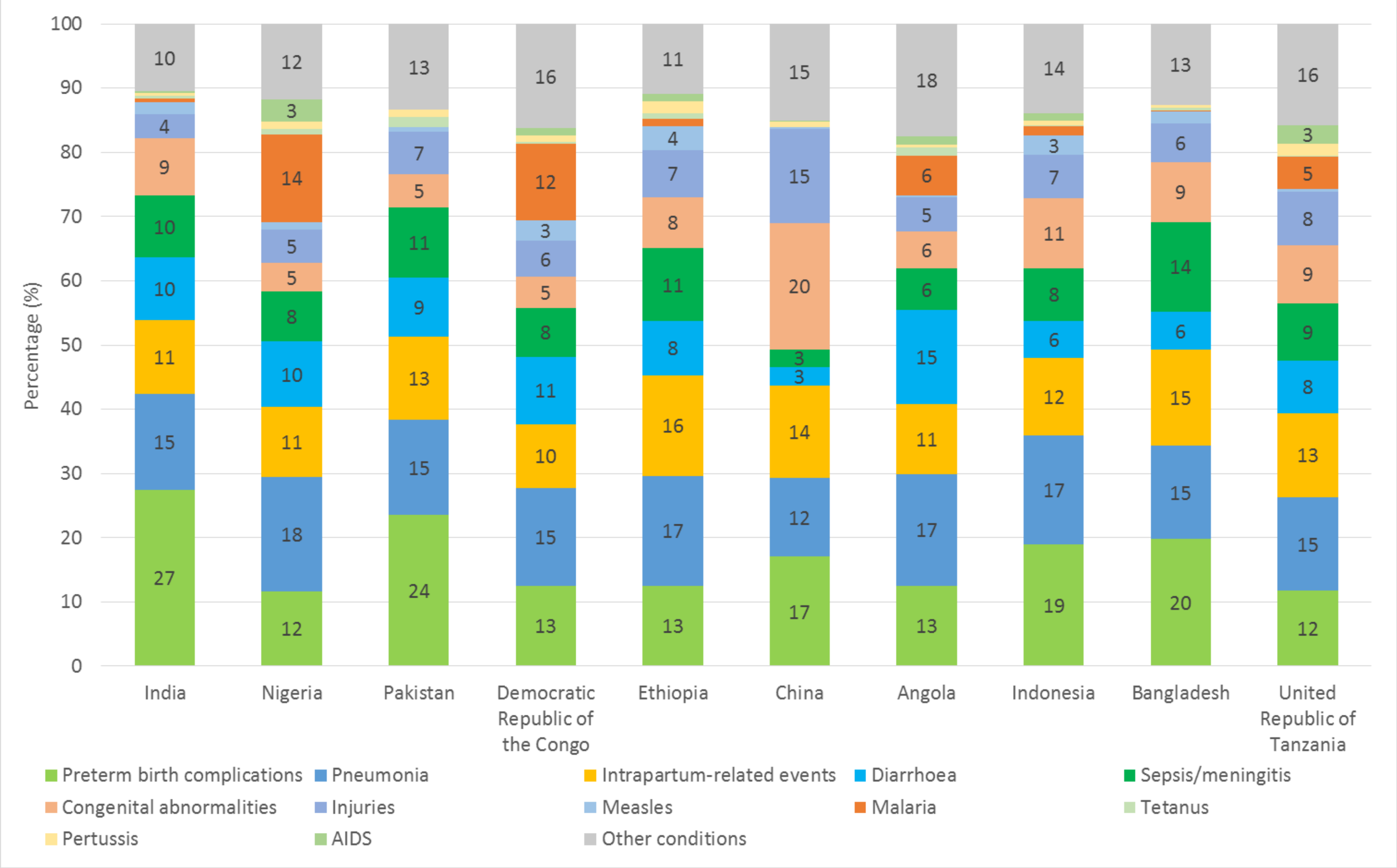

*CSMFs are not labeled if less than 2%

Countries are ordered from the highest number of under-five deaths to the lowest from left to right.

Webappendix 5. CSMFs of the 10 countries with the highest U5MR in 2015

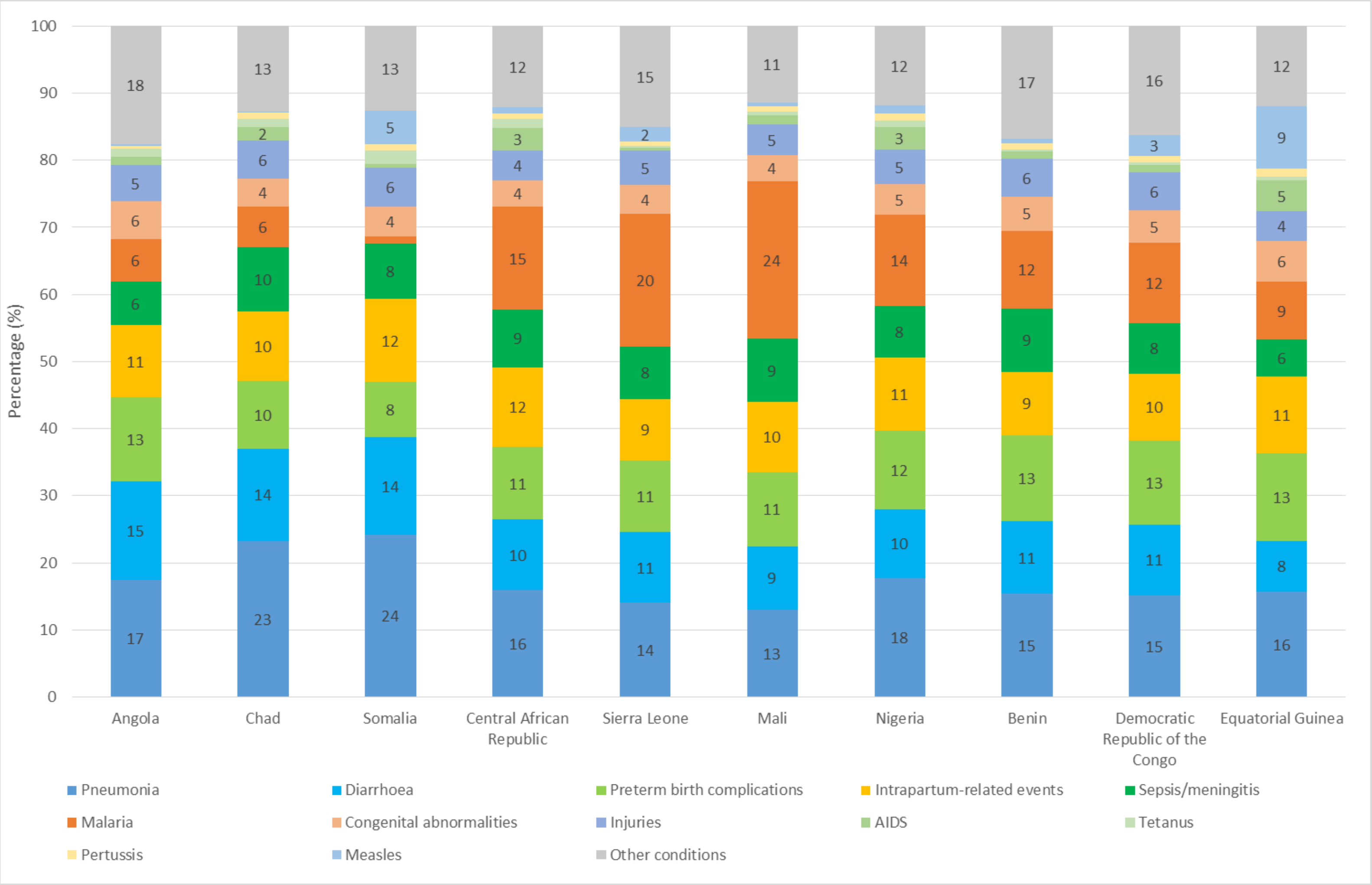

*CSMFs are not labeled if less than 2%

Countries are ordered from the highest U5MR to the lowest from left to right.

		0-28 days										1-59 months												
	Year	U5D[1]	NEO[2]	PNE[3]	PRE[4]	INT[5]	SEP[6]	TET[7]	OTH[8]	CON[9]	DIA[10]	POS[11]	PNE[3]	PRE[4]	INT[5]	MEN[12]	OTH[8]	CON[9]	DIA[10]	MEA[13]	INJ[14]	MAL[15]	AIDS	PER[16]
Global	2000	9961990	3910377	296390	1210449	1048068	532415	161202	304609	316858	40386	6051613	1441712	128090	59043	284347	849579	241696	1172901	484821	387225	725282	220327	56589
	2001	9703848	3823722	284993	1192124	1022584	528765	143047	297488	316245	38476	5880126	1395385	126607	58780	280687	829143	239657	1106008	470785	378528	710707	226726	57113
	2002	9325912	3725881	272931	1169905	991866	524339	126165	289623	314424	36628	5600032	1337931	124853	58330	270475	805759	237200	1043050	364904	369399	700492	229988	57651
	2003	9093774	3624030	261525	1147729	958204	518605	111066	280405	311623	34873	5469744	1284643	121742	57384	268643	779084	231555	974976	421407	357978	684444	230534	57353
	2004	8728101	3518402	249294	1122329	923081	512465	97550	270465	310165	33052	5209700	1242929	120315	57021	252045	758023	229457	918105	315096	383307	648313	227726	57363
	2005	8397848	3422233	236820	1102846	890420	505503	865120	260011	308802	31310	4975615	1196526	118057	56401	247566	734159	225493	860922	295887	345901	615134	222208	57294
	2006	8030261	3328401	224844	1080418	861296	497736	77301	249962	307428	29416	4701860	1164617	117555	56048	213561	718433	224649	814743	189851	343638	586959	214527	57289
	2007	7700945	3243240	213994	1061296	833240	492540	69711	240535	304230	27693	4457705	1118342	116741	55430	203232	704077	223252	769816	120105	339627	546643	202888	57551
	2008	7483245	3163636	205037	1041859	805607	487345	62716	233280	301790	26002	4319610	1076534	115417	54788	209323	689113	219658	725038	127845	356425	501552	186151	57766
	2009	7238082	3090280	197100	1024963	779690	480354	56811	226649	300294	24419	4147802	1012084	115746	55085	175167	688376	218739	696588	151336	335841	471832	169240	57767
	2010	7016748	3020837	190035	1012954	752476	469127	52269	219304	301514	23157	3995911	981982	115202	55538	157361	679828	217871	660876	124870	367776	428425	147880	58301
	2011	6798461	2949328	183802	1000043	725452	454170	48575	212446	302860	21980	3849133	947844	114763	55944	149452	671007	214671	626967	160001	331587	385802	131790	59306
	2012	6535191	2880775	177590	989203	698510	439262	44886	206413	303977	20934	3654416	901349	113647	56410	153998	660926	210984	590996	109600	328708	353850	113986	59962
	2013	6348818	2808809	171506	974318	673008	424863	40970	199924	304300	19919	3540009	843225	114263	58277	135011	667476	209592	568229	120639	331671	335943	97936	57748
	2014	6132134	2745066	166385	959498	651527	413548	37265	193173	304885	18786	3387068	792474	113488	58657	122613	664013	208786	535929	95092	330431	321704	88152	55729
	2015	5941937	2680667	161142	943318	631385	401577	33944	186730	304934	17637	3261270	759944	112062	57776	115093	654125	207077	508745	73838	326464	306371	85784	53990
Caucasus and Central Asia	2000	87742	36620	2304	14756	8630	3888	433	2276	4089	244	51122	15020	1194	1394	2655	11902	3571	10263	20	4725	0	147	229
	2001	82764	35517	2209	14206	8329	3835	383	2223	4112	220	47247	13916	1122	1311	2424	11062	3428	9099	18	4460	0	181	225
	2002	78819	34669	2134	13713	8115	3803	341	2191	4173	198	44150	13020	1065	1247	2224	10386	3336	8152	31	4254	0	210	225
	2003	75775	34195	2085	13333	8013	3796	307	2189	4291	180	41580	12278	1015	1192	2052	9823	3244	7369	45	4088	0	239	235
	2004	73538	33799	2042	12961	7939	3797	276	2198	4422	164	39739	11750	995	1147	1919	9427	3221	6765	24	3982	0	268	242
	2005	71980	33734	2020	12713	7939	3853	253	2224	4579	152	38246	11316	972	1119	1806	9096	3205	6251	38	3899	0	294	249
	2006	70935	33753	2004	12509	7940	3949	232	2249	4727	142	37182	11020	955	1106	1720	8867	3215	5846	25	3855	0	311	261
	2007	70210	33946	1997	12378	7980	4063	216	2287	4889	135	36264	10777	945	1090	1650	8670	3212	5495	18	3821	0	310	277
	2008	69654	34107	1989	12251	8005	4169	201	2324	5039	129	35547	10332	948	1102	1512	8673	3259	5283	0	3864	0	289	284
	2009	69100	34154	1975	12105	7997	4253	187	2351	5164	123	34946	9149	1006	1157	1122	9102	3455	5331	0	4085	0	247	293
	2010	68480	34063	1952	11937	7944	4325	173	2363	5251	117	34417	8820	1014	1169	1034	9131	3477	5144	1	4120	0	209	298
	2011	67736	33981	1931	11794	7891	4383	162	2375	5334	112	33755	8599	1010	1163	986	9038	3444	4909	8	4117	0	177	304
	2012	66950	33832	1906	11646	7825	4418	152	2382	5396	108	33118	8272	1009	1163	920	9002	3453	4712	0	4136	0	147	303
	2013	65793	33404	1868	11414	7697	4402	141	2369	5409	103	32389	8076	997	1149	884	8863	3420	4472	1	4105	0	126	295
	2014	63999	32588	1810	11075	7480	4318	131	2322	5355	97	31411	7779	984	1134	839	8725	3386	4099	1	4070	0	109	285
	2015	61500	31432	1736	10644	7186	4178	121	2246	5232	90	30068	7461	947	1092	801	8378	3277	3798	1	3937	0	104	271
Armenia	2000	1087	561	66	252	73	48	0	28	95	0	526	152	7	8	24	108	89	41	1	93	0	2	3
	2001	1054	548	63	243	70	46	0	29	97	0	506	142	7	9	22	104	89	35	1	91	0	3	3
	2002	1054	556	61	244	70	48	0	31	102	0	498	135	7	9	22	104	91	31	1	91	0	4	3
	2003	1069	570	59	248	71	50	0	33	109	0	499	130	8	9	21	106	95	29	1	93	0	4	4
	2004	1080	578	57	249	71	51	0	35	115	0	502	124	8	10	21	109	98	28	1	94	0	5	5
	2005	1073	578	54	247	71	50	0	37	119	0	495	117	8	10	20	110	100	26	1	94	0	5	5
	2006	1045	563	50	240	69	47	0	38	120	0	482	109	8	10	19	110	101	24	1	92	0	4	5
	2007	1001	532	45	225	64	43	0	37	117	0	469	102	8	10	18	108	101	21	1	91	0	4	5
	2008	944	505	41	213	61	39	0	36	115	0	439	92	9	10	16	102	98	19	0	85	0	4	5
	2009	887	471	37	197	56	36	0	35	110	0	416	85	9	11	15	96	96	16	0	80	0	4	5
	2010	831	441	33	184	52	33	0	33	105	0	390	73	11	13	16	95	92	13	0	70	0	3	5
	2011	779	413	30	171	49	31	0	32	101	0	366	64	13	16	15	87	90	11	0	63	0	2	5
	2012	723	382	26	158	45	28	0	30	95	0	341	58	13	16	13	82	86	10	0	58	0	2	4
	2013	671	355	23	146	42	25	0	29	90	0	316	52	12	15	12	76	81	9	0	53	0	2	4
	2014	620	324	20	133	38	23	0	27	84	0	296	48	12	15	11	71	78	8	0	49	0	1	3
	2015	577	301	18	124	35	20	0	25	79	0	276	44	12	14	10	67	74	7	0	45	0	1	3
Azerbaijan	2000	9630	4176	246	1825	985	389	81	245	367	38	5454	1639	123	151	239	1265	330	1184	9	488	0	3	24
	2001	8618	3905	227	1706	907	370	66	229	368	32	4713	1422	108	132	202	1105	294	976	11	435	0	4	23
	2002	7883	3712	214	1611	853	358	54	219	375	28	4171	1260	97	118	175	984	267	822	24	396	0	5	23
	2003	7379	3604	206	1547	823	354	46	214	390	25	3775	1140	88	108	153	894	247	708	38	368	0	7	24
	2004	7006	3531	201	1495	802	353	39	211	408	22	3475	1059	83	101	137	834	235	627	18	350	0	7	24
	2005	6737	3494	197	1457	790	358	34	211	427	20	3243	990	78	95	123	780	224	560	23	335	0	9	26
	2006	6519	3445	193	1416	774	363	29	210	442	18	3074	945	75	92	112	746	221	511	8	326	0	11	27
	2007	6329	3418	189	1386	761	372	26	210	458	16	2911	900	73	89	102	709	218	466	0	316	0	10	29
	2008	6211	3410	187	1366	751	385	23	210	473	15	2801	868	71	86	95	686	216	431	0	310	0	9	29
	2009	6200	3465	189	1372	755	403	21	214	497	14	2735	849	70	85	91	671	215	405	0	308	0	11	30
	2010	6327	3597	194	1408	777	430	20	223	531	14	2730	850	70	85	90	672	217	390	0	313	0	12	31
	2011	6611	3829	205	1484	822	466	19	239	579	14	2782	813	75	91	79	716	232	398	0	336	0	11	32
	2012	6982	4123	220	1582	880	508	19	260	639	15	2859	750	82	99	62	780	256	416	0	369	0	12	33
	2013	7296	4350	231	1654	924	541	19	276	689	15	2946	767	85	103	62	810	270	415	1	387	0	11	34
	2014	7403	4401	232	1663	931	551	17	281	711	15	3002	675	93	112	46	879	296	433	1	422	0	10	35
	2015	7206	4267	223	1606	897	536	16	274	702	14	2939	662	92	111	45	863	295	409	1	418	0	10	34
Georgia	2000	1863	1074	126	503	144	94	0	49	157	0	789	203	6	7	36	185	121	69	1	154	0	2	5
	2001	1709	1014	113	469	134	90	0	49	159	0	695	174	6	7	31	164	112	57	1	138	0	2	5
	2002	1603	965	102	441	126	87	0	48	162	0	638	153	6	7	27	151	106	51	2	127	0	3	5
	2003	1529	930	92	419	119	85	0	48	166	0	599	137	6	7	24	145	103	46	1	120	0	4	6
	2004	1460	897	84	397	113	84	0	48	171	0	563	124	6	7	22	138	102	40	1	114	0	4	6
	2005	1391	856	75	373	106	83	0	46	172	0	535	115	6	8	21	131	103	32	1	110	0	4	6
	2006	1323	814	67	349	100	82	0	45	172	0	509	106	7	8	19	125	103	26	0	105	0	4	6
	2007	1248	767	58	325	92	79	0	43	170	0	481	96	7	9	17	118	103	21	0	99	0	4	6
	2008	1173	720	51	302	86	74	0	42	166	0	453	86	7	9	16	113	100	19	0	92	0	5	6
	2009	1101	678	45	282	80	70	0	40	161	0	423	78	8	9	14	107	97	17	0	84	0	4	6
	2010	1026	629	39	260	74	64	0	38	154	0	397	65	10	12	16	106	95	14	0	72	0	2	6
	2011	955	581	33	239	68	58	0	36	146	0	374	58	12	14	14	98	93	12	0	65	0	2	6
	2012	877	536	29	220	63	52	0	34	138	0	341	51	12	14	12	88	88	10	0	58	0	2	6
	2013	801	494	25	203	58	46	0	32	130	0	307	44	12	14	11	79	81	8	0	51	0	2	5
	2014	734	445	21	183	52	41	0	29	119	0	289	40	12	14	10	74	78	7	0	48	0	1	5
	2015	673	408	19	168	48	36	0	28	110	0	265	36	11	14	8	68	73	6	0	43	0	1	5
Kazakhstan	2000	9335	4235	228	1818	897	412	26	239	601	15	5100	1465	133	163	174	1187	487	920	0	522	0	14	35
	2001	9064	4255	229	1789	906	422	24	243	628	14	4809	1382	128	156	160	1116	480	832	0	501	0	18	37
	2002	9054	4347	234	1781	938	433	23	254	670	14	4707	1356	127	155	153	1093	486	783	0	499	0	17	39
	2003	9155	4514	244	1794	993	444	22	272	731	13	4641	1339	127	155	147	1076	496	742	0	501	0	16	42
	2004	9285	4688	254	1800	1053	455	22	295	796	13	4597	1326	127	156	143	1058	509	705	0	504	0	21	49
	2005	9324	4746	257	1767	1072	473	20	304	839	12	4578	1323	128	156	140	1057	517	673	0	511	0	25	48
	2006	9255	4711	254	1708	1057	500	18	301	862	11	4544	1311	129	157	137	1043	532	639	0	516	0	28	52
	2007	9069	4619	248	1633	1028	519	16	294	872	10	4450	1285	127	155	133	1016	532	600	0	515	0	30	58
	2008	8785	4470	239	1540	991	518	13	286	875	9	4315	1141	129	158	105	1037	543	585	0	535	0	23	59
	2009	8393	4265	226	1433	944	499	12	276	867	8	4128	986	131	160	76	1035	554	560	0	546	0	19	62
	2010	7944	4008	210	1318	884	473	10	262	844	7	3936	946	125	153	73	987	530	512	0	533	0	16	62
	2011	7439	3773	196	1219	827	449	9	248	820	6	3666	888	116	142	68	921	487	458	0	509	0	15	62
	2012	6970	3504	180	1115	763	419	8	232	781	6	3466	768	114	139	52	903	477	430	0	508	0	16	60
	2013	6489	3230	164	1015	698	389	7	215	736	5	3259	695	109	133	44	864	456	394	0	493	0	15	56
	2014	5994	2989	150	931	641	361	6	200	695	5	3005	638	101	123	40	803	423	351	0	465	0	8	52
	2015	5528	2744	137	851	586	333	6	184	643	4	2784	593	94	115	37	746	393	314	0	437	0	7	48
Kyrgyzstan	2000	5380	2375	138	926	556	247	17	146	336	9	3005	855	77	94	164	691	276	530	0	295	0	3	19
	2001	5021	2268	131	878	527	240	15	139	329	8	2753	785	71	87	149	634	261	468	0	275	0	4	18
	2002	4685	2164	124	828	501	234	14	134	322	7	2521	722	66	81	136	582	243	413	0	256	0	4	18
	2003	4389	2082	118	785	482	229	12	130	319	7	2307	661	61	75	123	533	230	364	0	237	0	5	17
	2004	4189	2048	116	758	475	230	11	130	322	6	2141	614	68	59	113	495	220	325	0	223	0	7	16
	2005	4113	2075	117	752	483	238	11	133	334	6	2038	585	67	55	107	469	213	299	0	215	0	9	18
	2006	4132	2137	121	758	499	251	11	139	352	6	1995	574	64	57	104	460	213	282	0	213	0	11	18
	2007	4191	2214	125	768	518	266	10	147	375	6	1977	569	66	54	102	455	211	270	0	214	0	17	20
	2008	4245	2301	129	779	538	282	10	154	402	6	1944	560	60	58	99	446	211	255	0	213	0	21	21
	2009	4269	2322	130	767	543	289	9	158	420	6	1947	563	66	53	99	447	212	246	0	218	0	20	23

U5D[1]: total number of under 5 death; NEO[2]: total number of neonatal death; PNE[3]: pneumonia; PRE[4]: preterm; INT[5]: intrapartum related events; SEP[6]: sepsis/meningitis; TET[7]: tetanus; OTH[8]: other disorders; CON[9]: congenital; DIA[10]: diarrea; POS[11]: total number of post neonatal death; MEN[12]: meningitis; MEA[13]: measles; INJ[14]: injury; MAL[15]: malaria; PER[16]: pertussis

			0-28 days										1-59 months											
	Year	U5D[1]	NEO[2]	PNE[3]	PRE[4]	INT[5]	SEP[6]	TET[7]	OTH[8]	CON[9]	DIA[10]	POS[11]	PNE[3]	PRE[4]	INT[5]	MEN[12]	OTH[8]	CON[9]	DIA[10]	MEA[13]	INJ[14]	MAL[15]	AIDS	PER[16]
	2010	4229	2306	129	743	539	291	8	159	433	5	1923	464	70	61	62	490	232	257	0	243	0	20	24
	2011	4174	2257	125	710	526	288	7	157	439	5	1917	463	70	61	61	488	232	246	0	247	0	23	26
	2012	4097	2217	122	682	514	286	6	155	447	4	1880	456	69	60	60	478	229	232	0	247	0	23	27
	2013	4010	2169	119	656	501	282	6	153	449	4	1841	446	67	59	58	470	226	218	0	247	0	25	26
	2014	3871	2099	114	626	482	274	5	149	444	4	1772	430	65	57	56	452	218	202	0	240	0	26	25
	2015	3644	1985	107	587	453	260	5	141	428	4	1659	404	61	53	52	424	206	182	0	228	0	24	24
Tajikistan	2000	17821	5756	385	2178	1505	710	96	396	416	69	12065	3647	280	277	800	2708	576	2695	5	989	0	52	35
	2001	16323	5482	365	2052	1433	684	83	379	427	60	10841	3301	255	252	712	2464	528	2312	4	922	0	56	34
	2002	14801	5199	343	1917	1360	657	71	362	437	51	9602	2943	229	226	623	2207	480	1954	3	848	0	57	32
	2003	13412	4911	322	1780	1286	628	61	344	447	42	8501	2622	205	202	543	1975	431	1651	5	778	0	58	32
	2004	12341	4659	303	1657	1223	603	52	329	456	36	7682	2382	187	184	482	1803	397	1426	3	726	0	58	31
	2005	11663	4562	295	1590	1202	593	46	327	477	32	7101	2215	175	172	439	1682	373	1264	0	691	0	57	32
	2006	11291	4589	296	1568	1214	596	43	333	510	29	6702	2102	167	163	409	1602	356	1146	0	670	0	55	33
	2007	11235	4726	303	1585	1255	612	41	350	552	28	6509	2052	163	159	393	1567	347	1073	0	665	0	54	36
	2008	11367	4909	314	1620	1301	644	39	368	595	28	6458	1887	171	168	323	1654	367	1084	0	710	0	53	39
	2009	11604	5075	323	1653	1337	681	37	384	632	27	6529	1748	184	180	254	1786	392	1121	0	771	0	53	40
	2010	11822	5237	330	1688	1365	731	35	396	665	27	6585	1772	186	182	255	1815	396	1096	0	790	0	50	43
	2011	11985	5366	336	1712	1385	768	33	407	697	26	6619	1777	188	185	250	1846	401	1070	0	810	0	47	44
	2012	12110	5471	340	1730	1401	799	32	417	727	25	6639	1797	189	186	253	1859	403	1037	0	822	0	49	46
	2013	12154	5558	343	1744	1415	819	30	426	755	25	6596	1785	189	186	247	1867	402	1000	0	830	0	46	46
	2014	12055	5552	341	1733	1405	822	28	427	771	24	6503	1762	187	184	241	1856	398	956	0	831	0	43	45
	2015	11799	5494	335	1710	1381	814	27	423	781	23	6305	1714	182	179	234	1811	387	897	0	816	0	41	44
Turkmenistan	2000	7679	2736	168	1094	677	302	35	177	262	21	4943	1455	114	139	214	1191	325	1073	0	417	0	0	15
	2001	7582	2809	172	1114	695	316	34	184	275	20	4773	1411	112	137	205	1156	325	1003	0	409	0	0	15
	2002	7796	2983	182	1170	739	342	34	197	299	20	4813	1428	115	140	206	1170	342	977	0	419	0	0	16
	2003	8141	3186	193	1237	790	373	34	212	327	20	4955	1476	120	147	211	1208	363	973	0	438	0	0	19
	2004	8370	3338	202	1281	828	398	33	225	352	20	5032	1504	124	151	212	1231	384	954	0	452	0	0	20
	2005	8359	3395	204	1289	842	413	31	231	366	19	4964	1488	124	152	205	1219	395	909	0	452	0	0	20
	2006	8102	3302	197	1242	818	409	28	227	364	18	4800	1444	122	149	194	1182	395	850	0	444	0	0	20
	2007	7631	3108	184	1159	768	391	25	215	350	16	4523	1366	116	142	179	1118	382	775	0	424	0	0	20
	2008	7042	2875	169	1063	709	368	21	201	331	14	4167	1262	109	133	161	1033	364	689	0	397	0	0	19
	2009	6519	2689	157	985	661	349	19	190	316	12	3830	1165	101	123	145	955	340	613	0	370	0	0	17
	2010	6178	2553	148	928	626	335	17	182	306	11	3625	992	102	125	106	964	347	596	0	377	0	0	16
	2011	5984	2513	145	905	614	334	15	181	307	10	3471	928	100	122	93	938	342	559	0	372	0	0	17
	2012	5943	2532	146	904	618	340	15	185	316	10	3411	865	102	124	77	949	352	546	0	380	0	0	17
	2013	5954	2567	147	909	624	347	14	189	327	10	3387	861	102	125	75	945	357	524	0	382	0	0	17
	2014	5960	2600	148	915	630	354	13	192	338	9	3360	856	102	125	73	940	361	502	0	384	0	0	17
	2015	5868	2581	146	904	623	352	13	191	343	9	3287	839	101	123	71	922	360	475	0	380	0	0	16
Uzbekistan	2000	34947	15707	946	6161	3793	1686	178	996	1855	92	19240	5604	454	555	1004	4567	1367	3751	5	1769	0	71	93
	2001	33393	15236	910	5956	3657	1666	161	971	1830	85	18157	5298	435	531	942	4319	1340	3416	2	1689	0	94	90
	2002	31943	14743	874	5720	3528	1646	145	947	1805	78	17200	5023	418	511	884	4095	1319	3121	2	1618	0	120	89
	2003	30701	14398	850	5524	3448	1634	132	935	1803	72	16303	4772	400	489	829	3887	1279	2856	2	1554	0	145	91
	2004	29807	14060	826	5324	3373	1623	119	925	1803	67	15747	4615	392	479	789	3759	1276	2660	2	1519	0	166	91
	2005	29320	14028	821	5237	3372	1646	110	935	1844	63	15292	4483	385	471	751	3648	1281	2488	14	1492	0	185	94
	2006	29268	14192	827	5228	3410	1701	103	957	1905	60	15076	4430	384	469	727	3600	1294	2368	16	1490	0	198	100
	2007	29506	14562	844	5299	3493	1781	99	992	1996	59	14944	4407	385	471	706	3579	1318	2269	17	1498	0	191	103
	2008	29887	14917	858	5370	3569	1859	94	1026	2083	57	14970	4437	391	478	696	3602	1361	2201	0	1523	0	174	106
	2009	30127	15189	869	5416	3620	1926	89	1054	2161	55	14938	3675	438	536	428	4006	1549	2353	0	1707	0	136	110
	2010	30123	15292	869	5408	3628	1968	84	1069	2213	53	14831	3659	440	538	417	4002	1567	2266	1	1722	0	107	111
	2011	29809	15249	860	5353	3601	1990	78	1074	2244	50	14560	3606	436	533	406	3944	1567	2154	8	1715	0	78	112
	2012	29248	15067	844	5254	3542	1986	72	1069	2253	47	14181	3529	430	526	392	3861	1563	2033	0	1695	0	44	110
	2013	28418	14681	817	5087	3435	1952	66	1048	2232	44	13737	3427	421	514	376	3752	1548	1904	0	1662	0	27	107
	2014	27362	14178	784	4890	3300	1893	60	1016	2194	40	13184	3329	412	503	363	3649	1533	1640	0	1632	0	21	102
	2015	26205	13652	750	4694	3161	1827	55	980	2147	37	12553	3171	395	483	344	3477	1489	1508	0	1569	0	20	97
Developed region	2000	129276	70889	2959	29393	8246	4473	3	6910	18903	0	58387	8715	2755	845	2161	15692	14629	1935	34	10690	2	691	238
	2001	125705	69391	2762	28900	7947	4236	4	7085	18456	0	56314	8143	2735	850	1955	15145	14238	1758	25	10443	0	783	240
	2002	121724	67875	2553	28015	7796	4099	4	7060	18348	0	53849	7398	2711	899	1777	14917	13568	1591	24	9866	0	858	241
	2003	117598	66164	2294	27758	7189	3971	3	7248	17701	0	51434	6899	2498	841	1690	14326	13148	1476	22	9370	0	947	217
	2004	114105	63730	1893	27074	6731	3883	3	7051	17096	0	50375	6312	2700	783	1491	14258	13010	1383	16	9297	2	875	248
	2005	110665	62190	1803	26656	6523	3743	3	6803	16660	0	48475	5832	2642	752	1433	13889	12717	1291	24	8841	1	786	267
	2006	107786	60271	1619	25753	6220	3426	2	6833	16417	0	47515	5329	2564	795	1319	14043	12464	1243	25	8700	2	807	224
	2007	104994	58607	1600	24872	6085	3308	2	6795	15945	0	46387	5011	2520	739	1223	13879	12376	1166	25	8406	0	807	236
	2008	102389	57373	1440	24349	5865	3174	2	6806	15737	0	45016	4787	2576	752	1108	13581	11999	1154	21	8062	0	702	274
	2009	99235	55860	1327	23662	5814	3032	2	6665	15357	0	43375	4669	2369	689	1076	13087	11743	1159	19	7699	2	606	257
	2010	96292	53813	1217	22677	5682	2755	2	6355	15125	0	42479	4258	2189	696	1078	12886	11795	1148	39	7597	3	502	286
	2011	93113	51778	1086	21648	5507	2665	2	6267	14602	0	41335	4107	2132	684	904	12349	11347	1056	28	7907	2	542	278
	2012	89415	49631	986	20988	5260	2481	3	5893	14021	0	39784	3657	2085	697	830	12458	11145	960	33	7069	2	561	287
	2013	85951	47410	883	19919	5015	2355	3	5992	13243	0	38541	3491	1890	629	841	13164	10104	803	33	6836	1	501	250
	2014	82396	45379	825	19000	4824	2271	3	5598	12858	0	37017	3402	1939	658	797	11913	10272	827	30	6647	2	279	250
	2015	79550	43611	769	18261	4623	2178	3	5413	12364	0	35939	3263	1891	643	765	11565	10028	795	27	6448	2	269	241
Albania	2000	1380	585	56	258	76	49	3	30	113	0	795	210	17	14	33	189	171	40	2	112	0	0	6
	2001	1252	544	52	238	70	44	4	29	109	0	708	181	16	13	29	171	157	34	0	102	0	0	6
	2002	1128	503	47	218	64	37	4	28	104	0	625	155	14	12	25	153	142	28	0	91	0	0	5
	2003	1019	458	43	197	58	31	3	28	98	0	561	134	13	11	22	139	131	23	0	84	0	0	5
	2004	918	412	38	175	52	27	3	26	90	0	506	117	11	10	19	127	121	20	0	77	0	0	4
	2005	823	372	34	156	47	24	3	25	83	0	451	101	10	9	17	115	110	16	0	70	0	0	3
	2006	737	331	29	138	42	21	2	22	76	0	406	88	9	8	15	103	101	14	0	64	0	1	3
	2007	662	297	26	123	38	19	2	20	69	0	365	77	9	8	13	93	93	12	0	58	0	1	3
	2008	598	271	23	112	35	17	2	18	64	0	327	67	8	7	11	81	84	10	1	52	0	1	4
	2009	559	254	20	104	32	17	2	17	61	0	305	56	11	10	12	77	81	8	1	45	0	1	4
	2010	540	244	19	100	31	16	2	16	60	0	296	53	11	9	12	74	80	7	1	44	0	1	4
	2011	543	246	18	101	31	16	2	16	61	0	297	52	11	9	11	73	81	7	2	44	0	1	4
	2012	567	260	18	107	33	17	3	18	65	0	307	53	12	10	12	77	85	7	1	46	0	2	4
	2013	593	275	19	113	34	17	3	19	70	0	318	54	13	10	12	79	89	7	1	47	0	1	4
	2014	613	282	18	116	35	17	3	20	73	0	331	55	13	11	12	82	94	7	1	49	0	2	4
	2015	618	279	18	115	35	17	3	20	73	0	339	56	14	11	12	84	97	7	1	50	0	2	4
Andorra	2000	3	2	0	1	0	0	0	0	1	0	1	0	0	0	0	0	0	0	0	0	0	0	0
	2001	3	1	0	0	0	0	0	0	0	0	2	0	0	0	0	1	1	0	0	0	0	0	0
	2002	3	1	0	0	0	0	0	0	0	0	2	0	0	0	0	1	1	0	0	0	0	0	0
	2003	3	1	0	0	0	0	0	0	0	0	2	0	0	0	0	1	1	0	0	0	0	0	0
	2004	3	1	0	0	0	0	0	0	0	0	2	0	0	0	0	1	1	0	0	0	0	0	0
	2005	3	1	0	0	0	0	0	0	0	0	2	0	0	0	0	1	1	0	0	0	0	0	0
	2006	2	1	0	0	0	0	0	0	0	0	1	0	0	0	0	0	0	0	0	0	0	0	0
	2007	2	1	0	0	0	0	0	0	0	0	1	0	0	0	0	0	0	0	0	0	0	0	0
	2008	2	1	0	0	0	0	0	0	0	0	1	0	0	0	0	0	0	0	0	0	0	0	0
	2009	2	1	0	0	0	0	0	0	0	0	1	0	0	0	0	0	0	0	0	0	0	0	0
	2010	2	1	0	0	0	0	0	0	0	0	1	0	0	0	0	0	0	0	0	0	0	0	0
	2011	2	1	0	0	0	0	0	0	0	0	1	0	0	0	0	0	0	0	0	0	0	0	0
	2012	2	1	0	0	0	0	0	0	0	0	1	0	0	0	0	0	0	0	0	0	0	0	0
	2013	2	1	0	0	0	0	0	0	0	0	1	0	0	0	0	0	0	0	0	0	0	0	0
	2014	2	1	0	0	0	0	0	0	0	0	1	0	0	0	0	0	0	0	0	0	0	0	0
	2015	2	1	0	0	0	0	0	0	0	0	1	0	0	0	0	0	0	0	0	0	0	0	0
Australia	2000	1532	853	5	200	138	39	0	208	263	0	679	35	67	12	25	207	167	2	0	162	0	0	2
	2001	1494	847	3	193	128	28	0	231	264	0	647	48	62	7	37	206	136	7	0	139	0	3	1
	2002	1464	822	3	189	145	37	0	228	219	0	642	36	42	23	12	244	154	3	0	125	0	0	3
	2003	1472	828	0	170	145	17	0	253	243	0	644	51	23	9	24	257	137	1	0	140	0	0	0
	2004	1470	818	1	255	122	34	0	147	259	0	652	64	33	11	26	268	122	3	0	123	0	0	1

U5D[1]: total number of under 5 death; NEO[2]: total number of neonatal death; PNE[3]: pneumonia; PRE[4]: preterm; INT[5]: intrapartum related events; SEP[6]: sepsis/meningitis; TET[7]: tetanus; OTH[8]: other disorders; CON[9]: congenital; DIA[10]: diarrea; POS[11]: total number of post neonatal death; MEN[12]: meningitis; MEA[13]: measles; INJ[14]: injury; MAL[15]: malaria; PER[16]: pertussis

Webappendix 6. Estimated numbers of deaths by cause in neonates, children aged 1-59 months at the global, regional and country levels in 2000-2015

			0-28 days									1-59 months												
	Year	U5D[1]	NEO[2]	PNE[3]	PRE[4]	INT[5]	SEP[6]	TET[7]	OTH[8]	CON[9]	DIA[10]	POS[11]	PNE[3]	PRE[4]	INT[5]	MEN[12]	OTH[8]	CON[9]	DIA[10]	MEA[13]	INJ[14]	MAL[15]	AIDS	PER[16]
	2005	1458	843	2	291	122	29	0	149	250	0	615	40	37	11	18	239	141	4	0	125	0	0	1
	2006	1482	850	3	322	119	23	0	148	235	0	632	43	21	14	18	278	112	10	0	133	0	0	1
	2007	1494	865	4	262	142	27	0	149	281	0	629	46	26	8	11	267	142	1	0	129	0	0	0
	2008	1508	879	3	244	135	26	0	183	288	0	629	32	22	17	13	282	142	0	0	118	0	1	1
	2009	1504	883	1	246	152	13	0	205	265	0	621	41	31	7	7	248	159	9	0	119	0	0	0
	2010	1478	871	0	259	136	23	0	178	276	0	607	42	34	11	16	243	150	2	0	105	3	0	2
	2011	1396	811	3	250	148	21	0	157	232	0	585	30	56	10	10	235	143	2	0	97	0	0	2
	2012	1323	770	1	226	131	17	0	162	232	0	553	34	37	9	10	221	138	4	0	98	1	0	1
	2013	1243	725	1	213	123	16	0	153	219	0	518	32	34	8	10	207	129	4	0	92	1	0	1
	2014	1171	685	1	201	116	15	0	144	207	0	486	30	32	8	9	195	121	3	0	86	1	0	1
	2015	1146	657	1	193	112	15	0	138	198	0	489	30	33	8	9	196	122	3	0	86	1	0	1
Austria	2000	412	225	3	86	22	5	0	25	84	0	187	5	5	5	6	56	73	1	0	36	0	0	0
	2001	397	222	1	91	21	1	0	34	73	0	175	9	13	8	7	51	63	0	0	25	0	0	0
	2002	395	220	0	146	7	2	0	6	58	0	175	11	29	0	4	48	56	0	0	27	0	0	0
	2003	400	229	0	150	13	1	0	13	51	0	171	4	25	1	7	32	62	3	0	37	0	0	0
	2004	396	229	1	143	14	2	0	10	60	0	167	11	30	7	5	42	54	0	0	17	0	0	0
	2005	395	226	0	140	15	0	0	6	65	0	169	1	27	4	6	48	55	1	0	26	0	0	0
	2006	389	227	0	155	5	1	0	16	50	0	162	4	27	3	5	49	45	1	0	28	0	0	0
	2007	377	217	0	115	18	3	0	27	53	0	160	4	15	3	1	59	51	3	0	23	0	0	0
	2008	364	205	0	78	27	3	0	31	67	0	159	4	13	5	8	50	52	1	0	26	0	0	0
	2009	351	202	0	70	14	12	0	41	65	0	149	6	11	6	9	48	51	0	0	19	0	0	0
	2010	341	193	1	82	16	5	0	37	53	0	148	5	18	4	8	41	56	1	0	15	0	0	0
	2011	327	187	0	60	26	6	0	24	70	0	140	9	7	6	4	52	48	2	0	10	0	1	1
	2012	315	189	0	58	19	11	0	35	66	0	126	7	13	1	7	40	39	3	0	16	0	0	0
	2013	312	184	0	66	12	0	0	35	70	0	128	2	9	1	8	55	39	1	0	11	0	1	0
	2014	300	179	0	62	22	4	0	31	61	0	121	5	8	3	2	54	37	0	0	12	0	0	0
	2015	288	173	0	58	17	5	0	31	62	0	115	4	9	2	5	46	35	1	0	12	0	0	0
Belarus	2000	1322	681	51	280	80	38	0	52	181	0	641	131	29	18	25	141	175	21	0	91	0	3	8
	2001	1208	615	42	253	72	33	0	48	167	0	593	115	28	17	22	131	168	18	0	83	0	2	9
	2002	1113	557	35	229	65	29	0	44	154	0	556	102	28	17	19	123	163	16	0	77	0	2	9
	2003	1028	508	28	209	60	26	0	42	143	0	520	90	27	16	17	115	157	13	0	72	0	2	10
	2004	972	457	24	188	53	24	0	37	131	0	515	85	28	16	16	117	159	12	0	71	0	2	9
	2005	912	417	20	172	49	22	0	34	121	0	495	76	27	16	15	113	157	11	0	69	0	3	9
	2006	860	373	16	153	43	20	0	31	110	0	487	70	27	16	14	113	157	10	0	69	0	2	9
	2007	795	328	13	134	38	17	0	28	98	0	467	61	27	15	13	109	153	8	0	67	0	4	9
	2008	738	293	10	119	34	16	0	26	89	0	445	53	26	15	13	106	148	7	0	64	0	2	10
	2009	693	268	8	108	31	14	0	24	83	0	425	47	27	16	12	101	144	6	0	60	0	1	10
	2010	659	254	7	101	29	14	0	23	80	0	405	44	25	14	9	95	142	6	0	59	0	1	10
	2011	627	252	6	100	28	14	0	23	81	0	375	37	26	15	10	88	132	5	0	52	0	1	10
	2012	617	251	7	99	28	14	0	22	81	0	366	34	26	15	9	86	131	4	0	50	0	1	9
	2013	604	248	7	97	27	14	0	22	81	0	356	32	26	15	8	84	129	4	0	48	0	1	9
	2014	579	240	6	93	26	13	0	22	79	0	339	30	26	15	8	79	125	4	0	44	0	1	8
	2015	567	239	6	92	26	14	0	22	79	0	328	28	25	14	7	77	121	3	0	43	0	1	8
Belgium	2000	637	325	2	86	51	14	0	55	118	0	312	6	23	6	26	108	78	4	0	59	0	0	1
	2001	615	316	4	95	43	17	0	51	105	0	299	5	12	5	32	94	70	3	0	77	0	0	0
	2002	603	311	1	85	39	12	0	63	112	0	292	18	15	5	22	111	65	1	0	54	0	0	0
	2003	595	321	2	92	44	15	0	54	114	0	274	10	14	6	22	105	59	6	0	52	0	0	0
	2004	600	319	3	89	38	16	0	55	117	0	281	6	13	7	28	89	77	9	0	51	0	0	2
	2005	604	317	0	95	47	24	0	58	93	0	287	18	19	2	22	110	57	6	0	53	0	0	0
	2006	606	325	1	90	46	19	0	74	95	0	281	7	11	0	25	113	72	4	0	48	0	0	0
	2007	607	319	2	87	45	17	0	68	100	0	288	17	20	3	22	99	71	2	0	55	0	0	0
	2008	605	312	0	85	43	16	0	73	95	0	293	9	26	3	16	102	79	3	0	55	0	0	0
	2009	599	316	0	99	48	15	0	58	96	0	283	14	17	0	15	105	69	2	0	60	0	0	1
	2010	590	304	1	80	51	13	0	65	94	0	286	14	10	1	7	127	75	0	0	50	0	0	1
	2011	578	303	1	83	61	10	0	58	90	0	275	10	15	2	9	112	65	2	0	58	0	0	3
	2012	561	300	2	73	39	11	0	59	116	0	261	1	17	2	13	100	67	0	0	59	0	0	1
	2013	556	283	1	74	47	11	0	57	94	0	273	8	14	2	10	112	69	1	0	56	0	0	2
	2014	537	279	1	73	46	11	0	56	92	0	258	8	13	2	9	106	65	0	0	53	0	0	2
	2015	522	278	1	72	46	11	0	56	92	0	244	7	13	2	9	100	62	0	0	50	0	0	2
Bosnia and Herzegovina	2000	438	321	17	128	40	20	0	27	89	0	117	17	5	3	3	27	39	4	4	12	0	0	3
	2001	380	272	14	108	34	17	0	23	76	0	108	15	5	3	3	25	37	3	3	11	0	0	3
	2002	321	219	11	87	27	14	0	18	62	0	102	14	5	3	3	24	35	3	2	10	0	0	3
	2003	266	170	8	67	21	11	0	14	48	0	96	13	5	3	2	24	33	3	1	10	0	0	3
	2004	225	135	7	54	17	8	0	11	39	0	90	11	5	3	3	21	31	2	1	9	0	0	3
	2005	208	120	6	48	15	7	0	10	34	0	88	11	5	3	3	21	30	2	1	9	0	0	3
	2006	213	127	6	51	16	8	0	10	36	0	86	10	6	3	3	21	30	2	1	9	0	0	2
	2007	249	150	7	60	18	9	0	12	43	0	99	11	6	3	3	24	33	2	5	9	0	0	2
	2008	281	180	8	72	22	11	0	14	52	0	101	11	7	4	3	26	35	2	2	10	0	0	2
	2009	306	210	9	84	25	14	0	16	61	0	96	10	7	3	3	25	33	2	1	9	0	0	2
	2010	310	220	10	88	26	14	0	17	64	0	90	9	6	3	2	23	31	2	2	8	0	0	2
	2011	292	215	9	86	26	14	0	16	63	0	77	8	6	3	2	19	28	1	1	7	0	0	3
	2012	262	192	8	77	23	13	0	15	57	0	70	7	6	3	2	17	26	1	1	6	0	0	2
	2013	226	167	7	67	20	11	0	13	50	0	59	5	5	2	1	15	22	1	1	5	0	0	1
	2014	193	141	6	56	17	9	0	11	43	0	52	5	5	2	1	13	19	1	2	4	0	0	1
	2015	169	124	5	49	15	8	0	9	38	0	45	4	4	2	1	11	17	1	2	3	0	0	1
Bulgaria	2000	1397	748	45	194	120	53	0	110	226	0	649	245	0	0	21	119	166	35	0	64	0	0	0
	2001	1332	714	50	191	98	70	0	86	219	0	618	228	0	0	17	134	169	13	0	56	0	0	0
	2002	1262	677	55	201	64	57	0	84	215	0	585	183	0	0	9	164	134	28	0	67	0	0	0
	2003	1182	641	47	227	70	36	0	63	199	0	541	214	0	0	24	121	117	16	0	49	0	0	0
	2004	1112	604	45	204	81	29	0	60	185	0	508	151	0	0	20	134	117	12	0	73	0	0	0
	2005	1057	575	50	186	85	15	0	43	196	0	482	170	15	7	18	96	105	15	0	58	0	0	0
	2006	1030	560	42	189	92	32	0	27	178	0	470	163	27	11	23	116	71	16	0	43	0	0	0
	2007	1017	557	22	194	137	22	0	45	137	0	460	149	27	4	19	113	92	9	0	48	0	0	0
	2008	1022	559	44	244	66	29	0	19	156	0	463	132	25	7	25	104	109	11	0	50	0	0	0
	2009	1020	559	43	238	97	17	0	28	136	0	461	194	21	11	17	82	87	8	0	39	0	0	0
	2010	1001	552	44	218	96	28	0	29	138	0	449	151	22	8	17	83	107	8	11	41	0	0	0
	2011	949	514	21	206	98	20	0	32	136	0	435	119	37	6	13	91	119	9	0	42	0	0	0
	2012	861	464	22	222	72	28	0	11	109	0	397	112	25	4	13	87	94	12	0	48	0	0	0
	2013	773	412	23	174	71	21	0	19	104	0	361	108	24	5	12	74	90	8	3	37	0	0	0
	2014	689	367	20	155	64	18	0	17	92	0	322	96	21	5	11	66	80	7	3	33	0	0	0
	2015	634	335	18	142	58	17	0	16	84	0	299	89	20	4	10	61	75	7	2	31	0	0	0
Canada	2000	2041	1189	11	463	157	54	0	139	366	0	852	42	51	7	30	307	256	8	0	148	0	0	3
	2001	2005	1207	11	473	159	55	0	139	371	0	798	40	30	27	22	282	237	2	0	158	0	0	0
	2002	2011	1217	10	478	160	55	0	139	375	0	794	32	30	25	21	283	232	7	0	164	0	1	0
	2003	2014	1278	11	502	167	59	0	145	395	0	736	35	30	29	21	264	221	4	0	130	0	0	1
	2004	2065	1350	11	532	175	62	0	152	418	0	715	28	33	31	18	263	219	1	0	123	0	0	0
	2005	2122	1393	11	552	181	64	0	155	431	0	729	29	33	28	18	254	230	5	0	129	0	2	1
	2006	2149	1439	11	570	185	66	0	159	447	0	710	35	30	26	21	262	210	3	0	124	0	0	0
	2007	2181	1451	11	575	186	67	0	160	453	0	730	46	35	30	20	250	214	4	0	130	0	0	0
	2008	2206	1457	11	577	185	68	0	160	456	0	749	23	33	30	9	322	214	2	0	115	0	0	1
	2009	2217	1489	11	591	188	69	0	163	467	0	728	42	33	27	25	264	220	8	0	108	0	0	1
	2010	2201	1464	10	580	184	69	0	160	461	0	737	32	33	32	22	248	213	6	0	149	0	0	1
	2011	2121	1418	9	560	177	67	0	155	449	0	703	31	35	27	16	253	214	3	0	121	0	0	4
	2012	2056	1356	9	537	168	64	0	148	430	0	700	34	33	27	21	247	209	6	0	122	0	0	2
	2013	1978	1288	8	509	159	62	0	140	410	0	690	33	32	27	20	243	206	6	0	120	0	0	2
	2014	1872	1227	7	483	150	59	0	133	394	0	645	31	30	25	19	227	193	5	0	113	0	0	2
	2015	1824	1181	7	464	143	58	0	128	380	0	643	31	30	25	19	228	192	5	0	112	0	0	2

U5D[1]: total number of under 5 death; NEO[2]: total number of neonatal death; PNE[3]: pneumonia; PRE[4]: preterm; INT[5]: intrapartum related events; SEP[6]: sepsis/meningitis; TET[7]: tetanus; OTH[8]: other disorders; CON[9]: congenital; DIA[10]: diarrea; POS[11]: total number of post neonatal death; MEN[12]: meningitis; MEA[13]: measles; INJ[14]: injury; MAL[15]: malaria; PER[16]: pertussis

			0-28 days										1-59 months											
	Year	U5D[1]	NEO[2]	PNE[3]	PRE[4]	INT[5]	SEP[6]	TET[7]	OTH[8]	CON[9]	DIA[10]	POS[11]	PNE[3]	PRE[4]	INT[5]	MEN[12]	OTH[8]	CON[9]	DIA[10]	MEA[13]	INJ[14]	MAL[15]	AIDS	PER[16]
Croatia	2000	437	294	13	127	27	22	0	18	86	0	143	11	10	3	6	31	46	1	0	35	0	0	0
	2001	403	268	7	127	22	11	0	22	80	0	135	7	13	2	7	25	57	2	0	22	0	0	0
	2002	355	228	1	106	33	13	0	13	62	0	127	4	14	6	6	36	48	1	0	11	0	0	1
	2003	304	189	6	88	12	23	0	12	49	0	115	6	12	4	3	30	40	1	0	19	0	0	0
	2004	263	161	8	67	13	16	0	12	45	0	102	1	8	1	1	27	38	0	0	24	0	0	1
	2005	240	143	7	37	18	12	0	18	51	0	97	8	3	1	8	23	40	0	0	13	0	0	0
	2006	234	141	4	47	16	11	0	16	47	0	93	5	10	0	1	20	40	1	0	15	0	0	0
	2007	242	152	6	38	13	22	0	25	47	0	90	7	7	4	0	24	28	2	0	19	0	0	0
	2008	255	166	6	54	8	18	0	31	49	0	89	5	11	0	4	25	26	4	0	11	0	0	2
	2009	263	177	2	52	13	10	0	32	67	0	86	4	7	0	5	26	30	0	0	13	0	0	0
	2010	263	174	4	39	10	18	0	58	45	0	89	4	4	3	6	29	36	0	0	7	0	0	1
	2011	248	163	2	39	16	10	0	54	42	0	85	0	6	5	5	38	24	0	0	7	0	0	0
	2012	229	145	3	39	16	9	0	40	39	0	84	2	6	1	8	29	29	0	0	9	0	0	0
	2013	205	126	4	23	9	6	0	57	28	0	79	6	0	4	3	33	24	0	0	9	0	0	0
	2014	182	108	2	25	10	6	0	38	27	0	74	2	4	3	5	30	23	0	0	8	0	0	0
	2015	165	98	2	22	9	6	0	35	24	0	67	2	3	3	4	27	21	0	0	7	0	0	0
Cyprus	2000	84	40	1	16	5	2	0	5	12	0	44	4	2	2	1	13	14	0	0	6	0	0	1
	2001	75	36	1	14	4	2	0	4	11	0	39	3	2	2	1	11	13	0	0	6	0	0	1
	2002	70	33	0	13	4	2	0	4	10	0	37	3	2	2	1	11	12	0	0	5	0	0	1
	2003	65	30	0	12	4	2	0	3	9	0	35	3	2	2	1	10	12	0	0	5	0	0	1
	2004	61	29	0	11	3	2	0	3	9	0	32	2	2	2	1	9	11	0	0	4	0	0	1
	2005	57	28	0	11	3	2	0	3	9	0	29	2	2	1	1	8	10	0	0	4	0	0	1
	2006	56	28	0	11	3	2	0	3	9	0	28	2	2	2	1	8	10	0	0	4	0	0	1
	2007	53	27	0	10	3	2	0	3	9	0	26	1	3	1	1	7	9	0	0	3	0	0	1
	2008	52	27	0	10	3	2	0	3	9	0	25	1	2	1	1	7	9	0	0	3	0	0	1
	2009	49	26	0	10	3	2	0	3	9	0	23	1	2	1	0	6	8	0	0	3	0	0	1
	2010	47	25	0	9	3	1	0	3	8	0	22	1	1	2	0	6	8	0	0	3	0	0	1
	2011	45	24	0	9	3	1	0	3	8	0	21	1	2	1	0	6	8	0	0	2	0	0	1
	2012	43	23	0	9	2	1	0	3	8	0	20	1	2	1	0	5	7	0	0	2	0	0	1
	2013	40	21	0	8	2	1	0	2	7	0	19	1	2	1	0	5	7	0	0	2	0	0	1
	2014	38	20	0	7	2	1	0	2	7	0	18	1	2	1	0	5	7	0	0	2	0	0	0
	2015	35	19	0	7	2	1	0	2	6	0	16	1	2	1	0	4	6	0	0	2	0	0	0
Czech Republic	2000	491	276	4	92	53	13	0	57	58	0	215	17	13	8	8	74	41	1	0	53	0	0	0
	2001	472	275	5	79	40	5	0	66	79	0	197	20	18	4	9	65	54	0	0	28	0	0	0
	2002	495	288	2	96	45	8	0	62	75	0	207	20	25	6	5	70	43	1	0	38	0	0	0
	2003	521	316	13	82	47	10	0	79	85	0	205	19	16	11	5	65	54	2	0	33	0	0	0
	2004	553	341	2	120	53	19	0	91	56	0	212	12	24	5	12	77	41	4	0	37	0	0	0
	2005	564	347	7	132	50	32	0	46	80	0	217	17	24	10	7	55	58	0	0	44	0	0	0
	2006	571	352	9	104	70	19	0	79	70	0	219	24	8	6	3	85	47	0	0	46	0	0	0
	2007	555	339	5	120	73	20	0	23	100	0	216	21	20	9	13	61	36	6	0	50	0	0	0
	2008	541	332	11	126	71	37	0	24	63	0	209	18	35	9	5	41	65	4	0	33	0	0	0
	2009	517	306	7	101	67	55	0	16	60	0	211	14	31	8	12	54	54	6	0	29	0	0	1
	2010	486	288	11	95	68	32	0	30	52	0	198	26	14	13	4	56	52	1	0	33	0	0	0
	2011	451	266	4	91	40	32	0	32	66	0	185	17	19	8	7	59	38	3	0	33	0	0	0
	2012	419	229	1	89	45	11	0	19	64	0	190	19	31	6	4	57	40	6	0	27	0	0	0
	2013	386	204	4	81	34	21	0	21	43	0	182	24	25	3	5	59	36	3	0	27	0	0	0
	2014	347	183	3	69	31	17	0	19	45	0	164	18	22	5	5	52	34	3	0	26	0	0	0
	2015	328	168	2	63	28	15	0	17	42	0	160	17	22	5	5	50	33	3	0	25	0	0	0
Denmark	2000	359	221	1	98	26	4	0	22	70	0	138	7	8	3	6	43	50	2	0	18	0	0	0
	2001	350	213	2	90	29	5	0	24	62	0	137	8	9	3	8	52	41	4	0	12	0	0	0
	2002	343	207	1	93	20	5	0	19	70	0	136	9	6	8	6	44	42	1	0	19	0	0	0
	2003	338	203	0	94	25	1	0	20	62	0	135	10	7	1	6	36	55	0	0	19	0	0	1
	2004	329	199	2	108	20	3	0	17	49	0	130	4	12	6	7	31	50	2	0	18	0	0	0
	2005	319	201	0	94	27	5	0	18	57	0	118	7	12	4	3	35	35	0	0	22	0	0	1
	2006	309	198	1	97	24	6	0	15	54	0	111	3	8	4	1	39	42	1	0	12	0	0	0
	2007	300	194	1	83	26	6	0	27	50	0	106	1	14	1	3	38	35	1	0	12	0	0	0
	2008	289	196	0	100	28	4	0	21	42	0	93	2	7	0	2	31	36	3	0	12	0	0	0
	2009	282	189	0	112	15	2	0	31	29	0	93	7	10	0	2	39	25	2	0	9	0	0	0
	2010	265	186	0	95	22	4	0	26	39	0	79	3	8	0	2	29	26	2	0	9	0	0	0
	2011	250	172	0	88	20	4	0	24	36	0	78	3	8	0	2	29	26	2	0	9	0	0	0
	2012	231	162	0	82	19	4	0	22	34	0	69	2	7	0	2	26	23	1	0	8	0	0	0
	2013	211	145	0	74	17	3	0	20	30	0	66	2	7	0	2	24	22	1	0	7	0	0	0
	2014	196	137	0	70	16	3	0	19	29	0	59	2	6	0	1	22	19	1	0	7	0	0	0
	2015	187	129	0	66	15	3	0	18	27	0	58	2	6	0	1	22	19	1	0	6	0	0	0
Estonia	2000	135	77	5	25	6	15	0	8	18	0	58	5	0	0	1	7	19	2	0	24	0	0	0
	2001	131	74	4	22	8	17	0	4	19	0	57	2	1	0	2	18	17	1	0	16	0	0	0
	2002	126	73	2	19	16	11	0	3	23	0	53	6	0	0	2	11	16	0	0	18	0	0	0
	2003	120	69	1	19	18	8	0	4	19	0	51	3	1	0	1	10	16	1	0	18	0	0	0
	2004	114	66	0	28	13	5	0	3	17	0	48	5	6	0	1	11	14	0	0	10	0	1	0
	2005	110	62	0	13	19	4	0	4	22	0	48	4	0	0	2	16	10	0	0	16	0	0	0
	2006	104	59	2	25	5	3	0	3	22	0	45	0	1	1	1	10	16	0	0	15	0	0	0
	2007	98	56	0	20	6	9	0	6	14	0	42	2	5	0	1	10	15	0	0	7	0	0	1
	2008	90	52	1	12	7	5	0	7	19	0	38	1	0	2	3	9	9	0	0	13	0	1	0
	2009	83	47	1	8	11	8	0	6	13	0	36	2	1	1	2	9	10	0	0	10	0	0	0
	2010	74	41	1	4	14	7	0	4	10	0	33	4	0	0	2	8	6	1	0	11	0	0	0
	2011	66	36	0	5	7	7	0	4	13	0	30	2	2	0	2	8	8	0	0	9	0	0	0
	2012	57	29	1	10	6	1	0	2	10	0	28	2	1	1	0	7	10	0	0	6	0	0	0
	2013	49	24	1	4	6	4	0	2	7	0	25	2	1	0	1	6	7	0	0	7	0	0	0
	2014	42	21	0	4	5	4	0	2	6	0	21	2	1	0	1	5	6	0	0	6	0	0	0
	2015	37	18	0	3	4	3	0	2	5	0	19	2	1	0	1	5	5	0	0	5	0	0	0
Finland	2000	230	130	0	43	16	4	0	14	52	0	100	4	7	1	1	28	41	2	0	15	0	0	0
	2001	222	124	0	53	16	0	0	9	46	0	98	7	3	3	1	31	40	1	0	11	0	0	0
	2002	219	121	4	40	14	7	0	12	44	0	98	0	5	2	0	37	26	2	0	27	0	0	0
	2003	221	121	3	32	14	5	0	13	55	0	100	10	6	1	5	23	33	1	0	20	0	0	0
	2004	223	126	3	52	23	5	0	7	36	0	97	3	7	2	5	29	28	0	0	24	0	0	0
	2005	223	124	2	46	17	4	0	6	49	0	99	4	5	1	3	34	30	1	0	21	0	0	0
	2006	216	121	1	47	12	4	0	11	46	0	95	0	6	3	1	32	37	3	0	11	0	0	0
	2007	214	123	3	52	12	7	0	8	41	0	91	5	6	0	2	28	39	0	0	11	0	0	0
	2008	203	118	0	51	14	6	0	5	41	0	85	4	1	0	3	42	22	1	0	12	0	0	0
	2009	198	112	1	40	18	1	0	12	40	0	86	4	4	0	0	42	31	0	0	5	0	0	0
	2010	186	106	4	36	17	4	0	8	37	0	80	3	2	0	2	39	22	0	0	11	0	0	0
	2011	179	99	2	32	11	5	0	13	36	0	80	6	5	1	1	33	22	0	0	11	0	0	0
	2012	164	91	0	22	14	6	0	9	41	0	73	2	8	4	0	31	13	0	0	15	0	0	0
	2013	156	83	0	30	14	3	0	11	26	0	73	3	2	0	2	33	22	0	0	10	0	0	0
	2014	142	76	1	23	11	4	0	9	28	0	66	3	4	1	1	29	17	0	0	11	0	0	0
	2015	134	75	1	23	11	4	0	9	28	0	59	3	4	1	1	26	15	0	0	10	0	0	0
France	2000	4096	2140	7	548	435	150	0	254	746	0	1956	53	172	65	80	761	404	61	0	341	0	3	16
	2001	4065	2083	8	560	449	119	0	310	636	0	1982	48	156	91	73	761	408	59	0	382	0	1	2
	2002	3940	2097	8	526	492	130	0	370	571	0	1843	47	135	66	71	766	387	36	0	331	0	3	0
	2003	3804	2029	7	516	405	129	0	345	627	0	1775	55	114	74	63	746	349	57	0	313	0	2	3
	2004	3663	1957	2	530	364	132	0	349	579	0	1706	32	103	52	59	761	381	45	0	270	0	0	3
	2005	3595	1882	7	491	366	111	0	362	544	0	1713	58	162	65	42	711	358	41	0	262	0	2	14
	2006	3529	1807	4	476	348	116	0	356	506	0	1722	36	131	64	68	703	419	36	0	258	0	0	7
	2007	3455	1807	2	483	317	102	0	380	523	0	1648	66	119	55	51	712	383	29	0	232	0	0	2
	2008	3378	1806	4	439	346	123	0	370	522	0	1572	40	114	56	64	671	339	46	0	232	0	3	8
	2009	3377	1805	6	450	338	102	0	379	530	0	1572	53	104	37	43	700	355	43	0	232	0	5	0
	2010	3376	1803	4	541	278	109	0	363	508	0	1573	55	102	48	55	609	450	36	0	216	0	0	2

U5D[1]: total number of under 5 death; NEO[2]: total number of neonatal death; PNE[3]: pneumonia; PRE[4]: preterm; INT[5]: intrapartum related events; SEP[6]: sepsis/meningitis; TET[7]: tetanus; OTH[8]: other disorders; CON[9]: congenital; DIA[10]: diarrea; POS[11]: total number of post neonatal death; MEN[12]: meningitis; MEA[13]: measles; INJ[14]: injury; MAL[15]: malaria; PER[16]: pertussis

			0-28 days									1-59 months												
	Year	U5D[1]	NEO[2]	PNE[3]	PRE[4]	INT[5]	SEP[6]	TET[7]	OTH[8]	CON[9]	DIA[10]	POS[11]	PNE[3]	PRE[4]	INT[5]	MEN[12]	OTH[8]	CON[9]	DIA[10]	MEA[13]	INJ[14]	MAL[15]	AIDS	PER[16]
	2011	3379	1726	2	503	289	115	0	371	447	0	1653	56	117	56	51	650	434	40	0	247	0	0	2
	2012	3383	1728	4	484	293	105	0	360	481	0	1655	56	111	48	51	676	427	41	0	240	0	2	1
	2013	3463	1807	4	507	306	110	0	377	503	0	1656	56	112	48	51	677	427	41	0	240	0	2	1
	2014	3462	1807	4	507	306	110	0	377	503	0	1655	56	111	48	51	676	427	41	0	240	0	2	1
	2015	3378	1726	4	484	293	105	0	360	480	0	1652	56	111	48	51	675	426	41	0	239	0	2	1
Germany	2000	4375	2292	7	1061	164	78	0	215	767	0	2083	67	206	62	74	677	696	15	2	279	0	5	0
	2001	4158	2166	8	1008	174	82	0	229	664	0	1992	99	193	51	52	656	663	11	2	264	0	2	0
	2002	3936	2078	7	1022	145	75	0	197	631	0	1858	84	209	53	75	554	604	14	0	264	0	2	0
	2003	3673	1966	8	936	174	53	0	188	608	0	1707	84	160	50	67	528	572	14	0	230	0	2	0
	2004	3426	1792	6	906	121	62	0	173	523	0	1634	78	178	48	67	517	523	11	0	210	0	0	1
	2005	3161	1718	10	876	118	60	0	155	500	0	1443	58	140	34	49	514	469	3	0	177	0	0	0
	2006	3022	1681	4	841	148	51	0	201	436	0	1341	48	117	44	61	445	442	10	0	170	0	3	0
	2007	2930	1606	7	821	123	51	0	158	446	0	1324	55	149	40	38	470	408	9	0	154	0	1	0
	2008	2872	1550	8	750	126	56	0	175	435	0	1322	61	136	30	32	487	415	12	0	149	0	0	0
	2009	2827	1568	2	734	121	53	0	167	490	0	1259	61	120	29	22	433	422	10	0	163	0	0	0
	2010	2784	1519	7	645	136	51	0	219	461	0	1265	34	154	25	22	415	444	13	0	157	0	0	0
	2011	2734	1532	7	656	156	59	0	199	456	0	1202	52	130	34	19	421	403	9	0	130	0	1	3
	2012	2687	1546	7	655	158	63	0	216	447	0	1141	38	128	37	23	386	393	11	0	126	0	0	0
	2013	2641	1493	7	583	144	50	0	234	476	0	1148	40	98	19	21	498	341	12	0	118	0	0	0
	2014	2594	1506	7	624	151	56	0	214	454	0	1088	40	111	28	20	407	354	10	0	117	0	0	1
	2015	2545	1449	7	600	145	54	0	206	437	0	1096	41	112	28	20	410	357	10	0	117	0	0	1
Greece	2000	813	583	4	337	56	13	0	10	162	0	230	19	0	0	9	40	124	0	0	38	0	0	0
	2001	761	530	4	261	84	10	0	1	168	0	231	17	0	1	5	27	136	0	0	42	0	1	1
	2002	710	490	3	284	53	10	0	4	137	0	220	13	0	0	1	43	119	0	0	44	0	0	0
	2003	661	451	6	248	72	3	0	11	110	0	210	19	1	0	5	34	118	0	0	32	0	0	0
	2004	624	412	2	245	41	8	0	11	107	0	212	22	0	0	4	48	115	0	0	23	0	0	0
	2005	588	385	7	225	38	0	0	8	106	0	203	26	0	0	2	53	98	0	0	24	0	0	0
	2006	564	370	0	217	36	3	0	7	108	0	194	30	0	0	3	30	103	0	0	27	0	0	0
	2007	552	354	0	219	24	1	0	7	103	0	198	27	0	0	2	29	111	0	0	29	0	0	0
	2008	538	349	3	228	28	0	0	12	78	0	189	29	0	0	4	25	100	0	0	31	0	0	0
	2009	545	353	3	233	15	3	0	10	89	0	192	17	0	0	5	32	110	0	0	28	0	0	0
	2010	536	343	2	180	36	6	0	12	106	0	193	14	0	0	1	30	126	0	0	22	0	0	0
	2011	537	344	0	176	32	1	0	13	121	0	193	14	0	0	2	45	112	0	0	20	0	0	0
	2012	536	343	0	201	20	2	0	7	113	0	193	21	0	0	1	34	119	0	0	17	0	0	0
	2013	529	339	1	184	29	3	0	11	112	0	190	16	0	0	1	36	117	0	0	19	0	0	0
	2014	507	331	1	179	28	3	0	11	109	0	176	15	0	0	1	33	109	0	0	18	0	0	0
	2015	489	307	1	166	26	3	0	10	101	0	182	15	0	0	1	34	112	0	0	19	0	0	0
Hungary	2000	988	601	16	387	30	4	0	45	118	0	387	54	54	17	11	86	114	1	0	50	0	0	0
	2001	928	574	6	373	33	14	0	28	122	0	354	37	76	9	18	66	110	2	0	37	0	0	0
	2002	891	552	9	356	35	11	0	33	108	0	339	23	48	5	11	99	98	4	0	51	0	0	0
	2003	875	543	11	366	35	7	0	20	104	0	332	38	60	8	8	85	94	1	0	38	0	0	0
	2004	861	529	8	348	46	8	0	29	91	0	332	31	62	8	16	95	83	2	0	34	0	0	0
	2005	845	515	17	314	33	8	0	23	120	0	330	37	42	8	18	91	97	2	0	35	0	0	0
	2006	815	500	14	279	31	5	0	35	136	0	315	24	41	8	13	93	91	1	0	44	0	0	0
	2007	774	479	15	293	28	6	0	21	115	0	295	35	34	13	10	82	86	1	0	34	0	0	0
	2008	735	452	11	267	30	24	0	25	95	0	283	19	47	12	10	82	83	4	0	26	0	0	0
	2009	683	423	13	247	29	19	0	21	95	0	260	19	43	6	9	67	95	1	0	20	0	0	0
	2010	650	393	5	250	15	11	0	16	95	0	257	20	46	4	8	67	90	0	0	22	0	0	0
	2011	616	374	7	198	35	15	0	21	98	0	242	25	37	3	7	70	75	3	0	20	0	0	0
	2012	590	353	7	199	33	12	0	21	81	0	237	24	31	13	4	71	62	6	0	26	0	0	0
	2013	564	333	6	197	35	12	0	15	68	0	231	19	33	8	12	81	58	2	0	18	0	0	0
	2014	543	316	6	177	31	12	0	17	73	0	227	22	32	8	8	71	62	4	0	21	0	0	0
	2015	518	304	6	171	29	11	0	16	70	0	214	21	31	7	7	67	59	3	0	20	0	0	0
Iceland	2000	16	8	0	5	2	0	0	1	0	0	8	0	0	0	0	4	3	0	0	1	0	0	0
	2001	15	8	0	4	1	0	0	1	2	0	7	0	0	0	0	4	2	0	0	1	0	0	0
	2002	14	7	0	3	1	0	0	1	2	0	7	0	0	1	1	4	1	0	0	1	0	0	0
	2003	14	7	0	2	1	0	0	1	3	0	7	0	0	1	1	4	0	0	0	0	0	0	0
	2004	14	7	0	2	1	0	0	1	3	0	7	0	0	1	1	5	0	0	0	0	0	0	0
	2005	13	7	0	1	1	0	0	1	3	0	6	0	0	0	0	5	0	0	0	0	0	0	0
	2006	13	6	0	1	1	1	0	1	1	0	7	0	0	0	0	5	1	0	0	0	0	0	0
	2007	13	6	0	2	1	1	0	1	1	0	7	0	0	0	0	6	1	0	0	0	0	0	0
	2008	13	6	0	3	1	1	0	1	1	0	7	0	1	0	0	5	1	0	0	0	0	0	0
	2009	12	6	0	4	0	1	0	1	0	0	6	0	1	0	0	4	1	0	0	0	0	0	0
	2010	12	6	0	3	0	1	0	1	0	0	6	0	1	0	0	4	1	0	0	0	0	0	0
	2011	11	5	0	3	1	1	0	1	0	0	6	0	1	0	0	4	1	0	0	0	0	0	0
	2012	11	5	0	3	1	1	0	1	0	0	6	0	1	0	0	4	1	0	0	0	0	0	0
	2013	10	5	0	3	1	1	0	1	0	0	5	0	0	0	0	3	1	0	0	0	0	0	0
	2014	10	4	0	2	0	1	0	0	0	0	6	0	1	0	0	4	1	0	0	0	0	0	0
	2015	9	4	0	2	0	1	0	0	0	0	5	0	0	0	0	3	1	0	0	0	0	0	0
Ireland	2000	412	236	1	79	17	9	0	15	115	0	176	12	29	0	21	41	57	0	4	13	0	0	0
	2001	410	235	1	78	17	9	0	15	115	0	175	15	17	1	12	36	59	0	0	33	0	0	1
	2002	392	226	1	75	16	8	0	14	110	0	166	3	5	1	13	64	57	1	0	20	0	0	0
	2003	373	217	1	72	16	8	0	14	106	0	156	12	9	0	12	40	61	2	0	20	0	0	0
	2004	350	202	1	67	15	7	0	13	99	0	148	5	19	2	12	54	42	0	0	14	0	0	0
	2005	334	195	1	65	14	7	0	12	95	0	139	11	9	3	14	42	45	0	0	15	0	0	0
	2006	327	189	1	63	14	7	0	12	92	0	138	9	17	2	5	38	56	0	0	11	0	0	0
	2007	321	183	1	57	8	2	0	14	100	0	138	0	15	3	10	44	47	0	0	17	0	0	1
	2008	326	183	0	69	15	10	0	9	80	0	143	3	9	2	9	40	64	2	0	14	0	0	0
	2009	320	188	1	60	17	8	0	11	91	0	132	2	9	1	6	47	47	1	0	18	0	0	0
	2010	315	189	4	64	23	2	0	11	85	0	126	9	14	2	5	40	46	0	0	10	0	0	0
	2011	309	189	2	65	18	7	0	11	86	0	120	5	10	2	6	38	47	1	0	13	0	0	0
	2012	297	186	2	64	18	7	0	11	85	0	111	4	9	1	5	35	43	1	0	12	0	0	0
	2013	274	172	2	59	17	6	0	10	78	0	102	4	8	1	5	32	40	1	0	11	0	0	0
	2014	259	159	1	55	16	6	0	9	72	0	100	4	8	1	5	32	39	1	0	11	0	0	0
	2015	244	154	1	53	15	6	0	9	70	0	90	3	7	1	4	29	35	1	0	9	0	0	0
Israel	2000	896	474	5	231	23	25	0	46	145	0	422	10	39	13	1	208	90	7	0	53	0	0	0
	2001	871	467	3	220	28	26	0	28	162	0	404	13	52	2	6	156	103	6	0	63	0	2	2
	2002	848	441	2	215	36	14	0	44	130	0	407	17	37	2	15	171	103	2	0	59	0	3	0
	2003	808	426	2	195	31	21	0	43	134	0	382	5	52	1	12	158	100	5	0	48	0	0	0
	2004	770	386	4	153	31	18	0	32	149	0	384	24	40	3	7	144	102	5	0	55	2	0	3
	2005	753	363	3	159	20	13	0	36	132	0	390	21	48	3	5	122	146	2	0	44	0	0	0
	2006	724	343	1	155	18	17	0	38	114	0	381	7	33	4	14	152	114	2	0	53	0	0	2
	2007	714	352	1	132	36	12	0	39	131	0	362	10	30	4	5	140	125	1	0	42	0	0	4
	2008	707	349	1	128	26	19	0	49	127	0	358	19	27	4	10	132	118	0	0	46	0	0	1
	2009	700	361	1	144	27	12	0	52	125	0	339	12	34	3	8	117	136	3	0	25	0	0	0
	2010	706	372	0	170	25	17	0	37	123	0	334	9	24	3	7	123	117	1	0	50	0	0	0
	2011	699	387	0	109	18	22	0	102	136	0	312	10	7	1	9	131	111	6	0	36	0	0	0
	2012	706	384	1	133	34	23	0	51	142	0	322	17	14	3	6	109	125	3	0	45	0	0	0
	2013	709	395	0	144	27	21	0	65	138	0	314	12	15	2	7	118	115	3	0	42	0	0	0
	2014	706	384	0	140	26	20	0	63	134	0	322	12	15	2	7	121	117	3	0	43	0	0	0
	2015	693	368	0	134	25	19	0	61	129	0	325	12	15	2	7	122	119	3	0	44	0	0	0
Italy	2000	2845	1749	10	841	235	53	0	108	502	0	1096	43	84	25	16	352	452	1	0	116	0	8	0
	2001	2715	1666	3	778	225	58	0	122	480	0	1049	43	75	24	18	366	405	8	1	109	0	0	0
	2002	2661	1650	6	784	214	45	0	144	457	0	1011	56	71	21	18	358	387	0	1	99	0	0	0
	2003	2562	1585	4	548	190	64	0	332	446	0	977	25	85	28	29	351	358	0	2	99	0	0	0
	2004	2507	1566	5	568	180	70	0	304	438	0	941	32	78	23	19	352	339	2	1	94	0	0	0
	2005	2488	1535	6	583	169	75	0	275	426	0	953	32	82	26	19	362	336	1	1	94	0	0	0

U5D[1]: total number of under 5 death; NEO[2]: total number of neonatal death; PNE[3]: pneumonia; PRE[4]: preterm; INT[5]: intrapartum related events; SEP[6]: sepsis/meningitis; TET[7]: tetanus; OTH[8]: other disorders; CON[9]: congenital; DIA[10]: diarrea; POS[11]: total number of post neonatal death; MEN[12]: meningitis; MEA[13]: measles; INJ[14]: injury; MAL[15]: malaria; PER[16]: pertussis

			0-28 days									1-59 months												
	Year	U5D[1]	NEO[2]	PNE[3]	PRE[4]	INT[5]	SEP[6]	TET[7]	OTH[8]	CON[9]	DIA[10]	POS[11]	PNE[3]	PRE[4]	INT[5]	MEN[12]	OTH[8]	CON[9]	DIA[10]	MEA[13]	INJ[14]	MAL[15]	AIDS	PER[16]
	2006	2460	1493	7	592	157	80	0	245	412	0	967	28	83	24	25	371	342	3	0	92	0	0	0
	2007	2413	1441	8	566	171	60	0	290	347	0	972	22	86	26	21	394	320	3	0	100	0	0	0
	2008	2348	1435	3	534	167	61	0	285	385	0	913	29	90	26	14	371	294	0	0	88	0	1	0
	2009	2325	1418	3	546	167	89	0	280	333	0	907	17	79	35	20	392	265	0	0	95	0	3	0
	2010	2229	1335	4	542	157	80	0	247	304	0	894	23	78	31	16	397	266	4	0	78	0	0	0
	2011	2124	1299	6	488	128	60	0	265	352	0	825	31	79	22	10	351	254	8	0	71	0	0	0
	2012	2004	1200	1	451	125	80	0	260	283	0	804	26	60	21	12	384	243	3	0	56	0	0	0
	2013	1882	1103	3	425	118	63	0	222	271	0	779	25	67	23	12	350	236	4	0	63	0	0	0
	2014	1776	1018	3	392	109	59	0	205	250	0	758	24	65	22	11	340	229	4	0	61	0	0	0
	2015	1693	998	3	385	107	57	0	201	245	0	695	22	60	20	10	312	210	4	0	56	0	0	0
Japan	2000	5316	2130	27	470	259	122	0	244	1008	0	3186	331	90	23	59	898	1118	33	11	621	0	0	1
	2001	5037	1987	31	460	223	106	0	232	936	0	3050	261	76	42	53	813	1110	57	7	632	0	0	0
	2002	4735	1952	31	405	201	95	0	190	1029	0	2783	285	83	41	41	766	982	44	6	536	0	0	0
	2003	4542	1799	26	415	198	113	0	212	834	0	2743	288	74	44	49	779	970	41	4	492	0	0	2
	2004	4354	1655	24	359	193	102	0	165	812	0	2699	234	67	34	36	805	971	42	3	507	0	0	0
	2005	4080	1523	21	340	183	100	0	185	694	0	2557	258	63	20	41	804	865	50	0	454	0	3	0
	2006	3930	1405	18	285	180	93	0	200	629	0	2525	221	80	30	31	810	863	52	0	438	0	0	0
	2007	3805	1403	23	305	144	104	0	191	636	0	2402	233	48	36	40	734	878	55	0	376	0	0	1
	2008	3693	1297	17	278	135	78	0	203	586	0	2396	243	73	27	23	742	873	50	0	365	0	0	0
	2009	3580	1299	20	293	159	81	0	158	588	0	2281	183	61	27	31	749	858	55	0	317	0	0	0
	2010	3461	1189	17	253	130	75	0	150	565	0	2272	207	75	18	41	675	858	66	0	331	0	0	0
	2011	3670	1078	24	221	120	66	0	161	487	0	2592	200	44	25	18	606	742	60	0	893	0	1	1
	2012	3222	1073	25	217	144	60	0	150	477	0	2149	190	47	29	14	697	828	68	0	274	0	0	3
	2013	3094	1065	16	205	118	48	0	168	511	0	2029	174	76	30	16	711	722	58	1	241	0	0	0
	2014	2964	1056	21	211	125	57	0	157	484	0	1908	167	50	25	14	599	680	55	0	315	0	0	1
	2015	2834	941	19	188	112	51	0	140	432	0	1893	166	50	25	14	595	675	55	0	312	0	0	1
Latvia	2000	321	190	9	54	45	6	0	18	58	0	131	6	7	5	4	26	51	0	0	32	0	0	0
	2001	311	189	9	49	41	5	0	11	74	0	122	9	4	6	5	29	33	2	0	34	0	0	0
	2002	310	189	10	48	45	10	0	18	58	0	121	4	4	2	1	33	36	3	0	36	0	1	0
	2003	311	191	11	47	55	8	0	11	58	0	120	5	1	5	5	33	36	1	0	34	0	0	0
	2004	305	192	12	50	58	15	0	10	46	0	113	4	4	9	3	27	39	0	0	27	0	0	0
	2005	294	192	13	41	57	11	0	16	53	0	102	9	3	2	4	26	22	2	0	33	0	0	0
	2006	283	184	14	39	66	7	0	9	48	0	99	4	4	6	4	30	28	0	0	24	0	0	0
	2007	267	176	12	36	66	5	0	9	47	0	91	7	3	3	1	24	35	3	0	16	0	0	0
	2008	248	165	11	30	65	11	0	5	44	0	83	0	2	3	4	25	33	0	0	17	0	0	0
	2009	227	152	20	28	42	12	0	6	45	0	75	3	3	2	2	21	32	0	0	11	0	0	0
	2010	207	139	2	18	72	16	0	2	29	0	68	5	4	1	0	23	20	1	0	14	0	0	0
	2011	187	123	5	26	51	9	0	3	28	0	64	3	4	3	4	14	24	0	0	13	0	0	0
	2012	167	109	4	30	38	13	0	1	23	0	58	4	2	7	0	16	20	0	0	9	0	0	2
	2013	149	97	3	20	42	10	0	2	21	0	52	3	3	3	1	15	17	0	0	10	0	0	0
	2014	137	87	3	18	37	9	0	2	19	0	50	3	2	3	1	14	17	0	0	9	0	0	0
	2015	129	82	3	17	35	9	0	1	18	0	47	3	2	3	1	13	16	0	0	9	0	0	0
Lithuania	2000	419	192	7	54	29	9	0	7	85	0	227	30	4	4	10	39	71	1	0	68	0	0	0
	2001	386	178	6	44	28	8	0	22	70	0	208	19	2	1	10	39	74	0	0	60	0	0	1
	2002	358	169	8	37	35	7	0	4	79	0	189	28	4	3	10	32	57	3	0	53	0	0	0
	2003	332	163	9	67	20	8	0	8	52	0	169	20	7	5	12	28	52	0	0	44	0	0	0
	2004	314	160	2	54	22	14	0	7	62	0	154	20	3	0	3	25	52	3	0	48	0	0	0
	2005	299	155	7	46	30	14	0	9	49	0	144	22	4	0	7	25	47	0	0	39	0	0	0
	2006	287	146	4	43	12	12	0	7	67	0	141	16	4	3	7	33	43	0	0	35	0	0	0
	2007	269	136	7	40	21	15	0	7	47	0	133	10	3	0	5	26	50	0	0	39	0	0	0
	2008	252	129	4	37	16	15	0	11	46	0	123	8	4	3	4	28	43	3	0	30	0	0	0
	2009	234	119	1	30	16	22	0	13	37	0	115	19	2	2	2	24	35	1	0	28	0	0	0
	2010	215	111	1	27	18	22	0	4	39	0	104	5	6	0	9	28	32	1	0	24	0	0	0
	2011	198	106	9	30	15	14	0	7	32	0	92	3	4	3	6	22	34	0	0	21	0	0	0
	2012	182	96	4	35	11	15	0	3	28	0	86	12	1	1	6	25	24	0	0	15	0	0	1
	2013	172	90	4	27	12	14	0	4	29	0	82	6	3	1	6	22	26	0	0	17	0	0	1
	2014	167	86	4	25	12	14	0	4	27	0	81	6	3	1	6	22	26	0	0	17	0	0	1
	2015	164	81	3	24	11	13	0	4	26	0	83	6	3	1	6	22	26	0	0	17	0	0	1
Luxembourg	2000	26	13	0	5	5	0	0	0	2	0	13	0	0	0	2	6	1	0	0	4	0	0	0
	2001	25	12	0	5	4	1	0	0	2	0	13	0	0	0	1	6	1	0	0	4	0	0	0
	2002	23	12	0	7	2	1	0	1	2	0	11	0	0	0	0	6	1	0	0	3	0	0	0
	2003	22	11	0	5	2	1	0	2	2	0	11	0	1	0	0	7	1	0	0	2	0	0	0
	2004	20	10	0	5	2	0	0	2	2	0	10	0	1	1	0	6	2	0	0	1	0	0	0
	2005	19	10	0	5	3	0	0	2	1	0	9	0	1	1	0	4	2	0	0	2	0	0	0
	2006	18	9	0	5	2	0	0	1	1	0	9	1	0	1	0	3	3	0	0	2	0	0	0
	2007	16	8	0	3	3	0	0	1	1	0	8	1	0	0	0	4	2	0	0	2	0	0	0
	2008	15	8	0	3	3	1	0	0	1	0	7	1	0	0	0	4	1	0	0	2	0	0	0
	2009	15	7	0	3	2	1	0	1	1	0	8	0	0	0	0	4	1	0	0	2	0	0	0
	2010	14	7	0	3	1	1	0	1	1	0	7	0	0	0	1	4	1	0	0	2	0	0	0
	2011	14	7	0	3	1	0	0	2	1	0	7	0	0	0	0	4	2	0	0	1	0	0	0
	2012	13	7	0	4	1	0	0	1	1	0	6	0	0	0	0	4	1	0	0	1	0	0	0
	2013	13	6	0	4	1	0	0	1	1	0	7	0	0	0	0	4	2	0	0	1	0	0	0
	2014	13	7	0	4	1	0	0	1	1	0	6	0	0	0	0	3	2	0	0	1	0	0	0
	2015	13	6	0	3	1	0	0	1	1	0	7	0	0	0	0	4	2	0	0	1	0	0	0
Malta	2000	37	24	1	13	2	1	0	1	6	0	13	0	1	0	2	7	1	0	0	2	0	0	0
	2001	34	22	0	10	2	1	0	1	8	0	12	0	1	0	1	6	2	0	0	2	0	0	0
	2002	31	20	0	8	2	1	0	1	8	0	11	0	1	0	0	5	3	0	0	2	0	0	0
	2003	28	18	0	7	1	1	0	1	9	0	10	0	1	0	0	5	3	0	0	1	0	0	0
	2004	25	16	0	8	1	0	0	1	7	0	9	1	2	0	0	3	2	0	0	1	0	0	0
	2005	24	16	0	8	1	0	0	1	6	0	8	1	1	0	0	3	2	0	0	1	0	0	0
	2006	23	15	0	8	1	0	0	0	6	0	8	1	1	0	0	3	3	0	0	1	0	0	0
	2007	24	16	0	8	1	0	0	1	7	0	8	0	1	0	0	4	3	0	0	1	0	0	0
	2008	25	17	0	6	1	0	0	1	8	0	8	0	1	0	0	3	3	0	0	1	0	0	0
	2009	26	17	0	7	1	0	0	1	9	0	9	0	0	0	1	4	3	0	0	1	0	0	0
	2010	26	18	0	7	1	0	0	1	9	0	8	0	0	0	1	3	3	0	0	0	0	0	0
	2011	26	18	0	7	1	0	0	1	9	0	8	1	0	0	1	4	2	0	0	0	0	0	0
	2012	26	18	0	7	1	0	0	1	9	0	8	1	0	0	1	3	2	0	0	1	0	0	0
	2013	25	17	0	7	1	0	0	1	9	0	8	1	0	0	1	4	2	0	0	1	0	0	0
	2014	24	16	0	6	1	0	0	1	8	0	8	1	0	0	1	4	2	0	0	0	0	0	0
	2015	24	16	0	6	1	0	0	1	8	0	8	1	0	0	1	4	2	0	0	0	0	0	0
Monaco	2000	1	1	0	0	0	0	0	0	0	0	0	0	0	0	0	0	0	0	0	0	0	0	0
	2001	1	1	0	0	0	0	0	0	0	0	0	0	0	0	0	0	0	0	0	0	0	0	0
	2002	1	1	0	0	0	0	0	0	0	0	0	0	0	0	0	0	0	0	0	0	0	0	0
	2003	2	1	0	0	0	0	0	0	0	0	1	0	0	0	0	0	0	0	0	0	0	0	0
	2004	2	1	0	0	0	0	0	0	0	0	1	0	0	0	0	0	0	0	0	0	0	0	0
	2005	1	1	0	0	0	0	0	0	0	0	0	0	0	0	0	0	0	0	0	0	0	0	0
	2006	1	1	0	0	0	0	0	0	0	0	0	0	0	0	0	0	0	0	0	0	0	0	0
	2007	1	1	0	0	0	0	0	0	0	0	0	0	0	0	0	0	0	0	0	0	0	0	0
	2008	1	0	0	0	0	0	0	0	0	0	1	0	0	0	0	0	0	0	0	0	0	0	0
	2009	1	0	0	0	0	0	0	0	0	0	1	0	0	0	0	0	0	0	0	0	0	0	0
	2010	1	0	0	0	0	0	0	0	0	0	1	0	0	0	0	0	0	0	0	0	0	0	0
	2011	1	0	0	0	0	0	0	0	0	0	1	0	0	0	0	0	0	0	0	0	0	0	0
	2012	1	1	0	0	0	0	0	0	0	0	0	0	0	0	0	0	0	0	0	0	0	0	0
	2013	1	1	0	0	0	0	0	0	0	0	0	0	0	0	0	0	0	0	0	0	0	0	0
	2014	2	1	0	0	0	0	0	0	0	0	1	0	0	0	0	0	0	0	0	0	0	0	0
	2015	2	1	0	0	0	0	0	0	0	0	1	0	0	0	0	0	0	0	0	0	0	0	0
Montenegro	2000	115	75	2	25	33	3	0	5	7	0	40	1	8	4	0	14	8	1	0	4	0	0	0

U5D[1]: total number of under 5 death; NEO[2]: total number of neonatal death; PNE[3]: pneumonia; PRE[4]: preterm; INT[5]: intrapartum related events; SEP[6]: sepsis/meningitis; TET[7]: tetanus; OTH[8]: other disorders; CON[9]: congenital; DIA[10]: diarrea; POS[11]: total number of post neonatal death; MEN[12]: meningitis; MEA[13]: measles; INJ[14]: injury; MAL[15]: malaria; PER[16]: pertussis

				0-28 days								1-59 months												
	Year	U5D[1]	NEO[2]	PNE[3]	PRE[4]	INT[5]	SEP[6]	TET[7]	OTH[8]	CON[9]	DIA[10]	POS[11]	PNE[3]	PRE[4]	INT[5]	MEN[12]	OTH[8]	CON[9]	DIA[10]	MEA[13]	INJ[14]	MAL[15]	AIDS	PER[16]
	2001	108	71	2	24	32	3	0	5	6	0	37	1	10	4	0	13	6	1	0	3	0	0	0
	2002	103	68	2	23	30	3	0	4	6	0	35	1	9	4	1	10	7	0	0	3	0	0	0
	2003	97	64	2	21	29	3	0	4	6	0	33	0	8	4	1	11	6	1	0	2	0	0	0
	2004	91	61	2	20	27	2	0	4	6	0	30	1	5	2	2	9	7	1	0	2	0	0	0
	2005	86	56	2	17	26	2	0	4	5	0	30	2	5	2	1	11	6	1	0	2	0	0	0
	2006	79	53	1	17	24	2	0	4	5	0	26	3	4	1	1	9	6	0	0	2	0	0	0
	2007	74	49	1	16	24	2	0	2	3	0	25	2	3	2	1	11	3	0	0	2	0	0	0
	2008	67	45	1	17	22	2	0	0	4	0	22	1	4	3	1	10	2	0	0	1	0	0	0
	2009	61	42	2	15	21	2	0	0	3	0	19	0	5	3	1	9	0	0	0	1	0	0	0
	2010	56	37	1	13	18	1	0	0	3	0	19	1	5	3	0	8	1	0	0	1	0	0	0
	2011	50	33	1	12	16	1	0	0	3	0	17	1	3	2	1	7	1	0	0	1	0	0	0
	2012	44	29	1	11	14	1	0	0	2	0	15	1	3	2	0	7	1	0	0	1	0	0	0
	2013	39	26	1	9	13	1	0	0	2	0	13	1	3	2	0	6	1	0	0	1	0	0	0
	2014	36	23	1	8	11	1	0	0	2	0	13	1	3	2	0	6	1	0	0	1	0	0	0
	2015	33	21	1	8	10	1	0	0	2	0	12	1	2	2	0	5	1	0	0	1	0	0	0
Netherlands	2000	1196	729	6	214	132	39	0	38	300	0	467	22	39	9	34	171	127	0	0	65	0	0	0
	2001	1188	738	3	247	114	42	0	48	284	0	450	25	28	7	34	158	142	2	0	54	0	0	0
	2002	1167	733	1	229	123	51	0	59	271	0	434	18	27	4	33	168	120	4	0	60	0	0	0
	2003	1147	726	3	203	136	54	0	48	281	0	421	19	29	2	29	135	144	1	0	62	0	0	0
	2004	1137	713	2	198	145	40	0	59	269	0	424	19	21	6	12	168	136	3	0	58	0	0	1
	2005	1093	690	3	214	130	67	0	49	227	0	403	22	27	6	22	150	114	2	0	58	0	0	0
	2006	1034	655	0	194	112	47	0	48	254	0	379	16	24	1	10	168	113	6	0	42	0	0	0
	2007	963	613	2	180	116	70	0	63	181	0	350	13	24	1	14	141	106	0	0	50	0	0	0
	2008	888	550	5	150	99	51	0	65	180	0	338	13	26	4	10	146	90	4	0	45	0	0	0
	2009	821	510	2	161	68	39	0	65	175	0	311	15	23	5	13	140	79	0	0	36	0	0	0
	2010	765	479	7	130	73	40	0	59	170	0	286	15	27	0	15	107	84	2	0	36	0	0	0
	2011	739	459	1	117	75	37	0	62	167	0	280	13	21	7	13	112	81	3	0	31	0	0	0
	2012	725	445	3	152	60	37	0	57	136	0	280	16	31	4	8	108	79	0	0	32	0	0	2
	2013	699	435	0	145	64	44	0	47	136	0	264	11	33	3	1	123	60	7	0	27	0	0	0
	2014	692	426	1	132	63	38	0	53	139	0	266	13	28	5	7	111	71	3	0	29	0	0	1
	2015	682	432	1	134	64	38	0	53	141	0	250	12	26	4	7	104	66	3	0	27	0	0	1
New Zealand	2000	401	187	3	79	24	4	0	19	58	0	214	20	11	2	17	57	58	2	0	47	0	0	1
	2001	390	182	1	75	21	4	0	16	65	0	208	9	24	2	21	59	43	5	0	44	0	0	1
	2002	382	184	4	84	26	9	0	11	50	0	198	12	19	3	15	35	53	1	0	57	0	0	1
	2003	384	183	2	82	21	14	0	20	45	0	201	10	7	6	11	57	68	2	0	40	0	0	0
	2004	381	182	3	74	27	15	0	18	45	0	199	30	7	3	12	39	61	3	0	43	0	0	1
	2005	385	182	5	70	27	10	0	20	50	0	203	19	15	4	14	54	45	2	0	49	0	0	1
	2006	392	183	6	68	23	11	0	15	61	0	209	22	16	9	3	63	54	1	0	40	0	0	0
	2007	399	189	6	73	21	4	0	28	57	0	210	24	12	1	8	63	47	1	0	54	0	0	0
	2008	412	202	15	71	24	11	0	23	58	0	210	19	22	3	11	52	44	1	0	59	0	0	0
	2009	414	213	10	77	36	10	0	22	58	0	201	18	19	1	3	48	48	8	0	55	0	0	0
	2010	410	222	7	93	29	13	0	17	63	0	188	17	11	4	7	57	36	0	0	57	0	0	0
	2011	403	226	15	96	18	13	0	17	67	0	177	12	16	4	11	35	44	3	0	53	0	0	0
	2012	389	215	10	87	27	12	0	18	61	0	174	14	14	3	7	43	39	3	0	51	0	0	0
	2013	372	208	10	84	26	12	0	17	59	0	164	14	13	3	6	40	37	3	0	48	0	0	0
	2014	356	195	9	79	25	11	0	16	55	0	161	13	13	3	6	39	36	3	0	47	0	0	0
	2015	342	184	9	74	23	10	0	15	52	0	158	13	13	3	6	39	36	3	0	46	0	0	0
Norway	2000	287	156	3	26	26	3	0	36	61	0	131	6	9	3	14	34	45	1	0	18	0	0	0
	2001	271	148	0	26	35	3	0	29	56	0	123	4	5	4	0	44	44	0	0	20	0	2	0
	2002	256	141	0	24	27	1	0	35	54	0	115	5	4	7	2	50	34	0	0	12	0	0	0
	2003	248	134	1	38	33	3	0	21	39	0	114	6	6	6	6	42	31	0	0	19	0	0	0
	2004	236	134	0	29	21	3	0	23	57	0	102	1	0	8	3	30	39	0	0	20	0	0	1
	2005	227	129	1	29	27	2	0	34	35	0	98	6	13	4	0	33	32	0	0	10	0	0	0
	2006	225	127	1	31	33	8	0	18	36	0	98	0	7	7	5	29	28	5	0	16	0	0	0
	2007	219	125	0	28	28	2	0	30	37	0	94	10	7	8	4	30	25	0	0	10	0	0	0
	2008	214	123	0	28	17	3	0	38	38	0	91	2	6	1	0	40	29	3	0	10	0	0	0
	2009	213	121	1	30	22	4	0	28	37	0	92	10	8	4	5	27	27	2	0	9	0	0	0
	2010	204	122	0	31	22	5	0	23	41	0	82	3	4	6	1	34	27	3	0	4	0	0	0
	2011	198	116	0	30	15	2	0	28	41	0	82	3	10	4	4	28	28	0	0	6	0	0	0
	2012	190	108	1	30	15	3	0	23	36	0	82	6	6	6	1	35	21	0	0	7	0	0	0
	2013	174	99	0	27	17	5	0	22	28	0	75	1	3	1	1	37	25	1	0	5	0	0	0
	2014	166	97	0	26	14	3	0	22	31	0	69	3	5	3	2	29	21	0	0	5	0	0	0
	2015	160	91	0	24	13	3	0	21	30	0	69	3	5	3	2	29	21	0	0	5	0	0	0
Poland	2000	3376	2060	30	755	274	197	0	77	727	0	1316	138	118	37	38	301	499	6	0	179	0	0	0
	2001	3068	1845	26	698	228	214	0	58	622	0	1223	113	117	38	34	279	470	6	0	165	0	1	0
	2002	2874	1753	24	692	188	196	0	78	575	0	1121	85	121	48	24	251	427	3	0	161	0	1	0
	2003	2785	1703	30	685	180	193	0	82	532	0	1082	98	113	36	27	232	415	1	0	157	0	3	0
	2004	2741	1681	26	692	161	173	0	80	548	0	1060	103	128	34	22	213	423	1	0	134	0	1	0
	2005	2760	1676	26	657	165	165	0	78	585	0	1084	102	127	36	22	223	411	7	0	153	0	0	1
	2006	2773	1688	30	784	166	140	0	68	501	0	1085	98	146	33	32	235	409	1	0	130	0	1	0
	2007	2806	1710	27	769	159	123	0	70	562	0	1096	102	124	40	23	219	439	7	0	142	0	0	0
	2008	2833	1722	25	842	136	113	0	89	517	0	1111	93	165	24	27	242	409	3	0	147	0	0	0
	2009	2716	1699	22	876	134	100	0	74	492	0	1017	106	125	42	23	180	431	7	0	103	0	1	0
	2010	2581	1632	22	794	122	99	0	63	533	0	949	88	114	25	20	197	398	5	0	100	0	1	0
	2011	2427	1516	15	745	118	86	0	71	482	0	911	69	134	20	19	164	401	4	0	98	0	1	0
	2012	2262	1411	15	706	122	71	0	53	445	0	851	70	96	11	17	166	395	0	0	95	0	1	0
	2013	2108	1290	19	631	105	33	0	60	442	0	818	92	91	12	15	183	334	4	0	87	0	0	0
	2014	1999	1215	14	599	99	54	0	53	396	0	784	71	97	13	15	156	343	2	0	85	0	1	0
	2015	1917	1122	13	553	92	50	0	49	365	0	795	72	98	13	16	159	348	2	0	86	0	1	0
Portugal	2000	776	368	5	136	50	19	0	51	107	0	408	24	6	0	34	132	137	1	0	70	2	1	0
	2001	727	348	5	129	46	18	0	48	102	0	379	24	18	3	10	141	100	4	0	75	0	4	0
	2002	670	332	4	122	44	17	0	46	99	0	338	26	20	11	28	102	72	1	0	76	0	2	0
	2003	625	315	4	114	41	17	0	44	95	0	310	25	9	4	19	96	89	2	0	67	0	0	0
	2004	575	295	3	107	37	16	0	41	90	0	280	17	9	5	10	98	83	1	0	53	0	2	0
	2005	529	261	2	94	33	14	0	37	81	0	268	16	9	5	10	94	82	1	0	50	0	1	0
	2006	489	245	2	88	30	14	0	35	77	0	244	13	8	4	8	91	74	1	0	45	0	1	0
	2007	454	227	3	81	28	13	0	31	71	0	227	8	5	5	4	84	85	0	0	33	0	2	0
	2008	428	218	3	78	26	12	0	29	69	0	210	10	5	3	2	91	62	0	0	38	0	0	0
	2009	400	209	3	75	25	12	0	28	66	0	191	17	6	5	2	67	55	1	0	37	0	0	0
	2010	376	201	3	72	24	12	0	27	64	0	175	4	5	3	6	79	45	0	0	34	0	0	0
	2011	356	194	3	70	23	11	0	26	62	0	162	9	6	4	4	61	56	0	0	23	0	0	0
	2012	346	189	3	68	22	11	0	25	60	0	157	2	5	3	1	75	47	0	0	19	0	0	4
	2013	338	184	2	66	22	10	0	24	59	0	154	5	5	3	3	67	46	0	0	23	0	0	1
	2014	321	171	2	62	20	10	0	22	55	0	150	5	5	3	3	65	45	0	0	23	0	0	1
	2015	304	167	2	60	19	10	0	22	54	0	137	4	4	3	3	60	41	0	0	21	0	0	1
Republic of Moldova	2000	1490	974	217	87	200	122	0	50	297	0	516	175	0	1	25	82	85	20	0	127	0	0	0
	2001	1312	869	207	64	199	70	0	45	283	0	443	143	0	1	10	43	99	24	0	124	0	0	0
	2002	1142	765	175	46	153	77	0	25	290	0	377	105	0	0	14	52	88	17	1	100	0	1	0
	2003	1004	680	187	58	139	71	0	23	202	0	324	95	0	0	12	49	70	14	0	84	0	0	0
	2004	902	620	126	67	124	48	0	29	228	0	282	71	0	0	11	47	69	12	0	72	0	0	0
	2005	837	588	163	67	80	56	0	19	203	0	249	65	0	0	16	32	55	9	0	71	0	1	0
	2006	804	575	151	42	109	33	0	29	211	0	229	71	0	0	4	41	53	5	1	54	0	0	0
	2007	797	585	187	49	79	65	0	37	169	0	212	53	0	0	6	25	46	6	0	75	0	0	0
	2008	801	598	104	90	84	78	0	24	218	0	203	56	1	1	9	27	53	8	0	49	0	0	0
	2009	805	608	110	98	93	102	0	19	185	0	197	52	0	0	12	34	44	7	0	48	0	0	0
	2010	807	612	97	132	107	52	0	37	188	0	195	48	1	3	10	33	55	3	0	41	0	0	0
	2011	794	605	79	129	111	77	0	29	179	0	189	47	0	0	15	31	46	6	0	43	0	1	0

U5D[1]: total number of under 5 death; NEO[2]: total number of neonatal death; PNE[3]: pneumonia; PRE[4]: preterm; INT[5]: intrapartum related events; SEP[6]: sepsis/meningitis; TET[7]: tetanus; OTH[8]: other disorders; CON[9]: congenital; DIA[10]: diarrea; POS[11]: total number of post neonatal death; MEN[12]: meningitis; MEA[13]: measles; INJ[14]: injury; MAL[15]: malaria; PER[16]: pertussis

			0-28 days										1-59 months											
	Year	U5D[1]	NEO[2]	PNE[3]	PRE[4]	INT[5]	SEP[6]	TET[7]	OTH[8]	CON[9]	DIA[10]	POS[11]	PNE[3]	PRE[4]	INT[5]	MEN[12]	OTH[8]	CON[9]	DIA[10]	MEA[13]	INJ[14]	MAL[15]	AIDS	PER[16]
	2012	777	588	85	141	47	47	0	33	235	0	189	41	1	0	8	28	59	4	0	46	0	1	0
	2013	749	566	65	171	70	52	0	32	176	0	183	44	1	0	5	52	37	3	0	42	0	0	0
	2014	719	538	70	136	69	54	0	29	181	0	181	43	1	0	9	36	46	4	0	42	0	1	0
	2015	687	513	67	129	66	51	0	28	173	0	174	41	1	0	8	34	44	4	0	41	0	1	0
Romania	2000	7019	3493	475	1425	391	84	0	306	812	0	3526	1711	1	5	152	400	566	162	0	519	0	8	1
	2001	6799	3465	462	1316	339	107	0	313	927	0	3334	1595	5	2	151	364	544	132	0	538	0	2	0
	2002	6290	3313	443	1140	303	95	0	297	1034	0	2977	1418	4	1	157	346	518	122	0	407	0	5	0
	2003	5586	3025	364	1167	251	69	0	352	823	0	2561	1142	1	1	100	315	529	105	0	363	0	5	0
	2004	4912	2687	293	1254	172	67	0	260	642	0	2225	1116	4	1	73	218	358	91	0	362	0	2	0
	2005	4365	2421	262	1105	167	74	0	185	629	0	1944	929	5	0	66	179	403	68	9	283	0	2	0
	2006	3956	2227	227	1021	173	42	0	189	574	0	1729	817	2	0	60	209	307	57	2	274	0	1	0
	2007	3669	2074	275	920	147	18	0	174	539	0	1595	767	1	0	57	200	309	29	0	229	0	3	0
	2008	3438	1961	295	796	143	32	0	166	528	0	1477	749	2	0	37	190	238	34	0	227	0	0	0
	2009	3236	1847	222	798	113	32	0	151	531	0	1389	660	2	0	34	188	256	27	0	219	0	3	0
	2010	3010	1713	217	688	114	13	0	159	521	0	1297	648	0	0	39	177	223	30	0	178	0	2	0
	2011	2774	1550	195	654	125	30	0	99	447	0	1224	573	2	0	39	171	220	26	0	190	0	3	0
	2012	2495	1406	164	654	110	27	0	91	360	0	1089	501	4	0	20	158	242	16	3	144	0	1	0
	2013	2247	1256	155	540	95	19	0	93	355	0	991	472	2	0	26	139	190	19	1	140	0	2	0
	2014	2028	1126	139	484	85	17	0	83	319	0	902	429	2	0	24	127	173	18	1	128	0	2	0
	2015	1882	1047	129	450	79	16	0	77	296	0	835	397	1	0	22	117	160	16	1	118	0	1	0
Russian Federation	2000	33636	18482	1287	8011	2311	1676	0	1212	3984	0	15154	3344	322	193	701	3616	3090	591	0	2820	0	340	137
	2001	33123	18261	1214	7875	2265	1638	0	1210	4059	0	14862	3181	329	196	664	3542	3100	535	0	2765	0	408	141
	2002	31642	17450	1090	7479	2146	1543	0	1174	4017	0	14192	2908	325	194	608	3397	3029	472	0	2629	0	486	144
	2003	29534	16368	943	6979	1998	1430	0	1117	3901	0	13166	2556	313	185	538	3173	2879	399	0	2416	0	564	143
	2004	27451	15066	793	6394	1827	1288	0	1047	3716	0	12385	2305	309	182	489	3059	2812	344	0	2283	0	459	143
	2005	25573	13989	666	5932	1693	1149	0	990	3560	0	11584	2059	303	179	440	2915	2731	295	0	2135	0	385	142
	2006	23893	12925	553	5476	1560	1011	0	936	3388	0	10968	1843	299	175	396	2788	2654	255	0	1995	0	421	142
	2007	22343	12025	461	5099	1451	896	0	889	3229	0	10318	1639	292	169	353	2638	2554	223	0	1844	0	460	145
	2008	21141	11322	394	4805	1366	803	0	854	3099	0	9819	1485	287	165	320	2520	2476	201	0	1723	0	496	147
	2009	20221	10697	339	4541	1290	741	0	816	2971	0	9524	1392	291	166	298	2495	2466	189	0	1653	0	427	148
	2010	19607	10316	298	4380	1243	699	0	796	2899	0	9291	1317	296	167	279	2474	2463	180	0	1590	0	374	150
	2011	19314	10221	272	4339	1230	681	0	798	2900	0	9093	1240	298	167	260	2424	2434	170	0	1518	0	432	151
	2012	19145	10185	249	4321	1225	668	0	805	2918	0	8960	1143	322	179	288	2438	2406	156	0	1416	0	460	152
	2013	19260	10098	233	4282	1213	649	0	806	2914	0	9162	1152	342	190	286	2521	2510	156	0	1441	0	408	155
	2014	18916	10032	217	4249	1203	636	0	807	2919	0	8884	1115	353	196	273	2513	2535	150	0	1409	0	188	151
	2015	18542	9741	204	4121	1167	610	0	790	2850	0	8801	1087	356	198	262	2494	2537	146	0	1386	0	186	149
San Marino	2000	1	0	0	0	0	0	0	0	0	0	1	0	0	0	0	0	0	0	0	0	0	0	0
	2001	1	0	0	0	0	0	0	0	0	0	1	0	0	0	0	0	0	0	0	0	0	0	0
	2002	1	0	0	0	0	0	0	0	0	0	1	0	0	0	0	0	0	0	0	0	0	0	0
	2003	1	0	0	0	0	0	0	0	0	0	1	0	0	0	0	0	0	0	0	0	0	0	0
	2004	1	0	0	0	0	0	0	0	0	0	1	0	0	0	0	0	0	0	0	0	0	0	0
	2005	1	0	0	0	0	0	0	0	0	0	1	0	0	0	0	0	0	0	0	0	0	0	0
	2006	1	0	0	0	0	0	0	0	0	0	1	0	0	0	0	0	0	0	0	0	0	0	0
	2007	1	0	0	0	0	0	0	0	0	0	1	0	0	0	0	0	0	0	0	0	0	0	0
	2008	1	0	0	0	0	0	0	0	0	0	1	0	0	0	0	0	0	0	0	0	0	0	0
	2009	1	0	0	0	0	0	0	0	0	0	1	0	0	0	0	0	0	0	0	0	0	0	0
	2010	1	0	0	0	0	0	0	0	0	0	1	0	0	0	0	0	0	0	0	0	0	0	0
	2011	1	0	0	0	0	0	0	0	0	0	1	0	0	0	0	0	0	0	0	0	0	0	0
	2012	1	0	0	0	0	0	0	0	0	0	1	0	0	0	0	0	0	0	0	0	0	0	0
	2013	1	0	0	0	0	0	0	0	0	0	1	0	0	0	0	0	0	0	0	0	0	0	0
	2014	1	0	0	0	0	0	0	0	0	0	1	0	0	0	0	0	0	0	0	0	0	0	0
	2015	1	0	0	0	0	0	0	0	0	0	1	0	0	0	0	0	0	0	0	0	0	0	0
Serbia	2000	1607	985	13	469	233	75	0	22	174	0	622	74	32	32	23	114	250	15	0	77	0	6	0
	2001	1510	928	7	480	184	59	0	38	161	0	582	50	70	17	19	124	229	11	0	61	0	0	0
	2002	1371	836	8	413	197	49	0	28	140	0	535	40	54	37	11	170	175	3	0	46	0	0	0
	2003	1184	732	15	394	131	37	0	22	134	0	452	59	25	19	17	110	155	12	0	54	0	0	0
	2004	1014	627	2	333	105	33	0	26	129	0	387	55	19	9	14	89	164	0	0	35	0	0	2
	2005	897	547	13	284	113	39	0	12	87	0	350	42	18	26	20	93	122	8	0	22	0	0	0
	2006	815	498	9	261	101	24	0	16	87	0	317	25	31	19	11	99	107	0	0	25	0	0	0
	2007	765	476	10	278	76	9	0	19	84	0	289	39	31	17	12	69	93	5	0	24	0	0	0
	2008	744	460	1	264	79	10	0	22	84	0	284	35	11	11	4	87	112	0	0	24	0	0	0
	2009	720	447	15	240	104	7	0	15	65	0	273	31	26	15	10	84	86	0	0	20	0	2	0
	2010	697	437	6	269	73	9	0	10	71	0	260	24	41	21	3	60	84	0	0	28	0	0	0
	2011	668	428	9	249	56	14	0	24	75	0	240	28	17	19	8	76	72	0	0	21	0	0	0
	2012	651	412	6	243	65	4	0	23	71	0	239	24	37	16	2	85	52	0	0	23	0	0	0
	2013	635	405	11	249	69	8	0	16	52	0	230	16	31	18	4	65	72	0	0	24	0	0	0
	2014	628	398	8	237	61	9	0	20	63	0	230	22	27	17	4	73	64	0	0	22	0	0	0
	2015	619	389	8	232	60	8	0	20	61	0	230	22	27	17	4	73	64	0	0	22	0	0	0
Slovakia	2000	601	366	15	169	27	9	0	35	111	0	235	53	20	3	9	56	56	8	0	30	0	0	0
	2001	568	340	10	155	28	7	0	13	127	0	228	44	13	2	14	65	46	2	0	41	0	0	0
	2002	549	329	11	142	15	4	0	29	128	0	220	43	15	3	5	57	60	5	0	30	0	0	0
	2003	545	317	8	156	25	3	0	15	109	0	228	39	14	4	9	76	56	3	0	28	0	0	0
	2004	539	312	15	161	24	4	0	23	86	0	227	41	19	5	5	63	58	2	0	34	0	0	0
	2005	537	306	7	157	23	6	0	7	106	0	231	45	17	4	6	64	63	1	0	31	0	0	0
	2006	530	304	5	148	33	5	0	21	92	0	226	36	20	6	6	55	74	6	0	24	0	0	0
	2007	526	307	12	131	42	2	0	21	99	0	219	47	29	5	3	57	59	0	0	19	0	0	0
	2008	521	308	11	158	19	0	0	17	103	0	213	48	19	7	10	48	49	1	0	31	0	0	0
	2009	510	307	3	136	17	5	0	20	126	0	203	34	18	7	6	57	55	2	0	24	0	0	0
	2010	500	302	4	155	25	6	0	13	100	0	198	30	25	3	5	46	66	3	0	22	0	0	0
	2011	480	292	6	143	19	3	0	16	104	0	188	34	19	5	7	46	52	2	0	23	0	0	0
	2012	460	274	6	134	18	3	0	15	98	0	186	34	19	5	6	46	52	2	0	23	0	0	0
	2013	437	260	5	127	17	3	0	14	93	0	177	32	18	5	6	44	49	2	0	22	0	0	0
	2014	416	242	5	118	16	3	0	13	86	0	174	31	17	5	6	43	48	2	0	22	0	0	0
	2015	400	227	5	111	15	3	0	13	81	0	173	31	17	5	6	43	48	2	0	21	0	0	0
Slovenia	2000	101	61	0	31	6	5	0	0	20	0	40	0	2	2	0	10	19	0	0	7	0	0	0
	2001	95	57	1	19	10	5	0	8	14	0	38	1	1	0	0	14	21	0	0	0	0	0	0
	2002	90	54	0	20	7	4	0	1	21	0	36	2	0	0	3	8	16	1	0	5	0	0	0
	2003	83	49	0	24	2	2	0	4	17	0	34	4	1	1	0	14	7	1	0	5	0	0	0
	2004	78	45	0	23	4	2	0	3	14	0	33	1	3	0	0	10	13	1	0	4	0	0	1
	2005	76	44	0	27	4	0	0	2	10	0	32	1	3	3	1	9	10	1	0	5	0	0	0
	2006	73	42	0	24	0	7	0	2	9	0	31	0	3	2	0	10	11	0	0	6	0	0	0
	2007	73	42	0	12	4	3	0	2	20	0	31	3	1	0	0	13	8	0	0	6	0	0	0
	2008	73	43	1	20	5	8	0	0	9	0	30	0	6	2	0	11	8	0	0	4	0	0	0
	2009	74	41	0	21	5	2	0	2	10	0	33	2	4	2	0	8	10	2	0	6	0	0	0
	2010	72	40	0	28	2	4	0	2	4	0	32	2	7	0	0	15	4	0	0	4	0	0	0
	2011	69	38	0	21	4	4	0	2	7	0	31	1	6	1	0	11	7	0	0	4	0	0	0
	2012	67	36	0	20	4	4	0	2	7	0	31	1	6	1	0	11	7	0	0	4	0	0	0
	2013	63	34	0	18	3	4	0	1	6	0	29	1	5	1	0	11	7	0	0	4	0	0	0
	2014	60	33	0	18	3	4	0	1	6	0	27	1	5	1	0	10	6	0	0	4	0	0	0
	2015	57	31	0	17	3	4	0	1	6	0	26	1	5	1	0	9	6	0	0	4	0	0	0
Spain	2000	2439	1496	7	484	176	127	0	214	488	0	943	42	73	18	70	242	364	4	0	128	0	1	1
	2001	2436	1550	9	500	194	136	0	254	458	0	886	14	60	16	42	275	330	6	0	135	0	7	1
	2002	2514	1594	17	544	175	153	0	223	482	0	920	38	63	21	40	297	326	7	0	126	0	3	0
	2003	2614	1649	16	588	158	158	0	198	532	0	965	42	96	18	53	301	309	8	0	136	0	1	0
	2004	2665	1695	11	604	168	174	0	300	438	0	970	32	85	18	41	302	354	7	0	126	0	1	3
	2005	2679	1671	8	586	182	163	0	263	468	0	1008	31	93	31	56	314	349	6	0	122	0	4	1
	2006	2674	1675	26	544	192	142	0	282	490	0	999	40	97	30	46	330	328	15	0	112	0	0	0

U5D[1]: total number of under 5 death; NEO[2]: total number of neonatal death; PNE[3]: pneumonia; PRE[4]: preterm; INT[5]: intrapartum related events; SEP[6]: sepsis/meningitis; TET[7]: tetanus; OTH[8]: other disorders; CON[9]: congenital; DIA[10]: diarrea; POS[11]: total number of post neonatal death; MEN[12]: meningitis; MEA[13]: measles; INJ[14]: injury; MAL[15]: malaria; PER[16]: pertussis

			0-28 days										1-59 months											
	Year	U5D[1]	NEO[2]	PNE[3]	PRE[4]	INT[5]	SEP[6]	TET[7]	OTH[8]	CON[9]	DIA[10]	POS[11]	PNE[3]	PRE[4]	INT[5]	MEN[12]	OTH[8]	CON[9]	DIA[10]	MEA[13]	INJ[14]	MAL[15]	AIDS	PER[16]
	2007	2590	1610	16	578	169	193	0	240	414	0	980	50	108	22	45	312	293	17	0	127	0	0	6
	2008	2515	1571	11	530	185	170	0	248	427	0	944	30	84	26	38	316	320	4	0	120	0	0	7
	2009	2402	1557	13	520	222	167	0	260	376	0	845	38	79	28	44	283	266	12	0	92	0	1	1
	2010	2249	1465	9	456	200	116	0	247	436	0	784	33	60	17	24	278	258	6	0	104	0	0	4
	2011	2132	1410	6	419	218	155	0	205	407	0	722	28	73	17	26	237	267	3	0	62	0	1	8
	2012	1978	1327	10	449	169	108	0	253	337	0	651	20	46	17	19	270	199	4	0	68	1	0	6
	2013	1808	1194	7	385	155	121	0	225	301	0	614	16	40	16	26	267	165	3	0	79	0	0	4
	2014	1665	1081	6	346	148	106	0	189	286	0	584	18	46	15	21	229	184	3	0	62	0	0	5
	2015	1566	1039	6	332	143	102	0	182	275	0	527	17	42	13	19	207	166	2	0	56	0	0	5
Sweden	2000	310	163	2	34	20	15	0	22	71	0	147	4	17	8	4	31	69	0	0	12	0	0	1
	2001	318	173	1	31	27	13	0	40	61	0	145	5	13	2	3	57	48	0	0	17	0	0	0
	2002	340	186	3	46	33	14	0	29	61	0	154	5	12	3	6	64	49	0	0	14	0	0	2
	2003	373	204	5	49	35	15	0	30	71	0	169	9	14	14	5	63	48	0	0	15	0	0	0
	2004	393	218	0	29	42	17	0	38	92	0	175	11	6	3	4	63	40	1	0	44	0	0	2
	2005	408	223	0	45	42	18	0	43	75	0	185	10	20	4	5	78	52	1	0	12	1	0	0
	2006	397	216	0	50	38	19	0	30	79	0	181	6	13	5	4	71	63	3	0	16	0	0	0
	2007	387	215	0	54	36	14	0	33	78	0	172	9	5	3	8	80	52	3	0	10	0	2	0
	2008	368	199	4	36	26	21	0	48	64	0	169	19	8	2	2	58	57	3	0	17	0	0	3
	2009	349	182	2	49	32	13	0	26	60	0	167	8	14	1	1	74	49	0	0	19	0	1	0
	2010	345	178	0	39	30	14	0	38	57	0	167	11	11	4	4	82	45	1	0	8	0	0	0
	2011	332	176	7	35	38	12	0	31	53	0	156	13	13	6	0	71	37	1	0	14	0	0	0
	2012	333	176	3	49	36	10	0	33	45	0	157	6	7	5	3	84	44	2	0	7	0	0	0
	2013	335	176	1	38	32	9	0	38	58	0	159	10	23	2	1	65	44	1	0	11	0	1	0
	2014	338	178	4	41	35	11	0	35	53	0	160	10	15	4	1	75	43	2	0	11	0	0	0
	2015	343	181	4	42	36	11	0	35	54	0	162	10	15	4	1	76	43	2	0	11	0	0	0
Switzerland	2000	430	265	2	102	35	12	0	31	83	0	165	4	9	0	9	60	49	2	0	33	0	0	0
	2001	418	258	2	100	34	11	0	30	81	0	160	8	9	6	11	54	46	1	0	23	0	0	1
	2002	401	245	2	95	32	11	0	28	77	0	156	7	3	5	12	56	49	0	0	25	0	0	0
	2003	382	242	2	94	31	11	0	28	77	0	140	5	6	3	6	46	46	2	0	25	0	0	0
	2004	372	234	2	91	30	10	0	27	75	0	138	3	12	3	1	52	48	1	0	18	0	0	0
	2005	367	236	2	91	30	10	0	27	76	0	131	7	3	1	5	39	50	1	0	24	0	0	0
	2006	363	239	2	93	30	11	0	27	77	0	124	2	2	0	5	52	37	0	0	24	0	0	0
	2007	356	245	2	95	31	11	0	28	79	0	111	3	3	6	3	40	40	0	0	17	0	0	0
	2008	358	245	1	95	30	11	0	28	80	0	113	5	12	2	2	40	35	0	0	16	0	0	0
	2009	360	252	1	97	31	11	0	28	82	0	108	4	8	1	5	33	31	0	0	23	0	2	1
	2010	361	250	1	97	31	11	0	28	82	0	111	5	7	2	2	37	44	2	0	13	0	0	0
	2011	359	246	1	95	30	11	0	28	81	0	113	3	14	3	1	41	46	0	0	6	0	0	0
	2012	356	249	1	96	30	12	0	28	82	0	107	1	6	3	0	35	40	1	0	20	0	0	1
	2013	351	243	1	94	29	11	0	27	80	0	108	3	9	2	1	36	42	1	0	13	0	0	1
	2014	338	237	1	91	28	11	0	26	79	0	101	3	8	2	1	34	39	1	0	12	0	0	0
	2015	334	231	1	89	27	11	0	26	77	0	103	3	9	2	1	35	40	1	0	12	0	0	1
The former Yugoslav Republic of Macedonia	2000	417	237	1	147	46	10	0	2	31	0	180	33	2	0	4	55	38	36	0	12	0	0	0
	2001	389	220	1	135	41	11	0	1	32	0	169	36	2	1	8	42	46	22	0	12	0	0	0
	2002	369	214	1	129	37	12	0	1	33	0	155	30	2	1	7	42	46	18	0	11	0	0	0
	2003	361	215	0	128	36	14	0	1	36	0	146	28	1	1	7	41	44	14	0	10	0	0	0
	2004	355	222	0	131	35	17	0	0	39	0	133	25	1	1	7	37	42	11	0	10	0	0	0
	2005	343	222	0	129	33	19	0	0	42	0	121	23	2	0	2	30	41	11	0	11	0	0	0
	2006	323	212	2	141	18	17	0	6	29	0	111	21	0	2	4	28	49	0	0	7	0	0	0
	2007	295	197	2	123	19	6	0	7	40	0	98	17	0	0	12	21	36	3	0	9	0	0	0
	2008	267	177	0	112	21	16	0	5	24	0	90	8	3	0	5	30	34	6	0	3	0	0	0
	2009	242	159	0	116	14	6	0	5	19	0	83	17	0	0	5	33	23	2	0	4	0	0	0
	2010	215	140	2	108	14	4	0	1	11	0	75	13	0	0	9	16	26	2	0	9	0	0	0
	2011	188	122	1	87	12	6	0	3	13	0	66	10	1	0	5	21	22	3	0	5	0	0	0
	2012	167	108	1	77	11	5	0	2	12	0	59	9	1	0	4	19	20	2	0	4	0	0	0
	2013	154	99	1	70	10	5	0	2	11	0	55	8	1	0	4	17	18	2	0	4	0	0	0
	2014	145	92	1	65	9	5	0	2	10	0	53	8	1	0	4	17	18	2	0	4	0	0	0
	2015	135	87	1	62	9	4	0	2	10	0	48	7	1	0	4	15	16	2	0	3	0	0	0
Ukraine	2000	7543	4483	425	1849	529	298	0	316	1065	0	3060	686	101	64	119	543	652	151	10	424	0	273	37
	2001	6953	4125	376	1699	486	267	0	295	1002	0	2828	604	94	59	104	490	606	129	13	388	0	307	36
	2002	6481	3876	337	1595	456	245	0	281	963	0	2605	525	86	53	91	445	562	108	13	355	0	331	36
	2003	6171	3701	303	1523	435	232	0	271	938	0	2470	467	80	50	82	422	536	94	12	336	0	354	36
	2004	5989	3607	275	1486	424	227	0	265	930	0	2382	419	76	47	75	404	518	82	12	321	0	392	37
	2005	6008	3668	262	1513	431	234	0	268	958	0	2340	397	74	46	72	407	522	77	11	322	0	374	38
	2006	6176	3811	259	1574	448	244	0	278	1007	0	2365	379	77	47	81	426	531	73	21	321	0	366	41
	2007	6411	4013	261	1660	472	256	0	291	1073	0	2398	365	91	55	86	459	562	77	20	320	0	317	45
	2008	6660	4143	263	1716	488	256	0	300	1121	0	2517	378	110	67	92	538	632	102	18	339	0	189	51
	2009	6712	4219	263	1750	497	250	0	305	1155	0	2493	360	95	57	86	579	618	137	17	328	0	155	61
	2010	6588	4086	250	1696	481	232	0	297	1130	0	2502	352	84	51	81	612	615	172	26	323	0	119	67
	2011	6208	3833	227	1592	451	210	0	279	1072	0	2375	336	74	44	73	581	598	169	25	313	0	93	68
	2012	5628	3414	196	1418	402	183	0	250	965	0	2214	297	96	57	69	517	595	126	29	282	0	90	56
	2013	4968	3010	167	1249	353	158	0	223	860	0	1958	261	93	55	60	435	549	94	27	250	0	85	50
	2014	4418	2602	140	1077	305	135	0	195	750	0	1816	238	91	54	54	394	524	79	24	232	0	80	46
	2015	3997	2378	124	983	278	122	0	180	692	0	1619	207	83	50	46	350	477	68	22	204	0	71	41
United Kingdom	2000	4557	2601	32	1467	272	132	0	118	578	0	1956	152	127	10	130	762	500	58	0	211	0	3	3
	2001	4362	2503	44	1420	240	61	0	74	664	0	1859	149	162	27	143	726	442	4	0	193	0	5	7
	2002	4254	2415	33	1381	226	44	0	113	618	0	1839	121	220	33	113	697	447	3	1	196	0	3	5
	2003	4248	2415	37	1414	213	54	0	89	609	0	1833	117	201	20	126	725	448	7	0	185	0	3	1
	2004	4161	2383	26	1379	276	51	0	84	568	0	1778	122	221	40	107	686	421	4	0	169	0	1	5
	2005	4207	2459	35	1426	223	56	0	80	639	0	1748	132	202	30	107	645	464	11	0	150	0	1	7
	2006	4317	2510	34	1436	229	68	0	88	655	0	1807	124	154	30	76	780	456	7	0	174	0	1	3
	2007	4489	2592	37	1419	280	66	0	100	690	0	1897	123	190	25	80	851	452	4	0	164	0	1	6
	2008	4583	2672	43	1497	247	58	0	96	732	0	1911	145	240	36	77	793	439	4	0	165	0	3	9
	2009	4618	2706	40	1447	279	69	0	97	775	0	1912	144	272	18	81	750	482	2	0	160	0	0	3
	2010	4533	2670	45	1451	267	59	0	77	771	0	1863	133	233	42	79	757	460	3	0	155	0	0	2
	2011	4354	2557	33	1433	236	55	0	84	716	0	1797	148	215	35	68	722	444	26	0	128	0	1	10
	2012	4079	2388	32	1284	218	44	0	81	728	0	1691	122	218	25	52	686	427	12	0	141	0	0	10
	2013	3755	2196	22	1203	205	38	0	110	618	0	1559	93	144	25	32	785	347	10	0	119	0	0	4
	2014	3460	1946	24	1068	180	37	0	76	562	0	1514	108	172	25	45	661	364	14	0	116	0	0	7
	2015	3235	1820	22	999	168	35	0	71	526	0	1415	101	161	24	42	618	341	13	0	109	0	0	7
United States of America	2000	31515	17108	125	7704	1342	838	0	2622	4477	0	14407	863	933	162	294	4517	3496	633	2	3454	0	40	14
	2001	31613	17464	118	8029	1371	822	0	2792	4331	0	14149	887	912	140	236	4391	3509	639	0	3376	0	35	25
	2002	32070	18026	140	8040	1596	877	0	2850	4524	0	14044	859	914	170	214	4407	3485	629	0	3322	0	13	30
	2003	32671	18673	124	8676	1400	932	0	2951	4591	0	13998	1026	827	154	218	4387	3423	612	2	3327	0	11	11
	2004	33573	18816	107	8681	1330	1006	0	2995	4697	0	14757	955	1008	133	211	4581	3631	640	0	3563	0	12	22
	2005	33698	19156	114	9082	1368	1013	0	3025	4554	0	14542	903	931	115	228	4588	3608	625	2	3492	0	7	43
	2006	33518	18805	116	8757	1208	952	0	3015	4756	0	14713	934	954	141	204	4587	3612	638	0	3617	2	10	14
	2007	32977	18269	121	8466	1205	947	0	3009	4522	0	14708	820	889	112	198	4714	3705	640	0	3605	0	16	10
	2008	32172	18020	82	8387	1213	862	0	2993	4483	0	14152	876	843	132	175	4500	3512	614	0	3469	0	3	26
	2009	30845	17289	105	7975	1164	808	0	2948	4288	0	13556	932	706	102	175	4273	3353	594	0	3394	2	3	22
	2010	29940	16564	88	7555	1215	727	0	2741	4238	0	13376	744	565	113	218	4296	3399	577	0	3421	0	4	40
	2011	28688	15861	76	7186	1157	657	0	2696	4089	0	12827	815	543	104	125	4054	3295	478	0	3399	2	3	9
	2012	27790	15160	73	6916	1174	642	0	2454	3901	0	12630	645	539	114	101	4172	3219	458	0	3351	0	3	28
	2013	26598	14522	62	6634	1094	643	0	2553	3535	0	12076	589	422	77	135	4690	2575	339	0	3235	0	0	14
	2014	25627	14023	65	6386	1055	599	0	2372	3546	0	11604	632	464	91	112	4002	2804	393	0	3088	1	2	16
	2015	24923	13699	63	6238	1031	585	0	2317	3464	0	11224	611	449	88	108	3871	2713	380	0	2987	0	2	15
Eastern Asia	2000	615301	344503	50662	92061	96097	6481	3967	51219	41478	2538	270798	72850	3054	569	9444	50036	32923	30516	2032	67190	7	498	1680
	2001	580630	336638	46672	92365	94129	6543	3230	48511	43160	2029	243992	61291	3022	508	7843	46809	33765	25542	2421	60552	6	534	1701

U5D[1]: total number of under 5 death; NEO[2]: total number of neonatal death; PNE[3]: pneumonia; PRE[4]: preterm; INT[5]: intrapartum related events; SEP[6]: sepsis/meningitis; TET[7]: tetanus; OTH[8]: other disorders; CON[9]: congenital; DIA[10]: diarrea; POS[11]: total number of post neonatal death; MEN[12]: meningitis; MEA[13]: measles; INJ[14]: injury; MAL[15]: malaria; PER[16]: pertussis

			0-28 days										1-59 months											
	Year	U5D[1]	NEO[2]	PNE[3]	PRE[4]	INT[5]	SEP[6]	TET[7]	OTH[8]	CON[9]	DIA[10]	POS[11]	PNE[3]	PRE[4]	INT[5]	MEN[12]	OTH[8]	CON[9]	DIA[10]	MEA[13]	INJ[14]	MAL[15]	AIDS	PER[16]
	2002	542981	319748	41716	90633	87876	6434	2471	45390	43471	1727	223233	52715	2808	377	6778	44313	34047	23423	1919	54570	9	565	1709
	2003	502965	299908	37884	87595	80459	6134	1900	41837	42526	1572	203057	45150	2583	374	5962	41548	32163	21644	2037	49299	11	581	1706
	2004	461944	275499	33078	83069	70955	5389	1596	38055	41932	1425	186445	39562	2710	326	4979	38719	31457	19627	2031	44735	7	597	1695
	2005	423643	250419	27419	78389	62539	4668	1347	33738	41012	1306	173224	35339	2595	384	4335	35260	30279	16996	3089	42641	10	597	1700
	2006	387488	224336	22159	72180	56614	4023	1016	28783	38523	1038	163152	32953	2805	426	4224	32115	30201	14659	2543	40965	8	596	1658
	2007	352093	198406	17370	65386	50467	3701	864	24831	34900	886	153687	31217	2808	455	4208	29010	29962	12508	2737	38490	4	619	1670
	2008	318822	174182	14365	57266	44355	3754	726	22391	30606	720	144640	29631	2759	495	4305	26878	27801	10747	3272	36486	5	601	1659
	2009	289219	154637	12116	50825	39455	3666	496	20156	27349	573	134582	27599	2861	462	4226	25942	26996	9597	1481	33237	2	520	1660
	2010	266132	138724	10494	45455	34350	3646	273	18525	25365	616	127408	25028	3117	1114	3999	25884	26187	8220	836	30890	4	455	1674
	2011	247900	128124	10002	41065	30353	3725	127	17841	24296	715	119776	22492	3345	1951	3628	25532	24250	7273	480	28715	8	414	1689
	2012	233171	120228	9641	37967	27028	3669	67	17362	23582	911	112943	20366	3525	2826	3125	24572	22245	6619	324	27247	3	384	1706
	2013	218767	112387	9295	34730	23878	3626	35	16984	22671	1169	106380	18693	3897	4076	2645	23258	20058	5869	570	25285	6	418	1606
	2014	205968	106936	8968	32528	22619	3581	24	16528	21465	1222	99032	17082	3695	4382	2351	21627	18514	5329	523	23629	4	404	1493
	2015	193985	100048	8382	30432	21156	3366	23	15443	20104	1141	93937	16230	3507	4157	2230	20522	17573	5043	491	22382	3	383	1416
China	2000	585027	331419	49967	86546	93219	5217	3861	50390	39745	2475	253608	68125	2454	282	8477	45735	31882	27345	1995	65184	7	495	1628
	2001	554286	324671	46069	87387	91570	5317	3160	47716	41472	1982	229615	57382	2467	240	7038	43202	32848	23098	2371	58782	6	532	1651
	2002	519912	308734	41180	86246	85588	5225	2423	44635	41745	1692	211178	49482	2344	184	6105	41302	33174	21534	1872	52948	9	563	1661
	2003	482382	289686	37409	83503	78381	4996	1863	41149	40841	1545	192696	42430	2151	183	5399	38939	31345	20142	2016	47845	11	578	1658
	2004	443233	265937	32634	79309	69022	4314	1566	37387	40303	1402	177296	37206	2233	177	4484	36382	30723	18376	2016	43453	7	592	1648
	2005	406101	241394	27012	74798	60765	3644	1320	33107	39462	1286	164707	33162	2186	237	3886	32999	29587	15873	3076	41445	10	592	1654
	2006	370640	215710	21765	68769	54926	3043	991	28187	37008	1020	154930	30845	2391	289	3783	29890	29535	13598	2531	39854	8	593	1614
	2007	335768	190082	16693	62110	48824	2760	840	24242	33444	868	145686	29161	2429	310	3768	36846	29289	11489	2726	37421	4	616	1627
	2008	303084	166097	14002	54070	42768	2826	703	21796	29229	703	136987	27648	2385	357	3895	24830	27150	9794	3270	35438	5	598	1618
	2009	274052	146867	11764	47749	37940	2791	475	19589	26002	557	127185	25665	2491	323	3825	23965	26373	8682	1479	32243	2	517	1619
	2010	251554	131241	10157	42521	32887	2797	253	17966	24059	601	120313	23164	2730	975	3612	24024	25570	7370	834	29946	4	449	1634
	2011	233813	120859	9678	38219	28946	2912	109	17315	22979	701	112954	20692	2981	1818	3247	23705	23680	6481	477	27807	8	410	1649
	2012	219549	113103	9326	35197	25645	2864	51	16849	22273	898	106446	18722	3210	2702	2803	22760	21686	5863	322	26330	3	380	1664
	2013	205470	105373	8985	32024	22527	2833	19	16471	21356	1157	100097	17229	3562	3947	2402	21430	19508	5111	569	24354	6	414	1565
	2014	193048	100075	8667	29893	21305	2808	10	16028	20154	1210	92973	15660	3375	4258	2116	19860	17987	4618	523	22721	4	400	1453
	2015	181574	93435	8092	27910	19891	2622	9	14965	18816	1130	88139	14862	3201	4037	2005	18826	17069	4383	491	21503	3	379	1378
Democratic People's Republic of Korea	2000	23970	10615	615	4292	2544	1056	100	701	1254	53	13355	4037	400	162	835	3298	496	2777	7	1298	0	2	43
	2001	20313	9475	536	3650	2234	1077	62	659	1218	38	10838	3298	329	133	689	2692	414	2120	7	1112	0	2	41
	2002	17294	8512	468	3167	1957	1052	42	607	1191	28	8782	2687	271	109	566	2189	345	1615	7	951	0	2	40
	2003	15040	7771	417	2844	1747	981	31	553	1175	22	7269	2235	227	91	472	1816	293	1262	7	824	0	3	40
	2004	13495	7228	381	2624	1594	922	25	509	1154	19	6267	1934	199	79	408	1569	258	1036	8	734	0	3	39
	2005	12607	6793	355	2471	1481	860	22	470	1117	16	5814	1801	186	74	380	1460	244	926	8	695	0	3	38
	2006	12177	6530	339	2385	1414	828	21	447	1080	15	5647	1755	182	73	371	1426	241	874	8	679	0	3	35
	2007	11886	6308	327	2311	1363	802	20	430	1039	15	5578	1740	180	73	369	1417	240	844	8	673	0	3	33
	2008	11469	6101	315	2237	1316	779	20	416	1004	14	5368	1683	174	71	357	1372	233	793	0	651	0	3	31
	2009	11037	5845	301	2139	1258	750	18	398	967	13	5192	1634	168	70	347	1333	226	747	0	633	0	3	30
	2010	10613	5635	289	2054	1210	725	17	384	944	12	4978	1573	161	67	334	1284	217	695	0	613	0	4	29
	2011	10240	5461	279	1976	1168	705	16	374	931	12	4779	1516	155	65	321	1237	208	647	0	597	0	4	29
	2012	9966	5376	273	1932	1145	695	15	368	938	11	4590	1385	155	66	274	1238	208	624	0	605	0	4	31
	2013	9798	5321	269	1902	1128	686	14	364	948	10	4477	1207	163	69	196	1309	220	634	0	645	0	4	30
	2014	9577	5227	263	1859	1103	671	13	356	952	10	4350	1177	159	68	191	1277	214	595	0	636	0	4	29
	2015	9271	5091	255	1801	1070	652	12	345	946	9	4180	1135	153	65	184	1232	206	553	0	620	0	4	28
Mongolia	2000	3029	1234	72	512	286	131	11	76	140	6	1795	524	41	50	82	426	116	364	17	166	0	0	9
	2001	2790	1172	68	481	270	127	9	72	140	5	1618	469	37	45	73	381	108	310	34	152	0	0	9
	2002	2547	1103	63	446	252	122	8	68	139	4	1444	416	34	41	64	339	101	263	40	138	0	0	8
	2003	2325	1032	58	410	235	116	6	64	137	4	1293	380	31	38	58	308	96	229	15	129	0	0	8
	2004	2158	984	55	383	224	113	6	62	138	3	1174	347	29	35	52	281	92	200	8	121	0	0	8
	2005	2053	962	54	366	219	113	5	61	142	3	1091	308	28	35	42	270	92	184	5	119	0	0	8
	2006	2006	974	54	361	222	116	4	63	151	3	1032	277	28	34	35	262	94	171	4	118	0	0	9
	2007	1994	995	54	360	227	121	4	66	161	3	999	256	28	34	29	259	96	162	3	120	0	0	10
	2008	1997	1018	55	359	232	125	4	68	172	2	979	242	29	35	26	259	100	155	2	122	0	0	10
	2009	1985	1015	55	350	231	127	3	69	179	2	970	240	29	35	25	256	101	148	2	124	0	0	11
	2010	1946	997	53	337	226	126	3	68	182	2	949	235	28	34	24	251	101	139	2	123	0	0	11
	2011	1893	965	51	321	218	123	3	66	182	2	928	229	28	34	23	246	101	131	2	123	0	0	11
	2012	1826	925	49	303	207	119	2	64	179	2	901	223	27	33	22	239	100	122	1	122	0	0	11
	2013	1751	887	46	287	198	115	2	62	176	2	864	214	26	32	20	230	98	113	0	118	0	0	11
	2014	1673	843	44	270	187	109	2	59	170	1	830	206	26	31	19	221	97	105	0	115	0	0	10
	2015	1581	789	41	252	174	103	2	55	162	1	792	196	25	30	18	211	95	97	0	111	0	0	10
Republic of Korea	2000	3275	1235	24	561	114	127	0	66	342	0	2040	164	159	75	50	576	429	30	13	542	0	1	0
	2001	3241	1320	13	713	112	84	0	78	320	0	1921	143	188	90	42	534	394	14	9	506	0	0	0
	2002	3228	1399	17	676	132	104	0	94	375	0	1829	130	159	43	43	484	427	11	0	533	0	0	0
	2003	3218	1419	11	719	150	110	0	85	345	0	1799	105	173	62	34	485	429	11	0	500	0	0	0
	2004	3058	1350	18	637	168	109	0	112	305	0	1708	74	250	34	36	487	384	15	0	427	0	1	0
	2005	2882	1270	9	641	127	120	0	115	258	0	1612	68	194	38	27	531	356	13	0	383	0	2	0
	2006	2665	1122	10	550	106	103	0	102	252	0	1543	77	204	30	35	536	331	16	0	313	0	0	0
	2007	2445	1021	6	491	106	86	0	108	225	0	1424	60	171	37	42	489	337	13	0	276	0	0	0
	2008	2272	966	3	486	91	91	0	126	169	0	1306	58	172	32	28	417	319	5	0	275	0	0	0
	2009	2145	910	5	476	77	65	0	115	171	0	1235	60	173	34	29	387	296	19	0	237	0	0	0
	2010	2019	851	3	438	77	62	0	122	148	0	1168	56	197	37	29	326	298	16	0	207	0	2	0
	2011	1954	839	2	449	69	48	0	101	170	0	1115	55	181	34	37	343	261	14	2	187	0	0	0
	2012	1830	824	1	439	78	53	0	96	156	0	1006	37	133	25	27	334	251	9	0	190	0	0	0
	2013	1748	806	2	425	72	53	0	102	152	0	942	42	146	27	27	289	232	11	0	168	0	0	0
	2014	1670	791	2	417	71	52	0	100	149	0	879	39	136	26	25	269	216	10	0	156	0	0	0
	2015	1559	733	2	387	66	48	0	93	138	0	826	37	128	24	23	253	203	10	0	147	0	0	0
Latin America and the Caribbean	2000	378184	173081	8041	69422	30100	23145	896	15104	26107	267	205103	48028	4409	2509	8357	51480	28410	33608	0	22705	422	3793	1383
	2001	356629	163982	7075	65135	28287	22047	846	14655	25687	251	192647	43776	4185	2457	7418	50069	27091	30192	0	21999	409	3691	1362
	2002	335497	155081	6702	60431	26770	20936	758	14426	24819	238	180416	40316	4190	2375	6532	46136	26220	28050	2	21403	358	3540	1294
	2003	315788	146864	6109	56702	24945	20178	719	13738	24246	229	168924	37459	4054	2267	5988	43818	25066	25644	0	19590	350	3395	1292
	2004	297458	139700	5975	51909	24051	19649	661	13177	24058	219	157758	35262	3824	2264	5695	40002	24743	22356	0	18811	325	3132	1345
	2005	280078	133398	5727	49950	22549	18649	629	12489	23194	211	146680	31235	3849	2173	5113	37224	23674	20844	0	18097	338	2792	1343
	2006	264583	126753	5512	45758	21680	17439	577	12282	23305	200	137830	29753	3842	2171	4919	34121	23204	18162	0	17507	295	2526	1331
	2007	250769	120497	5083	43202	20569	16859	547	11843	22204	190	130272	27747	3879	2088	4429	33527	22444	15832	0	16575	257	2187	1308
	2008	238291	116066	4956	41599	19687	16061	515	11081	21988	178	122225	25525	3728	1965	4127	32109	22042	13713	0	15537	234	1900	1345
	2009	227778	114379	4849	40618	19033	16128	483	11085	22016	166	113399	24320	3524	1811	3708	29598	20406	11982	0	14771	310	1640	1329
	2010	257879	115020	4850	40460	18724	15915	393	11582	22932	163	142859	23017	3545	1921	3715	27962	19806	11160	0	48656	300	1505	1274
	2011	212544	114251	4939	39621	18374	16193	347	11508	23124	146	98293	21187	3519	1687	3133	24764	18668	9398	0	13040	234	1214	1448
	2012	207516	112971	4746	38608	17656	15991	321	12082	23432	137	94545	19828	3454	1637	2874	23913	18476	8809	0	12848	203	956	1547
	2013	203423	110279	4434	37730	17293	15160	306	12288	22937	130	93144	18972	3338	1619	2541	24437	18227	8837	1	12764	187	840	1381
	2014	200050	106528	4385	36543	16744	14866	289	11458	22120	123	93522	18890	3488	1690	2628	24557	18651	8428	0	12853	161	797	1378
	2015	196240	101876	4190	34973	16003	14172	274	10929	21219	117	94364	18918	3554	1731	2629	24943	19096	8238	1	12969	146	789	1351
Antigua and Barbuda	2000	25	15	0	7	4	0	0	1	3	0	10	0	1	1	0	5	2	0	0	0	0	0	0
	2001	24	15	0	8	3	0	0	1	2	0	9	1	1	1	1	4	1	0	0	1	0	0	0
	2002	23	14	0	7	0	0	0	1	5	0	9	1	1	1	1	2	3	0	0	1	0	0	0
	2003	21	13	0	6	1	1	0	0	5	0	8	1	0	1	1	2	2	0	0	1	0	0	0
	2004	20	12	0	5	0	0	0	1	6	0	8	1	0	1	0	2	2	0	0	1	0	0	0
	2005	19	11	0	7	1	0	0	1	2	0	8	1	1	0	0	3	1	0	0	1	0	0	0
	2006	18	11	0	5	1	0	0	2	2	0	7	1	0	0	0	3	1	0	0	1	0	0	0
	2007	17	10	0	4	2	1	0	2	1	0	7	1	0	0	0	3	1	0	0	1	0	0	0
	2008	16	10	0	2	2	1	0	3	2	0	6	1	0	0	0	3	1	0	0	1	0	0	0
	2009	15	9	0	2	3	1	0	2	1	0	6	1	0	0	0	3	1	0	0	1	0	0	0
	2010	15	9	0	3	4	0	0	2	1	0	6	0	0	0	0	3	1	0	0	1	0	0	0
	2011	14	9	0	4	3	0	0	1	1	0	5	0	0	0	0	3	1	0	0	1	0	0	0
	2012	13	8	0	4	2	0	0	0	2	0	5	0	0	0	0	3	1	0	0	1	0	0	0

U5D[1]: total number of under 5 death; NEO[2]: total number of neonatal death; PNE[3]: pneumonia; PRE[4]: preterm; INT[5]: intrapartum related events; SEP[6]: sepsis/meningitis; TET[7]: tetanus; OTH[8]: other disorders; CON[9]: congenital; DIA[10]: diarrea; POS[11]: total number of post neonatal death; MEN[12]: meningitis; MEA[13]: measles; INJ[14]: injury; MAL[15]: malaria; PER[16]: pertussis

			0-28 days									1-59 months												
	Year	U5D[1]	NEO[2]	PNE[3]	PRE[4]	INT[5]	SEP[6]	TET[7]	OTH[8]	CON[9]	DIA[10]	POS[11]	PNE[3]	PRE[4]	INT[5]	MEN[12]	OTH[8]	CON[9]	DIA[10]	MEA[13]	INJ[14]	MAL[15]	AIDS	PER[16]
	2013	13	8	0	4	2	0	0	0	2	0	5	0	0	0	0	3	1	0	0	0	0	0	0
	2014	12	8	0	4	2	0	0	0	2	0	4	0	0	0	0	2	1	0	0	1	0	0	0
	2015	12	7	0	3	2	0	0	0	2	0	5	0	0	0	0	3	1	0	0	1	0	0	0
Argentina	2000	14023	7807	144	3601	786	854	0	651	1770	0	6216	824	295	77	202	1769	1566	296	0	1107	0	55	25
	2001	13841	7781	146	3641	695	860	0	598	1840	0	6060	855	253	50	182	1716	1617	325	0	1007	0	32	23
	2002	13758	7595	143	3491	736	1000	0	466	1760	0	6163	818	305	58	195	1961	1465	357	0	934	0	56	14
	2003	13632	7341	156	3356	648	990	0	473	1718	0	6291	1265	314	31	220	1804	1467	256	0	876	0	35	23
	2004	13367	7113	115	3277	623	853	0	484	1760	0	6254	916	317	40	222	1927	1668	200	0	902	0	27	35
	2005	12904	6965	148	3216	582	816	0	477	1726	0	5939	737	335	49	199	1832	1653	207	0	888	0	22	15
	2006	12389	6660	140	3030	497	716	0	477	1800	0	5729	784	293	45	191	1643	1582	232	0	885	0	38	36
	2007	11854	6379	125	2937	527	707	0	455	1628	0	5475	917	274	40	159	1664	1373	138	0	816	0	21	73
	2008	11422	6001	106	2781	467	648	0	424	1574	0	5421	787	285	49	162	1617	1553	170	0	702	0	10	86
	2009	11017	5715	114	2584	438	587	0	407	1585	0	5302	962	288	54	157	1449	1460	162	0	720	0	24	26
	2010	10681	5470	112	2443	415	614	0	373	1513	0	5211	711	271	108	174	1489	1476	188	0	756	0	10	28
	2011	10424	5273	118	2365	362	558	0	402	1468	0	5151	724	365	43	127	1509	1390	140	0	718	0	20	114
	2012	10224	5179	86	2270	359	529	0	449	1487	0	5045	622	282	21	138	1505	1494	119	0	801	0	6	58
	2013	9973	5096	74	2384	369	445	0	458	1365	0	4877	646	243	48	121	1523	1401	136	0	731	0	13	15
	2014	9770	4923	87	2223	345	485	0	415	1368	0	4847	640	285	36	124	1460	1379	127	0	724	0	12	59
	2015	9513	4797	85	2166	336	472	0	404	1333	0	4716	623	278	35	121	1420	1342	123	0	705	0	12	58
Bahamas	2000	82	44	9	13	9	5	0	2	7	0	38	10	3	1	1	6	7	1	0	8	0	1	0
	2001	79	43	7	15	6	5	0	3	7	0	36	8	2	2	1	8	5	1	0	8	0	1	0
	2002	80	44	7	16	3	7	0	5	6	0	36	7	3	3	1	9	7	0	0	6	0	0	0
	2003	82	45	4	15	7	6	0	5	8	0	37	4	1	5	0	11	8	1	0	6	0	0	0
	2004	85	48	4	14	11	9	0	4	7	0	37	3	2	5	0	10	10	0	0	6	0	0	0
	2005	87	49	5	14	10	10	0	4	6	0	38	4	1	3	0	9	12	0	0	8	0	0	0
	2006	87	50	7	15	7	13	0	4	4	0	37	5	1	1	0	10	12	0	0	7	0	0	0
	2007	87	51	11	15	5	11	0	5	4	0	36	7	0	0	0	12	12	0	0	6	0	0	0
	2008	87	52	13	14	7	7	0	5	5	0	35	9	0	0	0	15	6	0	0	5	0	0	0
	2009	86	51	10	17	7	5	0	5	7	0	35	9	0	0	1	16	3	0	0	5	0	0	0
	2010	85	50	7	21	6	4	0	4	7	0	35	8	0	1	1	16	1	0	0	8	0	1	0
	2011	83	49	3	24	6	3	0	3	9	0	34	7	0	1	1	16	1	0	0	7	0	1	0
	2012	80	46	3	23	6	3	0	4	7	0	34	7	0	1	0	15	1	1	0	8	0	1	0
	2013	76	44	4	21	6	2	0	3	8	0	32	8	0	1	0	15	1	1	0	6	0	0	0
	2014	73	42	3	21	5	3	0	3	7	0	31	7	0	1	0	14	1	0	0	7	0	1	0
	2015	70	40	3	20	5	3	0	3	7	0	30	6	0	1	0	14	1	0	0	7	0	1	0
Barbados	2000	58	30	0	18	8	0	0	2	2	0	28	0	3	3	2	11	3	0	0	3	0	3	0
	2001	59	31	0	16	8	1	0	2	4	0	28	1	4	2	1	9	5	0	0	3	0	3	0
	2002	60	33	0	17	6	2	0	3	4	0	27	2	2	3	1	8	4	0	0	5	0	3	0
	2003	61	34	0	12	9	4	0	3	5	0	27	2	3	3	0	7	5	0	0	6	0	1	0
	2004	61	36	1	13	9	4	0	2	7	0	25	2	2	3	0	8	3	1	0	6	0	1	0
	2005	60	36	1	13	9	5	0	2	7	0	24	2	2	1	0	11	3	1	0	4	0	0	0
	2006	59	36	1	12	8	6	0	3	6	0	23	4	1	0	0	13	3	1	0	2	0	0	0
	2007	56	35	0	10	9	6	0	4	5	0	21	3	1	0	0	12	3	0	0	1	0	0	0
	2008	53	33	0	8	10	4	0	4	7	0	20	2	2	0	0	12	2	0	0	2	0	0	0
	2009	51	31	0	8	8	4	0	3	8	0	20	1	2	1	0	13	3	0	0	0	0	0	0
	2010	49	30	0	8	7	5	0	3	7	0	19	1	1	2	0	13	1	0	0	1	0	0	0
	2011	48	30	0	8	4	7	0	4	7	0	18	1	1	2	0	9	2	0	0	2	0	0	0
	2012	47	29	0	8	4	6	0	5	7	0	18	2	1	2	0	8	2	0	0	3	0	0	0
	2013	46	28	0	8	3	6	0	4	7	0	18	1	2	2	0	8	2	0	0	3	0	0	0
	2014	45	28	0	7	4	6	0	4	7	0	17	1	1	2	0	9	2	0	0	2	0	0	0
	2015	44	27	0	7	4	6	0	4	7	0	17	1	1	2	0	9	2	0	0	2	0	0	0
Belize	2000	191	96	1	46	19	7	0	1	22	0	95	23	5	1	6	14	11	9	0	24	0	3	0
	2001	187	94	1	38	20	11	0	4	20	0	93	17	0	2	6	30	12	8	0	16	0	2	0
	2002	181	91	1	30	26	12	0	7	15	0	90	27	2	0	9	27	10	3	0	13	0	0	0
	2003	175	87	5	38	10	12	0	4	19	0	88	21	0	0	2	21	3	12	0	19	0	11	0
	2004	169	84	3	27	22	8	0	8	18	0	85	21	1	0	5	16	12	10	0	13	0	6	0
	2005	163	82	3	41	17	5	0	3	12	0	81	11	2	0	0	20	13	12	0	16	0	6	0
	2006	158	79	5	23	16	7	0	11	16	0	79	21	0	0	1	15	8	10	0	19	0	4	0
	2007	152	76	7	21	16	9	0	10	13	0	76	4	3	0	3	21	19	11	0	11	0	4	0
	2008	148	74	6	22	14	9	0	9	15	0	74	6	0	0	0	40	7	9	0	13	0	0	0
	2009	144	72	4	23	12	8	0	8	15	0	72	7	0	0	0	37	7	5	0	14	0	2	0
	2010	141	71	3	24	11	8	0	7	17	0	70	9	1	0	2	25	14	6	0	11	0	2	0
	2011	140	71	3	26	10	8	0	7	18	0	69	2	0	0	0	27	21	9	0	6	0	4	0
	2012	139	71	2	23	17	12	0	6	12	0	68	19	0	2	2	20	13	4	0	9	0	0	0
	2013	140	71	3	20	5	12	0	12	18	0	69	7	3	0	4	24	19	1	0	11	0	1	0
	2014	139	71	2	23	11	11	0	8	16	0	68	9	1	1	2	23	17	4	0	9	0	2	0
	2015	138	70	2	23	10	10	0	8	16	0	68	9	1	1	2	23	17	4	0	9	0	2	0
Bolivia (Plurinational State of)	2000	20137	7571	518	2296	2273	1062	132	578	651	60	12566	3226	387	380	397	2494	1141	3049	0	1164	6	29	292
	2001	19162	7335	496	2231	2181	1035	116	561	662	54	11827	3042	371	362	376	2359	1113	2751	0	1121	3	33	296
	2002	18132	7163	479	2193	2107	1005	105	546	678	49	10969	2828	349	339	351	2203	1059	2446	0	1064	2	37	291
	2003	17148	7035	465	2175	2042	977	98	534	697	46	10113	2595	327	316	318	2051	1006	2168	0	1005	3	41	283
	2004	16167	6909	451	2150	1987	941	91	525	720	44	9258	2358	304	293	284	1874	950	1909	0	944	2	45	295
	2005	15285	6767	437	2116	1928	907	84	513	741	40	8518	2170	283	271	258	1726	889	1688	0	889	3	48	294
	2006	14441	6556	418	2055	1851	871	75	497	753	36	7885	2022	266	250	240	1588	834	1496	0	839	4	49	298
	2007	13637	6305	397	1980	1758	839	66	476	759	32	7332	1879	251	233	220	1478	789	1336	0	798	3	50	296
	2008	12918	6085	378	1913	1672	817	58	456	763	29	6833	1767	243	221	203	1411	762	1105	0	774	2	51	293
	2009	12244	5836	358	1841	1578	790	51	433	759	26	6408	1654	234	209	185	1345	736	958	0	749	2	50	288
	2010	11661	5632	341	1784	1500	767	45	414	757	23	6029	1572	223	196	176	1263	695	850	0	718	2	43	291
	2011	11107	5437	326	1730	1429	739	40	397	755	21	5670	1467	214	184	161	1190	667	768	0	689	1	39	290
	2012	10616	5237	310	1669	1363	708	36	383	749	20	5379	1400	206	174	154	1113	639	702	0	661	1	31	299
	2013	10130	5092	299	1626	1312	682	34	370	750	18	5038	1310	194	164	142	1053	607	632	0	628	2	28	280
	2014	9743	4935	287	1582	1260	655	31	357	746	17	4808	1082	197	167	96	1092	622	611	0	644	1	27	268
	2015	9415	4823	279	1554	1220	633	29	346	746	16	4592	1033	189	160	92	1050	601	563	0	622	1	26	256
Brazil	2000	121560	61046	2151	25968	9280	8991	20	6382	8255	0	60514	12778	1398	798	3074	18018	9053	9208	0	5465	88	564	70
	2001	112275	56608	1595	23815	8872	8187	12	6002	8125	0	55667	11050	1295	783	2655	18002	8157	8152	0	4991	48	509	24
	2002	102800	52117	1455	20935	8359	7666	3	6098	7601	0	50683	9597	1255	808	2075	15866	8234	7483	0	4919	31	385	28
	2003	93417	47688	1197	19135	7134	7312	5	5604	7301	0	45729	8749	1113	734	1733	14707	7668	6249	0	4368	34	332	42
	2004	84492	43470	1084	16190	6997	6753	3	5380	7064	0	41022	7988	1004	722	1625	12616	7217	5335	0	4201	33	240	41
	2005	75911	39575	923	14766	6195	6222	2	5121	6345	0	36336	6625	936	649	1456	11128	6627	4783	0	3849	41	199	42
	2006	68023	35652	766	12265	5954	5631	2	4953	6082	0	32371	6014	997	665	1374	9413	6313	3976	0	3354	34	199	34
	2007	61178	32709	713	11024	5371	5248	0	4689	5664	0	28469	5050	987	670	1121	8445	5977	2699	0	3312	30	147	31
	2008	55055	30871	701	10538	4983	4737	2	4272	5639	0	24184	4123	882	532	966	7255	5267	2171	0	2800	21	144	21
	2009	50456	30328	612	10142	4908	4785	2	4319	5559	0	20128	3654	701	428	791	6009	4455	1585	0	2380	26	82	16
	2010	47835	30378	594	9976	4866	4310	0	4808	5824	0	17457	2821	713	418	674	5321	4043	1190	0	2139	22	94	21
	2011	46876	30977	642	10013	4754	4514	5	4990	6059	0	15899	2825	644	391	458	4779	3883	839	0	1960	20	57	44
	2012	47449	31639	661	9802	4782	4555	0	5594	6246	0	15810	2741	681	319	409	4614	3975	889	0	1991	18	79	94
	2013	49094	31645	642	9582	4789	4428	4	5947	6253	0	17449	3065	687	358	388	5117	4424	993	1	2221	11	81	104
	2014	51117	30421	628	9489	4623	4357	3	5332	5989	0	20696	3635	849	451	531	6114	5171	1145	0	2599	9	91	101
	2015	52415	28604	590	8922	4347	4096	3	5014	5631	0	23811	4183	977	519	611	7035	5950	1318	1	2991	7	105	117
Chile	2000	2789	1452	37	587	118	94	0	60	556	0	1337	239	77	8	52	284	379	25	0	258	0	4	11
	2001	2620	1394	32	601	112	68	0	85	495	0	1226	260	68	5	70	263	361	14	0	172	0	6	7
	2002	2473	1334	37	517	105	65	0	89	521	0	1139	126	64	8	50	310	397	19	0	151	0	6	8
	2003	2377	1322	28	483	94	68	0	113	535	0	1055	124	49	6	52	273	355	25	0	156	0	4	11
	2004	2306	1336	27	504	121	58	0	122	503	0	970	120	51	2	43	267	344	12	0	118	0	1	11
	2005	2239	1326	38	551	99	69	0	83	486	0	913	104	49	6	47	219	341	9	0	127	0	2	10
	2006	2200	1342	29	538	112	65	0	97	501	0	858	77	61	7	29	252	287	3	0	130	0	5	6
	2007	2162	1333	24	549	91	84	0	108	477	0	829	94	69	9	23	212	297	6	0	102	0	2	14

U5D[1]: total number of under 5 death; NEO[2]: total number of neonatal death; PNE[3]: pneumonia; PRE[4]: preterm; INT[5]: intrapartum related events; SEP[6]: sepsis/meningitis; TET[7]: tetanus; OTH[8]: other disorders; CON[9]: congenital; DIA[10]: diarrea; POS[11]: total number of post neonatal death; MEN[12]: meningitis; MEA[13]: measles; INJ[14]: injury; MAL[15]: malaria; PER[16]: pertussis

			0-28 days									1-59 months												
	Year	U5D[1]	NEO[2]	PNE[3]	PRE[4]	INT[5]	SEP[6]	TET[7]	OTH[8]	CON[9]	DIA[10]	POS[11]	PNE[3]	PRE[4]	INT[5]	MEN[12]	OTH[8]	CON[9]	DIA[10]	MEA[13]	INJ[14]	MAL[15]	AIDS	PER[16]
	2008	2150	1326	18	565	77	68	0	120	478	0	824	105	48	8	25	206	279	10	0	132	0	4	8
	2009	2114	1319	22	543	70	71	0	104	510	0	795	101	44	7	19	192	290	6	0	126	0	2	6
	2010	2104	1289	12	546	81	67	0	112	470	0	815	76	51	8	32	196	298	4	0	135	0	7	7
	2011	2070	1282	28	527	83	71	0	83	488	0	788	48	47	9	19	203	341	1	0	105	0	1	13
	2012	2036	1252	11	499	83	60	0	113	486	0	784	49	46	4	23	203	320	0	0	124	0	1	14
	2013	1979	1223	18	487	62	39	0	107	510	0	756	57	37	16	11	217	280	3	0	131	0	0	5
	2014	1948	1195	18	481	72	54	0	97	472	0	753	50	42	9	17	202	304	1	0	117	0	1	10
	2015	1896	1144	17	461	69	52	0	93	452	0	752	50	42	9	17	201	304	1	0	116	0	1	10
**Colombia**	2000	20708	11036	481	4820	1719	1124	2	1017	1874	0	9672	2151	173	92	378	2895	1281	1262	0	1268	99	42	31
	2001	20045	10742	393	4449	1451	1424	3	1083	1940	0	9303	1870	154	54	352	3194	1321	839	0	1285	138	45	52
	2002	19658	10548	462	4431	1407	1064	6	1104	2073	0	9110	1476	189	63	354	2831	1415	1157	0	1436	118	44	27
	2003	19340	10386	408	4119	1521	1039	6	1114	2180	0	8954	1590	169	58	345	2743	1396	1213	0	1260	99	68	12
	2004	18941	10255	437	3519	1406	1532	1	1117	2242	0	8686	1611	141	56	319	2702	1481	988	0	1242	69	68	10
	2005	18364	10016	406	3754	1269	1435	3	1044	2106	0	8348	1567	200	33	353	2627	1367	831	0	1226	54	59	31
	2006	17686	9644	442	3452	1173	1245	3	1078	2252	0	8042	1637	165	56	335	2397	1403	706	0	1224	58	46	14
	2007	16853	9105	367	3005	1184	1301	0	1118	2130	0	7748	1518	166	63	245	2338	1398	722	0	1173	64	52	9
	2008	15943	8614	308	3087	1160	1020	1	1004	2033	0	7329	1309	206	98	227	2259	1592	489	0	1079	29	26	16
	2009	15070	8146	328	2871	999	1007	0	1009	1932	0	6924	1230	230	95	226	2234	1370	477	0	979	28	37	17
	2010	14307	7667	256	2676	730	857	2	1032	2115	0	6640	1116	323	86	184	2160	1434	347	0	912	46	30	2
	2011	13743	7346	272	2619	733	1028	0	742	1953	0	6397	1148	348	115	165	1965	1425	274	0	882	20	28	26
	2012	13190	7076	303	2370	564	1111	0	760	1968	0	6114	1083	345	149	124	1944	1338	241	0	775	21	23	72
	2013	12772	6837	258	2371	626	930	0	782	1869	0	5935	1039	316	109	147	1883	1302	267	0	796	21	25	31
	2014	12364	6603	249	2290	605	898	0	755	1805	0	5761	1009	307	106	142	1828	1265	259	0	773	18	24	31
	2015	11934	6356	240	2204	582	865	0	727	1738	0	5578	977	297	103	138	1771	1225	251	0	749	16	23	30
**Costa Rica**	2000	1022	603	19	227	71	42	0	34	210	0	419	55	24	5	9	132	114	36	0	41	0	2	1
	2001	938	566	23	220	58	49	0	32	184	0	372	45	16	4	7	102	113	26	0	52	0	2	4
	2002	866	533	21	244	43	30	0	34	160	0	333	39	16	1	14	94	107	15	0	45	0	0	1
	2003	795	508	23	217	51	35	0	30	151	0	287	33	8	3	5	64	103	22	0	49	0	0	1
	2004	754	488	16	202	45	33	0	30	163	0	266	32	6	2	3	95	70	17	0	40	0	0	1
	2005	724	483	12	185	52	29	0	30	176	0	241	40	10	6	7	53	76	15	0	34	0	1	1
	2006	721	481	18	178	66	47	0	29	142	0	240	22	11	4	6	84	79	9	0	20	0	0	6
	2007	736	486	12	202	47	40	0	20	165	0	250	30	15	1	6	54	91	13	0	34	0	1	5
	2008	757	494	14	188	58	40	0	41	152	0	263	19	14	3	9	77	96	8	0	30	0	0	5
	2009	766	498	6	183	64	13	0	25	206	0	268	19	9	4	7	84	104	11	0	29	0	0	1
	2010	772	503	11	188	61	13	0	23	207	0	269	23	14	3	7	82	100	9	0	29	0	0	2
	2011	760	491	15	187	57	12	0	19	200	0	269	22	15	2	7	83	102	8	0	28	0	0	2
	2012	745	480	19	187	53	11	0	16	194	0	265	15	13	4	8	83	105	12	0	25	0	0	0
	2013	717	458	10	197	54	11	0	18	167	0	259	28	20	1	5	82	93	3	0	26	0	0	0
	2014	690	437	14	175	50	11	0	16	172	0	253	21	16	2	7	79	96	7	0	25	0	0	0
	2015	663	421	13	169	48	10	0	16	165	0	242	20	15	2	6	76	92	7	0	24	0	0	0
**Cuba**	2000	1292	650	20	198	137	85	0	42	168	0	642	61	15	10	47	202	187	19	0	101	0	0	0
	2001	1220	627	52	155	123	69	0	64	164	0	593	61	20	6	35	176	174	20	0	101	0	0	0
	2002	1143	593	62	138	104	70	0	62	158	0	550	52	13	8	18	163	178	13	0	102	0	2	0
	2003	1055	538	54	137	86	66	0	54	143	0	517	48	20	13	27	167	132	16	0	93	0	1	0
	2004	970	485	51	111	96	62	0	40	124	0	485	42	10	11	21	163	148	12	0	77	0	2	0
	2005	896	440	34	108	53	62	0	62	120	0	456	54	11	7	26	149	128	19	0	62	0	0	0
	2006	845	402	32	116	59	50	0	45	100	0	443	57	22	9	21	142	106	14	0	72	0	0	0
	2007	801	381	39	97	42	70	0	49	84	0	420	47	19	9	16	140	115	11	0	60	0	1	0
	2008	777	351	34	101	47	51	0	49	70	0	426	39	17	18	21	148	99	16	0	68	0	1	0
	2009	756	336	41	97	50	47	0	23	78	0	420	66	15	17	13	146	99	11	0	54	0	0	0
	2010	736	322	28	95	52	48	0	37	61	0	414	47	16	16	14	145	112	12	0	52	0	0	0
	2011	718	309	26	92	47	44	0	37	63	0	409	50	15	17	20	147	106	8	0	45	0	0	0
	2012	693	297	23	98	37	24	0	30	85	0	396	56	35	12	22	114	88	9	0	59	0	0	0
	2013	680	285	21	93	43	34	0	41	53	0	395	56	21	7	15	125	97	10	0	62	0	1	0
	2014	665	284	23	90	40	32	0	34	64	0	381	51	23	12	18	123	92	9	0	53	0	0	0
	2015	647	270	22	86	38	31	0	33	61	0	377	51	23	12	18	121	91	8	0	53	0	0	0
**Dominica**	2000	16	12	0	7	1	1	0	1	2	0	4	0	0	0	0	1	1	0	0	0	0	0	0
	2001	15	10	0	7	1	0	0	0	1	0	5	0	0	0	0	2	1	0	0	0	0	0	0
	2002	14	10	0	7	1	0	0	1	1	0	4	0	0	0	0	2	1	0	0	0	0	0	0
	2003	14	10	0	7	2	0	0	1	0	0	4	0	0	0	0	3	1	0	0	0	0	0	0
	2004	14	10	0	5	2	1	0	1	2	0	4	0	0	0	0	3	0	0	0	0	0	0	0
	2005	15	11	0	6	1	1	0	1	2	0	4	1	0	0	0	2	1	0	0	0	0	0	0
	2006	15	11	1	6	0	2	0	1	2	0	4	1	0	0	0	2	1	0	0	0	0	0	0
	2007	16	12	1	6	2	1	0	0	2	0	4	1	0	0	0	2	1	0	0	0	0	0	0
	2008	17	13	1	4	2	4	0	0	2	0	4	1	0	0	0	2	1	0	0	0	0	0	0
	2009	18	14	0	3	6	4	0	0	1	0	4	1	0	0	0	2	1	0	0	0	0	0	0
	2010	20	15	0	2	4	6	0	1	2	0	5	0	0	0	0	2	2	0	0	1	0	0	0
	2011	21	16	0	3	6	4	0	1	2	0	5	0	0	0	0	2	2	0	0	1	0	0	0
	2012	22	16	0	3	4	4	0	1	3	0	6	1	0	0	0	2	1	0	0	1	0	0	0
	2013	23	17	0	4	5	4	0	1	3	0	6	1	0	0	0	2	1	0	0	1	0	0	0
	2014	23	17	0	3	3	5	0	2	4	0	6	1	0	0	0	2	1	1	0	1	0	0	0
	2015	23	17	0	3	4	4	0	2	4	0	6	1	0	0	0	2	1	0	0	1	0	0	0
**Dominican Republic**	2000	8580	4968	281	2026	1072	499	41	295	727	28	3612	885	139	43	112	690	312	554	0	320	1	514	41
	2001	8390	5003	282	2019	1080	510	40	297	747	27	3387	791	134	41	96	651	305	507	0	305	1	516	41
	2002	8313	5082	288	1949	1119	572	36	318	773	25	3231	719	132	40	82	629	306	475	0	298	1	508	42
	2003	8307	5178	292	1906	1151	638	32	342	793	24	3129	651	133	40	67	626	316	459	0	299	1	493	43
	2004	8276	5257	294	1885	1172	682	30	359	811	23	3019	625	131	39	63	610	314	434	0	293	2	465	43
	2005	8206	5341	298	1924	1188	681	31	361	835	23	2865	587	127	38	57	583	307	404	0	283	3	435	43
	2006	8076	5347	297	1917	1188	683	30	365	844	23	2729	572	121	37	56	555	299	374	0	271	3	399	43
	2007	7908	5337	295	1905	1185	684	29	367	848	23	2571	557	115	35	55	521	288	342	0	257	2	354	44
	2008	7707	5257	289	1867	1163	683	28	365	840	22	2450	531	113	36	50	510	288	325	0	253	1	300	43
	2009	7504	5178	284	1838	1138	680	27	358	832	21	2326	515	109	35	49	491	284	304	0	245	1	249	43
	2010	7348	5095	277	1805	1113	681	25	352	821	21	2253	507	108	36	47	491	283	294	0	245	2	199	41
	2011	7221	5040	273	1780	1096	683	24	350	815	20	2181	514	107	36	49	482	285	281	0	242	1	141	42
	2012	7094	4980	269	1756	1079	678	23	346	811	19	2114	513	107	37	49	480	288	267	0	242	1	89	42
	2013	7000	4905	264	1731	1060	665	22	341	804	19	2095	514	109	37	48	489	298	235	0	247	0	74	42
	2014	6854	4801	257	1697	1033	647	21	333	795	18	2053	463	113	39	38	504	311	226	0	255	1	63	41
	2015	6710	4706	251	1667	1009	631	20	325	786	17	2004	452	111	38	37	492	309	214	0	250	0	62	40
**Ecuador**	2000	10853	5451	270	2338	844	656	114	395	834	0	5402	1303	31	22	167	1407	771	910	0	636	0	99	57
	2001	10416	5242	252	2211	803	691	69	395	822	0	5174	1239	32	23	158	1352	766	825	0	628	0	96	56
	2002	9972	5014	235	2081	767	667	69	390	806	0	4958	1172	33	23	150	1298	759	752	0	621	0	93	58
	2003	9585	4817	223	1973	741	637	69	381	794	0	4768	1035	41	29	175	1372	752	644	0	574	0	89	58
	2004	9265	4640	211	1874	719	609	72	374	781	0	4625	980	49	34	171	1332	772	577	0	568	0	83	59
	2005	9003	4496	202	1790	702	596	63	370	774	0	4507	951	52	36	166	1291	784	515	0	575	0	79	57
	2006	8829	4420	197	1741	697	587	53	367	777	0	4409	927	56	38	162	1250	796	462	0	581	0	80	57
	2007	8706	4369	197	1716	690	567	55	356	787	0	4337	907	59	39	158	1222	807	427	0	588	0	73	56
	2008	8634	4325	200	1709	680	542	53	339	802	0	4309	900	63	41	155	1216	825	411	0	601	0	42	55
	2009	8508	4268	205	1705	662	511	53	317	815	0	4240	880	66	42	151	1197	833	397	0	604	0	16	55
	2010	8332	4181	208	1693	631	484	51	294	820	0	4151	851	68	43	144	1169	833	379	0	599	0	8	55
	2011	8139	4062	208	1662	597	455	50	274	816	0	4077	825	70	44	139	1145	834	363	0	596	0	5	55
	2012	7868	3920	203	1612	560	433	49	259	804	0	3948	786	72	44	131	1107	824	342	0	582	0	3	56
	2013	7602	3765	197	1548	525	413	47	249	786	0	3837	753	73	45	124	1077	814	325	0	568	0	3	54
	2014	7333	3644	190	1493	502	397	45	245	772	0	3689	714	74	46	116	1036	794	307	0	547	0	3	52
	2015	7079	3530	183	1438	480	382	44	243	760	0	3549	678	72	45	109	997	776	291	0	528	0	3	50
**El Salvador**	2000	4590	2009	97	779	357	250	23	181	322	0	2581	738	24	10	75	583	396	423	0	255	0	50	27
	2001	4190	1832	90	705	319	226	21	165	306	0	2358	644	23	9	66	537	369	394	0	238	0	51	26
	2002	3830	1673	85	641	287	198	18	150	294	0	2157	524	31	12	73	548	353	338	0	207	0	46	25

U5D[1]: total number of under 5 death; NEO[2]: total number of neonatal death; PNE[3]: pneumonia; PRE[4]: preterm; INT[5]: intrapartum related events; SEP[6]: sepsis/meningitis; TET[7]: tetanus; OTH[8]: other disorders; CON[9]: congenital; DIA[10]: diarrea; POS[11]: total number of post neonatal death; MEN[12]: meningitis; MEA[13]: measles; INJ[14]: injury; MAL[15]: malaria; PER[16]: pertussis

		0-28 days										1-59 months												
	Year	U5D[1]	NEO[2]	PNE[3]	PRE[4]	INT[5]	SEP[6]	TET[7]	OTH[8]	CON[9]	DIA[10]	POS[11]	PNE[3]	PRE[4]	INT[5]	MEN[12]	OTH[8]	CON[9]	DIA[10]	MEA[13]	INJ[14]	MAL[15]	AIDS	PER[16]
	2003	3501	1536	81	589	260	172	15	136	282	0	1965	465	33	13	66	500	337	296	0	195	0	39	22
	2004	3216	1426	78	547	238	152	13	125	273	0	1790	418	33	12	59	452	320	256	0	185	0	32	22
	2005	2977	1331	74	508	218	136	13	117	264	0	1646	382	34	13	54	412	309	218	0	177	0	27	21
	2006	2777	1265	70	481	204	126	13	113	258	0	1512	346	36	13	49	372	297	183	0	166	0	29	20
	2007	2633	1223	67	462	194	119	15	112	254	0	1410	317	37	13	45	342	286	160	0	156	0	32	21
	2008	2505	1187	64	446	185	115	15	112	251	0	1318	289	37	13	41	316	272	148	0	146	0	34	22
	2009	2402	1151	61	431	177	109	15	110	248	0	1251	267	36	13	37	298	257	147	0	138	0	35	24
	2010	2291	1122	59	419	170	104	14	109	246	0	1169	242	35	12	33	275	241	141	0	128	0	37	25
	2011	2179	1071	56	400	161	96	14	105	239	0	1108	224	36	12	31	258	232	132	0	122	0	37	25
	2012	2059	1016	53	379	151	89	13	101	230	0	1043	209	37	13	28	241	225	121	0	116	0	29	25
	2013	1949	961	49	358	142	81	12	97	221	0	988	196	38	13	26	227	217	111	0	111	0	25	24
	2014	1848	910	46	338	134	76	12	93	212	0	938	184	39	13	24	215	210	102	0	105	0	24	22
	2015	1763	868	42	322	127	71	11	90	205	0	895	173	39	13	23	204	203	96	0	100	0	23	21
Grenada	2000	35	17	0	8	5	4	0	0	0	0	18	3	1	3	0	5	1	1	0	3	0	0	0
	2001	34	16	0	9	4	3	0	0	0	0	18	2	2	5	0	4	1	1	0	3	0	0	0
	2002	31	16	0	9	4	3	0	0	1	0	15	2	2	3	0	4	1	1	0	3	0	0	0
	2003	29	15	0	7	5	2	0	1	1	0	14	1	3	3	0	2	3	0	0	3	0	0	0
	2004	27	14	0	4	6	1	0	1	1	0	13	1	2	1	0	2	3	0	0	2	0	0	0
	2005	26	13	0	4	5	1	0	2	2	0	13	2	2	1	0	1	4	0	0	3	0	0	0
	2006	26	13	0	4	5	2	0	1	1	0	13	3	1	1	0	2	2	0	0	2	0	0	0
	2007	26	14	0	5	4	1	0	2	2	0	12	2	1	0	0	5	1	0	0	2	0	0	0
	2008	27	14	0	3	3	2	0	2	3	0	13	2	0	0	0	7	3	0	0	1	0	0	0
	2009	28	14	0	3	3	2	0	3	3	0	14	0	0	0	0	9	3	0	0	1	0	0	0
	2010	28	15	1	4	3	2	0	2	3	0	13	0	0	0	0	6	5	0	0	2	0	0	0
	2011	28	14	1	4	4	1	0	2	2	0	14	0	0	0	0	6	4	1	0	2	0	0	0
	2012	27	14	1	5	3	1	0	2	1	0	13	1	0	0	0	5	4	1	0	2	0	0	0
	2013	26	13	1	5	3	1	0	2	1	0	13	1	0	0	0	5	4	1	0	2	0	0	0
	2014	25	13	1	6	3	1	0	2	1	0	12	1	0	0	0	5	4	0	0	2	0	0	0
	2015	24	12	1	5	3	1	0	2	1	0	12	1	0	0	0	4	4	1	0	2	0	0	0
Guatemala	2000	20153	8362	587	2258	2377	1377	82	627	1002	53	11791	3883	428	134	521	2311	529	2483	0	1321	10	92	78
	2001	19476	8124	564	2179	2293	1354	73	611	1001	49	11352	3745	415	132	503	2238	523	2305	0	1291	7	113	81
	2002	18813	7898	542	2115	2211	1323	65	594	1001	46	10915	3602	402	129	485	2168	516	2134	0	1258	7	134	80
	2003	18228	7755	527	2074	2155	1304	60	583	1009	43	10473	3453	387	126	465	2088	507	1970	0	1220	6	168	82
	2004	17646	7557	508	2022	2085	1271	54	568	1008	41	10089	3329	376	124	448	2026	502	1830	0	1190	5	177	81
	2005	17017	7337	490	1964	2015	1222	50	552	1007	39	9680	2713	402	135	291	2173	545	1876	0	1281	7	173	84
	2006	16401	7168	474	1924	1958	1182	46	539	1008	37	9233	2588	389	130	274	2097	535	1737	0	1243	5	149	86
	2007	15750	6943	456	1868	1887	1132	43	522	1001	35	8807	2495	373	126	267	2004	520	1594	0	1196	2	142	88
	2008	15099	6760	438	1812	1819	1116	39	509	994	33	8339	2310	359	121	237	1930	507	1471	0	1157	1	161	85
	2009	14615	6601	423	1749	1762	1111	34	503	988	31	8014	2239	347	117	233	1864	496	1364	0	1121	1	148	85
	2010	14197	6476	408	1683	1712	1127	29	504	986	28	7721	2169	340	116	223	1829	491	1194	0	1104	1	167	86
	2011	13849	6353	395	1629	1671	1114	25	505	987	26	7496	2167	331	113	231	1782	484	1048	0	1081	1	169	89
	2012	13558	6255	385	1598	1635	1093	23	503	992	25	7303	2063	329	113	209	1774	486	979	0	1078	1	184	87
	2013	13370	6162	377	1584	1605	1057	22	497	996	24	7208	1971	333	114	197	1796	496	951	0	1092	1	172	86
	2014	13147	6049	368	1567	1565	1025	21	487	994	23	7098	1847	333	114	180	1799	501	965	0	1094	1	178	85
	2015	12858	5946	359	1551	1528	996	20	477	992	22	6912	1798	326	112	175	1764	496	909	0	1074	1	174	83
Guyana	2000	882	483	12	227	99	71	0	27	46	0	399	62	2	3	6	88	39	108	0	41	28	21	0
	2001	845	464	18	193	114	57	0	32	50	0	381	45	2	6	11	84	33	116	0	23	30	32	0
	2002	806	443	13	212	74	79	0	23	42	0	363	60	4	9	7	69	40	82	0	28	23	41	0
	2003	761	419	2	215	85	61	0	20	37	0	342	43	0	0	8	64	53	71	0	38	30	35	0
	2004	716	394	5	170	78	74	0	34	33	0	322	49	4	7	9	59	50	34	0	48	33	31	0
	2005	668	368	12	165	73	69	0	14	35	0	300	40	0	2	9	59	25	60	0	23	55	27	0
	2006	616	337	10	154	64	39	0	27	43	0	279	39	0	0	5	43	34	68	0	50	25	15	0
	2007	563	306	6	132	69	36	2	36	25	0	257	34	7	2	7	59	43	34	0	42	9	21	0
	2008	517	280	8	99	62	34	0	50	28	0	237	28	0	0	7	93	24	35	0	30	11	9	0
	2009	486	263	8	94	43	31	0	43	43	0	223	30	0	0	13	60	21	39	0	40	16	4	0
	2010	478	261	4	83	67	38	0	31	38	0	217	27	0	3	3	55	23	33	0	34	37	2	0
	2011	492	278	11	83	62	51	0	47	25	0	214	28	0	0	8	58	20	18	0	32	45	5	0
	2012	532	310	9	100	66	47	0	47	41	0	222	27	0	1	8	55	20	28	0	33	46	3	0
	2013	582	346	11	112	73	53	0	52	46	0	236	30	0	1	8	62	23	32	0	37	39	4	0
	2014	623	376	11	121	80	57	0	57	50	0	247	31	0	1	9	64	24	33	0	39	42	4	0
	2015	642	391	12	126	83	59	0	59	52	0	251	32	0	1	9	66	24	34	0	40	40	4	0
Haiti	2000	27905	8146	605	2735	2274	1131	156	640	479	126	19759	5934	466	392	1134	2983	806	4682	0	1525	143	1338	356
	2001	26914	8028	592	2710	2229	1114	149	629	484	121	18886	5693	450	381	1092	2864	793	4326	0	1486	146	1304	352
	2002	25938	7951	581	2706	2184	1110	144	620	489	118	17987	5445	433	368	1050	2745	775	3989	0	1442	149	1248	344
	2003	25004	7902	572	2721	2138	1114	139	611	493	115	17102	5200	416	355	1008	2623	757	3674	0	1396	150	1185	338
	2004	24196	7845	559	2751	2083	1113	134	600	493	112	16351	4995	401	345	972	2525	741	3408	0	1358	157	1116	333
	2005	23553	7765	546	2777	2023	1106	127	585	492	108	15788	4869	393	339	951	2468	734	3209	0	1338	153	1006	328
	2006	23006	7693	535	2776	1979	1112	121	573	493	104	15313	4785	388	337	936	2429	737	3048	0	1330	148	846	328
	2007	22564	7651	529	2720	1965	1151	114	569	502	100	14913	4725	385	335	926	2401	740	2909	0	1327	127	710	328
	2008	22146	7590	523	2617	1956	1209	105	573	512	95	14556	4645	378	333	911	2365	737	2764	0	1319	154	622	327
	2009	21710	7537	516	2505	1949	1281	92	583	523	89	14173	4542	370	329	892	2316	730	2612	0	1303	217	537	327
	2010	59719	8161	556	2649	2108	1450	88	641	578	91	51558	5483	389	457	1078	2869	889	3049	0	36325	164	523	331
	2011	20630	7301	491	2338	1879	1333	72	581	529	78	13329	4370	356	322	857	2228	719	2347	0	1279	124	395	331
	2012	19947	7125	475	2270	1825	1319	66	569	528	73	12822	4284	349	319	837	2187	713	2223	0	1267	90	220	333
	2013	19191	6935	458	2207	1768	1292	60	555	526	69	12256	3678	370	339	609	2351	763	2267	0	1353	70	136	321
	2014	18465	6733	442	2147	1708	1255	55	538	523	65	11732	3720	353	323	651	2241	734	1924	0	1300	52	126	307
	2015	17841	6537	426	2096	1648	1213	52	521	519	61	11304	3597	343	314	628	2177	718	1797	0	1271	40	121	296
Honduras	2000	7347	3443	157	1361	578	430	29	413	474	0	3904	998	44	26	113	956	482	732	0	174	9	336	34
	2001	6961	3345	147	1302	549	432	28	415	473	0	3616	908	44	26	103	891	463	668	0	167	6	307	33
	2002	6569	3244	136	1239	518	438	26	416	470	0	3325	820	43	25	93	824	440	607	0	160	4	276	33
	2003	6199	3105	124	1167	484	438	26	405	461	0	3094	751	42	25	85	769	424	552	0	155	3	254	33
	2004	5858	2989	116	1110	456	437	25	389	456	0	2869	693	43	25	79	717	412	496	0	151	4	215	34
	2005	5550	2880	110	1061	431	427	28	371	452	0	2670	644	45	26	74	675	406	446	0	149	4	169	33
	2006	5268	2778	107	1023	413	413	23	348	452	0	2490	593	44	25	68	629	392	407	0	146	3	148	35
	2007	5003	2666	104	983	393	389	23	325	450	0	2337	550	44	25	63	591	382	375	0	144	3	125	35
	2008	4765	2557	101	941	373	366	23	308	446	0	2208	513	45	25	59	558	373	349	0	142	2	107	35
	2009	4524	2428	96	888	349	343	23	293	436	0	2096	481	47	26	56	527	367	325	0	141	3	88	34
	2010	4294	2294	89	830	325	325	23	285	418	0	2000	451	46	26	52	503	356	315	0	138	3	74	36
	2011	4070	2170	81	772	300	312	22	282	401	0	1900	417	45	25	47	481	339	309	0	132	2	65	37
	2012	3839	2059	74	722	279	300	21	278	384	0	1780	378	43	24	43	450	318	300	0	123	2	61	39
	2013	3633	1953	68	676	261	287	20	274	367	0	1680	348	42	24	39	425	302	287	0	116	2	58	37
	2014	3467	1861	64	637	245	273	19	268	354	0	1606	328	42	24	36	407	292	274	0	111	2	55	35
	2015	3333	1787	60	608	233	260	18	263	345	0	1546	312	43	24	34	390	286	261	0	107	1	53	34
Jamaica	2000	1269	979	31	361	137	133	4	120	195	0	290	37	3	3	5	33	32	17	0	26	0	123	10
	2001	1196	917	28	333	125	129	4	117	182	0	279	35	3	3	5	32	31	15	0	26	0	122	9
	2002	1120	849	27	312	116	114	3	104	173	0	271	33	3	3	4	31	31	13	0	25	0	119	9
	2003	1043	781	27	290	107	99	3	92	163	0	262	30	3	3	5	32	30	12	0	24	0	115	8
	2004	980	727	26	274	100	85	3	83	155	0	253	28	4	4	5	31	31	11	0	23	0	109	8
	2005	935	685	25	261	94	75	3	78	149	0	250	30	4	4	6	35	35	12	0	27	0	89	8
	2006	909	668	25	254	91	71	3	79	146	0	241	32	4	4	6	39	38	13	0	29	0	66	8
	2007	894	661	24	251	89	69	3	79	145	0	233	35	5	5	6	44	41	15	0	33	0	42	8
	2008	886	655	24	249	87	68	3	79	144	0	231	38	5	5	7	49	45	17	0	36	0	20	9
	2009	870	645	25	245	85	67	3	78	143	0	225	35	5	5	6	45	43	16	0	34	0	28	9
	2010	843	632	23	240	82	65	3	77	142	0	211	33	5	5	6	41	41	15	0	33	0	23	10
	2011	799	595	22	225	77	61	3	73	134	0	204	31	4	5	5	39	40	14	0	31	0	25	10
	2012	749	551	21	209	70	56	2	66	126	0	198	30	5	5	5	39	40	14	0	31	0	21	9
	2013	694	512	20	194	65	52	2	62	118	0	182	27	4	4	4	35	36	12	0	28	0	24	8

U5D[1]: total number of under 5 death; NEO[2]: total number of neonatal death; PNE[3]: pneumonia; PRE[4]: preterm; INT[5]: intrapartum related events; SEP[6]: sepsis/meningitis; TET[7]: tetanus; OTH[8]: other disorders; CON[9]: congenital; DIA[10]: diarrea; POS[11]: total number of post neonatal death; MEN[12]: meningitis; MEA[13]: measles; INJ[14]: injury; MAL[15]: malaria; PER[16]: pertussis

			0-28 days									1-59 months												
	Year	U5D[1]	NEO[2]	PNE[3]	PRE[4]	INT[5]	SEP[6]	TET[7]	OTH[8]	CON[9]	DIA[10]	POS[11]	PNE[3]	PRE[4]	INT[5]	MEN[12]	OTH[8]	CON[9]	DIA[10]	MEA[13]	INJ[14]	MAL[15]	AIDS	PER[16]
	2014	652	480	18	182	60	48	2	58	112	0	172	25	4	4	4	34	35	11	0	27	0	19	8
	2015	631	464	17	175	58	46	2	57	109	0	167	24	4	4	4	33	35	11	0	26	0	18	8
Mexico	2000	64086	25193	1475	9540	3971	3183	0	2081	4942	0	38893	8983	646	349	1004	9608	7456	4975	0	5760	1	103	8
	2001	59954	23032	1297	8694	3551	3051	0	1839	4600	0	36922	8019	683	430	780	9102	7210	4586	0	5989	0	98	24
	2002	55646	21060	1144	8065	3090	2883	2	1669	4206	0	34586	8155	717	335	692	8437	6426	4084	0	5619	0	112	10
	2003	52056	19578	1032	7590	2894	2536	0	1489	4037	0	32478	6862	805	368	700	8193	6407	3852	0	5130	0	143	18
	2004	49081	18875	1066	7326	2804	2386	0	1321	3973	0	30206	6907	720	380	647	7314	6195	3166	0	4728	0	133	15
	2005	46553	18490	1008	7083	2751	2440	0	1128	4080	0	28063	5887	733	398	529	7024	5891	3123	0	4344	0	87	48
	2006	44763	17648	974	6505	2567	2331	0	1080	4192	0	27115	5591	729	389	570	6715	5904	2572	0	4515	0	104	25
	2007	43385	16171	773	6152	2285	2089	0	1068	3804	0	27214	5104	830	332	547	7878	5863	2618	0	3931	0	104	8
	2008	42138	15453	726	5671	2136	2196	0	1015	3708	0	26685	4843	833	290	515	8053	6016	2201	0	3806	0	106	22
	2009	40965	16173	749	5926	2179	2330	0	1097	3893	0	24792	4643	777	246	376	7497	5720	1674	0	3699	0	103	57
	2010	39580	18086	898	6694	2365	2575	0	1167	4387	0	21494	3979	699	219	399	6520	5318	1307	0	2961	0	65	28
	2011	37776	19329	1044	6656	2642	2920	0	1298	4769	0	18447	3557	665	186	341	5003	4606	1151	0	2814	0	66	57
	2012	36230	19170	963	6616	2447	3013	0	1297	4834	0	17060	2962	627	210	267	4690	4446	1049	0	2684	0	48	78
	2013	34428	18284	817	6430	2320	2698	0	1196	4823	0	16144	2779	589	160	249	4749	4025	1035	0	2449	0	48	61
	2014	32785	17590	874	6105	2294	2672	0	1174	4472	0	15195	2727	554	164	251	4256	3847	953	0	2338	0	47	58
	2015	31278	16596	824	5760	2164	2521	0	1108	4219	0	14682	2635	535	158	242	4112	3718	920	0	2259	0	46	56
Nicaragua	2000	5511	2333	168	978	443	207	29	184	323	0	3178	825	23	16	91	788	336	853	0	161	6	52	25
	2001	5175	2238	160	930	419	200	27	178	324	0	2937	773	25	17	86	721	337	738	0	161	3	50	25
	2002	4853	2143	152	882	395	193	25	172	325	0	2710	705	24	17	79	663	327	665	0	155	2	48	25
	2003	4567	2050	144	838	372	185	23	165	323	0	2517	642	24	17	72	615	317	607	0	150	2	45	25
	2004	4326	1939	134	786	347	175	20	158	318	0	2387	599	24	16	67	580	311	569	0	149	2	43	27
	2005	4106	1852	126	745	327	164	23	153	315	0	2254	567	26	17	64	545	314	504	0	149	2	40	26
	2006	3932	1776	117	708	309	157	24	150	311	0	2156	550	28	18	62	518	320	445	0	153	1	36	26
	2007	3790	1721	110	679	294	152	28	149	308	0	2069	536	31	20	61	492	329	387	0	157	0	32	25
	2008	3666	1654	102	645	278	150	28	148	303	0	2012	525	33	22	60	475	337	347	0	160	0	28	24
	2009	3545	1608	97	621	266	149	28	147	300	0	1937	506	35	23	57	455	338	317	0	161	0	20	25
	2010	3405	1539	91	590	251	144	27	143	293	0	1866	483	36	24	54	436	336	296	0	159	0	17	26
	2011	3255	1469	86	560	237	136	26	138	286	0	1786	457	37	24	51	419	330	277	0	155	0	12	25
	2012	3102	1392	81	528	222	127	25	132	277	0	1710	432	37	24	48	402	323	260	0	151	0	9	24
	2013	2928	1313	76	496	207	119	23	126	266	0	1615	402	38	24	44	380	313	240	0	144	0	7	23
	2014	2787	1249	72	469	195	112	22	122	257	0	1538	379	37	24	41	362	303	225	0	138	0	7	22
	2015	2662	1170	67	437	181	104	21	116	244	0	1492	364	36	23	39	351	300	216	0	135	1	7	21
Panama	2000	1883	1071	74	377	154	118	0	41	307	0	812	111	7	1	28	300	208	88	0	56	0	13	0
	2001	1834	1043	54	471	109	82	0	70	257	0	791	103	4	0	26	272	215	96	0	62	0	8	4
	2002	1766	996	26	406	128	126	0	49	261	0	770	93	1	0	23	221	254	79	0	80	0	9	8
	2003	1683	953	20	296	144	102	0	116	274	0	730	102	6	1	11	206	216	90	0	88	0	7	1
	2004	1616	909	31	315	109	110	0	68	276	0	707	104	7	1	22	209	187	91	0	68	0	14	3
	2005	1567	883	64	272	112	104	0	64	267	0	684	130	3	3	15	224	118	112	0	71	0	7	1
	2006	1539	866	34	291	114	137	0	76	215	0	673	122	8	2	17	177	196	63	0	68	0	6	13
	2007	1528	865	36	270	127	142	0	49	241	0	663	146	3	2	16	175	126	106	0	74	0	10	6
	2008	1517	869	43	217	165	132	0	45	268	0	648	157	1	1	10	177	162	65	0	66	0	4	5
	2009	1516	862	33	290	95	136	0	72	235	0	654	101	4	2	22	171	139	137	0	65	0	3	9
	2010	1489	854	48	215	109	157	0	61	263	0	635	180	4	3	20	153	113	87	0	68	0	4	2
	2011	1456	830	48	225	107	156	0	59	235	0	626	160	1	2	21	159	151	74	0	53	0	1	2
	2012	1421	808	31	286	116	97	0	55	222	0	613	137	1	3	14	175	163	71	0	46	0	2	0
	2013	1371	774	43	275	117	91	0	67	180	0	597	145	1	3	16	178	147	74	0	28	0	5	1
	2014	1327	747	38	244	105	106	0	57	197	0	580	139	1	3	16	162	146	69	0	40	0	3	1
	2015	1277	721	36	236	102	103	0	55	190	0	556	134	1	3	15	155	140	67	0	39	0	2	1
Paraguay	2000	4912	2595	110	1095	377	347	63	208	395	0	2317	595	11	6	65	454	328	458	0	232	0	15	153
	2001	4692	2473	105	1034	353	311	82	205	384	0	2219	567	12	6	62	424	323	424	0	232	0	16	152
	2002	4433	2331	98	966	327	333	37	198	371	0	2102	532	13	7	58	386	313	393	0	228	0	18	154
	2003	4152	2201	92	908	305	309	36	190	361	0	1951	452	19	10	67	398	305	323	0	203	0	20	154
	2004	3916	2077	89	856	285	291	28	178	350	0	1839	426	22	12	64	358	300	282	0	201	0	22	153
	2005	3735	1998	86	823	272	279	20	171	349	0	1737	403	24	13	60	326	295	244	0	198	0	25	149
	2006	3634	1954	84	805	265	265	17	166	352	0	1680	386	26	14	57	313	295	225	0	197	0	20	147
	2007	3583	1935	83	798	261	247	21	163	361	0	1648	370	28	15	55	310	293	223	0	195	0	13	146
	2008	3553	1925	82	793	260	237	21	163	370	0	1628	353	30	17	52	309	290	226	0	192	0	14	147
	2009	3519	1917	80	788	257	229	21	163	378	0	1602	337	32	18	49	299	286	226	0	189	0	16	151
	2010	3446	1871	77	767	250	226	14	160	378	0	1575	324	35	20	47	292	286	216	0	187	0	18	150
	2011	3330	1807	73	738	241	216	12	155	373	0	1523	308	38	22	45	270	281	204	0	181	0	20	155
	2012	3192	1725	69	704	228	201	11	147	363	0	1467	292	41	23	42	248	274	194	0	174	0	21	159
	2013	3039	1635	65	666	215	187	11	140	351	0	1404	274	43	25	39	236	264	184	0	165	0	22	152
	2014	2896	1552	62	631	203	174	10	133	339	0	1344	257	46	26	36	223	256	174	0	156	0	24	146
	2015	2780	1471	58	596	192	162	10	127	327	0	1309	247	50	28	34	215	252	167	0	151	0	23	142
Peru	2000	23784	9652	557	3999	1641	1112	201	763	1379	0	14132	3395	75	88	606	3760	1897	2049	0	1866	6	283	106
	2001	21979	9162	497	3693	1515	1047	224	799	1387	0	12817	2981	70	83	526	3397	1777	1858	0	1724	8	280	113
	2002	20439	8758	462	3458	1416	984	219	813	1407	0	11681	2633	68	82	463	3093	1679	1690	0	1590	4	269	110
	2003	19121	8362	434	3248	1326	924	206	809	1415	0	10759	2331	65	79	407	2857	1583	1606	0	1463	3	255	110
	2004	17917	8019	410	3073	1248	881	187	794	1425	0	9898	2011	80	97	369	2663	1551	1464	0	1306	2	248	109
	2005	16867	7719	390	2935	1181	832	182	767	1432	0	9148	1809	77	94	329	2457	1477	1339	0	1216	1	243	108
	2006	15893	7378	371	2798	1110	786	165	722	1426	0	8515	1679	78	96	304	2256	1457	1136	0	1171	2	230	106
	2007	14947	7085	355	2693	1050	741	145	675	1427	0	7862	1542	79	96	279	2063	1422	953	0	1122	1	198	107
	2008	14063	6731	334	2572	982	668	140	626	1409	0	7332	1417	79	97	257	1912	1390	817	0	1084	1	173	106
	2009	13339	6459	318	2482	928	608	136	591	1397	0	6880	1292	78	95	235	1792	1341	736	0	1048	1	157	106
	2010	12676	6157	297	2379	870	612	72	557	1369	0	6519	1191	77	94	218	1693	1307	664	0	1030	0	140	106
	2011	12116	5942	278	2303	826	596	52	537	1351	0	6174	1100	76	93	202	1613	1271	611	0	1008	0	93	107
	2012	11658	5750	260	2233	787	565	50	523	1333	0	5908	1020	77	94	188	1550	1244	566	0	984	1	80	105
	2013	11267	5498	240	2134	743	528	48	507	1297	0	5769	970	75	91	177	1527	1225	555	0	970	1	75	103
	2014	10843	5349	224	2075	717	503	47	502	1281	0	5494	905	74	91	163	1470	1191	527	0	927	0	49	98
	2015	10483	5104	208	1978	681	470	45	486	1237	0	5379	868	75	92	155	1443	1181	514	0	908	0	47	96
Saint Kitts and Nevis	2000	19	12	0	6	3	1	0	1	1	0	7	1	0	0	0	3	2	1	0	0	0	0	0
	2001	18	11	0	5	4	0	0	1	0	0	7	1	0	1	0	3	2	0	0	1	0	0	0
	2002	16	10	0	4	5	0	0	1	0	0	6	1	0	0	0	2	1	0	0	1	0	0	0
	2003	15	10	0	3	5	0	0	1	1	0	5	1	0	1	0	2	1	0	0	1	0	0	0
	2004	14	9	0	3	3	1	0	0	1	0	5	0	0	2	0	2	1	0	0	0	0	0	0
	2005	14	8	0	4	3	1	0	0	1	0	6	0	0	2	0	2	1	0	0	1	0	0	0
	2006	13	8	0	3	3	1	0	1	1	0	5	0	0	1	0	1	1	0	0	1	0	0	0
	2007	13	8	0	2	3	1	0	1	1	0	5	0	0	0	0	2	0	0	0	2	0	0	0
	2008	13	8	0	2	3	0	0	1	1	0	5	0	1	0	0	3	0	0	0	1	0	0	0
	2009	13	8	0	2	4	1	0	1	1	0	5	0	1	0	0	3	0	0	0	1	0	0	0
	2010	12	8	0	2	4	0	0	0	0	0	4	0	1	0	0	2	0	0	0	1	0	0	0
	2011	12	7	0	1	4	0	0	0	1	0	5	0	1	0	0	2	1	0	0	1	0	0	0
	2012	11	7	0	2	4	0	0	0	1	0	4	0	0	0	0	2	1	0	0	0	0	0	0
	2013	11	7	0	1	4	0	0	0	2	0	4	0	1	0	1	1	1	1	0	0	0	0	0
	2014	10	6	0	1	4	1	0	0	1	0	4	0	1	0	0	2	1	0	0	1	0	0	0
	2015	10	6	0	1	4	1	0	0	1	0	4	0	1	0	0	2	1	0	0	1	0	0	0
Saint Lucia	2000	55	34	0	17	6	6	0	2	5	0	21	2	0	0	0	9	3	1	0	3	0	1	0
	2001	53	33	0	17	6	4	0	2	4	0	20	3	0	0	0	10	2	1	0	4	0	1	0
	2002	51	33	0	17	7	3	0	2	4	0	18	3	0	0	0	8	3	0	0	3	0	0	0
	2003	50	32	0	16	6	4	0	1	5	0	18	2	0	0	0	8	3	0	0	4	0	0	0
	2004	49	32	0	14	6	4	0	2	6	0	17	2	0	0	0	6	4	0	0	3	0	0	0
	2005	49	32	0	12	5	6	0	4	4	0	17	3	0	0	0	6	3	0	0	3	0	0	0
	2006	48	31	1	9	5	5	0	6	5	0	17	4	0	0	0	7	3	0	0	2	0	0	0
	2007	48	31	2	6	2	4	0	8	9	0	17	3	0	0	0	10	2	0	0	1	0	0	0
	2008	47	31	2	6	4	2	0	8	9	0	16	2	0	0	0	9	4	0	0	0	0	0	0

U5D[1]: total number of under 5 death; NEO[2]: total number of neonatal death; PNE[3]: pneumonia; PRE[4]: preterm; INT[5]: intrapartum related events; SEP[6]: sepsis/meningitis; TET[7]: tetanus; OTH[8]: other disorders; CON[9]: congenital; DIA[10]: diarrea; POS[11]: total number of post neonatal death; MEN[12]: meningitis; MEA[13]: measles; INJ[14]: injury; MAL[15]: malaria; PER[16]: pertussis

		0-28 days										1-59 months												
	Year	U5D[1]	NEO[2]	PNE[3]	PRE[4]	INT[5]	SEP[6]	TET[7]	OTH[8]	CON[9]	DIA[10]	POS[11]	PNE[3]	PRE[4]	INT[5]	MEN[12]	OTH[8]	CON[9]	DIA[10]	MEA[13]	INJ[14]	MAL[15]	AIDS	PER[16]
	2009	47	30	2	9	5	2	0	5	8	0	17	3	0	0	1	9	5	0	0	0	0	0	0
	2010	46	30	1	12	7	4	0	3	3	0	16	2	1	0	1	7	5	0	0	0	0	0	0
	2011	45	29	1	13	5	5	0	2	3	0	16	2	1	0	1	6	4	0	0	2	0	0	0
	2012	44	29	1	13	5	5	0	3	3	0	15	1	2	0	0	7	3	0	0	2	0	0	0
	2013	43	28	1	14	5	3	0	2	3	0	15	1	1	0	0	6	4	0	0	3	0	0	0
	2014	42	27	1	12	5	4	0	2	3	0	15	2	1	0	0	6	4	0	0	2	0	0	0
	2015	40	26	1	12	5	4	0	2	3	0	14	1	1	0	0	6	4	0	0	2	0	0	0
Saint Vincent and the Grenadines	2000	48	29	0	16	5	2	0	1	6	0	19	2	2	1	1	6	2	2	0	3	0	2	0
	2001	46	28	0	14	4	3	0	2	4	0	18	2	2	1	0	6	2	1	0	3	0	2	0
	2002	44	27	0	13	5	4	0	4	2	0	17	1	1	2	0	6	1	2	0	4	0	1	0
	2003	43	27	0	13	3	4	0	5	2	0	16	1	2	2	0	4	2	1	0	4	0	1	0
	2004	42	26	0	13	2	3	0	4	4	0	16	1	1	2	0	4	2	2	0	3	0	1	0
	2005	41	26	0	12	2	5	0	3	4	0	15	1	2	1	0	5	3	1	0	2	0	0	0
	2006	41	26	0	12	4	6	0	1	3	0	15	1	1	0	0	8	3	1	0	1	0	1	0
	2007	41	26	0	11	4	8	0	1	3	0	15	0	1	0	0	10	2	0	0	1	0	1	0
	2008	42	26	1	12	3	6	0	2	2	0	16	0	0	0	0	11	1	0	0	3	0	1	0
	2009	42	26	0	13	3	5	0	2	2	0	16	1	0	0	0	10	1	0	0	3	0	0	0
	2010	41	26	1	15	3	3	0	1	3	0	15	1	0	0	0	7	4	0	0	3	0	0	0
	2011	39	25	0	16	4	2	0	0	2	0	14	2	0	0	0	6	5	0	0	1	0	0	0
	2012	37	23	0	13	4	3	0	1	2	0	14	2	1	0	0	5	5	0	0	2	0	0	0
	2013	34	22	0	11	4	4	0	1	2	0	12	2	1	0	0	4	3	0	0	2	0	0	0
	2014	32	20	0	9	4	3	0	1	2	0	12	2	2	0	0	4	3	0	0	1	0	0	0
	2015	30	19	0	10	3	3	0	1	2	0	11	1	1	0	0	4	3	0	0	1	0	0	0
Suriname	2000	373	200	11	75	26	55	0	2	30	0	173	19	3	3	8	54	15	25	0	31	11	5	0
	2001	355	191	4	73	43	18	0	27	26	0	164	20	9	0	6	51	19	10	0	21	10	17	0
	2002	335	179	0	66	55	22	0	6	30	0	156	10	5	2	14	63	16	5	0	18	6	17	0
	2003	317	170	0	90	39	14	0	5	22	0	147	30	2	0	3	49	20	8	0	18	7	10	0
	2004	301	161	0	71	38	18	0	10	25	0	140	12	4	0	6	52	20	11	0	26	2	8	0
	2005	289	156	0	77	35	12	0	11	22	0	133	24	4	0	2	61	13	4	0	21	0	6	0
	2006	278	150	0	63	35	27	0	6	19	0	128	16	4	3	7	38	22	6	0	26	0	6	0
	2007	270	146	1	62	21	28	0	11	23	0	124	22	9	0	2	30	23	5	0	23	0	8	0
	2008	262	142	1	59	25	22	0	4	31	0	120	14	5	3	5	45	16	3	0	16	0	12	0
	2009	256	139	0	59	23	22	0	11	23	0	117	20	2	4	6	41	16	6	0	17	0	6	0
	2010	251	136	2	57	22	22	0	10	22	0	115	20	3	3	10	28	21	0	0	26	0	5	0
	2011	243	131	2	46	24	28	0	8	23	0	112	13	6	7	5	29	34	3	0	14	0	0	0
	2012	235	126	0	62	21	17	0	5	21	0	109	18	3	3	0	33	12	6	0	25	0	9	0
	2013	225	121	1	51	21	20	0	7	21	0	104	16	4	4	4	28	20	3	0	20	0	4	0
	2014	216	116	1	49	20	20	0	7	20	0	100	15	3	4	4	27	20	3	0	19	0	4	0
	2015	209	112	1	47	19	19	0	6	19	0	97	15	3	4	4	26	19	3	0	19	0	4	0
Trinidad and Tobago	2000	529	344	8	152	128	15	0	7	34	0	185	9	46	13	6	46	32	5	0	11	0	16	0
	2001	529	345	15	155	49	28	0	26	72	0	184	12	0	0	3	53	39	7	0	49	0	22	0
	2002	533	349	16	119	32	42	0	42	97	0	184	16	0	0	4	62	19	0	0	49	0	35	0
	2003	539	352	9	151	34	50	0	33	74	0	187	31	0	2	9	63	31	17	0	25	0	8	0
	2004	541	353	12	130	56	37	0	47	71	0	188	25	5	5	2	62	42	0	0	26	0	20	0
	2005	538	351	20	124	50	41	0	37	78	0	187	22	2	2	4	55	55	8	0	28	0	10	0
	2006	532	348	14	93	72	30	0	37	102	0	184	15	10	2	5	56	44	6	0	34	0	12	0
	2007	520	339	16	105	60	16	0	67	76	0	181	23	0	0	3	64	50	0	0	26	0	15	0
	2008	505	330	23	127	42	19	0	40	79	0	175	14	0	0	4	55	48	4	0	44	0	6	0
	2009	491	320	25	138	38	20	0	55	45	0	171	15	0	0	3	76	48	2	0	26	0	2	0
	2010	474	308	20	115	44	17	0	51	61	0	166	16	0	0	3	62	46	2	0	30	0	7	0
	2011	457	297	19	111	43	16	0	49	59	0	160	16	0	0	3	59	44	2	0	29	0	7	0
	2012	440	286	19	107	41	15	0	47	57	0	154	15	0	0	3	57	43	2	0	28	0	7	0
	2013	421	273	18	102	39	15	0	45	54	0	148	14	0	0	3	55	41	2	0	27	0	6	0
	2014	404	261	17	98	38	14	0	43	52	0	143	14	0	0	3	53	40	2	0	26	0	6	0
	2015	387	249	16	93	36	13	0	41	49	0	138	13	0	0	3	51	38	2	0	25	0	6	0
Uruguay	2000	890	441	11	173	59	58	0	32	108	0	449	68	22	7	11	127	123	28	0	61	0	3	0
	2001	850	432	10	148	61	62	0	39	114	0	418	82	12	2	21	104	112	16	0	63	0	6	0
	2002	824	425	10	151	50	58	0	41	115	0	399	74	19	3	16	94	103	20	0	65	0	3	0
	2003	794	413	9	152	40	53	0	44	114	0	381	71	15	3	16	90	102	19	0	62	0	3	0
	2004	766	397	9	152	30	49	0	45	113	0	369	52	22	0	15	80	111	18	0	69	0	2	0
	2005	737	381	7	146	34	49	0	41	104	0	356	66	13	2	16	89	96	15	0	57	0	0	0
	2006	707	358	5	139	36	48	0	36	93	0	349	61	13	2	14	91	101	13	0	54	0	0	0
	2007	677	335	4	131	38	48	0	32	83	0	342	72	0	2	22	88	83	13	0	62	0	0	0
	2008	658	323	4	120	36	37	0	20	107	0	335	66	19	2	13	84	89	10	0	53	0	0	0
	2009	638	311	8	99	24	62	0	25	93	0	327	63	9	2	16	92	89	12	0	45	0	0	0
	2010	614	304	6	99	29	51	0	24	94	0	310	47	12	1	11	82	98	10	0	46	0	0	3
	2011	595	292	5	97	34	41	0	22	93	0	303	41	12	1	9	82	101	9	0	44	0	0	4
	2012	571	285	3	97	40	32	0	20	93	0	286	37	11	0	6	77	109	4	0	35	0	0	8
	2013	542	268	1	76	40	40	0	22	90	0	274	23	13	0	8	74	97	11	0	42	0	0	6
	2014	514	256	3	81	35	34	0	19	84	0	258	30	11	0	7	70	92	7	0	36	0	0	5
	2015	487	246	3	78	33	33	0	19	80	0	241	28	10	0	6	65	86	7	0	34	0	0	5
Venezuela (Bolivarian Republic of)	2000	12577	6957	207	3112	1120	1227	0	313	979	0	5620	807	57	13	235	1432	894	1307	0	777	15	26	58
	2001	12257	6777	214	3047	1126	1016	0	368	1006	0	5480	900	80	23	186	1407	889	1159	0	769	8	18	41
	2002	11977	6525	221	2993	1081	862	0	399	970	0	5452	746	61	23	168	1311	975	1226	2	874	10	30	27
	2003	11677	6201	182	2755	1046	1022	0	375	821	0	5476	874	52	19	118	1406	757	1480	0	700	10	32	27
	2004	11363	5808	238	2516	869	1014	0	301	869	0	5555	910	57	21	174	1235	968	1224	0	862	12	26	66
	2005	10980	5525	262	2486	811	842	0	321	802	0	5455	789	76	24	141	953	1148	1186	0	1049	13	31	46
	2006	10653	5597	337	2362	821	779	0	395	904	0	5056	796	88	21	128	964	1100	953	0	912	12	39	42
	2007	10371	5783	328	2399	897	920	2	315	923	0	4588	753	86	15	123	835	1066	734	0	922	14	30	10
	2008	10198	6025	409	2408	927	1053	0	285	943	0	4173	712	30	29	129	888	952	541	0	818	10	25	37
	2009	10023	6086	424	2420	899	1107	0	289	946	0	3937	650	84	38	107	818	859	452	0	833	15	32	51
	2010	9909	6028	420	2342	819	1130	0	290	1027	0	3881	627	71	22	102	726	934	551	0	773	20	31	24
	2011	9809	5919	412	2363	867	985	1	333	957	0	3890	660	81	29	129	702	943	505	0	776	19	26	20
	2012	9648	5810	409	2341	799	878	1	318	1064	0	3838	628	102	38	116	707	959	407	0	783	23	30	45
	2013	9424	5700	398	2262	798	960	1	302	979	0	3724	611	81	29	111	681	905	467	0	744	39	28	29
	2014	9191	5524	386	2192	774	930	1	293	949	0	3667	602	80	28	109	671	892	460	0	733	36	27	28
	2015	8946	5339	373	2119	748	899	1	283	917	0	3607	591	79	28	107	659	876	452	0	720	40	27	28
Northern Africa	2000	143144	71740	5313	29166	15625	6337	1426	4476	9241	157	71404	20490	2870	899	1735	14668	8337	12090	3657	6066	0	43	548
	2001	137498	70368	5019	28392	15158	6437	1281	4442	9496	143	67130	18989	2803	867	1605	13981	8138	10859	3401	5897	0	46	543
	2002	132032	69440	4799	27848	14799	6487	1136	4446	9792	133	62592	17619	2727	835	1485	13362	7926	9930	2377	5737	0	50	543
	2003	128663	68925	4628	27509	14548	6494	1021	4470	10128	126	59738	16357	2695	818	1387	12780	7708	9064	2765	5569	0	55	540
	2004	123103	68354	4460	27071	14303	6569	895	4502	10434	121	54749	15405	2651	798	1304	12324	7566	8373	241	5485	0	60	543
	2005	120254	68076	4314	26730	14140	6637	825	4540	10774	116	52178	14485	2591	775	1230	11838	7391	7663	195	5398	0	61	551
	2006	118211	67365	4146	26212	13916	6642	805	4525	11007	113	50846	13808	2528	754	1170	11510	7293	7123	642	5391	0	63	565
	2007	116618	66139	3958	25476	13608	6597	801	4469	11120	110	50479	12760	2543	764	999	11500	7309	6806	1646	5495	0	68	589
	2008	113585	65155	3794	24760	13342	6605	780	4529	11238	108	48430	11724	2561	769	847	11485	7317	6537	904	5598	0	66	621
	2009	112149	64610	3680	24183	13177	6662	758	4634	11410	106	47539	11362	2567	769	822	11391	7310	6260	709	5638	0	63	646
	2010	111232	64458	3613	23754	13095	6778	712	4778	11625	102	46774	11138	2603	778	804	11419	7381	6092	138	5696	0	59	667
	2011	112666	65286	3618	23773	13176	6885	680	5027	12029	99	47380	11009	2711	813	781	11692	7584	5837	142	6063	0	64	685
	2012	114517	66463	3648	24009	13307	6912	698	5305	12490	95	48054	10813	2880	861	722	12060	7900	5848	200	6001	0	67	702
	2013	116664	67549	3681	24294	13396	6867	720	5569	12930	91	49115	10883	3031	900	712	12341	8216	5785	344	6131	0	60	712
	2014	116456	67521	3644	24168	13258	6744	727	5746	13147	87	48935	10789	3126	920	698	12353	8378	5619	133	6129	0	79	710
	2015	114406	65915	3527	23486	12827	6515	710	5772	12995	83	48491	10613	3178	929	680	12248	8436	5415	129	6081	0	78	703
Algeria	2000	23449	11908	635	4677	2603	1507	90	658	1697	40	11541	3106	437	129	360	2413	785	1923	1139	1137	0	6	106
	2001	22964	11743	624	4614	2553	1485	85	649	1695	39	11221	2967	422	124	341	2308	772	1779	1305	1092	0	6	104
	2002	22676	11769	623	4599	2552	1489	81	654	1733	37	10907	2844	411	122	324	2214	770	1649	1409	1055	0	7	104
	2003	23628	11978	633	4633	2597	1519	79	670	1810	37	11650	2751	402	119	310	2140	769	1540	2473	1031	0	9	106

U5D[1]: total number of under 5 death; NEO[2]: total number of neonatal death; PNE[3]: pneumonia; PRE[4]: preterm; INT[5]: intrapartum related events; SEP[6]: sepsis/meningitis; TET[7]: tetanus; OTH[8]: other disorders; CON[9]: congenital; DIA[10]: diarrea; POS[11]: total number of post neonatal death; MEN[12]: meningitis; MEA[13]: measles; INJ[14]: injury; MAL[15]: malaria; PER[16]: pertussis

						0-28 days										1-59 months								
	Year	U5D[1]	NEO[2]	PNE[3]	PRE[4]	INT[5]	SEP[6]	TET[7]	OTH[8]	CON[9]	DIA[10]	POS[11]	PNE[3]	PRE[4]	INT[5]	MEN[12]	OTH[8]	CON[9]	DIA[10]	MEA[13]	INJ[14]	MAL[15]	AIDS	PER[16]
	2004	21343	12342	652	4704	2681	1574	77	697	1920	37	9001	2689	397	118	300	2089	777	1452	38	1018	0	11	110
	2005	21651	12811	678	4806	2789	1642	75	729	2056	37	8840	2644	396	118	294	2049	791	1377	30	1013	0	13	117
	2006	22210	13301	703	4908	2902	1712	73	763	2203	36	8909	2664	402	121	295	2063	817	1337	39	1033	0	15	124
	2007	22945	13949	739	5070	3047	1799	73	805	2380	37	8996	2691	409	123	298	2074	847	1302	40	1055	0	19	138
	2008	23695	14482	765	5143	3173	1912	69	850	2535	37	9213	2292	461	140	197	2332	963	1410	50	1197	0	17	155
	2009	24328	15013	790	5171	3301	2070	63	906	2677	36	9315	2310	469	144	197	2361	990	1381	58	1225	0	15	165
	2010	24692	15299	798	5065	3373	2252	54	964	2758	34	9393	2335	473	147	199	2384	1001	1350	67	1250	0	13	174
	2011	24930	15474	801	4988	3414	2372	48	1009	2810	33	9456	2352	476	151	200	2401	1023	1316	75	1266	0	17	178
	2012	25026	15456	796	4915	3407	2410	45	1027	2824	31	9570	2383	482	156	202	2438	1047	1293	80	1289	0	21	178
	2013	24984	15340	789	4878	3375	2383	43	1020	2820	31	9644	2405	488	158	204	2465	1068	1267	91	1304	0	15	179
	2014	24676	15138	777	4824	3322	2343	42	1007	2792	30	9538	2395	488	158	203	2458	1074	1226	26	1302	0	30	178
	2015	24269	14773	756	4727	3232	2279	41	982	2727	29	9496	2389	489	159	202	2454	1082	1189	25	1300	0	30	177
Egypt	2000	80229	38142	3403	16869	8049	1515	1059	2357	4890	0	42087	12142	1861	465	661	8438	5901	6960	2460	2891	0	6	301
	2001	77381	37842	3201	16576	7910	1611	1001	2357	5187	0	39539	11214	1846	461	613	8110	5808	6239	2066	2877	0	7	299
	2002	74203	37705	3050	16352	7802	1737	887	2395	5482	0	36498	10345	1813	453	566	7810	5677	5752	925	2849	0	8	299
	2003	71526	37629	2926	16163	7711	1828	789	2447	5765	0	33897	9536	1778	444	523	7479	5531	5279	212	2813	0	9	292
	2004	69555	37117	2780	15792	7533	1894	679	2477	5962	0	32438	8905	1758	440	489	7243	5441	4896	153	2812	0	10	292
	2005	67458	36696	2646	15475	7381	1917	623	2498	6156	0	30762	8248	1724	431	454	6936	5308	4447	121	2790	0	11	292
	2006	65666	35636	2475	14921	7116	1854	613	2450	6207	0	30030	7719	1700	425	427	6733	5228	4095	587	2803	0	12	299
	2007	63953	33888	2273	14090	6719	1727	623	2351	6104	0	30065	7257	1683	421	405	6583	5177	3800	1589	2830	0	13	308
	2008	60716	32428	2094	13345	6365	1639	611	2360	6014	0	28288	6792	1654	414	381	6398	5093	3541	839	2838	0	15	323
	2009	59103	31520	1971	12824	6117	1593	600	2408	6007	0	27583	6480	1656	414	364	6329	5089	3376	647	2876	0	17	335
	2010	58411	31379	1912	12637	6029	1618	568	2501	6115	0	27032	6305	1693	423	354	6393	5176	3328	64	2938	0	18	342
	2011	59907	32414	1934	12891	6150	1716	547	2717	6459	0	27493	6257	1762	441	348	6552	5343	3344	57	3022	0	19	348
	2012	62548	33980	1991	13350	6370	1800	573	2990	6905	0	28568	6342	1882	470	349	6844	5630	3416	111	3147	0	21	355
	2013	65368	35645	2058	13831	6601	1886	601	3290	7378	0	29723	6464	2021	505	351	7105	5943	3425	246	3272	0	22	367
	2014	66378	36401	2069	13949	6658	1919	614	3519	7672	0	29977	6449	2125	531	345	7201	6141	3366	101	3320	0	25	372
	2015	65775	35818	2014	13569	6476	1877	604	3619	7658	0	29957	6362	2193	548	335	7191	6237	3271	98	3326	0	25	372
Libya	2000	3351	1813	67	746	357	139	0	143	361	0	1538	302	14	4	28	447	302	99	0	323	0	0	19
	2001	3311	1795	66	730	349	138	0	149	364	0	1516	290	13	4	27	441	299	95	0	328	0	0	20
	2002	3238	1746	64	707	332	135	0	147	360	0	1492	277	13	4	26	433	297	90	0	332	0	0	21
	2003	3126	1690	60	684	314	132	0	144	356	0	1436	259	13	4	24	418	289	81	0	327	0	0	21
	2004	3013	1628	56	656	295	128	0	140	352	0	1385	243	13	4	23	403	284	72	0	323	0	0	22
	2005	2901	1567	52	628	277	123	0	138	349	0	1334	226	13	4	21	389	278	64	0	317	0	0	23
	2006	2811	1527	49	608	263	120	0	137	351	0	1284	209	12	3	20	376	273	57	0	310	0	0	23
	2007	2717	1466	45	579	247	113	0	134	348	0	1251	186	16	4	23	379	278	50	0	292	0	0	23
	2008	2621	1422	42	558	234	107	0	132	347	0	1199	170	19	5	22	358	282	46	0	275	0	0	23
	2009	2512	1369	40	535	221	101	0	129	342	0	1143	158	19	5	20	338	275	44	0	261	0	0	23
	2010	2378	1286	38	502	204	93	0	123	327	0	1092	149	19	5	18	319	269	43	0	247	0	0	23
	2011	2503	1218	37	473	190	86	0	118	313	0	1285	144	20	6	17	304	265	43	0	465	0	0	22
	2012	2101	1127	34	438	174	78	0	110	293	0	974	131	19	5	15	278	249	40	0	214	0	0	22
	2013	1946	1042	32	404	159	70	0	103	273	0	904	120	18	5	14	257	235	38	0	197	0	0	20
	2014	1797	962	29	373	146	63	0	96	255	0	835	109	17	5	12	238	221	35	0	180	0	0	19
	2015	1684	894	26	346	135	57	0	91	239	0	790	101	16	5	11	226	212	32	0	170	0	0	18
Morocco	2000	30721	16765	985	5632	4021	2992	209	1060	1749	116	13956	4341	471	276	649	2787	940	2811	58	1483	0	31	109
	2001	28853	16102	927	5342	3805	3008	159	1036	1721	104	12751	3988	436	252	591	2581	870	2483	30	1381	0	33	107
	2002	27277	15542	880	5156	3617	2950	132	1007	1704	95	11735	3677	405	231	539	2398	808	2202	44	1291	0	35	105
	2003	26084	15133	845	5079	3471	2855	119	975	1699	90	10951	3430	380	215	497	2251	760	1977	80	1219	0	37	107
	2004	25154	14907	822	5033	3370	2823	106	957	1712	84	10247	3226	360	201	462	2135	720	1790	50	1161	0	38	104
	2005	24433	14776	804	4995	3298	2817	96	950	1737	80	9657	3051	342	189	433	2035	681	1631	44	1111	0	36	104
	2006	23888	14782	795	4998	3264	2829	88	951	1780	76	9106	2894	324	179	406	1943	646	1491	17	1066	0	35	104
	2007	23510	14798	789	4997	3241	2834	82	954	1827	74	8712	2329	345	190	254	2085	685	1524	17	1143	0	35	105
	2008	23175	14842	789	5002	3230	2830	77	958	1884	72	8333	2196	334	183	230	2035	663	1422	15	1119	0	33	103
	2009	22936	14798	786	4973	3213	2790	73	959	1933	70	8138	2156	327	178	224	2011	642	1348	5	1110	0	30	106
	2010	22552	14619	777	4889	3173	2714	68	954	1974	68	7933	2105	320	173	217	1981	624	1268	7	1100	0	27	111
	2011	22189	14336	764	4775	3114	2615	63	942	1997	66	7853	2042	336	180	197	2090	649	1040	9	1164	0	26	120
	2012	21733	14069	749	4669	3051	2531	59	930	2016	63	7664	1762	348	185	138	2173	667	1015	9	1216	0	23	130
	2013	21279	13715	728	4554	2962	2438	54	908	2010	61	7564	1704	346	184	127	2188	661	974	7	1224	0	22	128
	2014	20579	13240	699	4407	2839	2333	50	874	1982	57	7339	1656	335	178	123	2141	638	915	7	1200	0	22	124
	2015	19759	12706	665	4250	2700	2222	45	835	1935	53	7053	1593	322	171	118	2076	609	852	7	1165	0	21	120
Tunisia	2000	5394	3112	223	1241	595	184	68	257	544	0	2282	599	86	26	37	584	408	298	0	232	0	1	13
	2001	4988	2886	201	1132	542	196	35	251	530	0	2102	531	85	25	33	542	389	262	0	220	0	1	13
	2002	4637	2678	182	1034	495	175	36	242	513	0	1959	476	85	25	30	507	374	236	0	210	0	1	14
	2003	4300	2495	164	950	455	160	35	234	497	0	1805	380	123	36	33	493	359	187	0	179	0	1	14
	2004	4039	2360	150	886	424	149	33	230	488	0	1679	342	123	36	30	454	345	163	0	171	0	1	15
	2005	3811	2226	135	826	395	138	31	226	476	0	1585	315	117	34	28	429	333	145	0	167	0	2	15
	2006	3636	2119	123	777	371	128	30	224	466	0	1517	321	89	26	21	394	328	143	0	179	0	2	15
	2007	3493	2038	113	739	353	124	23	225	461	0	1455	297	90	26	20	379	322	129	0	175	0	2	15
	2008	3378	1981	104	712	340	116	22	229	458	0	1397	275	92	27	18	362	316	118	0	170	0	2	17
	2009	3270	1910	95	680	324	107	22	231	451	0	1360	258	95	28	17	352	314	110	0	166	0	2	17
	2010	3199	1875	88	662	315	101	22	237	450	0	1324	243	98	29	16	343	311	104	0	162	0	2	17
	2011	3137	1844	82	646	308	96	22	241	449	0	1293	215	117	35	18	345	304	92	0	146	0	3	17
	2012	3110	1831	78	637	304	93	21	247	451	0	1279	195	150	44	17	328	306	84	0	135	0	3	17
	2013	3087	1807	74	626	298	89	21	248	450	0	1280	190	158	47	17	326	310	81	0	133	0	2	17
	2014	3026	1780	70	615	293	86	21	249	447	0	1246	180	160	47	15	315	304	76	0	128	0	3	17
	2015	2919	1724	65	594	282	80	20	245	437	0	1195	168	157	47	14	301	296	71	0	121	0	3	16
Oceania	2000	16591	6458	420	2197	1767	829	115	481	596	53	10133	2153	197	123	403	1266	372	1440	1124	668	1989	209	187
	2001	16929	6449	420	2158	1778	839	114	483	604	53	10480	2228	204	129	420	1315	385	1445	1346	692	1890	234	195
	2002	17400	6431	419	2124	1781	850	112	484	609	52	10969	2365	217	138	449	1401	405	1485	1777	735	1538	255	204
	2003	16621	6406	418	2097	1777	856	110	483	614	51	10215	2295	211	134	438	1375	395	1395	1050	719	1741	270	193
	2004	16040	6377	416	2080	1768	858	106	482	618	50	9663	2107	195	123	402	1269	371	1243	502	667	2324	280	181
	2005	15840	6357	413	2057	1766	862	102	484	624	49	9483	2355	217	140	448	1419	414	1349	304	747	1604	282	203
	2006	15589	6374	415	2041	1776	872	98	488	634	49	9215	2318	215	137	439	1406	413	1286	67	744	1711	280	198
	2007	15646	6401	417	2035	1786	882	95	493	646	48	9245	2378	222	141	446	1443	428	1276	95	773	1553	274	216
	2008	15628	6435	417	2041	1788	896	91	496	660	46	9193	2393	227	145	440	1469	440	1256	121	798	1414	257	232
	2009	15617	6452	416	2056	1775	909	87	492	671	45	9165	1927	218	138	307	1430	428	1158	180	802	2139	231	206
	2010	15296	6394	409	2041	1742	917	81	487	674	43	8902	2129	238	150	341	1545	461	1215	79	846	1438	228	233
	2011	15062	6287	399	2006	1697	918	75	477	673	41	8775	2166	247	157	339	1608	475	1211	127	880	1111	215	240
	2012	14710	6165	390	1963	1652	915	69	466	671	39	8545	1936	223	141	299	1455	435	1052	142	803	1644	197	219
	2013	14234	6005	377	1920	1596	896	65	451	664	37	8229	1866	219	139	282	1433	427	995	43	793	1651	170	211
	2014	13832	5869	366	1884	1549	875	61	438	660	35	7963	1876	217	137	287	1417	422	948	69	790	1456	137	209
	2015	13449	5781	359	1867	1516	856	59	428	663	34	7668	1805	210	132	275	1370	408	881	66	768	1421	132	200
Cook Islands	2000	7	4	0	1	1	0	0	1	1	0	3	1	0	0	0	1	1	0	0	0	0	0	0
	2001	6	3	0	1	0	0	0	0	1	0	3	1	0	0	0	1	1	0	0	0	0	0	0
	2002	6	3	0	1	0	0	0	0	1	0	3	1	0	0	0	1	1	0	0	0	0	0	0
	2003	5	3	0	1	0	0	0	0	1	0	2	0	0	0	0	1	0	0	0	0	0	0	0
	2004	5	3	0	1	0	0	0	0	1	0	2	0	0	0	0	1	1	0	0	0	0	0	0
	2005	5	2	0	1	0	0	0	0	1	0	3	1	0	0	0	1	1	0	0	0	0	0	0
	2006	4	2	0	1	0	0	0	0	1	0	2	0	0	0	0	1	1	0	0	0	0	0	0
	2007	4	2	0	1	0	0	0	0	1	0	2	0	0	0	0	1	1	0	0	0	0	0	0
	2008	4	2	0	1	0	0	0	0	1	0	2	0	0	0	0	1	1	0	0	0	0	0	0
	2009	4	2	0	1	0	0	0	0	1	0	2	0	0	0	0	1	1	0	0	0	0	0	0
	2010	4	2	0	1	0	0	0	0	1	0	2	0	0	0	0	1	1	0	0	0	0	0	0
	2011	4	2	0	1	0	0	0	0	1	0	2	0	0	0	0	1	1	0	0	0	0	0	0
	2012	3	2	0	1	0	0	0	0	1	0	1	0	0	0	0	0	0	0	0	0	0	0	0
	2013	3	2	0	1	0	0	0	0	1	0	1	0	0	0	0	0	0	0	0	0	0	0	0
	2014	3	2	0	1	0	0	0	0	1	0	1	0	0	0	0	0	0	0	0	0	0	0	0

U5D[1]: total number of under 5 death; NEO[2]: total number of neonatal death; PNE[3]: pneumonia; PRE[4]: preterm; INT[5]: intrapartum related events; SEP[6]: sepsis/meningitis; TET[7]: tetanus; OTH[8]: other disorders; CON[9]: congenital; DIA[10]: diarrea; POS[11]: total number of post neonatal death; MEN[12]: meningitis; MEA[13]: measles; INJ[14]: injury; MAL[15]: malaria; PER[16]: pertussis

						0-28 days												1-59 months						
	Year	U5D[1]	NEO[2]	PNE[3]	PRE[4]	INT[5]	SEP[6]	TET[7]	OTH[8]	CON[9]	DIA[10]	POS[11]	PNE[3]	PRE[4]	INT[5]	MEN[12]	OTH[8]	CON[9]	DIA[10]	MEA[13]	INJ[14]	MAL[15]	AIDS	PER[16]
	2015	3	2	0	1	0	0	0	0	1	0	1	0	0	0	0	0	0	0	0	0	0	0	0
Fiji	2000	484	271	11	100	42	30	2	34	52	0	213	42	5	3	10	46	38	23	9	34	0	1	3
	2001	466	264	10	97	41	29	2	33	51	0	202	41	5	3	9	46	37	21	4	33	0	1	3
	2002	474	259	10	96	40	29	1	32	50	0	215	39	5	3	9	45	37	19	21	33	0	1	3
	2003	467	253	10	94	39	29	1	31	50	0	214	39	5	3	9	46	37	18	19	34	0	1	3
	2004	454	248	10	93	38	28	1	30	49	0	206	40	6	3	9	48	39	18	5	36	0	1	3
	2005	456	242	10	91	37	27	1	29	48	0	214	41	6	3	9	50	41	18	5	38	0	1	3
	2006	461	228	10	86	34	25	1	26	45	0	233	44	7	3	10	54	44	18	7	42	0	1	3
	2007	457	214	10	82	32	23	1	24	42	0	243	46	7	4	10	58	47	19	1	47	0	1	3
	2008	460	207	10	79	31	22	1	23	41	0	253	47	8	4	11	61	49	19	1	50	0	1	3
	2009	460	201	10	77	30	21	1	22	40	0	259	48	8	4	11	64	49	19	0	52	0	1	3
	2010	455	197	10	76	29	21	1	21	39	0	258	47	8	4	10	64	49	19	0	53	0	1	3
	2011	447	194	9	75	29	20	1	20	39	0	253	46	8	4	10	63	49	18	0	52	0	1	3
	2012	435	188	9	73	28	20	1	20	38	0	247	44	8	4	10	61	48	18	0	52	0	0	3
	2013	419	179	9	69	26	19	1	18	36	0	240	43	8	4	9	60	47	17	0	50	0	0	3
	2014	404	171	8	66	25	18	1	18	35	0	233	41	8	4	9	58	45	16	0	49	0	0	3
	2015	388	164	8	64	24	17	1	17	34	0	224	39	8	4	8	56	44	16	0	47	0	0	3
Kiribati	2000	181	75	5	24	22	10	1	6	7	0	106	32	3	3	6	24	7	22	0	10	0	0	0
	2001	167	68	4	22	19	9	1	5	7	0	99	30	2	3	5	22	7	20	0	9	0	0	0
	2002	150	59	4	19	17	8	1	4	6	0	91	28	2	3	5	21	6	18	0	9	0	0	0
	2003	133	51	3	17	14	7	1	4	5	0	82	25	2	2	4	19	6	16	1	8	0	0	0
	2004	122	46	3	15	13	7	0	3	5	0	76	23	2	2	4	18	5	14	1	7	0	0	0
	2005	124	46	3	15	13	7	0	3	5	0	78	24	2	2	4	18	6	14	0	8	0	0	0
	2006	135	52	3	17	14	8	0	4	5	0	83	25	2	2	4	19	6	14	2	8	0	0	0
	2007	156	64	4	21	17	9	1	5	7	0	92	29	2	3	5	22	7	16	0	9	0	0	0
	2008	182	76	5	25	21	11	1	6	8	0	106	28	3	3	4	28	9	20	0	11	0	0	0
	2009	203	87	5	28	24	13	1	7	9	0	116	31	3	4	4	31	9	21	0	13	0	0	0
	2010	214	93	6	30	25	14	1	8	10	0	121	32	3	4	4	32	10	21	0	13	0	0	0
	2011	214	91	6	29	24	14	1	7	10	0	123	32	4	4	4	33	10	21	0	14	0	0	0
	2012	204	86	5	27	23	13	1	7	9	0	118	31	3	4	4	32	10	20	0	13	0	0	0
	2013	188	79	5	25	21	12	0	7	9	0	109	26	3	4	3	31	9	19	0	13	0	0	0
	2014	173	72	4	23	19	11	0	6	8	0	101	25	3	4	3	29	9	17	0	12	0	0	0
	2015	162	67	4	21	18	10	0	6	8	0	95	23	3	3	3	28	8	15	0	12	0	0	0
Marshall Islands	2000	88	41	3	15	10	5	0	3	6	0	47	13	2	1	2	11	4	9	0	5	0	0	0
	2001	87	41	2	15	10	5	0	3	6	0	46	13	2	1	2	11	4	9	0	5	0	0	0
	2002	85	39	2	14	9	5	0	3	5	0	46	12	2	1	1	12	4	9	0	5	0	0	0
	2003	84	38	2	14	9	5	0	2	5	0	46	11	2	1	1	11	4	8	3	5	0	0	0
	2004	79	36	2	13	8	5	0	2	5	0	43	12	2	1	2	11	4	8	0	5	0	0	0
	2005	76	35	2	13	8	5	0	2	5	0	41	11	2	1	1	10	4	7	0	5	0	0	0
	2006	73	33	2	12	7	5	0	2	4	0	40	11	2	1	1	10	4	7	0	5	0	0	0
	2007	70	31	2	11	7	4	0	2	4	0	39	10	2	1	1	10	4	7	0	5	0	0	0
	2008	67	30	2	11	7	4	0	2	4	0	37	9	1	1	1	10	3	6	0	5	0	0	0
	2009	64	29	2	11	6	4	0	2	4	0	35	8	1	1	1	10	3	5	0	5	0	0	0
	2010	62	28	2	10	6	4	0	2	4	0	34	8	1	1	1	9	3	5	0	4	0	0	0
	2011	60	27	2	10	6	4	0	2	4	0	33	8	1	1	1	9	3	5	0	4	0	0	0
	2012	58	26	1	10	6	4	0	2	4	0	32	8	1	1	1	9	3	5	0	4	0	0	0
	2013	56	25	1	9	5	4	0	2	3	0	31	8	1	1	1	9	3	4	0	4	0	0	0
	2014	53	24	1	9	5	3	0	2	3	0	29	8	1	1	1	8	3	4	0	4	0	0	0
	2015	49	23	1	8	5	3	0	2	3	0	26	7	1	1	1	7	2	3	0	3	0	0	0
Micronesia (Federated States of)	2000	175	84	5	31	22	8	1	6	10	1	91	24	3	2	3	22	6	18	1	10	0	0	1
	2001	167	80	5	29	21	8	1	6	10	1	87	23	3	2	3	21	6	17	1	10	0	0	1
	2002	157	76	5	28	19	8	1	6	9	1	81	22	3	2	3	20	6	15	1	9	0	0	1
	2003	147	71	4	26	18	8	1	5	9	1	76	21	3	2	3	19	5	14	1	9	0	0	0
	2004	138	67	4	24	16	8	1	5	8	1	71	19	2	2	3	17	5	13	1	8	0	0	1
	2005	129	63	4	23	15	7	0	5	8	0	66	18	2	2	2	17	5	11	1	8	0	0	0
	2006	121	60	4	22	14	7	0	4	8	0	61	17	2	1	2	16	4	10	1	7	0	0	0
	2007	115	58	4	21	14	7	0	4	8	0	57	15	2	1	2	15	4	9	1	7	0	0	0
	2008	109	55	3	20	13	7	0	4	7	0	54	15	2	1	2	14	4	9	1	7	0	0	0
	2009	105	54	3	19	13	7	0	4	7	0	51	14	2	1	2	14	4	8	1	6	0	0	0
	2010	101	52	3	18	12	7	0	4	7	0	49	12	2	1	1	14	4	7	1	7	0	0	0
	2011	96	51	3	18	12	7	0	4	7	0	45	11	2	1	1	13	4	7	0	6	0	0	0
	2012	93	49	3	17	11	7	0	4	7	0	44	11	2	1	1	13	4	6	0	6	0	0	0
	2013	90	48	3	16	11	6	0	4	7	0	42	10	2	1	1	12	3	6	0	6	0	0	0
	2014	87	47	3	16	11	6	0	4	7	0	40	10	2	1	1	12	3	5	0	6	0	0	0
	2015	85	46	3	16	11	6	0	4	7	0	39	10	2	1	1	12	3	5	0	6	0	0	0
Nauru	2000	7	4	0	1	1	0	0	0	1	0	3	1	0	0	0	1	0	1	0	0	0	0	0
	2001	7	4	0	1	1	0	0	0	1	0	3	1	0	0	0	1	0	1	0	0	0	0	0
	2002	7	4	0	1	1	0	0	0	1	0	3	1	0	0	0	1	0	1	0	0	0	0	0
	2003	7	4	0	1	1	1	0	0	1	0	3	1	0	0	0	1	0	1	0	0	0	0	0
	2004	7	4	0	1	1	1	0	0	1	0	3	1	0	0	0	1	0	1	0	0	0	0	0
	2005	7	4	0	1	1	1	0	0	1	0	3	1	0	0	0	1	0	1	0	0	0	0	0
	2006	7	4	0	1	1	1	0	0	1	0	3	1	0	0	0	1	0	1	0	0	0	0	0
	2007	7	4	0	1	1	1	0	0	1	0	3	1	0	0	0	1	0	1	0	0	0	0	0
	2008	7	4	0	1	1	1	0	0	1	0	3	1	0	0	0	1	0	0	0	0	0	0	0
	2009	6	4	0	1	1	1	0	0	1	0	2	1	0	0	0	1	0	0	0	0	0	0	0
	2010	6	4	0	1	1	1	0	0	1	0	2	1	0	0	0	1	0	0	0	0	0	0	0
	2011	6	4	0	1	1	1	0	0	1	0	2	1	0	0	0	1	0	0	0	0	0	0	0
	2012	6	4	0	1	1	1	0	0	1	0	2	1	0	0	0	1	0	0	0	0	0	0	0
	2013	6	4	0	1	1	1	0	0	1	0	2	1	0	0	0	1	0	0	0	0	0	0	0
	2014	5	3	0	1	1	0	0	0	0	0	2	1	0	0	0	1	0	0	0	0	0	0	0
	2015	5	3	0	1	1	0	0	0	0	0	2	1	0	0	0	1	0	0	0	0	0	0	0
Niue	2000	1	0	0	0	0	0	0	0	0	0	1	0	0	0	0	0	0	0	0	0	0	0	0
	2001	1	0	0	0	0	0	0	0	0	0	1	0	0	0	0	0	0	0	0	0	0	0	0
	2002	1	0	0	0	0	0	0	0	0	0	1	0	0	0	0	0	0	0	0	0	0	0	0
	2003	1	0	0	0	0	0	0	0	0	0	1	0	0	0	0	0	0	0	0	0	0	0	0
	2004	1	1	0	0	0	0	0	0	0	0	0	0	0	0	0	0	0	0	0	0	0	0	0
	2005	1	1	0	0	0	0	0	0	0	0	0	0	0	0	0	0	0	0	0	0	0	0	0
	2006	1	0	0	0	0	0	0	0	0	0	1	0	0	0	0	0	0	0	0	0	0	0	0
	2007	1	0	0	0	0	0	0	0	0	0	1	0	0	0	0	0	0	0	0	0	0	0	0
	2008	1	0	0	0	0	0	0	0	0	0	1	0	0	0	0	0	0	0	0	0	0	0	0
	2009	1	0	0	0	0	0	0	0	0	0	1	0	0	0	0	0	0	0	0	0	0	0	0
	2010	1	0	0	0	0	0	0	0	0	0	1	0	0	0	0	0	0	0	0	0	0	0	0
	2011	1	0	0	0	0	0	0	0	0	0	1	0	0	0	0	0	0	0	0	0	0	0	0
	2012	1	0	0	0	0	0	0	0	0	0	1	0	0	0	0	0	0	0	0	0	0	0	0
	2013	1	0	0	0	0	0	0	0	0	0	1	0	0	0	0	0	0	0	0	0	0	0	0
	2014	1	0	0	0	0	0	0	0	0	0	1	0	0	0	0	0	0	0	0	0	0	0	0
	2015	1	0	0	0	0	0	0	0	0	0	1	0	0	0	0	0	0	0	0	0	0	0	0
Palau	2000	7	4	0	2	1	0	0	0	1	0	3	1	0	0	0	1	0	0	0	1	0	0	0
	2001	7	4	0	2	1	0	0	0	1	0	3	1	0	0	0	1	0	0	0	1	0	0	0
	2002	8	4	0	2	1	0	0	0	1	0	4	1	0	0	0	1	1	0	0	1	0	0	0
	2003	8	5	0	2	1	1	0	0	1	0	3	0	0	0	0	1	0	0	0	1	0	0	0
	2004	9	5	0	2	1	1	0	0	1	0	4	1	0	0	0	1	1	0	0	1	0	0	0
	2005	9	5	0	2	1	1	0	0	1	0	4	1	0	0	0	1	1	0	0	1	0	0	0
	2006	8	4	0	2	1	0	0	0	1	0	4	1	0	0	0	1	1	0	0	1	0	0	0
	2007	7	4	0	2	1	0	0	0	1	0	3	0	0	0	0	1	1	0	0	1	0	0	0
	2008	6	3	0	1	0	0	0	0	1	0	3	0	0	0	0	1	0	0	0	1	0	0	0
	2009	5	3	0	1	0	0	0	0	1	0	2	0	0	0	0	1	0	0	0	1	0	0	0

U5D[1]: total number of under 5 death; NEO[2]: total number of neonatal death; PNE[3]: pneumonia; PRE[4]: preterm; INT[5]: intrapartum related events; SEP[6]: sepsis/meningitis; TET[7]: tetanus; OTH[8]: other disorders; CON[9]: congenital; DIA[10]: diarrea; POS[11]: total number of post neonatal death; MEN[12]: meningitis; MEA[13]: measles; INJ[14]: injury; MAL[15]: malaria; PER[16]: pertussis

		0-28 days										1-59 months												
	Year	U5D[1]	NEO[2]	PNE[3]	PRE[4]	INT[5]	SEP[6]	TET[7]	OTH[8]	CON[9]	DIA[10]	POS[11]	PNE[3]	PRE[4]	INT[5]	MEN[12]	OTH[8]	CON[9]	DIA[10]	MEA[13]	INJ[14]	MAL[15]	AIDS	PER[16]
	2010	6	2	0	1	0	0	0	0	0	0	4	0	0	0	0	1	0	0	1	1	0	0	0
	2011	5	2	0	1	0	0	0	0	0	0	3	0	0	0	0	1	1	0	0	1	0	0	0
	2012	5	2	0	1	0	0	0	0	0	0	3	0	0	0	0	1	1	0	0	1	0	0	0
	2013	5	3	0	1	0	0	0	0	1	0	2	0	0	0	0	0	0	0	0	1	0	0	0
	2014	5	3	0	1	0	0	0	0	1	0	2	0	0	0	0	0	0	0	0	1	0	0	0
	2015	5	3	0	1	0	0	0	0	1	0	2	0	0	0	0	0	0	0	0	1	0	0	0
Papua New Guinea	2000	14847	5618	375	1900	1593	732	108	407	453	51	9229	1937	176	109	364	1097	276	1297	1113	563	1921	208	167
	2001	15229	5627	376	1867	1609	744	108	411	463	50	9602	2018	183	115	382	1149	289	1310	1340	589	1819	234	174
	2002	15713	5628	376	1840	1617	756	106	413	469	50	10085	2164	196	124	412	1239	313	1360	1753	634	1452	254	184
	2003	14954	5616	376	1818	1617	762	104	413	475	49	9338	2097	190	120	401	1213	304	1277	1022	618	1655	269	172
	2004	14392	5594	374	1803	1611	764	101	413	480	48	8798	1918	175	110	367	1111	280	1130	489	567	2210	279	161
	2005	14185	5578	372	1781	1610	768	97	416	486	47	8607	2154	196	125	411	1251	317	1228	296	640	1527	281	181
	2006	13908	5598	373	1767	1620	778	94	422	498	47	8310	2110	192	122	400	1230	312	1163	56	632	1636	279	177
	2007	13937	5621	374	1760	1628	787	90	427	510	46	8316	2152	197	125	404	1251	319	1146	93	649	1518	273	188
	2008	13883	5649	373	1765	1628	798	86	430	524	44	8234	2174	199	126	404	1256	324	1116	120	662	1396	256	201
	2009	13823	5663	372	1779	1615	811	82	426	534	43	8160	1705	189	119	271	1211	308	1018	178	633	2123	230	174
	2010	13529	5606	364	1764	1583	818	76	421	538	41	7923	1909	209	131	306	1325	340	1079	77	702	1418	227	202
	2011	13326	5514	356	1734	1542	821	71	413	539	39	7812	1951	217	136	304	1380	354	1080	126	737	1100	215	211
	2012	13024	5418	348	1700	1503	821	65	404	540	37	7606	1726	194	121	265	1231	317	927	141	663	1635	196	190
	2013	12616	5290	337	1668	1454	807	60	392	537	35	7326	1666	190	119	250	1216	312	878	42	656	1643	169	184
	2014	12285	5189	328	1645	1415	791	57	382	538	33	7096	1683	189	118	256	1209	311	838	67	658	1449	137	182
	2015	11963	5127	322	1637	1388	775	55	374	544	32	6836	1620	182	114	246	1169	300	778	64	640	1415	132	175
Samoa	2000	111	59	3	26	7	6	0	3	14	0	52	11	1	1	2	13	11	5	0	7	0	0	1
	2001	107	58	3	25	7	6	0	3	14	0	49	10	1	1	2	12	10	5	0	7	0	0	1
	2002	104	57	3	25	7	6	0	3	13	0	47	9	1	1	1	12	10	5	0	7	0	0	1
	2003	103	56	3	24	7	6	0	3	13	0	47	9	1	1	1	12	10	5	0	6	0	0	1
	2004	103	56	3	24	7	6	0	3	13	0	47	8	1	1	1	13	9	7	0	6	0	0	1
	2005	103	56	3	24	7	6	0	3	13	0	47	8	1	1	1	13	9	8	0	6	0	0	1
	2006	103	57	3	24	7	6	0	4	14	0	46	7	1	1	1	13	8	8	0	6	0	0	1
	2007	103	57	3	24	7	6	0	4	14	0	46	7	1	1	1	13	9	8	0	6	0	0	1
	2008	104	57	3	24	7	6	0	4	14	0	47	7	1	1	1	15	9	7	0	5	0	0	1
	2009	133	57	3	24	7	6	0	4	14	0	76	7	1	1	1	15	10	6	0	34	0	0	1
	2010	103	57	3	24	7	6	0	4	14	0	46	7	2	1	1	14	10	5	0	6	0	0	1
	2011	100	55	3	23	7	6	0	4	13	0	45	7	2	1	1	13	10	4	0	6	0	0	1
	2012	96	52	3	21	6	6	0	4	12	0	44	7	2	1	1	12	10	3	0	6	0	0	1
	2013	90	49	2	20	6	5	0	4	12	0	41	6	2	1	1	12	9	3	0	6	0	0	1
	2014	85	45	2	19	5	5	0	3	11	0	40	6	2	1	1	11	9	3	0	6	0	0	1
	2015	80	43	2	18	5	5	0	3	10	0	37	6	2	1	1	10	9	2	0	5	0	0	1
Solomon Islands	2000	455	199	12	58	52	30	1	15	32	1	256	63	6	4	13	33	11	34	0	24	55	0	12
	2001	463	202	12	59	52	30	1	15	32	1	261	63	6	4	13	33	11	33	0	24	62	0	12
	2002	474	206	12	60	54	30	1	16	33	1	268	66	6	4	14	34	11	33	0	25	60	0	13
	2003	488	214	12	62	56	31	1	16	35	1	274	71	7	5	15	38	12	35	0	27	50	0	13
	2004	504	220	13	64	57	32	1	17	35	1	284	64	6	4	14	35	11	31	0	25	84	0	12
	2005	518	226	13	66	58	33	1	17	36	1	292	73	7	5	15	39	13	34	0	28	64	0	14
	2006	533	233	14	68	60	34	1	18	37	1	300	76	7	5	16	42	14	35	0	29	63	0	13
	2007	542	237	14	69	61	35	1	18	38	1	305	89	9	6	18	48	16	40	0	34	28	0	17
	2008	547	238	14	69	61	36	1	18	38	1	309	82	10	7	13	58	19	46	0	41	14	0	19
	2009	545	235	13	68	60	36	1	18	38	1	310	83	10	7	13	60	20	45	0	42	10	0	20
	2010	541	234	13	68	59	36	1	18	38	1	307	82	10	7	13	59	20	43	0	41	12	0	20
	2011	531	229	13	67	57	35	1	18	38	1	302	80	10	7	12	63	20	42	0	42	7	0	18
	2012	519	223	12	65	56	34	1	17	37	1	296	79	10	7	12	62	20	41	0	42	6	0	18
	2013	504	217	12	64	54	32	1	17	37	1	287	78	10	7	12	60	19	38	0	41	6	0	17
	2014	485	209	12	62	52	31	1	16	36	1	276	76	10	7	12	57	19	35	0	39	5	0	17
	2015	470	203	11	60	50	30	1	15	35	1	267	74	9	6	11	56	18	33	0	38	4	0	16
Tonga	2000	45	19	1	8	2	2	0	1	5	0	26	5	1	0	1	7	6	2	0	4	0	0	0
	2001	45	19	1	8	2	2	0	1	5	0	26	5	1	0	1	7	6	2	0	4	0	0	0
	2002	45	19	1	8	2	2	0	1	5	0	26	5	1	0	1	7	6	2	0	4	0	0	0
	2003	46	20	1	8	2	2	0	1	5	0	26	4	1	0	1	7	6	2	0	4	0	0	0
	2004	47	20	1	8	2	2	0	1	5	0	27	5	1	0	1	8	6	2	0	4	0	0	0
	2005	48	21	1	9	2	2	0	1	5	0	27	4	1	1	1	8	7	2	0	4	0	0	0
	2006	49	21	1	9	2	2	0	1	5	0	28	4	1	1	1	8	7	2	0	4	0	0	0
	2007	49	21	1	9	2	2	0	1	5	0	28	4	1	1	1	8	7	2	0	4	0	0	0
	2008	49	21	1	9	2	2	0	1	5	0	28	4	1	1	1	8	7	2	0	4	0	0	0
	2009	49	20	1	8	2	2	0	1	5	0	29	4	1	1	1	8	7	2	0	4	0	0	0
	2010	49	20	1	8	2	2	0	1	5	0	29	4	1	1	1	8	7	2	0	5	0	0	0
	2011	49	20	1	8	2	2	0	1	5	0	29	4	1	1	1	8	7	2	0	5	0	0	0
	2012	47	20	1	8	2	2	0	1	5	0	27	4	1	1	1	8	6	2	0	4	0	0	0
	2013	46	19	1	8	2	2	0	1	5	0	27	4	1	1	1	8	6	2	0	4	0	0	0
	2014	44	18	1	7	2	2	0	1	4	0	26	4	1	1	1	8	6	2	0	4	0	0	0
	2015	43	17	1	7	2	2	0	1	4	0	26	4	1	1	1	8	6	1	0	4	0	0	0
Tuvalu	2000	12	7	1	3	1	1	0	1	1	0	5	2	0	0	0	1	1	1	0	1	0	0	0
	2001	11	7	1	3	1	1	0	1	1	0	4	1	0	0	0	1	1	1	0	1	0	0	0
	2002	11	7	1	3	1	1	0	1	1	0	4	1	0	0	0	1	1	0	0	1	0	0	0
	2003	10	6	0	3	1	0	0	0	1	0	4	1	0	0	0	1	1	0	0	1	0	0	0
	2004	9	6	0	3	1	0	0	0	1	0	3	1	0	0	0	1	0	0	0	0	0	0	0
	2005	8	5	0	2	1	0	0	0	1	0	3	1	0	0	0	1	0	0	0	0	0	0	0
	2006	8	5	0	2	1	0	0	0	1	0	3	1	0	0	0	1	0	0	0	0	0	0	0
	2007	7	5	0	2	1	0	0	0	1	0	2	1	0	0	0	0	0	0	0	0	0	0	0
	2008	7	5	0	2	1	0	0	0	1	0	2	1	0	0	0	0	0	0	0	0	0	0	0
	2009	7	4	0	2	1	0	0	0	1	0	3	1	0	0	0	1	0	0	0	0	0	0	0
	2010	6	4	0	2	1	0	0	0	1	0	2	1	0	0	0	0	0	0	0	0	0	0	0
	2011	6	4	0	2	1	0	0	0	1	0	2	1	0	0	0	0	0	0	0	0	0	0	0
	2012	6	4	0	2	1	0	0	0	1	0	2	1	0	0	0	0	0	0	0	0	0	0	0
	2013	6	4	0	2	1	0	0	0	1	0	2	1	0	0	0	0	0	0	0	0	0	0	0
	2014	6	4	0	2	1	0	0	0	1	0	2	1	0	0	0	0	0	0	0	0	0	0	0
	2015	6	4	0	2	1	0	0	0	1	0	2	0	0	0	0	0	0	0	0	0	0	0	0
Vanuatu	2000	170	73	5	29	14	5	1	5	15	0	97	22	0	0	2	9	12	26	0	8	13	0	3
	2001	167	72	5	29	14	5	1	5	14	0	95	22	1	0	2	9	12	27	1	8	9	0	4
	2002	165	70	5	28	13	5	1	5	14	0	95	17	0	0	2	7	10	22	2	7	25	0	3
	2003	168	69	5	27	13	4	2	5	14	0	99	14	0	0	2	6	8	18	4	6	36	0	3
	2004	170	71	5	28	13	5	1	5	14	0	99	15	0	0	2	7	9	20	6	6	30	0	3
	2005	170	73	5	29	13	6	1	5	14	0	97	20	1	0	2	9	12	26	2	8	13	0	4
	2006	179	77	5	31	13	6	1	6	15	0	102	21	1	0	2	10	13	28	2	9	11	0	4
	2007	191	83	6	33	14	6	2	6	16	0	108	23	1	1	2	14	14	30	0	10	6	0	7
	2008	202	88	6	35	15	7	2	6	17	0	114	24	1	1	3	16	15	31	0	11	5	0	8
	2009	212	93	7	37	16	7	2	7	17	0	119	25	1	1	3	14	16	32	1	12	6	0	8
	2010	219	95	7	38	16	8	2	7	18	0	124	26	1	1	3	16	17	33	0	13	7	0	8
	2011	218	94	7	38	16	8	2	7	17	0	124	24	2	1	3	24	17	30	1	12	3	0	8
	2012	213	91	7	37	15	7	2	7	17	0	122	23	2	1	3	24	16	30	1	12	3	0	7
	2013	204	86	6	35	14	7	2	6	16	0	118	22	2	1	3	24	16	29	1	11	2	0	7
	2014	196	82	6	33	13	7	2	6	15	0	114	21	2	1	3	23	15	28	2	11	2	0	6
	2015	189	79	6	32	13	6	2	6	15	0	110	21	2	1	3	22	15	26	2	11	2	0	6
South-eastern Asia	2000	548491	231292	15787	78034	58568	30191	3108	17076	26842	1686	317199	90865	6532	5065	14596	56671	15261	61919	25503	28756	5379	2027	4627
	2001	525715	225157	15188	76061	56764	29151	2808	16758	26849	1578	300558	87159	6335	4929	13869	54829	14915	56967	23074	28113	3536	2139	4694
	2002	505179	220427	14705	74609	55145	28496	2530	16490	26977	1475	284752	83899	6157	4807	13146	53301	14797	52688	18196	27545	3198	2174	4844
	2003	490281	217231	14368	73693	53787	28081	2305	16282	27332	1384	273050	80496	5969	4664	12385	51836	14720	48700	17011	26888	3328	2208	4845
	2004	505440	214268	14045	72818	52423	27815	2103	16048	27718	1299	291172	77356	5820	4553	11712	50480	14580	45232	18079	52937	3291	2311	4820

U5D[1]: total number of under 5 death; NEO[2]: total number of neonatal death; PNE[3]: pneumonia; PRE[4]: preterm; INT[5]: intrapartum related events; SEP[6]: sepsis/meningitis; TET[7]: tetanus; OTH[8]: other disorders; CON[9]: congenital; DIA[10]: diarrea; POS[11]: total number of post neonatal death; MEN[12]: meningitis; MEA[13]: measles; INJ[14]: injury; MAL[15]: malaria; PER[16]: pertussis

Webappendix 6. Estimated numbers of deaths by cause in neonates, children aged 1-59 months at the global, regional and country levels in 2000-2015

		0-28 days										1-59 months												
	Year	U5D[1]	NEO[2]	PNE[3]	PRE[4]	INT[5]	SEP[6]	TET[7]	OTH[8]	CON[9]	DIA[10]	POS[11]	PNE[3]	PRE[4]	INT[5]	MEN[12]	OTH[8]	CON[9]	DIA[10]	MEA[13]	INJ[14]	MAL[15]	AIDS	PER[16]
	2005	462502	209682	13624	71418	50743	27195	1926	15732	27830	1213	252820	73086	5561	4351	11993	48141	14191	41451	17823	25195	3772	2405	4852
	2006	447870	203851	13129	69666	48808	26316	1762	15283	27760	1128	244019	70129	5422	4188	10272	46625	13909	38525	19549	24578	3449	2513	4859
	2007	424566	197715	12604	67797	46871	25290	1626	14823	27656	1048	226851	67082	5270	4026	9672	44888	13464	35601	13976	23883	1522	2593	4873
	2008	425442	191855	12097	65956	44986	24434	1496	14349	27567	971	233587	64582	5147	3901	9282	43527	12986	33096	6110	44403	3253	2565	4736
	2009	384855	185515	11544	63692	43018	23832	1336	13880	27324	891	199340	59609	4863	3611	8381	40309	12329	29399	8509	21901	3159	2509	4763
	2010	370554	180386	11054	61901	41256	23542	1183	13452	27179	820	190168	55963	4890	3534	7739	39498	12050	27200	7488	21401	3057	2485	4863
	2011	362784	176771	10689	60734	39862	23377	1060	13127	27160	762	186013	53748	4909	3478	7318	38504	11916	25364	9295	21042	3052	2522	4865
	2012	352704	174571	10417	60194	38891	23139	982	12879	27347	722	178133	51985	4874	3421	6973	37668	11776	23706	6205	20725	3492	2510	4799
	2013	347410	172209	10148	59707	37938	22672	926	12641	27492	685	175201	48850	4983	3514	5965	39201	11929	23302	5982	21569	2699	2468	4740
	2014	340654	169024	9844	58861	36906	22056	874	12360	27471	652	171630	47015	4979	3502	5643	38803	11861	22462	6088	21401	2822	2423	4631
	2015	330950	164608	9486	57572	35631	21288	823	12001	27187	620	166342	45609	4916	3432	5444	37892	11656	21084	5741	20958	2762	2350	4497
**Brunei Darussalam**	2000	64	33	0	13	5	1	1	4	9	0	31	3	1	0	1	9	8	0	0	6	0	0	1
	2001	62	32	0	13	4	1	1	4	9	0	30	3	1	0	1	9	8	0	0	6	0	0	1
	2002	60	32	0	13	4	1	1	4	9	0	28	2	1	1	1	8	8	0	0	5	0	0	1
	2003	59	32	0	13	4	1	1	4	9	0	27	2	1	0	1	8	7	0	0	5	0	0	1
	2004	58	31	0	12	4	0	1	4	9	0	27	2	1	0	1	8	7	0	0	5	0	0	1
	2005	57	31	0	12	4	0	1	4	9	0	26	2	1	0	1	8	7	0	0	5	0	0	1
	2006	56	30	0	12	4	0	1	4	9	0	26	2	1	0	1	8	7	0	0	5	0	0	1
	2007	54	28	0	11	4	0	1	3	8	0	26	2	1	0	1	8	7	0	0	5	0	0	1
	2008	53	27	0	11	4	0	1	3	8	0	26	2	1	0	1	8	7	0	0	5	0	0	1
	2009	53	27	0	11	4	0	1	3	8	0	26	2	1	0	1	8	7	0	0	5	0	0	1
	2010	55	27	0	11	4	1	0	3	8	0	28	2	1	1	1	8	7	0	0	6	0	0	1
	2011	58	28	0	11	4	1	0	3	8	0	30	2	1	1	1	9	8	0	0	6	0	0	1
	2012	63	29	0	12	4	1	0	3	8	0	34	3	2	1	1	10	9	0	0	7	0	0	1
	2013	69	31	0	13	4	1	0	4	9	0	38	3	2	1	1	12	10	1	0	8	0	0	1
	2014	74	32	0	13	4	1	0	4	9	0	42	3	2	1	1	13	11	1	0	9	0	0	1
	2015	77	33	0	14	4	1	0	4	9	0	44	4	2	1	1	14	11	1	0	10	0	0	1
**Cambodia**	2000	35775	11212	789	3616	3321	1438	393	798	685	173	24563	6634	519	292	1373	3833	577	5071	1357	1679	2257	648	323
	2001	31809	10581	737	3412	3125	1385	306	768	702	145	21228	6218	492	274	1265	3593	552	4463	900	1642	823	659	347
	2002	27975	10027	687	3226	2922	1372	228	742	733	119	17948	5473	437	241	1094	3135	494	3671	294	1517	580	637	376
	2003	25189	9673	649	3081	2755	1420	168	726	775	99	15516	4706	381	207	925	2673	434	2954	118	1367	773	602	378
	2004	23273	9376	613	2943	2601	1490	123	713	810	83	13897	4324	353	191	835	2460	406	2556	69	1305	462	561	375
	2005	21899	9148	584	2818	2498	1526	96	708	848	71	12751	4060	334	180	768	2291	389	2273	95	1266	223	471	401
	2006	20827	8970	561	2711	2424	1539	78	705	891	61	11857	3794	315	169	704	2139	370	2017	105	1217	224	398	405
	2007	19817	8806	541	2616	2355	1545	65	698	935	52	11011	3580	299	160	654	2029	354	1810	125	1180	92	330	399
	2008	19111	8592	517	2519	2262	1545	54	682	968	45	10519	3324	280	149	601	1907	333	1599	479	1125	88	263	371
	2009	18082	8298	488	2411	2145	1518	45	661	991	39	9784	3020	255	135	542	1734	305	1382	655	1050	141	207	358
	2010	16312	7914	456	2295	2004	1455	38	628	1003	34	8398	2294	258	137	319	1771	310	1328	263	1092	85	175	366
	2011	14809	7371	416	2139	1831	1353	31	583	988	30	7438	2049	233	123	280	1577	280	1136	170	1008	95	125	363
	2012	13245	6813	377	1981	1663	1239	26	536	964	26	6432	1808	207	109	242	1365	249	958	43	916	59	107	368
	2013	11980	6203	339	1805	1496	1110	22	486	922	23	5777	1652	188	99	222	1250	227	828	37	847	29	65	332
	2014	11022	5661	306	1651	1350	999	18	442	876	20	5361	1504	175	92	196	1172	212	734	78	799	34	59	305
	2015	10257	5282	282	1547	1245	920	16	410	845	18	4975	1398	163	86	181	1100	198	655	74	754	28	55	284
**Indonesia**	2000	234920	97002	6213	29645	26695	14304	692	7812	11138	504	137918	38913	2654	2685	5455	24852	4607	24944	18543	13101	1173	63	927
	2001	220654	93966	5947	28831	25633	13704	630	7549	11212	461	126688	36845	2514	2585	5147	23789	4461	22698	13973	12601	1045	98	933
	2002	211345	91461	5721	28236	24690	13231	580	7295	11283	425	119884	35214	2401	2509	4865	22972	4350	20873	12233	12215	1134	151	968
	2003	203614	89646	5543	27856	23908	12892	539	7079	11430	399	113968	33467	2285	2424	4560	22065	4235	19083	11723	11778	1135	229	984
	2004	225321	88245	5393	27587	23228	12652	502	6897	11610	377	137076	32073	2187	2357	4314	21352	4135	17598	12526	38108	1077	338	1010
	2005	192897	86012	5199	27048	22361	12258	464	6677	11651	355	106885	29833	2033	2225	3961	20021	3921	15763	14787	10790	2058	473	1020
	2006	187376	83548	5000	26534	21466	11699	433	6431	11647	338	103828	28437	1951	2132	3726	19222	3799	14475	16239	10417	1745	634	1051
	2007	173563	81232	4812	26068	20644	11090	405	6240	11649	324	92331	27320	1886	2057	3531	18566	3700	13401	9523	10131	283	816	1117
	2008	161609	79020	4629	25678	19792	10569	376	6035	11627	314	82589	25201	1750	1906	3212	17217	3460	11910	4531	9463	1792	1019	1128
	2009	157823	77428	4472	25401	19023	10418	341	5878	11590	305	80395	24039	1677	1824	3023	16515	3336	10956	5733	9130	1809	1201	1152
	2010	153388	75790	4306	25184	18141	10427	307	5624	11505	295	77598	23199	1624	1764	2882	16066	3242	10189	5392	8920	1807	1362	1150
	2011	152723	75354	4215	25353	17582	10614	281	5452	11568	289	77369	22751	1595	1736	2798	15915	3202	9638	6349	8845	1923	1495	1121
	2012	150629	75957	4198	25862	17372	10768	268	5370	11833	286	74672	22451	1580	1719	2738	15886	3191	9178	4003	8821	2458	1591	1057
	2013	151401	76305	4172	26248	17200	10740	255	5319	12089	281	75096	22724	1624	1767	2735	16422	3299	9080	3755	9115	1835	1668	1071
	2014	151192	75977	4115	26342	16930	10557	243	5244	12273	273	75215	21728	1638	1783	2526	16664	3349	8817	4567	9248	2054	1787	1053
	2015	147162	73921	3968	25817	16294	10146	227	5054	12157	259	73241	21197	1604	1746	2457	16381	3299	8313	4290	9099	2091	1740	1024
**Lao People's Democratic Republic**	2000	19470	6975	577	1789	2207	809	531	500	429	134	12495	3737	292	167	659	1718	329	2981	1228	909	262	8	204
	2001	18262	6701	552	1739	2126	777	477	481	425	124	11561	3653	287	164	636	1689	326	2798	794	905	92	12	204
	2002	17733	6515	533	1711	2072	752	437	468	427	115	11218	3530	279	160	606	1649	320	2597	911	890	64	16	198
	2003	18030	6366	519	1694	2029	728	402	456	431	107	11664	3428	272	156	580	1611	315	2423	1724	880	55	21	198
	2004	17964	6288	508	1691	2008	714	375	450	441	100	11676	3336	267	152	556	1576	311	2265	2071	871	43	26	201
	2005	16970	6251	501	1701	1999	703	350	447	455	94	10719	3253	262	149	533	1544	308	2123	1414	865	32	31	206
	2006	16311	6222	495	1719	1986	695	326	444	468	89	10089	3184	258	146	514	1519	305	1997	1020	861	41	33	211
	2007	16813	6261	492	1767	1983	695	306	446	485	86	10552	3117	254	142	496	1479	302	1878	1718	858	38	38	232
	2008	14921	6280	489	1790	1968	720	280	449	501	83	8641	3054	251	139	479	1458	299	1769	15	855	42	43	237
	2009	14710	6287	483	1786	1951	771	245	457	515	80	8423	2977	247	135	461	1425	295	1658	55	847	31	45	246
	2010	14430	6203	469	1726	1904	853	190	466	521	75	8227	2527	265	144	326	1563	318	1706	119	920	46	47	245
	2011	14039	6017	446	1648	1818	915	139	465	516	69	8022	2437	261	141	307	1547	314	1605	184	914	26	47	240
	2012	13286	5809	420	1580	1723	949	108	459	507	63	7477	2310	250	134	286	1496	301	1470	1	881	75	47	226
	2013	12638	5573	395	1517	1628	945	89	446	496	57	7065	2156	241	129	258	1457	292	1358	9	857	50	46	214
	2014	12107	5373	375	1471	1550	929	76	433	488	52	6734	1738	251	134	168	1554	306	1362	25	902	47	43	204
	2015	11613	5220	358	1441	1489	909	68	422	485	48	6393	1652	239	128	159	1499	294	1248	24	868	48	40	194
**Malaysia**	2000	5648	2937	73	1165	451	198	55	165	830	0	2711	411	92	61	81	652	753	169	0	338	9	47	98
	2001	5125	2668	65	1059	404	159	55	162	764	0	2457	355	89	59	70	578	705	148	0	309	12	37	97
	2002	4753	2448	59	969	366	129	57	162	706	0	2305	320	87	57	64	541	675	134	0	295	10	30	94
	2003	4425	2279	53	899	336	113	55	164	659	0	2146	287	84	54	58	503	638	118	0	279	5	27	93
	2004	4198	2160	48	847	314	108	49	169	623	0	2038	244	106	68	66	472	607	98	0	249	9	28	92
	2005	4023	2042	43	796	292	103	49	173	585	0	1981	228	110	70	62	459	591	89	0	244	8	31	89
	2006	3842	1965	39	760	276	105	47	180	558	0	1877	210	107	68	58	438	557	80	0	235	5	32	88
	2007	3651	1931	35	741	266	106	49	191	542	0	1720	188	100	63	51	399	508	70	0	218	4	32	87
	2008	3531	1907	34	726	258	110	47	201	530	0	1624	174	98	61	48	378	478	64	0	209	7	26	81
	2009	3466	1828	32	692	244	105	47	204	505	0	1638	174	99	61	47	387	480	63	0	214	6	26	80
	2010	3484	1839	31	694	242	107	47	213	506	0	1645	173	101	62	47	389	481	62	0	219	8	22	81
	2011	3539	1870	30	703	243	109	47	223	514	0	1669	174	106	64	47	402	491	62	0	224	4	13	82
	2012	3631	1940	29	728	250	113	49	238	534	0	1691	173	112	67	46	403	502	60	0	227	4	11	87
	2013	3752	2073	29	774	265	120	52	261	572	0	1679	169	112	67	44	401	501	58	0	225	3	11	86
	2014	3787	2134	28	793	271	123	54	275	590	0	1653	162	114	68	42	393	499	56	0	219	4	12	85
	2015	3793	2149	26	795	271	124	54	282	596	0	1644	159	115	69	40	391	499	54	0	216	4	12	85
**Myanmar**	2000	93214	43233	2794	15416	11355	6002	854	3185	3116	511	49981	15024	892	1039	3575	9926	1494	10656	1453	4287	1000	419	215
	2001	94949	42728	2748	15197	11273	5857	776	3205	3191	481	52221	14823	887	1027	3411	9846	1493	10128	4672	4281	957	472	222
	2002	90591	41992	2679	14810	11076	5849	685	3212	3236	444	48599	14543	877	1009	3240	9722	1480	9571	2178	4251	994	521	213
	2003	87798	41290	2615	14434	10896	5820	616	3212	3285	411	46508	14130	859	982	3050	9493	1457	8959	1608	4180	1017	564	209
	2004	85710	40279	2530	13958	10605	5781	553	3169	3308	376	45431	13591	834	946	2842	9186	1421	8298	2463	4071	980	599	199
	2005	80830	38917	2423	13354	10233	5664	490	3110	3302	340	41913	12985	802	903	2631	8816	1367	7637	1027	3937	976	626	205
	2006	77231	37166	2292	12613	9730	5548	427	3001	3253	302	40065	12301	767	854	2421	8392	1307	6965	1477	3778	958	650	194
	2007	73230	35155	2146	11837	9153	5358	374	2847	3172	266	38075	11559	727	800	2224	7923	1236	6303	2034	3596	824	659	190
	2008	94282	33460	2025	11223	8662	5175	332	2697	3112	236	60822	12544	796	864	2374	8657	1348	6590	652	25083	1109	613	192
	2009	62676	30887	1854	10343	7953	4805	291	2472	2964	204	31789	9979	638	685	1866	6886	1077	5049	656	3182	1022	561	188
	2010	58610	29111	1736	9743	7470	4513	259	2324	2885	181	29499	9346	600	638	1727	6468	1009	4556	467	3017	961	522	189
	2011	56214	27672	1642	9299	7078	4220	237	2196	2836	163	28542	8654	559	589	1579	5999	937	4064	1739	2828	897	502	194
	2012	53153	26583	1573	9010	6788	3933	224	2090	2815	150	26570	8110	527	550	1459	5637	880	3672	1604	2681	818	439	193
	2013	50750	25626	1511	8810	6514	3664	213	1977	2797	140	25124	6763	552	575	1000	5939	923	3700	1575	2832	709	372	185
	2014	48048	24851	1459	8630	6277	3475	201	1890	2789	130	23197	6461	528	550	948	5703	884	3409	922	2736	611	268	176
	2015	46284	24302	1418	8503	6091	3351	191	1827	2798	123	21982	6157	504	525	898	5462	845	3135	872	2636	527	254	167

U5D[1]: total number of under 5 death; NEO[2]: total number of neonatal death; PNE[3]: pneumonia; PRE[4]: preterm; INT[5]: intrapartum related events; SEP[6]: sepsis/meningitis; TET[7]: tetanus; OTH[8]: other disorders; CON[9]: congenital; DIA[10]: diarrea; POS[11]: total number of post neonatal death; MEN[12]: meningitis; MEA[13]: measles; INJ[14]: injury; MAL[15]: malaria; PER[16]: pertussis

			0-28 days										1-59 months											
	Year	U5D[1]	NEO[2]	PNE[3]	PRE[4]	INT[5]	SEP[6]	TET[7]	OTH[8]	CON[9]	DIA[10]	POS[11]	PNE[3]	PRE[4]	INT[5]	MEN[12]	OTH[8]	CON[9]	DIA[10]	MEA[13]	INJ[14]	MAL[15]	AIDS	PER[16]
Philippines	2000	90090	38356	2397	13454	9529	4591	284	2694	5061	347	51734	16838	1626	655	2399	8898	1925	10199	330	6164	53	13	2635
	2001	90089	38761	2403	13732	9537	4583	280	2714	5158	353	51328	16661	1615	655	2355	8809	1935	9757	676	6134	50	15	2668
	2002	89689	39119	2405	13986	9543	4560	277	2734	5258	358	50570	16446	1597	658	2306	8645	1942	9311	761	6090	53	17	2747
	2003	89178	39107	2385	14106	9441	4532	269	2718	5300	357	50071	16251	1584	658	2260	8579	1951	8895	1014	6053	69	19	2739
	2004	87545	38875	2351	14126	9273	4527	258	2682	5306	352	48670	15973	1559	656	2201	8493	1945	8452	618	5985	73	21	2695
	2005	85243	38394	2296	14059	9043	4498	245	2631	5276	345	46849	15210	1486	633	3173	8042	1874	7779	157	5733	68	24	2672
	2006	82659	37369	2212	13763	8712	4391	230	2548	5182	331	45290	15117	1488	627	2038	8056	1884	7473	149	5732	51	26	2648
	2007	79903	36134	2120	13340	8375	4243	214	2460	5070	313	43769	14655	1451	606	1951	7849	1843	7007	157	5587	51	28	2584
	2008	76696	34625	2020	12661	8032	4098	194	2374	4957	288	42071	14158	1411	584	1862	7666	1797	6543	88	5431	34	30	2469
	2009	73883	33269	1928	11879	7789	4009	172	2340	4894	257	40614	13696	1372	562	1780	7366	1749	6117	144	5287	27	32	2484
	2010	72083	32392	1861	11249	7659	3994	153	2354	4892	229	39691	13310	1344	545	1709	7072	1716	5765	413	5181	26	32	2579
	2011	70833	31711	1806	10766	7537	4009	138	2357	4892	206	39122	12824	1350	542	1603	7083	1722	5578	544	5213	12	34	2617
	2012	69255	31258	1765	10487	7411	4037	127	2337	4903	191	37997	12396	1342	541	1519	7046	1720	5298	253	5209	8	38	2627
	2013	68191	30684	1717	10237	7222	4044	117	2294	4873	179	37507	10732	1454	586	1067	7893	1871	5278	300	5667	8	41	2611
	2014	66959	30080	1669	10028	7025	3999	109	2243	4835	171	36879	10856	1404	566	1143	7595	1813	5192	211	5496	5	30	2568
	2015	65613	29601	1630	9887	6859	3940	103	2201	4816	165	36012	10623	1377	555	1115	7482	1785	4910	208	5415	4	29	2508
Singapore	2000	180	68	1	26	5	4	0	1	30	0	112	15	10	1	6	39	29	0	0	11	0	0	0
	2001	160	61	1	14	3	3	0	6	35	0	99	18	4	0	2	31	26	2	0	15	0	0	0
	2002	148	58	1	33	1	1	0	5	17	0	90	19	8	1	4	27	20	2	0	9	0	0	0
	2003	144	56	3	26	2	1	0	7	18	0	88	27	9	2	0	24	19	0	0	8	0	0	0
	2004	141	59	2	10	7	3	0	2	35	0	82	21	9	0	1	22	14	1	0	12	0	0	0
	2005	140	61	3	15	7	0	0	4	33	0	79	23	9	2	2	19	16	0	0	8	0	0	0
	2006	142	58	3	31	4	1	0	1	18	0	84	22	0	0	0	18	37	0	0	7	0	0	0
	2007	145	59	4	27	1	1	0	6	20	0	86	27	4	0	2	16	26	2	0	10	0	0	0
	2008	142	60	4	26	1	4	0	4	21	0	82	15	10	0	2	23	25	0	0	8	0	0	0
	2009	143	61	4	23	5	3	0	6	19	0	82	17	9	5	3	19	17	2	0	11	0	0	0
	2010	145	57	0	22	9	1	0	0	25	0	88	11	11	0	0	36	23	2	0	6	0	0	0
	2011	147	57	3	23	8	1	0	7	16	0	90	13	9	2	2	24	29	2	0	9	0	0	0
	2012	148	58	3	17	4	4	0	8	22	0	90	23	4	0	0	30	27	0	0	7	0	0	0
	2013	149	59	0	24	2	3	0	11	19	0	90	16	2	2	0	45	18	0	0	8	0	0	0
	2014	150	59	2	21	5	3	0	8	20	0	91	18	5	1	1	33	25	1	0	8	0	0	0
	2015	145	53	2	19	4	3	0	7	18	0	92	18	5	1	1	34	25	1	0	8	0	0	0
Thailand	2000	20439	11397	799	4567	1599	1148	79	851	2355	0	9042	1853	187	62	225	2026	1816	990	50	823	211	718	82
	2001	19257	10702	731	4256	1481	1067	76	832	2260	0	8555	1698	185	63	209	1943	1752	898	92	804	136	694	81
	2002	18216	10096	664	3989	1381	992	76	815	2180	0	8120	1575	186	64	196	1896	1714	816	80	793	114	606	81
	2003	17341	9617	607	3779	1300	938	68	803	2122	0	7724	1470	187	65	184	1908	1685	737	57	785	62	503	79
	2004	16439	9125	550	3577	1220	876	68	782	2054	0	7314	1356	187	66	172	1826	1640	659	48	766	71	446	79
	2005	15459	8591	493	3368	1134	805	68	747	1976	0	6868	1232	184	66	157	1752	1575	584	43	734	49	414	77
	2006	14544	8062	444	3174	1049	730	67	701	1898	0	6482	1128	182	65	145	1680	1519	524	43	708	34	381	74
	2007	13681	7584	406	3011	972	654	63	650	1828	0	6097	1034	179	64	134	1584	1457	469	47	684	29	343	72
	2008	12815	7180	374	2881	907	580	58	603	1776	0	5635	938	175	63	122	1417	1380	416	95	652	30	277	69
	2009	12073	6716	343	2722	839	504	57	552	1700	0	5357	880	176	63	114	1383	1344	383	74	641	21	211	66
	2010	11476	6372	317	2601	790	452	56	515	1641	0	5104	832	177	64	108	1360	1311	356	37	632	24	138	65
	2011	10939	6056	290	2481	747	410	55	490	1583	0	4883	783	177	64	101	1310	1271	331	37	612	13	121	63
	2012	10450	5771	265	2370	709	380	52	469	1526	0	4679	736	178	64	94	1230	1234	307	64	591	11	108	62
	2013	10058	5571	247	2290	684	358	51	457	1486	0	4487	697	178	64	88	1206	1198	288	36	569	11	92	59
	2014	9560	5279	223	2172	648	333	48	436	1420	0	4281	655	178	64	82	1155	1159	268	35	542	7	79	57
	2015	9173	4958	204	2040	608	304	45	414	1342	0	4215	639	178	64	79	1136	1150	260	34	535	6	78	56
Timor-Leste	2000	4318	1479	112	456	448	185	55	114	91	17	2839	866	68	35	141	418	69	677	0	217	311	0	37
	2001	3970	1351	104	397	417	182	46	101	89	15	2619	790	62	32	135	381	63	589	0	203	326	0	37
	2002	3647	1241	96	335	392	185	39	94	87	13	2406	771	60	31	136	370	62	548	0	203	185	0	40
	2003	3336	1139	88	290	363	182	31	89	85	12	2197	710	55	29	126	341	57	482	0	192	166	0	39
	2004	3027	1046	81	257	333	175	24	84	83	10	1981	505	39	21	87	242	41	326	0	140	550	0	29
	2005	2728	950	73	234	301	158	19	77	79	9	1778	513	40	21	83	244	41	316	0	146	340	1	33
	2006	2455	876	67	216	275	147	16	71	77	8	1579	435	34	18	67	203	35	256	0	127	370	1	33
	2007	2210	806	61	197	252	136	13	66	74	7	1404	442	34	18	64	203	36	248	0	131	188	1	38
	2008	2025	754	56	179	235	132	10	63	73	6	1271	413	32	17	57	186	34	223	0	126	142	1	41
	2009	1932	741	54	169	232	132	8	64	76	5	1191	406	32	17	54	176	33	209	0	126	89	2	48
	2010	1947	762	56	169	238	138	7	68	81	5	1185	406	32	17	53	177	33	201	0	128	90	2	48
	2011	2058	842	61	185	262	152	7	76	93	5	1216	426	33	18	54	186	35	203	0	136	72	2	51
	2012	2256	948	68	209	292	173	8	85	108	6	1308	470	37	20	60	213	38	216	0	152	48	4	50
	2013	2467	1058	75	234	322	196	8	94	123	6	1409	440	44	24	47	266	47	251	0	187	44	4	55
	2014	2612	1140	80	253	343	213	8	100	135	6	1472	465	46	25	51	279	49	252	0	197	47	4	57
	2015	2642	1162	81	259	346	219	8	102	141	6	1480	471	47	25	51	285	49	246	0	202	42	4	58
Viet Nam	2000	44373	18600	2032	7887	2955	1513	164	953	3097	0	25773	6571	190	66	679	4300	3654	6232	2541	1220	104	111	104
	2001	41378	17606	1899	7412	2760	1434	161	935	3004	0	23772	6095	200	70	637	4160	3594	5486	1966	1213	93	152	105
	2002	41021	17438	1859	7302	2699	1426	152	960	3041	0	23583	6007	224	77	634	4338	3732	5164	1740	1276	65	197	128
	2003	41167	18026	1906	7516	2752	1454	156	1024	3218	0	23141	6016	252	87	641	4631	3922	5048	768	1360	46	244	126
	2004	41764	18784	1970	7810	2830	1489	149	1098	3439	0	22980	5931	278	96	637	4843	4052	4977	283	1425	26	293	139
	2005	42257	19285	2008	8012	2872	1480	143	1155	3616	0	22972	5746	300	104	622	4945	4103	4885	300	1467	18	336	148
	2006	42427	19585	2017	8133	2882	1461	136	1197	3759	0	22842	5499	319	110	600	4950	4089	4736	515	1492	20	358	153
	2007	41499	19719	1986	8184	2867	1460	135	1215	3871	0	21780	5158	334	115	566	4832	3994	4411	373	1483	12	347	154
	2008	40256	19950	1949	8263	2864	1501	143	1237	3994	0	20306	4758	344	118	525	4610	3825	3983	250	1444	9	293	147
	2009	40015	19973	1885	8254	2834	1567	128	1243	4062	0	20042	4420	357	122	490	4410	3686	3580	1192	1408	12	225	140
	2010	38625	19919	1822	8206	2796	1601	125	1256	4112	0	18706	3863	476	162	568	4588	3600	3035	797	1281	11	186	139
	2011	37425	19793	1778	8126	2751	1593	123	1276	4146	0	17632	3634	584	199	548	4451	3628	2745	272	1245	9	184	132
	2012	36588	19405	1718	7939	2674	1542	120	1283	4128	0	17183	3505	637	217	526	4352	3624	2547	236	1234	11	166	128
	2013	35956	19026	1662	7754	2602	1491	118	1294	4106	0	16930	3497	587	200	503	4310	3544	2461	270	1254	9	169	126
	2014	35143	18438	1587	7486	2504	1424	114	1287	4036	0	16705	3423	639	217	486	4242	3554	2370	250	1246	12	141	124
	2015	34191	17927	1515	7252	2420	1371	111	1279	3979	0	16264	3293	682	232	462	4111	3499	2261	240	1214	12	137	121
Southern Asia	2000	3575436	1779867	124217	565574	449418	281359	95975	124806	113026	25493	1795569	562282	51873	17968	96613	303414	58585	448021	104497	112410	17369	7745	14791
	2001	3474010	1724579	119135	555993	431777	277054	83633	120954	111659	24375	1749432	539426	50857	17871	92576	291416	58043	421080	128625	110276	16023	8617	14624
	2002	3320133	1667178	113828	545436	413496	271518	72797	116819	110130	23153	1652955	513375	49493	17601	92210	277826	57155	392909	106035	107118	15601	9221	14411
	2003	3189984	1606800	108362	533509	394857	264251	62981	111368	108565	21907	1583184	489938	48241	17378	87622	265775	56382	367619	107089	104463	15152	9525	13998
	2004	3061458	1547493	102977	521273	376681	256504	54269	107961	107177	20651	1513965	467635	46969	17185	83359	254201	55569	343858	102610	107034	12287	9575	13682
	2005	2921879	1497226	98198	511782	360761	249238	47349	103980	106429	19488	1424653	446233	45620	16961	75679	243303	54718	321548	84872	99603	13540	9287	13288
	2006	2837903	1454424	93951	504436	346522	242733	41901	100369	106142	18371	1383479	425644	44684	16358	71887	232769	53841	300816	107162	97011	11451	8786	13071
	2007	2701190	1417729	90144	499359	333551	236647	37482	97047	106187	17311	1283461	404997	43575	15738	68072	221877	52770	280678	68675	94261	11543	8067	13206
	2008	2608001	1382458	86467	494319	320703	231176	33420	93876	106228	16269	1225543	385126	42406	15119	64403	211458	51611	261825	68970	91440	12592	7257	13336
	2009	2525979	1345083	82716	488813	307125	224794	29906	90427	106057	15246	1180896	349472	42130	15123	55271	208903	51465	250920	81917	92173	13593	6601	13328
	2010	2405131	1304238	78761	482654	292441	217061	26956	86513	105621	14231	1100893	330827	40947	14555	51920	199927	50180	234259	57844	89403	11725	6112	13194
	2011	2301704	1256313	74454	474429	276358	206706	24241	81971	104968	13186	1045390	314145	39650	13947	49033	190750	48709	218254	55338	86490	10158	5687	13230
	2012	2178529	1208093	70222	465193	260486	196458	21451	77496	104648	12138	970436	294988	37830	13308	45868	180000	46599	201031	39753	82622	10044	5297	13097
	2013	2068738	1156680	65961	453161	244465	186067	18563	72997	104397	11070	912058	268793	37069	13252	40526	176747	46131	191064	30369	81750	8880	4982	12497
	2014	1973359	1115298	62377	443610	230931	177669	16001	69293	105306	10112	858061	249284	35434	12708	37028	168527	44217	176999	30968	78474	7854	4739	11829
	2015	1890635	1078216	59191	434529	218929	169779	13741	65988	106792	9267	812419	227047	34929	12478	31155	164782	43845	168845	29287	77119	7187	4452	11294
Afghanistan	2000	131541	42488	3200	13087	11785	5099	3809	2704	2071	734	89053	25777	2569	642	5748	11693	2358	21854	10893	5806	205	19	1490
	2001	136474	42876	3228	12928	11996	5518	3593	2772	2123	719	93598	26262	2624	656	5846	12108	2420	21441	14534	5988	226	20	1473
	2002	130180	43497	3277	12721	12329	6140	3221	2906	2202	700	86683	26516	2659	665	5844	12398	2466	20824	7217	6129	470	21	1474
	2003	123862	43979	3332	12223	12749	7070	2497	3128	2299	682	79883	26831	2702	676	5854	12750	2523	20267	211	6289	283	23	1473
	2004	123733	44213	3345	11553	13050	8237	1605	3397	2380	646	79520	26892	2718	679	5804	13010	2554	19528	338	6395	145	25	1432
	2005	124109	43987	3293	11089	13001	8875	1169	3537	2413	611	80122	26689	2707	677	5692	13157	2559	18628	2069	6441	95	27	1381
	2006	124798	44007	3254	11081	12916	9109	1039	3567	2446	596	80791	26575	2702	675	5594	13328	2563	17844	3529	6501	80	29	1370
	2007	121311	43856	3210	11302	12758	8968	1062	3493	2472	592	77455	26265	2674	669	5434	13417	2544	16976	1517	6510	85	30	1335
	2008	119737	43469	3144	11455	12526	8811	1052	3418	2480	583	76268	25883	2639	660	5256	13474	2517	16117	1818	6492	69	32	1312
	2009	119935	42791	3061	11592	12212	8501	1069	3305	2476	574	77144	22798	2827	707	3926	14751	2703	16606	4404	7028	53	32	1310
	2010	116309	42311	2985	11976	11889	8079	1166	3170	2471	575	73998	22127	2776	694	3705	14737	2660	15692	3263	6968	63	34	1279

U5D[1]: total number of under 5 death; NEO[2]: total number of neonatal death; PNE[3]: pneumonia; PRE[4]: preterm; INT[5]: intrapartum related events; SEP[6]: sepsis/meningitis; TET[7]: tetanus; OTH[8]: other disorders; CON[9]: congenital; DIA[10]: diarrea; POS[11]: total number of post neonatal death; MEN[12]: meningitis; MEA[13]: measles; INJ[14]: injury; MAL[15]: malaria; PER[16]: pertussis

			0-28 days										1-59 months											
	Year	U5D[1]	NEO[2]	PNE[3]	PRE[4]	INT[5]	SEP[6]	TET[7]	OTH[8]	CON[9]	DIA[10]	POS[11]	PNE[3]	PRE[4]	INT[5]	MEN[12]	OTH[8]	CON[9]	DIA[10]	MEA[13]	INJ[14]	MAL[15]	AIDS	PER[16]
	2011	113006	41052	2849	12210	11325	7482	1225	2980	2419	563	71954	21327	2696	674	3497	14568	2587	14671	3739	6843	72	34	1247
	2012	108496	39588	2700	12200	10738	7073	1160	2818	2361	538	68908	20561	2595	649	3343	14256	2495	13581	3489	6661	37	36	1206
	2013	100914	38263	2566	12034	10248	6844	1037	2703	2322	508	62651	19552	2495	624	3132	13966	2405	12559	223	6479	28	38	1150
	2014	97692	37038	2444	11817	9814	6673	911	2608	2295	476	60654	17046	2528	632	2477	14461	2442	12234	1022	6641	31	39	1103
	2015	94261	36242	2353	11756	9494	6536	818	2538	2297	450	58019	16331	2426	606	2367	14117	2349	11285	973	6447	27	37	1055
Bangladesh	2000	317011	154428	11817	42522	38231	34342	3943	11819	9651	2104	162583	50526	4226	1591	9862	24690	3745	36333	12653	14023	4224	2	708
	2001	300606	148315	11195	41772	36019	33237	3345	11276	9561	1909	152291	47817	3966	1577	9244	23537	3610	32872	11187	13564	4219	2	696
	2002	283060	142672	10616	41014	34118	31978	2885	10766	9550	1745	140388	42917	3530	1484	12559	21255	3307	28203	9488	12450	4499	3	693
	2003	267975	136668	10036	39771	32515	30391	2504	10253	9589	1609	131307	39830	3247	1440	11535	19888	3131	25039	10036	11806	4683	4	667
	2004	262810	130245	9428	37901	31105	28734	2155	9754	9676	1492	132565	38009	3066	1437	10890	19156	3040	22857	20973	11512	975	5	646
	2005	245834	123764	8821	35603	29985	26997	1850	9288	9835	1384	122070	36104	2882	1427	6687	18383	2944	20777	20079	11169	977	6	636
	2006	224887	117106	8213	33143	28819	25295	1577	8816	9981	1262	107781	33089	2653	1321	6048	16996	2744	18221	14845	10456	768	8	632
	2007	195983	109823	7581	30786	27256	23513	1337	8267	9964	1119	86160	30206	2433	1218	5450	15665	2549	15924	1612	9744	741	9	609
	2008	183218	103027	6989	28834	25568	21909	1131	7737	9873	986	80191	27042	2185	1103	4813	14167	2320	13647	4073	8906	1336	10	589
	2009	168511	96815	6453	27253	23826	20507	949	7247	9708	872	71696	20637	2282	1160	2788	14962	2453	13594	3002	9451	771	11	584
	2010	157045	91438	5988	25990	22233	19297	799	6822	9528	781	65607	19066	2096	1070	2573	13830	2279	11922	2873	8810	498	12	579
	2011	149236	86839	5584	24933	20843	18251	677	6452	9395	704	62397	17381	1928	988	2308	12794	2122	10458	5136	8213	479	14	575
	2012	137961	82874	5235	24071	19632	17303	585	6118	9288	641	55087	16029	1787	919	2108	11924	1990	9254	2199	7713	565	17	581
	2013	130611	79859	4956	23490	18659	16567	514	5851	9228	593	50752	14822	1662	855	2103	11188	1869	8216	1679	7247	556	18	537
	2014	125294	77111	4702	23000	17770	15881	457	5603	9146	552	48183	13885	1567	806	1963	10641	1780	7403	2421	6890	306	18	503
	2015	119326	74378	4455	22507	16898	15210	408	5358	9027	515	44948	12953	1470	756	1825	10081	1688	6648	2266	6516	258	17	470
Bhutan	2000	1284	518	36	135	146	110	7	41	40	5	766	242	25	11	38	140	34	169	31	70	4	1	2
	2001	1223	497	33	133	138	104	6	39	40	4	726	221	23	10	34	131	32	147	57	65	4	2	2
	2002	1091	471	31	129	129	98	5	37	40	4	620	201	21	9	31	123	29	128	10	61	3	2	2
	2003	1002	448	28	126	120	93	4	34	39	3	554	180	19	8	27	113	27	110	5	56	4	3	2
	2004	925	426	26	124	111	88	3	32	39	3	499	163	17	8	24	105	25	95	4	52	2	4	2
	2005	861	407	24	122	104	83	3	30	39	2	454	148	16	7	22	98	23	83	3	48	1	4	2
	2006	811	394	23	120	98	80	3	29	40	2	417	136	14	6	20	91	22	73	3	45	2	4	2
	2007	768	384	22	119	94	77	2	27	41	2	384	125	13	6	18	86	21	64	0	42	0	5	2
	2008	729	373	21	117	89	75	2	26	41	2	356	116	13	6	16	81	19	57	0	40	0	6	2
	2009	686	358	19	114	84	71	2	25	41	1	328	106	12	5	15	76	18	50	1	37	1	6	2
	2010	637	337	18	109	77	66	1	23	41	1	300	96	11	5	13	70	17	43	1	35	0	7	2
	2011	587	314	16	104	70	60	1	21	39	1	273	88	10	4	12	64	16	38	1	32	0	7	2
	2012	534	287	15	97	63	54	1	19	37	1	247	65	10	4	7	65	16	36	1	33	0	8	2
	2013	484	262	13	91	57	48	1	17	35	1	222	58	9	4	6	59	14	31	1	30	0	8	2
	2014	444	242	12	85	51	43	1	15	33	1	202	53	8	4	5	54	13	28	1	27	0	7	2
	2015	414	226	11	81	47	39	1	14	32	1	188	49	8	3	5	51	13	25	1	26	0	6	2
India	2000	2514889	1253657	86070	416944	315114	189886	59557	86935	80661	18490	1261231	395234	38238	10890	66575	222887	42665	319302	71695	68618	11796	7643	5688
	2001	2443341	1215523	82589	409494	303365	185364	53151	84106	79793	17662	1227818	377078	37647	10847	63517	212313	42376	300556	91725	67184	10325	8488	5762
	2002	2337203	1175504	78909	401691	290827	180266	47236	81042	78771	16763	1161699	358723	36944	10757	60425	201630	41985	282158	79143	65598	9443	9064	5830
	2003	2249583	1134014	75106	393515	277713	174674	41738	77773	77690	15805	1115569	340667	36163	10644	57384	191315	41543	264499	85297	63938	9076	9339	5702
	2004	2139049	1091529	71237	384951	264243	168698	36624	74368	76604	14803	1047520	321971	35219	10488	54234	180611	40920	246839	69844	62017	10420	9362	5594
	2005	2037087	1054134	67722	378027	251955	163306	32083	71256	75939	13847	982952	303801	34182	10285	51174	170138	40232	230047	57163	60039	11286	9047	5559
	2006	1975025	1016659	64241	370841	239628	157716	27945	68088	75326	12874	958366	287153	33523	9814	48369	160614	39571	214875	82781	58172	9518	8521	5455
	2007	1867629	981810	60965	364355	227918	152321	24303	65058	74957	11932	885819	269832	32653	9299	45452	150570	38686	199624	60878	56012	9552	7781	5481
	2008	1783995	946336	57717	357415	216042	146601	21069	61921	74576	10995	837659	253086	31722	8771	42631	141206	37710	185236	60791	53781	10612	6953	5161
	2009	1711551	909587	54425	349795	203849	141003	18182	58672	74151	10063	801964	235911	30612	8219	39738	131348	36448	170985	73583	51233	12519	6282	5086
	2010	1602003	871304	51090	341361	191315	133841	15558	55296	73705	9138	730699	220764	29625	7732	37187	122620	35320	158504	48418	48959	10746	5770	5054
	2011	1512325	831083	47699	331801	178451	126723	13130	51791	73274	8215	681241	206481	28621	7245	34781	114339	34142	146956	42422	46698	9156	5326	5075
	2012	1412260	792589	44476	322342	166158	119666	10938	48418	73258	7333	619671	190548	27174	6794	32097	105070	32430	134575	28061	43838	9039	4915	5129
	2013	1328929	752702	41282	311508	153870	112239	8945	45007	73383	6470	576227	176053	26362	6591	28969	99504	31769	126234	21000	42338	8041	4580	4788
	2014	1260762	721743	38686	303388	143792	106019	7299	42218	74607	5734	539019	162337	25000	6250	26564	92305	30219	116288	24226	39867	7213	4307	4443
	2015	1200998	695852	36497	296177	135303	100451	5973	39870	76470	5112	505146	143228	24851	6213	21106	90016	30253	112277	22897	39478	6627	4036	4164
Iran (Islamic Republic of)	2000	44855	24636	1306	8763	5502	3590	111	1564	3729	71	20219	6052	858	214	701	4619	1416	3660	154	2299	1	15	231
	2001	41515	23334	1221	8375	5139	3324	98	1470	3643	64	18181	5460	783	196	626	4158	1320	3173	107	2106	0	18	234
	2002	38307	21841	1129	7892	4740	3065	86	1362	3510	57	16466	4947	719	180	560	3767	1239	2763	105	1937	0	21	227
	2003	35386	20662	1058	7490	4426	2866	76	1277	3417	52	14724	4423	652	163	494	3358	1150	2374	101	1758	1	25	224
	2004	32955	19554	993	7087	4144	2685	68	1202	3328	48	13401	4053	607	152	447	3067	1097	2092	0	1634	0	28	225
	2005	31119	18821	950	6796	3961	2552	61	1156	3301	45	12298	3724	562	140	405	2797	1029	1849	1	1523	0	32	235
	2006	29989	18337	922	6586	3838	2455	57	1126	3312	42	11652	3527	539	135	380	2641	1007	1686	2	1462	0	36	238
	2007	29135	18005	902	6428	3751	2382	53	1104	3346	40	11130	3371	519	130	360	2512	985	1551	2	1415	1	39	246
	2008	28270	17552	873	6185	3642	2332	47	1086	3349	38	10718	3248	505	126	344	2407	972	1438	2	1382	1	38	257
	2009	27467	17099	844	5908	3537	2323	41	1080	3330	35	10368	3146	491	123	331	2318	953	1342	3	1355	0	41	267
	2010	26641	16440	803	5537	3392	2320	35	1078	3244	32	10201	3097	486	121	324	2276	950	1274	5	1347	0	46	274
	2011	25681	15848	766	5243	3258	2287	30	1067	3168	29	9833	2993	469	117	312	2191	918	1189	3	1314	0	50	276
	2012	24611	15114	725	4952	3094	2200	27	1030	3059	27	9497	2894	456	114	302	2114	898	1110	4	1281	0	49	276
	2013	23477	14266	682	4669	2906	2066	25	967	2925	25	9211	2809	445	111	293	2056	883	1042	3	1253	0	48	267
	2014	22214	13486	642	4412	2733	1943	24	910	2800	23	8728	2665	424	106	279	1953	847	955	3	1198	0	45	253
	2015	21010	12799	606	4192	2580	1833	22	859	2684	22	8211	2509	401	100	263	1841	808	870	3	1136	0	42	238
Maldives	2000	281	159	11	74	22	19	0	14	19	0	122	35	1	2	3	35	19	15	0	11	0	0	1
	2001	242	141	9	64	19	15	0	13	19	0	101	28	1	1	3	29	17	11	0	9	0	0	1
	2002	220	126	8	56	17	13	0	12	20	0	94	22	1	1	2	22	15	9	13	8	0	0	1
	2003	183	110	7	48	14	10	0	11	19	0	73	18	1	1	2	19	14	7	1	8	0	0	1
	2004	177	97	6	41	12	9	0	10	19	0	80	15	1	1	2	16	14	6	1	24	0	0	1
	2005	144	86	5	36	10	7	0	9	18	0	58	12	1	1	1	14	13	5	2	6	0	0	1
	2006	128	76	4	31	9	7	0	7	17	0	52	11	1	1	1	13	13	4	1	6	0	0	1
	2007	117	69	4	28	8	6	0	7	16	0	48	9	1	1	1	13	12	3	0	6	0	0	1
	2008	108	63	3	25	7	5	0	6	16	0	45	8	1	1	1	12	12	3	0	5	0	0	1
	2009	100	58	3	23	7	5	0	6	15	0	42	7	1	1	1	12	11	3	0	5	0	0	1
	2010	93	54	2	22	6	4	0	5	14	0	39	6	1	1	1	11	11	2	0	4	0	0	1
	2011	86	50	2	20	6	4	0	5	13	0	36	6	1	1	1	10	10	2	0	4	0	0	1
	2012	80	46	2	18	5	3	0	5	12	0	34	5	1	1	1	9	10	2	0	4	0	0	1
	2013	76	44	2	18	5	3	0	4	12	0	32	4	1	2	1	9	10	1	0	3	0	0	1
	2014	71	41	1	16	5	3	0	4	11	0	30	4	1	2	1	8	9	1	0	3	0	0	1
	2015	67	38	1	15	4	3	0	4	11	0	29	4	1	2	1	8	9	1	0	3	0	0	1
Nepal	2000	63022	30187	2377	7707	8497	5385	1201	2245	2346	430	32835	9631	672	476	1911	3991	782	6512	5153	2771	14	45	879
	2001	60411	28629	2214	7176	8050	5404	882	2175	2347	381	31782	9037	626	455	1785	3727	743	5836	5948	2664	11	62	886
	2002	55459	27051	2047	6640	7570	5398	633	2100	2331	332	28408	8333	575	427	1639	3410	694	5137	4683	2513	27	80	888
	2003	53727	25328	1873	6154	7010	5259	470	1994	2280	288	28399	7661	526	400	1502	3131	647	4513	6677	2362	19	95	865
	2004	50375	23727	1722	5800	6491	4976	381	1870	2233	254	26648	6985	477	371	1363	2849	597	3933	6918	2201	10	108	835
	2005	42197	22249	1588	5552	6009	4628	329	1737	2178	228	19948	6408	435	347	1245	2586	554	3453	1895	2060	14	118	834
	2006	38177	21031	1478	5401	5606	4287	296	1611	2142	208	17146	5823	396	318	1125	2390	511	3004	773	1910	15	122	760
	2007	35788	19927	1376	5269	5250	3967	269	1501	2105	190	15861	5364	365	296	1029	2189	477	2652	811	1791	8	125	753
	2008	33671	18952	1284	5150	4934	3691	243	1403	2074	173	14719	4912	336	273	935	2004	443	2329	957	1670	7	124	729
	2009	30845	17901	1186	4976	4592	3453	211	1305	2021	155	12944	4528	310	254	855	1876	415	2059	274	1567	6	118	681
	2010	28690	16869	1090	4781	4259	3243	180	1216	1962	138	11821	4130	284	234	774	1692	384	1802	274	1454	8	111	675
	2011	26710	15679	988	4527	3892	3014	150	1117	1869	121	11031	3206	297	246	468	1868	407	1802	462	1537	4	98	636
	2012	24895	14595	897	4296	3564	2790	127	1030	1783	107	10300	2898	267	222	423	1659	371	1555	787	1401	4	89	624
	2013	22271	13580	816	4081	3261	2580	108	947	1692	95	8691	2632	246	204	379	1554	344	1368	19	1298	2	76	567
	2014	21017	12800	753	3927	3024	2414	94	883	1620	86	8217	2428	228	190	349	1466	323	1216	212	1212	3	67	523
	2015	19900	12252	706	3831	2846	2294	83	836	1576	80	7648	2257	213	177	323	1392	305	1090	198	1140	2	63	487
Pakistan	2000	497089	270407	19218	75114	69676	42711	27323	18973	13733	3660	226682	74417	5266	4130	11724	34818	7053	59953	3918	18515	1108	20	5762
	2001	484848	261938	18476	74849	66617	43873	22531	18589	13367	3635	222910	73179	5165	4114	11473	34867	7017	56839	5066	18402	1226	24	5539
	2002	469284	252679	17649	74091	63336	44349	18703	18066	12932	3552	216605	71389	5023	4063	11104	34668	6914	53493	5376	18130	1153	28	5265
	2003	452898	242226	16765	72974	59876	43675	15665	17355	12448	3468	210672	70007	4908	4029	10779	34629	6835	50622	4761	17951	1086	35	5032
	2004	441076	234244	16064	72578	57082	42862	13405	16759	12088	3405	206832	69254	4841	4034	10552	34836	6830	48340	4532	17922	735	42	4915
	2005	435347	230530	15658	73393	55319	42596	11822	16435	11935	3371	204817	69059	4810	4060	10413	35561	6854	46545	3661	18031	1166	50	4607

U5D[1]: total number of under 5 death; NEO[2]: total number of neonatal death; PNE[3]: pneumonia; PRE[4]: preterm; INT[5]: intrapartum related events; SEP[6]: sepsis/meningitis; TET[7]: tetanus; OTH[8]: other disorders; CON[9]: congenital; DIA[10]: diarrea; POS[11]: total number of post neonatal death; MEN[12]: meningitis; MEA[13]: measles; INJ[14]: injury; MAL[15]: malaria; PER[16]: pertussis

			0-28 days									1-59 months												
	Year	U5D[1]	NEO[2]	PNE[3]	PRE[4]	INT[5]	SEP[6]	TET[7]	OTH[8]	CON[9]	DIA[10]	POS[11]	PNE[3]	PRE[4]	INT[5]	MEN[12]	OTH[8]	CON[9]	DIA[10]	MEA[13]	INJ[14]	MAL[15]	AIDS	PER[16]
	2006	439164	233735	15690	76127	55211	43608	10955	16623	12134	3388	205429	69068	4830	4069	10313	36144	6912	44964	5230	18188	1068	62	4579
	2007	445798	240970	15971	80034	56146	45257	10427	17119	12581	3436	204828	69582	4887	4100	10294	36889	7008	43753	3856	18482	1156	75	4745
	2008	453906	250014	16334	84176	57552	47615	9848	17840	13156	3493	203892	70610	4975	4159	10375	37591	7142	42882	1327	18919	567	91	5253
	2009	462758	257989	16634	88255	58701	49362	9425	18374	13693	3545	204769	62131	5565	4632	7588	43059	7994	46174	651	21262	242	109	5362
	2010	469801	263193	16702	92050	58979	50114	9184	18520	14078	3567	206608	61357	5619	4663	7308	44202	8072	44929	3009	21613	409	129	5298
	2011	470354	263319	16476	94822	58244	48800	8994	18180	14251	3553	207035	62491	5576	4635	7619	44434	8027	43054	3575	21640	446	151	5385
	2012	466145	261003	16105	96494	56980	47290	8582	17722	14339	3491	205142	61824	5485	4566	7556	44430	7917	40841	5211	21490	399	178	5245
	2013	458601	255837	15583	96593	55225	45647	7904	17185	14321	3379	202764	52707	5792	4822	5612	47949	8377	41541	7443	22907	253	208	5152
	2014	442647	251055	15080	96317	53519	44626	7188	16751	14335	3239	191592	50723	5621	4679	5364	47197	8145	38808	3082	22452	301	250	4970
	2015	431568	244746	14507	95360	51545	43348	6410	16225	14262	3088	186822	49578	5502	4580	5239	46841	7990	36586	2948	22193	273	244	4848
Sri Lanka	2000	5464	3387	182	1229	445	217	25	512	776	0	2077	367	19	13	51	541	515	223	0	299	18	1	30
	2001	5350	3326	170	1202	433	214	26	515	767	0	2024	346	20	14	48	545	509	206	0	294	11	2	30
	2002	5328	3337	162	1201	431	211	29	529	774	0	1991	329	21	14	46	552	505	194	0	291	6	2	31
	2003	5369	3365	157	1208	433	214	28	543	783	0	2004	321	23	15	45	572	512	186	0	296	1	2	31
	2004	10358	3458	154	1238	444	215	28	569	810	0	6900	295	23	16	42	552	492	169	0	5277	0	2	32
	2005	5181	3248	138	1164	418	193	32	531	771	0	1933	287	26	17	41	570	509	161	0	286	0	3	33
	2006	4924	3079	125	1105	396	178	30	502	743	0	1845	263	27	18	37	551	497	144	0	271	0	4	33
	2007	4661	2885	113	1037	371	157	29	472	707	0	1776	243	28	19	35	535	489	131	0	259	0	4	33
	2008	4368	2672	101	962	343	138	27	439	663	0	1696	222	29	20	32	516	477	118	0	245	0	4	33
	2009	4127	2485	91	895	318	120	26	412	622	0	1642	208	31	21	30	502	470	108	0	236	0	4	34
	2010	3912	2292	82	827	292	97	33	382	578	0	1620	182	49	34	36	489	487	91	0	213	0	5	33
	2011	3718	2129	74	769	270	85	33	358	540	0	1589	173	52	36	34	482	481	84	0	208	0	6	33
	2012	3547	1997	67	723	252	79	31	336	509	0	1550	164	54	38	32	473	471	77	0	203	0	6	33
	2013	3375	1867	61	676	235	73	29	315	478	0	1508	155	56	39	30	462	459	71	0	196	0	7	32
	2014	3218	1782	57	646	224	69	27	300	458	0	1436	145	57	40	28	442	437	66	0	184	0	7	31
	2015	3091	1683	53	610	211	65	26	285	434	0	1408	139	57	40	26	435	431	63	0	181	0	7	30
Sub-Saharan Africa	2000	4272148	1104867	83576	327390	341057	161376	57805	62076	61111	10474	3167281	596330	51741	28282	145183	319632	66981	557971	340135	124808	699363	205147	31708
	2001	4223624	1103540	83594	326429	341592	165140	52670	62766	61114	10235	3120084	597031	51926	28477	149546	320995	67701	535278	310990	127257	688074	210471	32338
	2002	4108332	1099727	83323	324253	341000	169184	47342	63356	61333	9935	3008605	585192	52051	28682	142975	322035	68495	512179	242262	129762	678929	213084	32959
	2003	4087690	1095255	82815	322041	339440	173498	42359	63707	61813	9581	2992435	572735	51140	28386	148323	316932	68088	480324	287224	129797	663120	213281	33084
	2004	3889732	1089637	82067	319230	336995	178076	37689	63859	62559	9162	2800095	567381	51210	28545	138497	317523	68844	458329	163864	132350	629578	210594	33381
	2005	3837005	1083682	81189	316224	334081	182240	33839	63790	63605	8714	2753323	558224	50766	28452	143224	314649	69148	432979	187810	133395	595406	205671	33601
	2006	3621425	1075262	80018	312550	330171	185358	30549	63684	64678	8253	2546163	556557	51337	28834	115587	318399	70726	417023	48855	136888	569481	198609	33867
	2007	3524667	1068956	79114	309118	326308	189557	27675	63153	66207	7824	2455711	539984	51831	29126	110609	321494	72215	401129	36472	140073	530918	187926	33936
	2008	3463594	1062192	77942	305564	322404	192687	25066	63200	67877	7452	2401402	526545	51950	29280	121459	322692	73353	382663	60864	142468	483614	172474	34039
	2009	3374372	1056974	77011	303586	319263	193726	23155	63282	69773	7177	2317398	508510	53099	30061	98457	331872	75945	372548	55796	147839	452450	156784	34038
	2010	3293859	1052345	76425	304753	315964	191895	22213	62242	71823	7030	2241514	506381	53625	30457	85029	336077	77558	358834	59830	151372	411508	136278	34564
	2011	3260239	1046671	75548	306459	312634	187711	21767	61436	74174	6941	2213568	497221	54334	30918	81850	341402	79344	346176	99618	155572	370942	120914	35276
	2012	3150724	1040591	74705	307835	308554	184540	21198	60568	76335	6856	2110133	477564	54728	31183	91086	344473	80720	331183	62598	158714	338092	103826	35968
	2013	3108944	1036411	73989	308817	305427	182965	20316	59836	78323	6738	2072533	452196	55802	31839	79503	353478	83027	320700	86924	163771	322181	88329	34784
	2014	2999404	1031544	73348	308157	302329	182290	19261	59339	80233	6588	1967860	425481	56785	32425	71261	361761	85250	305132	39283	168464	309151	79141	33727
	2015	2947137	1026860	72698	307528	299777	181034	18270	59064	82056	6433	1920277	418432	56170	32106	70070	358570	85172	288863	38218	168037	294613	77185	32842
Angola	2000	157186	43116	3114	12244	13261	5219	3923	2229	2739	388	114070	27505	2406	1253	6695	15058	2999	29971	1292	4724	19827	1449	890
	2001	163043	44214	3184	12585	13568	5707	3730	2308	2741	391	118829	28173	2480	1300	6781	15659	3140	29692	3896	4877	20343	1611	878
	2002	167682	45094	3241	12858	13844	6158	3458	2393	2743	399	122588	29612	2629	1386	4588	16702	3394	30201	5036	5164	21211	1773	891
	2003	167942	46400	3335	13202	14351	6446	3331	2538	2790	407	121542	30482	2731	1447	4626	17422	3598	30075	1776	5357	21173	1931	922
	2004	169745	47568	3422	13533	14817	6695	3168	2686	2831	417	122177	31581	2860	1524	4664	18317	3856	30144	23	5594	20609	2072	933
	2005	173224	48603	3492	13859	15186	6987	2995	2811	2847	425	124621	32914	3011	1615	4745	19329	4152	30367	270	5882	19165	2203	969
	2006	176239	49499	3531	14246	15351	7450	2812	2862	2817	429	126740	34366	3189	1709	4831	20474	4502	30598	675	6207	16938	2249	1001
	2007	177174	50458	3575	14587	15496	8061	2629	2905	2777	428	126716	30148	3627	1941	3343	23425	5247	33002	165	7042	15553	2321	902
	2008	178009	50990	3586	14840	15517	8537	2429	2932	2721	428	127019	31124	3785	2017	3432	24412	5590	32657	261	7345	13107	2394	894
	2009	178779	51640	3612	15204	15623	8789	2338	2959	2686	429	127139	32120	3868	2055	3602	24888	5829	31650	943	7519	11303	2432	929
	2010	177885	52107	3638	15729	15674	8692	2368	2919	2653	434	125778	31062	4017	2128	3246	25908	6167	31180	324	7831	10607	2443	865
	2011	176999	52434	3656	16166	15733	8524	2388	2901	2630	436	124565	31240	4020	2122	3331	25932	6261	29637	494	7892	10222	2546	868
	2012	176071	52604	3667	16445	15774	8427	2335	2904	2618	435	123467	30532	4047	2128	3190	26072	6419	28262	1127	7979	10238	2622	851
	2013	175642	52860	3686	16583	15893	8482	2199	2957	2629	432	122782	29314	4078	2145	2965	26269	6597	27011	2648	8088	10401	2430	836
	2014	171585	53041	3693	16708	15948	8563	2067	2994	2641	427	118544	26376	4260	2240	2447	27452	7027	26019	454	8493	10851	2103	821
	2015	169310	53164	3695	16855	15961	8611	1943	3018	2659	421	116146	25912	4236	2228	2395	27268	7127	24520	442	8507	10647	2060	805
Benin	2000	42530	11333	721	3727	3047	2470	165	603	519	80	31197	6517	589	318	1303	4338	800	5902	2818	1426	6315	575	296
	2001	43661	11299	714	3753	3018	2459	156	598	522	78	32362	6384	579	314	1941	4274	796	5561	3893	1415	6294	605	306
	2002	40897	11346	714	3775	3024	2477	145	603	532	75	29551	6338	577	314	1919	4268	800	5303	1193	1427	6473	627	315
	2003	40239	11366	712	3753	3029	2513	130	615	543	71	28873	6263	573	312	1891	4236	802	5032	628	1432	6733	644	326
	2004	39586	11396	709	3703	3036	2583	111	634	554	67	28190	6159	566	310	1855	4183	802	4754	127	1430	7011	655	338
	2005	39543	11457	706	3691	3037	2648	99	647	565	63	28086	5711	578	318	1620	4280	828	4643	287	1475	7336	661	349
	2006	39222	11511	703	3707	3025	2698	93	648	576	61	27711	5387	611	337	922	4557	884	4703	137	1575	7586	661	351
	2007	39186	11541	699	3749	3004	2712	91	639	587	59	27645	5369	627	347	901	4703	915	4625	322	1630	7204	655	346
	2008	39349	11586	698	3799	3000	2704	91	636	600	59	27763	5588	643	357	954	4838	948	4554	685	1683	6544	617	353
	2009	39411	11654	702	3849	3014	2686	90	639	617	58	27757	5688	667	371	962	5043	993	4534	913	1755	5935	548	348
	2010	38955	11716	706	3887	3032	2666	89	646	634	57	27239	5851	684	380	997	5186	1025	4470	623	1811	5375	485	352
	2011	38729	11816	711	3934	3055	2663	88	655	653	57	26913	5467	717	400	891	5468	1084	4511	556	1912	5072	479	356
	2012	38148	11838	709	3954	3053	2653	85	661	668	56	26310	4894	741	414	1105	5664	1130	4479	204	1988	4849	479	362
	2013	38228	11895	710	3988	3054	2657	83	665	684	55	26333	5123	745	416	942	5700	1144	4323	517	2007	4611	448	358
	2014	37510	11921	708	4013	3045	2655	80	665	701	54	25589	5056	744	416	921	5705	1152	4143	261	2021	4424	394	351
	2015	37092	11955	706	4043	3036	2653	78	666	720	53	25137	4995	739	413	908	5669	1154	3947	256	2021	4303	387	345
Botswana	2000	4506	1130	71	438	287	151	8	74	96	4	3376	258	29	12	31	204	47	194	689	73	21	1810	8
	2001	3870	1140	71	444	287	153	8	74	98	4	2730	268	31	13	32	213	50	195	106	77	11	1726	8
	2002	3721	1151	72	451	286	155	8	74	101	5	2570	280	32	14	33	223	53	197	51	81	4	1595	8
	2003	3618	1162	72	459	286	157	8	73	103	5	2456	290	34	14	34	232	56	197	42	84	4	1461	8
	2004	3499	1173	72	465	285	160	8	72	107	5	2326	303	36	15	35	243	60	198	31	89	7	1299	8
	2005	3190	1183	72	468	283	163	8	68	116	5	2007	319	38	16	37	257	65	197	28	97	1	944	8
	2006	2932	1195	72	470	282	167	8	66	125	5	1737	327	39	16	37	264	68	191	10	103	5	666	8
	2007	2951	1208	72	475	284	170	7	67	127	5	1743	341	41	17	38	276	72	193	15	108	1	632	8
	2008	2976	1222	73	477	286	175	7	69	130	5	1754	350	42	18	39	284	75	192	19	111	2	614	8
	2009	2849	1233	73	473	287	181	7	69	138	5	1616	355	43	18	39	287	77	185	40	116	2	445	9
	2010	2777	1247	73	473	288	186	6	70	147	5	1530	355	43	18	39	288	78	177	86	119	2	317	9
	2011	2733	1261	74	476	290	188	6	71	152	5	1472	299	49	20	25	324	88	191	43	135	1	288	10
	2012	2685	1280	74	482	293	190	6	71	159	5	1405	265	52	22	18	347	95	176	33	146	0	242	8
	2013	2589	1294	75	485	294	191	6	71	168	5	1295	253	53	22	17	349	96	168	21	149	1	158	8
	2014	2537	1290	74	483	291	190	6	70	172	5	1247	239	53	22	14	352	98	162	18	152	0	129	8
	2015	2488	1268	72	475	285	186	5	69	172	4	1220	235	52	22	14	345	97	153	18	150	0	126	7
Burkina Faso	2000	100120	21824	1495	7267	6025	4064	671	1078	1019	205	78296	8312	695	357	2782	4405	792	8034	10797	1575	37894	2028	624
	2001	96835	21953	1496	7297	6047	4198	609	1094	1012	201	74882	8803	736	379	2940	4792	842	8214	6316	1686	37691	1879	603
	2002	96850	21971	1483	7291	6014	4352	541	1102	995	194	74879	9339	783	404	3109	5198	901	8394	5903	1816	36706	1726	602
	2003	95124	21822	1462	7210	5950	4473	467	1103	974	184	73302	9897	832	431	3282	5642	964	8540	4585	1956	35016	1570	587
	2004	90108	21614	1431	7090	5858	4616	396	1100	951	171	68494	10372	875	454	3423	6028	1023	8553	984	2099	32675	1424	584
	2005	87556	21226	1393	6886	5754	4680	330	1095	934	155	66330	10505	891	462	3452	6195	1051	8245	525	2187	30942	1289	586
	2006	85272	20827	1358	6674	5657	4706	275	1089	929	140	64445	8781	909	471	2449	6366	1084	7926	912	2288	31525	1143	591
	2007	81824	20548	1332	6499	5607	4714	232	1093	945	126	61276	7873	851	440	2107	5950	1026	6960	826	2200	31461	993	589
	2008	77832	20300	1308	6354	5548	4706	201	1095	974	114	57532	7048	773	400	1853	5396	942	5945	511	2063	31093	920	588
	2009	91064	20047	1280	6200	5476	4705	173	1097	1012	103	71017	6637	730	378	1144	5083	897	5280	17473	2008	30083	703	603
	2010	72981	19645	1243	6032	5354	4638	149	1088	1047	94	53336	6631	732	379	1136	5129	908	4999	2756	2072	27349	628	616
	2011	68916	19246	1208	5880	5227	4556	131	1078	1081	86	49670	7064	786	407	1201	5594	979	5093	1552	2281	23515	572	626
	2012	74155	18926	1178	5792	5119	4455	119	1066	1116	81	55229	7593	851	441	1948	6143	1069	5241	9298	2519	18982	508	636
	2013	63832	18652	1153	5731	5014	4365	111	1051	1150	77	45180	8118	914	474	1839	6718	1157	5296	1229	2752	15634	441	608
	2014	62096	18403	1130	5692	4909	4279	104	1033	1182	74	43693	6642	1036	537	1207	7765	1323	5072	1436	3166	14528	397	584
	2015	60477	18346	1117	5712	4852	4244	99	1025	1224	72	42131	6656	1049	544	1208	7930	1349	4904	1386	3247	12912	383	563
Burundi	2000	43663	10887	847	3404	3361	1692	320	609	544	110	32776	6696	525	306	1667	3522	606	5678	2125	1425	8518	1432	277

U5D[1]: total number of under 5 death; NEO[2]: total number of neonatal death; PNE[3]: pneumonia; PRE[4]: preterm; INT[5]: intrapartum related events; SEP[6]: sepsis/meningitis; TET[7]: tetanus; OTH[8]: other disorders; CON[9]: congenital; DIA[10]: diarrea; POS[11]: total number of post neonatal death; MEN[12]: meningitis; MEA[13]: measles; INJ[14]: injury; MAL[15]: malaria; PER[16]: pertussis

			0-28 days										1-59 months											
	Year	U5D[1]	NEO[2]	PNE[3]	PRE[4]	INT[5]	SEP[6]	TET[7]	OTH[8]	CON[9]	DIA[10]	POS[11]	PNE[3]	PRE[4]	INT[5]	MEN[12]	OTH[8]	CON[9]	DIA[10]	MEA[13]	INJ[14]	MAL[15]	AIDS	PER[16]
	2001	43305	10923	848	3340	3409	1739	300	626	556	105	32382	6668	523	306	1668	3553	606	5428	2412	1442	8021	1477	278
	2002	40702	11039	856	3294	3483	1806	277	649	574	100	29663	6554	515	303	2522	3532	603	5118	196	1442	7096	1497	285
	2003	40306	11249	869	3317	3577	1852	264	674	599	96	29057	6951	548	322	1759	3815	645	5202	18	1558	6450	1496	292
	2004	40253	11473	883	3374	3663	1885	254	695	627	92	28780	6188	621	366	1248	4407	734	5622	94	1796	5928	1473	302
	2005	40106	11715	897	3448	3742	1923	242	714	660	89	28391	6212	634	374	1864	4551	756	5461	128	1871	4836	1394	311
	2006	39911	12006	913	3524	3816	2003	227	738	698	87	27905	6395	669	396	1254	4865	804	5475	178	2018	4235	1299	317
	2007	39468	12261	923	3541	3872	2126	199	776	741	84	27207	6342	683	406	1205	5019	829	5301	52	2107	3746	1193	324
	2008	39274	12539	929	3539	3930	2271	170	833	788	80	26735	6438	681	406	1260	5035	833	5022	118	2147	3362	1087	347
	2009	39132	12720	930	3529	3961	2356	145	892	831	76	26412	6436	687	411	1249	5141	848	4835	147	2213	3135	950	360
	2010	38789	12974	940	3621	4012	2383	138	924	882	75	25815	6399	690	415	1232	5220	858	4633	78	2264	2889	766	370
	2011	38687	13088	943	3739	4017	2342	138	911	923	75	25599	6253	709	429	1163	5433	888	4558	78	2365	2722	620	381
	2012	38356	13169	940	3844	3997	2324	137	892	960	75	25187	5037	779	474	639	6077	984	4796	103	2639	2753	516	391
	2013	37823	13201	934	3908	3967	2311	133	880	995	73	24622	4992	776	472	634	6126	986	4572	31	2662	2566	422	383
	2014	37343	13261	929	3953	3948	2319	127	880	1034	71	24082	5070	787	479	658	6291	1007	3831	38	2733	2490	323	375
	2015	36970	13236	920	3982	3910	2292	122	874	1067	70	23734	5010	781	475	651	6306	1006	3647	37	2742	2392	318	369
Cabo Verde	2000	453	218	10	81	39	20	6	30	31	0	235	65	2	1	6	31	32	44	28	14	0	10	1
	2001	403	196	9	72	34	14	10	27	31	0	207	56	2	1	5	29	30	36	25	12	0	10	1
	2002	367	177	8	64	31	16	5	24	30	0	190	49	2	1	4	27	29	30	25	11	1	10	1
	2003	342	164	7	59	28	15	3	21	29	0	178	44	2	1	4	25	27	26	25	11	1	10	1
	2004	326	156	7	57	27	14	3	19	28	0	170	42	2	1	4	25	27	23	25	10	1	9	1
	2005	320	152	7	57	26	12	3	18	28	0	168	41	2	1	4	25	27	21	25	11	1	9	2
	2006	304	152	8	58	26	12	3	17	28	0	152	42	2	1	4	26	28	20	7	12	3	7	1
	2007	308	154	8	60	27	12	2	16	29	0	154	42	3	1	4	29	29	19	7	12	1	7	2
	2008	313	158	8	62	27	13	2	16	30	0	155	42	3	1	4	30	29	18	7	13	1	5	2
	2009	316	160	9	63	27	13	2	16	30	0	156	41	3	1	4	32	30	18	8	13	1	5	2
	2010	311	159	9	63	27	14	1	16	30	0	152	40	3	1	4	34	30	18	3	14	0	5	2
	2011	305	156	9	62	26	14	1	15	30	0	149	35	4	1	4	36	30	17	3	12	1	4	2
	2012	296	150	8	59	25	13	1	15	29	0	146	34	4	1	4	37	30	16	3	12	0	3	2
	2013	287	143	8	56	23	13	1	14	28	0	144	33	4	1	4	37	30	16	3	12	0	2	2
	2014	277	138	7	54	22	12	1	13	28	0	139	31	4	2	4	35	29	15	4	12	0	2	2
	2015	267	133	7	52	21	12	1	13	27	0	134	29	4	1	3	34	29	14	4	12	0	2	2
Cameroon	2000	97581	21876	1609	7205	6895	3074	558	1219	1135	180	75705	14556	1407	758	2556	8628	2135	13613	4653	3118	18708	4828	746
	2001	99242	21630	1594	7064	6821	3123	497	1225	1134	173	77612	14080	1361	735	3768	8333	2073	12632	7005	3068	18770	5030	756
	2002	91181	21532	1587	6906	6792	3250	436	1249	1147	165	69649	13986	1356	733	2438	8286	2076	12005	223	3112	19530	5143	762
	2003	89713	21562	1586	6759	6792	3437	381	1280	1170	157	68151	13583	1320	715	2359	8038	2032	11151	150	3087	19761	5194	761
	2004	88593	21687	1586	6647	6803	3658	334	1313	1198	149	66906	13256	1291	701	2297	7834	1999	10417	359	3073	19711	5198	770
	2005	87587	21890	1590	6573	6839	3870	301	1342	1233	141	65697	13188	1287	701	2283	7817	2005	9937	514	3112	18867	5224	762
	2006	86174	21894	1579	6431	6828	4025	270	1367	1263	132	64280	13454	1317	720	2329	8012	2071	9721	404	3232	17084	5172	763
	2007	84516	21773	1565	6239	6822	4088	239	1403	1295	122	62743	13607	1335	732	3605	8124	2112	9435	224	3325	14382	5101	761
	2008	82905	21613	1549	6055	6808	4100	213	1448	1327	114	61292	14105	1387	762	3737	8470	2209	9393	160	3502	11874	4932	761
	2009	81385	21521	1543	5977	6821	4019	202	1480	1370	110	59864	12665	1572	866	1898	9670	2518	10193	282	4023	10659	4737	781
	2010	79233	21509	1543	5996	6825	3939	202	1476	1421	107	57724	12708	1602	885	1887	9895	2577	9956	122	4175	8974	4167	776
	2011	77712	21329	1531	6034	6763	3791	205	1439	1460	106	56383	12161	1649	914	1765	10197	2670	9813	290	4361	7989	3781	793
	2012	76137	21157	1513	5991	6687	3747	197	1424	1495	103	54980	10209	1744	969	1299	10832	2840	9943	373	4677	7739	3563	792
	2013	74253	21143	1505	5975	6653	3755	188	1425	1543	100	53110	9809	1734	964	1311	10790	2837	9467	166	4713	7390	3161	768
	2014	72574	21118	1494	5931	6614	3782	177	1434	1589	97	51456	9797	1721	956	1329	10721	2829	8399	117	4737	7180	2925	744
	2015	71348	21071	1484	5924	6571	3766	171	1430	1629	95	50277	9609	1694	941	1303	10553	2798	7940	113	4710	7028	2858	727
Central African Republic	2000	25848	7093	516	2286	2126	912	451	367	347	88	18755	2631	233	127	988	1357	310	2598	1634	517	6141	2042	178
	2001	25991	7147	518	2317	2134	929	446	368	347	87	18844	2655	235	129	997	1377	313	2539	1496	524	6388	2012	180
	2002	25886	7136	514	2335	2118	939	435	366	344	86	18750	2690	237	131	662	1406	316	2489	1386	534	6775	1945	179
	2003	25399	7093	509	2315	2103	955	425	361	341	85	18306	2631	231	128	649	1379	309	2356	1048	525	7019	1852	178
	2004	25813	7057	505	2275	2099	985	413	357	339	83	18756	2561	225	125	632	1350	301	2217	1708	514	7214	1736	173
	2005	24731	7002	501	2214	2093	1023	398	354	338	81	17729	2487	218	122	936	1315	292	2081	880	503	7114	1608	172
	2006	23608	6943	498	2132	2092	1078	371	355	337	79	16665	2594	228	128	633	1385	307	2095	69	529	7088	1436	173
	2007	23283	6913	496	2050	2101	1158	329	363	338	77	16370	2711	238	134	654	1465	322	2114	110	558	6597	1293	174
	2008	22918	6862	492	1943	2107	1270	257	380	339	74	16056	2806	247	139	1022	1527	334	2107	152	584	5768	1196	175
	2009	22337	6848	488	1890	2107	1346	212	393	340	71	15489	2809	276	155	615	1733	374	2261	36	662	5334	1059	177
	2010	22063	6846	486	1898	2099	1359	200	393	340	70	15217	2937	291	164	637	1850	395	2294	108	709	4697	957	177
	2011	22614	6863	488	1964	2103	1290	219	384	344	70	15751	2994	306	173	647	1961	415	2315	935	756	4213	857	179
	2012	21699	6903	493	2037	2127	1195	245	383	352	71	14796	2776	319	180	887	2061	433	2318	212	801	3853	773	183
	2013	22258	6974	501	2083	2179	1113	264	397	365	72	15284	3163	316	179	837	2051	430	2209	839	804	3485	790	181
	2014	21317	6987	504	2100	2199	1068	263	408	375	70	14330	2874	332	188	721	2179	452	2224	191	860	3408	724	177
	2015	21029	6993	504	2106	2208	1049	255	418	384	69	14036	2863	332	188	732	2188	452	2131	186	871	3212	709	174
Chad	2000	73873	18910	1497	5222	5956	2555	1574	885	1046	175	54963	14307	1172	624	3127	6594	1335	14158	3920	2671	4683	1830	542
	2001	79235	19326	1536	5397	6075	2633	1530	917	1053	184	59909	14621	1200	639	4823	6729	1372	13955	6427	2756	4817	1993	576
	2002	81191	19822	1587	5549	6220	2759	1484	960	1066	199	61369	15828	1304	693	3334	7322	1497	14578	5809	3010	5255	2142	597
	2003	82015	20376	1644	5707	6381	2914	1419	1015	1082	213	61639	15939	1317	700	4984	7357	1519	14165	4363	3058	5336	2272	628
	2004	86174	20908	1696	5910	6506	3074	1339	1066	1094	224	65266	16523	1374	729	5010	7630	1600	14175	6471	3197	5525	2372	660
	2005	84527	21348	1739	6087	6612	3189	1286	1100	1104	231	63179	17082	1429	758	5062	7914	1681	14151	3026	3332	5633	2439	671
	2006	84301	21800	1783	6255	6749	3256	1271	1128	1120	238	62501	18192	1530	814	3478	8505	1821	14551	1104	3578	5819	2452	658
	2007	84462	22161	1821	6339	6920	3270	1263	1166	1142	242	62301	18047	1525	813	5234	8405	1833	13910	310	3587	5499	2441	696
	2008	85213	22499	1856	6403	7085	3298	1250	1200	1163	244	62714	18341	1634	874	3392	9006	1980	14300	588	3878	5599	2393	729
	2009	84793	22727	1882	6445	7184	3382	1195	1218	1175	245	62066	17788	1625	870	4944	8911	1985	13599	102	3892	5310	2304	736
	2010	85073	22964	1903	6566	7226	3474	1150	1217	1181	247	62109	17502	1679	900	4720	9232	2067	13459	263	4063	5287	2217	719
	2011	85643	23086	1912	6678	7226	3514	1110	1213	1184	249	62557	17820	1685	906	4834	9226	2092	12930	887	4124	5167	2141	744
	2012	84899	23220	1923	6830	7228	3512	1077	1208	1190	251	61679	17480	1722	927	4616	9446	2155	12630	551	4265	5170	1990	726
	2013	83810	23414	1941	6911	7289	3549	1042	1220	1210	252	60396	17460	1746	940	4197	9568	2200	12224	73	4376	5150	1746	716
	2014	83343	23606	1959	6965	7358	3601	1000	1236	1234	253	59737	17431	1740	937	4182	9521	2209	11645	171	4412	5064	1719	707
	2015	82728	23789	1977	6974	7445	3662	957	1258	1264	252	58939	17309	1737	935	4131	9484	2221	11103	168	4454	5005	1696	697
Comoros	2000	2019	849	59	315	221	130	26	42	48	9	1170	314	29	16	64	185	41	240	0	82	174	0	25
	2001	2051	864	59	312	225	142	23	45	50	9	1187	323	30	16	66	193	42	240	0	84	168	0	25
	2002	2086	874	60	305	228	154	19	49	50	8	1212	332	31	17	67	205	44	239	0	87	167	0	23
	2003	2110	881	60	300	231	161	16	54	51	8	1229	340	32	17	69	211	46	238	0	89	162	0	25
	2004	2123	887	60	302	232	162	15	56	52	8	1236	343	32	17	70	216	46	232	0	90	162	0	27
	2005	2125	898	61	309	233	162	14	57	54	8	1227	342	32	17	69	217	47	224	0	91	161	0	28
	2006	2118	903	61	317	232	160	15	56	55	8	1215	340	32	17	69	218	47	215	0	91	157	0	28
	2007	2109	909	61	325	232	156	15	55	57	8	1200	337	32	17	69	219	47	205	0	91	155	0	28
	2008	2097	909	60	331	230	152	14	54	59	8	1188	335	32	17	69	220	47	196	0	92	151	0	28
	2009	2075	910	60	336	229	149	14	54	61	8	1165	310	32	18	58	228	49	194	0	95	151	0	30
	2010	2055	914	60	340	229	148	13	54	63	8	1141	270	31	17	45	220	47	179	0	93	207	1	29
	2011	2027	912	59	340	227	146	13	54	65	7	1115	284	34	19	46	243	51	186	0	101	115	6	30
	2012	2004	910	59	341	224	145	12	54	67	7	1094	260	31	17	42	224	48	165	0	95	163	20	30
	2013	1970	907	58	342	222	143	11	54	69	7	1063	252	30	17	41	217	46	153	0	92	167	20	28
	2014	1937	898	57	341	218	141	10	53	71	7	1039	251	30	17	42	216	46	146	0	92	151	20	28
	2015	1897	889	56	339	214	138	10	52	73	7	1008	243	29	16	40	211	45	136	0	90	150	20	27
Congo	2000	14274	3890	270	1409	1177	438	77	252	239	28	10384	1933	200	103	281	1291	327	1691	1042	463	1978	910	165
	2001	14853	3923	271	1420	1176	461	71	253	244	28	10930	2004	208	108	293	1351	344	1694	1338	483	2034	901	171
	2002	15145	3901	268	1408	1157	484	62	249	248	26	11244	2010	210	109	296	1367	350	1630	1724	493	2007	876	170
	2003	13997	3846	262	1381	1124	508	53	241	254	24	10151	1973	207	108	293	1351	349	1525	951	496	1888	842	167
	2004	13319	3759	253	1349	1076	527	45	228	260	22	9560	1923	203	106	290	1329	346	1411	801	498	1695	798	160
	2005	12007	3698	246	1318	1042	540	39	222	272	20	8309	1857	197	103	283	1285	340	1289	89	498	1450	757	161
	2006	11370	3620	237	1279	1002	549	33	215	287	18	7750	1775	189	100	272	1236	330	1162	133	494	1181	725	154
	2007	10733	3537	229	1236	965	550	28	210	303	17	7196	1695	181	96	260	1179	319	1047	140	489	955	679	155
	2008	9950	3464	221	1201	927	551	24	202	322	15	6486	1600	171	91	243	1117	304	933	14	480	776	607	150
	2009	9367	3377	212	1163	890	539	21	195	341	14	5990	1300	172	91	163	1121	308	881	43	497	686	575	152
	2010	8832	3282	203	1123	854	524	19	189	357	14	5550	1205	161	86	148	1040	291	780	51	479	585	571	153
	2011	8520	3209	197	1091	826	509	17	184	372	13	5311	1126	154	82	133	986	279	708	164	468	519	535	156

U5D[1]: total number of under 5 death; NEO[2]: total number of neonatal death; PNE[3]: pneumonia; PRE[4]: preterm; INT[5]: intrapartum related events; SEP[6]: sepsis/meningitis; TET[7]: tetanus; OTH[8]: other disorders; CON[9]: congenital; DIA[10]: diarrea; POS[11]: total number of post neonatal death; MEN[12]: meningitis; MEA[13]: measles; INJ[14]: injury; MAL[15]: malaria; PER[16]: pertussis

			0-28 days									1-59 months												
	Year	U5D[1]	NEO[2]	PNE[3]	PRE[4]	INT[5]	SEP[6]	TET[7]	OTH[8]	CON[9]	DIA[10]	POS[11]	PNE[3]	PRE[4]	INT[5]	MEN[12]	OTH[8]	CON[9]	DIA[10]	MEA[13]	INJ[14]	MAL[15]	AIDS	PER[16]
	2012	8179	3119	189	1055	797	489	15	179	382	12	5060	1059	150	80	121	949	273	656	156	464	474	517	161
	2013	7862	3063	185	1032	777	474	14	175	393	12	4799	863	153	82	79	982	281	639	142	481	462	481	153
	2014	7502	3011	181	1012	759	461	13	172	402	11	4491	823	149	80	72	962	277	547	52	476	435	473	145
	2015	7269	2948	176	988	738	448	13	168	407	11	4321	795	145	78	69	934	270	509	50	467	410	455	140
CÙte d'Ivoire	2000	95582	33147	2300	10422	9531	5908	1230	1855	1575	325	62435	6998	656	339	1143	3843	911	6424	3786	1526	31481	4640	687
	2001	96063	33168	2296	10382	9525	6042	1136	1881	1587	319	62895	6907	647	336	1138	3810	902	6118	3805	1522	32349	4673	689
	2002	95546	32987	2278	10357	9470	6016	1071	1886	1596	312	62559	6756	634	331	1123	3729	888	5769	3861	1506	32646	4618	698
	2003	93652	32558	2240	10295	9319	5936	1009	1863	1592	304	61094	6611	620	325	1108	3656	872	5435	3272	1493	32482	4515	705
	2004	91142	32138	2204	10227	9169	5871	943	1836	1592	295	59004	6440	604	319	1659	3579	855	5094	2352	1475	31565	4371	691
	2005	87971	31670	2154	10166	8937	5894	872	1780	1584	284	56301	6501	609	323	1679	3668	866	4942	873	1512	30535	4131	664
	2006	85464	31286	2109	10025	8746	6021	779	1752	1582	271	54178	6677	627	333	1721	3824	895	4880	57	1576	28975	3965	648
	2007	83842	30916	2056	9785	8531	6311	662	1736	1581	254	52926	7070	665	354	1187	4106	954	4964	218	1696	27393	3666	653
	2008	81932	30786	2024	9547	8436	6614	555	1763	1611	236	51146	7411	698	372	1900	4358	1004	4986	364	1812	24694	2890	659
	2009	80472	30766	2013	9486	8408	6703	503	1778	1651	225	49706	6850	795	425	973	5091	1150	5451	189	2095	23423	2615	649
	2010	79778	30841	2017	9600	8433	6595	494	1774	1707	221	48937	7313	863	462	1028	5595	1251	5678	466	2305	20903	2415	658
	2011	79376	30917	2018	9757	8463	6429	497	1769	1764	220	48459	8215	914	490	1224	5888	1332	5777	822	2472	18137	2432	755
	2012	78010	31068	2022	9899	8457	6417	484	1751	1822	216	46942	8190	968	520	1763	6371	1416	5874	224	2653	16031	2239	693
	2013	77305	31241	2026	9983	8477	6431	463	1758	1891	212	46064	8540	1008	542	1497	6704	1480	5868	343	2803	14692	1910	678
	2014	76127	31362	2023	10058	8462	6457	440	1755	1959	207	44765	8878	1019	548	1602	6822	1501	5687	154	2873	13419	1602	661
	2015	75393	31467	2023	10128	8469	6431	422	1765	2025	203	43926	8919	1027	552	1604	6915	1517	5506	152	2929	12584	1572	649
Democratic Republic of the Congo	2000	346592	83222	5758	30643	25315	8905	2983	4592	4254	771	263370	36051	2932	1725	9472	22097	3631	34033	37279	7432	100577	5313	2827
	2001	347414	84739	5853	30971	25732	9596	2841	4634	4356	755	262675	35048	2863	1681	14219	21558	3568	31798	36440	7335	99925	5318	2922
	2002	359243	86510	5982	30895	26322	10722	2599	4762	4496	733	272733	34666	2846	1668	14118	21528	3568	30204	46181	7371	102353	5297	2935
	2003	346580	88548	6095	31116	26845	11885	2352	4901	4645	709	258032	34069	2815	1647	13852	21336	3561	28483	31453	7367	105251	5238	2959
	2004	361888	90322	6168	31463	27147	12980	2140	4965	4776	682	271566	34097	2837	1656	9031	21660	3622	27348	45044	7501	110738	5153	2880
	2005	349020	91602	6196	31916	27255	13719	1990	4970	4904	652	257418	32840	2750	1603	13242	20983	3546	25248	31846	7357	110098	5030	2875
	2006	329903	91704	6156	31863	27100	14192	1818	4959	5002	615	238199	33602	2831	1656	8811	21741	3699	24749	14159	7671	111439	4965	2876
	2007	322918	91903	6123	31559	26992	14930	1599	5009	5114	577	231015	33785	2863	1682	13484	22174	3794	23855	9237	7853	104595	4901	2794
	2008	315896	91993	6077	31129	26871	15654	1391	5098	5230	542	223903	35745	3043	1798	14194	23770	4087	24205	4681	8456	96287	4774	2862
	2009	312197	92046	6052	30863	26796	16019	1250	5176	5376	514	220151	34601	3565	2122	7298	28493	4857	27074	3901	10043	90701	4692	2804
	2010	310210	92134	6043	31187	26759	15678	1230	5174	5564	500	218076	39054	3931	2343	8560	31582	5412	28491	3775	11194	76229	4523	2981
	2011	331052	92409	6076	31689	26831	15112	1246	5167	5798	490	238643	41387	4370	2615	8588	35678	6078	30291	26594	12603	63050	4533	2855
	2012	315540	92723	6083	32070	26806	14884	1230	5153	6019	479	222817	43059	4790	2876	8606	39377	6740	31729	12857	13965	51655	4253	2911
	2013	312817	93345	6100	32231	26863	15053	1163	5209	6262	463	219472	42774	5068	3043	9644	41900	7207	32036	12274	14923	44054	3698	2852
	2014	308095	93669	6093	32280	26797	15268	1087	5235	6461	448	214426	40153	5419	3254	7941	45012	7787	32752	9679	16076	40190	3345	2817
	2015	304558	94252	6108	32437	26845	15377	1038	5290	6722	435	210306	40247	5481	3291	7932	45571	7959	31631	9448	16408	36293	3281	2763
Djibouti	2000	2309	961	64	348	266	90	42	71	62	16	1348	303	28	14	54	212	37	254	261	79	15	88	4
	2001	2133	959	64	351	262	94	39	70	63	16	1174	294	27	14	52	208	36	238	114	78	15	93	4
	2002	2063	955	63	353	257	101	35	68	64	15	1108	287	26	14	51	205	36	223	76	77	15	94	4
	2003	2004	946	62	349	251	108	31	67	65	14	1058	281	26	13	50	202	35	210	53	76	15	92	4
	2004	2014	936	60	341	246	117	27	66	66	13	1078	275	26	13	49	199	35	198	106	75	8	89	4
	2005	2203	922	59	329	241	125	23	66	66	12	1281	267	25	13	47	194	35	185	344	74	7	87	4
	2006	1864	899	57	313	235	130	20	66	67	11	965	259	24	13	45	190	34	173	63	73	5	81	4
	2007	1769	871	55	298	228	131	18	64	67	10	898	239	24	13	40	190	35	165	38	73	3	75	4
	2008	1828	843	53	287	221	128	16	62	68	9	985	202	25	13	28	197	36	164	168	76	2	70	4
	2009	1652	816	51	279	213	123	15	60	68	8	836	193	24	13	26	189	35	150	59	74	1	67	4
	2010	1564	795	49	273	206	119	13	59	68	8	769	186	24	12	25	183	34	139	24	72	1	64	4
	2011	1564	782	48	269	202	116	12	58	69	7	782	182	23	12	25	179	34	131	62	71	2	57	4
	2012	1646	772	48	267	199	113	11	57	71	7	874	184	23	12	25	179	34	125	174	71	4	40	4
	2013	1480	766	47	266	196	110	11	56	72	7	714	145	24	13	15	189	36	127	33	76	7	45	4
	2014	1469	752	46	262	192	107	10	55	73	6	717	145	24	12	16	185	36	118	53	74	4	47	4
	2015	1429	737	45	258	187	104	9	54	74	6	692	140	23	12	15	180	35	110	51	73	4	45	4
Equatorial Guinea	2000	3197	920	66	292	275	111	37	73	47	20	2277	421	43	22	45	256	69	390	274	90	607	38	23
	2001	2992	921	65	300	275	105	36	73	47	19	2071	404	42	21	41	246	68	360	99	87	632	49	24
	2002	3068	922	65	304	275	104	35	73	48	19	2146	378	40	20	37	228	65	323	214	83	671	61	26
	2003	3053	922	65	303	275	106	32	74	49	18	2131	346	37	18	32	208	62	284	247	77	714	78	27
	2004	3029	922	65	301	275	108	30	75	50	17	2107	323	35	17	29	194	60	254	266	73	733	96	27
	2005	3086	920	65	303	274	107	28	76	51	16	2166	317	35	17	28	191	60	239	360	73	709	109	28
	2006	2813	920	65	305	273	107	26	76	52	16	1893	335	38	18	28	205	65	242	113	78	619	123	29
	2007	2896	923	65	306	274	109	24	77	54	15	1973	363	41	20	30	225	73	251	218	86	495	140	30
	2008	3048	933	65	305	278	113	21	80	56	14	2115	386	44	21	31	242	80	256	395	94	381	153	32
	2009	2973	943	65	304	281	117	19	84	59	14	2030	397	46	22	32	250	84	253	340	98	292	183	33
	2010	2650	950	65	306	282	120	18	86	61	13	1700	399	47	22	33	254	86	244	43	100	253	187	33
	2011	2747	952	65	307	281	120	17	86	63	12	1795	390	46	22	33	249	86	229	174	99	233	203	34
	2012	3132	953	65	309	280	119	16	86	65	12	2179	373	44	21	32	238	84	210	605	96	220	221	34
	2013	2696	950	65	310	278	119	15	85	67	11	1746	377	45	21	33	244	87	204	219	99	228	155	33
	2014	2701	946	64	310	276	118	15	85	69	11	1755	364	47	22	30	255	91	202	251	103	234	122	33
	2015	2655	944	64	311	274	117	14	84	71	10	1711	356	46	22	30	251	91	190	244	103	229	119	32
Eritrea	2000	13462	3262	252	840	1004	678	40	185	242	21	10200	2152	174	96	436	1006	200	1527	3580	594	35	239	161
	2001	10843	3344	253	826	1032	722	33	197	262	19	7499	2107	171	94	431	990	198	1429	964	595	108	252	162
	2002	10637	3467	259	844	1067	756	32	205	285	18	7170	2134	173	96	441	1003	203	1383	671	615	19	262	170
	2003	10640	3592	265	868	1098	791	31	214	308	18	7048	2132	174	97	447	1000	204	1323	568	627	29	267	179
	2004	10213	3675	269	884	1115	813	29	219	328	18	6538	2139	175	98	454	1005	208	1271	89	640	8	265	185
	2005	10177	3693	268	887	1114	815	27	220	344	18	6484	2122	174	98	457	996	208	1209	117	647	15	248	192
	2006	10485	3724	267	893	1117	818	26	224	361	18	6761	2114	173	98	461	996	210	1155	460	656	9	233	197
	2007	10065	3680	262	882	1099	801	24	223	372	17	6385	2081	171	97	460	982	209	1091	219	657	10	208	201
	2008	9716	3617	256	861	1077	781	22	223	381	16	6099	1697	191	109	316	1126	234	1167	126	744	6	181	202
	2009	9605	3548	248	836	1053	764	20	223	388	15	6057	1645	185	107	309	1093	229	1088	295	732	11	159	204
	2010	9219	3451	239	802	1019	746	17	223	390	14	5768	1588	179	104	299	1056	223	1011	194	717	48	144	205
	2011	8956	3357	229	773	984	729	16	222	390	14	5599	1543	174	102	290	1022	219	947	220	706	41	124	212
	2012	9144	3271	220	751	952	711	14	220	390	13	5873	1509	168	98	286	977	212	877	708	687	31	101	219
	2013	8379	3186	212	734	920	689	13	215	390	12	5193	1449	162	95	274	948	206	812	254	668	31	85	208
	2014	7998	3112	206	720	893	670	13	210	389	12	4886	1404	157	92	265	929	201	733	138	654	41	71	200
	2015	7764	3056	200	711	870	655	12	206	390	12	4708	1353	152	89	255	904	195	682	135	637	45	69	193
Ethiopia	2000	416657	141366	12152	34161	45642	19982	13437	6831	7796	1364	275291	74180	6706	2166	28865	31519	6086	63172	16280	16203	11708	14489	3917
	2001	402636	138779	12021	33106	45208	20788	11584	6892	7841	1340	263857	73146	6627	2152	28339	31035	6057	59449	12812	16348	9788	14152	3950
	2002	400727	135084	11755	31867	44261	21543	9584	6915	7863	1297	265643	69847	6347	2071	26909	29378	5848	54036	27279	16026	10296	13540	4066
	2003	365200	130274	11357	30470	42774	22099	7613	6866	7864	1231	234926	63406	5783	1895	24234	26431	5377	46573	12307	14981	17255	12716	3968
	2004	337975	124779	10874	28958	40913	22430	5838	6748	7874	1145	213196	62183	5701	1876	23446	25850	5361	43276	7623	15168	6986	11721	4007
	2005	314236	119208	10345	27479	38865	22441	4543	6566	7917	1053	195028	58373	5384	1778	21633	24102	5126	38443	6453	14723	4402	10645	3967
	2006	297848	113488	9803	25985	36768	22029	3603	6330	8005	965	184360	56242	5223	1727	13363	23163	5034	35028	12650	14679	3918	9410	3922
	2007	268832	108592	9321	24679	34926	21560	2932	6105	8182	887	160240	47796	5168	1713	10425	22745	5052	32535	5067	14916	2751	8177	3896
	2008	269416	103658	8829	23334	33082	21011	2377	5860	8356	808	165758	42293	4677	1552	13571	20190	4643	27660	24895	13827	1860	6842	3749
	2009	244494	99556	8388	22176	31478	20607	1960	5656	8551	741	144938	39681	4503	1494	8211	19137	4540	25046	16467	13586	2714	5836	3723
	2010	223069	96265	8059	21582	30124	19870	1789	5393	8742	706	126804	36287	4241	1405	7290	17776	4340	22264	8204	13008	3497	4901	3592
	2011	209070	93980	7854	21475	29152	18867	1803	5138	8990	701	115090	33497	4144	1373	6520	17104	4302	20597	5239	12895	1693	4099	3628
	2012	200968	92167	7721	21738	28431	17473	1958	4893	9236	718	108801	29783	4104	1359	5518	16686	4325	19363	5435	12904	2027	3588	3708
	2013	195749	90094	7543	21613	27705	16443	1951	4707	9420	711	105655	26666	4075	1350	5505	16584	4360	18286	6883	12918	2687	2818	3524
	2014	190132	88851	7405	21503	27210	15892	1890	4601	9648	700	101281	25192	4153	1376	4956	16967	4510	16132	7154	13254	2198	2006	3383
	2015	184186	87414	7227	21160	26632	15625	1740	4527	9826	676	96772	24119	4015	1330	4715	16305	4426	14865	6837	12890	2121	1916	3232
Gabon	2000	3447	1197	77	490	310	140	18	60	93	8	2250	321	38	18	31	199	70	255	513	90	290	376	49
	2001	4455	1199	78	486	312	142	18	61	96	8	3256	334	39	19	33	208	74	256	1478	94	263	406	51
	2002	4084	1193	76	475	312	145	17	63	98	8	2891	346	41	20	34	217	78	257	1091	99	228	430	52
	2003	3948	1181	76	467	309	145	16	63	99	8	2767	357	42	20	36	227	82	256	956	103	189	446	52
	2004	3766	1180	75	463	308	146	15	63	101	7	2586	365	44	21	37	233	85	253	787	106	149	454	53
	2005	3016	1186	76	464	309	148	15	63	105	7	1830	370	44	22	37	237	88	247	46	109	121	455	54
	2006	3076	1194	76	460	311	153	14	65	109	7	1882	373	45	22	38	239	90	240	112	111	103	455	55

U5D[1]: total number of under 5 death; NEO[2]: total number of neonatal death; PNE[3]: pneumonia; PRE[4]: preterm; INT[5]: intrapartum related events; SEP[6]: sepsis/meningitis; TET[7]: tetanus; OTH[8]: other disorders; CON[9]: congenital; DIA[10]: diarrea; POS[11]: total number of post neonatal death; MEN[12]: meningitis; MEA[13]: measles; INJ[14]: injury; MAL[15]: malaria; PER[16]: pertussis

			0-28 days										1-59 months											
	Year	U5D[1]	NEO[2]	PNE[3]	PRE[4]	INT[5]	SEP[6]	TET[7]	OTH[8]	CON[9]	DIA[10]	POS[11]	PNE[3]	PRE[4]	INT[5]	MEN[12]	OTH[8]	CON[9]	DIA[10]	MEA[13]	INJ[14]	MAL[15]	AIDS	PER[16]
	2007	3114	1207	78	457	314	160	13	65	113	7	1907	373	45	22	38	246	92	231	169	112	95	436	49
	2008	2947	1222	78	455	317	167	12	67	119	7	1725	370	45	22	38	243	93	220	38	113	93	401	50
	2009	2948	1228	77	451	317	172	11	69	124	6	1720	369	45	22	37	241	94	210	70	115	96	368	52
	2010	2953	1235	77	451	317	175	10	69	129	6	1718	318	48	24	27	252	100	212	118	122	110	332	56
	2011	2945	1233	76	447	315	177	10	69	134	6	1712	311	48	24	26	254	101	204	154	124	122	290	55
	2012	2911	1228	75	440	311	179	9	69	138	6	1683	300	47	23	24	251	101	192	188	124	136	242	54
	2013	2697	1223	74	430	309	183	8	70	143	6	1474	295	46	23	24	247	99	180	57	123	146	183	51
	2014	2630	1218	74	423	306	184	7	71	148	5	1412	291	45	22	24	238	96	165	72	120	150	142	48
	2015	2579	1195	72	413	299	181	7	70	148	5	1384	283	44	22	23	232	95	155	71	118	155	139	47
Gambia	2000	6327	2260	150	585	672	551	30	138	124	10	4067	851	106	53	148	647	147	940	272	268	478	106	52
	2001	6166	2253	149	584	668	548	28	139	127	10	3913	846	105	52	224	642	148	888	139	269	427	124	50
	2002	6066	2274	150	587	673	554	26	142	132	10	3792	855	108	54	149	657	153	869	85	279	389	141	53
	2003	6026	2291	151	593	676	555	25	144	138	9	3735	846	108	54	147	660	156	832	97	284	342	156	53
	2004	5877	2315	152	601	682	557	24	146	144	9	3562	836	109	54	144	659	158	795	3	287	293	170	55
	2005	5837	2314	152	605	680	550	24	145	150	9	3523	828	109	54	142	663	160	762	15	293	260	182	55
	2006	5800	2307	151	605	677	542	22	145	155	9	3493	826	109	55	142	663	162	730	26	297	236	191	57
	2007	5754	2312	151	609	679	535	22	146	162	9	3442	818	109	55	141	657	164	692	35	298	218	198	58
	2008	5697	2330	152	615	684	532	21	148	169	9	3367	804	108	55	137	656	165	662	16	301	207	197	59
	2009	5671	2351	154	623	689	528	20	150	177	9	3320	794	108	55	134	655	166	632	25	304	197	187	60
	2010	5658	2374	155	629	695	529	20	152	185	9	3284	684	117	59	103	708	181	652	34	331	205	148	63
	2011	5646	2411	157	644	702	532	20	152	195	9	3235	653	118	60	95	715	183	632	41	338	202	133	66
	2012	5616	2443	157	663	705	535	21	151	202	9	3173	606	119	61	121	728	188	615	12	346	201	109	67
	2013	5610	2464	158	683	705	529	22	147	210	9	3146	612	120	62	83	735	191	588	17	351	199	122	66
	2014	5573	2477	159	696	704	525	22	145	218	9	3096	616	122	62	85	746	194	499	12	358	199	137	65
	2015	5540	2471	158	701	699	517	21	143	224	9	3069	612	121	62	84	746	196	478	12	360	197	135	65
Ghana	2000	66835	22698	1602	6868	6691	4248	429	1264	1436	160	44137	6345	564	342	1146	3362	837	5019	7476	1660	14563	2193	629
	2001	63946	22794	1600	6960	6665	4245	404	1279	1479	161	41152	6532	582	359	1172	3460	882	4966	4869	1736	13683	2239	672
	2002	63284	22974	1607	7034	6697	4254	380	1305	1536	161	40310	5786	640	402	881	3864	992	5230	4105	1928	13529	2263	691
	2003	59528	23424	1630	7095	6831	4386	355	1353	1617	157	36104	5942	661	422	1367	4010	1050	5176	61	2009	12433	2265	707
	2004	60033	23839	1644	7074	6969	4573	328	1405	1700	147	36194	6212	694	451	1422	4250	1130	5226	247	2131	11458	2244	728
	2005	60544	24295	1660	7038	7108	4808	304	1459	1780	138	36249	6375	726	481	1435	4465	1214	5246	460	2241	10667	2197	743
	2006	60750	24642	1669	7039	7201	4961	288	1502	1850	132	36108	6603	765	504	973	4690	1304	5258	567	2352	10203	2132	755
	2007	60026	24815	1669	7081	7210	5022	276	1522	1906	129	35211	6488	785	516	923	4828	1363	5140	192	2409	9932	1906	729
	2008	59528	24950	1668	7176	7196	5017	269	1532	1962	129	34578	6331	772	506	1354	4701	1361	4816	337	2368	9610	1684	737
	2009	58792	25063	1665	7257	7184	4999	262	1542	2027	127	33729	6223	771	504	866	4667	1383	4578	377	2367	9621	1633	739
	2010	58117	25203	1665	7305	7197	4995	253	1556	2108	124	32914	6098	763	496	836	4585	1383	4317	476	2353	9389	1477	741
	2011	57262	25373	1664	7338	7229	5006	241	1573	2204	119	31889	6192	778	503	843	4649	1431	4183	206	2406	8873	1074	752
	2012	57080	25617	1669	7406	7273	5027	230	1589	2309	114	31463	5672	832	536	1067	4993	1553	3965	455	2583	8271	778	759
	2013	56434	25721	1663	7448	7262	5021	219	1595	2403	110	30713	5145	898	578	698	5435	1702	3842	373	2800	7732	767	742
	2014	55364	25709	1650	7481	7206	4990	208	1586	2480	107	29655	4922	930	599	612	5654	1793	3730	122	2914	6968	689	722
	2015	54061	25402	1617	7434	7062	4902	196	1560	2528	103	28659	4861	931	600	603	5658	1823	3559	117	2928	6216	666	698
Guinea	2000	66725	17419	1243	4654	5323	3666	554	964	862	153	49306	7508	544	482	1620	3945	879	7207	9760	1499	14342	1084	435
	2001	63983	16737	1195	4497	5099	3595	443	944	819	144	47246	7490	544	482	1612	3960	882	6850	8307	1534	14061	1089	435
	2002	57874	16290	1163	4377	4960	3549	371	937	797	135	41584	7422	541	480	1597	3946	884	6468	3680	1559	13494	1080	433
	2003	55593	16018	1140	4271	4883	3551	314	942	791	126	39575	7344	536	477	1586	3930	882	6100	2905	1581	12737	1066	433
	2004	51412	15780	1118	4125	4839	3570	265	953	795	115	35632	7326	536	477	1589	3946	887	5806	62	1615	11911	1048	428
	2005	50643	15577	1096	3976	4801	3610	220	966	804	104	35066	7317	537	479	1594	3960	893	5537	456	1650	11180	1030	434
	2006	49689	15150	1054	3797	4672	3597	177	960	800	93	34539	7311	538	480	1600	3980	901	5289	701	1684	10611	1004	440
	2007	47981	14689	1010	3646	4508	3567	145	938	793	82	33292	7290	538	480	1598	3997	906	5042	154	1715	10198	936	438
	2008	47349	14418	978	3589	4381	3561	128	912	793	75	32931	7009	519	464	2353	3834	879	4640	527	1682	9664	903	458
	2009	46801	14250	958	3611	4287	3519	124	878	802	71	32551	6332	547	490	1258	4082	931	4678	893	1805	10157	912	466
	2010	45310	14261	960	3706	4284	3424	133	857	828	70	31049	6101	539	483	1192	4031	921	4406	173	1808	10035	901	461
	2011	44874	14261	966	3806	4311	3252	146	846	863	70	30613	5993	529	474	1170	3953	907	4137	461	1803	9873	840	473
	2012	44514	14280	973	3871	4356	3101	154	855	901	69	30234	5933	525	471	1154	3929	905	3935	651	1815	9623	811	481
	2013	43437	14337	978	3914	4388	3032	154	864	939	68	29100	5953	528	474	1154	3985	915	3793	154	1851	9396	428	470
	2014	42802	14270	970	3907	4359	2994	146	864	965	65	28532	6111	526	472	1212	3988	914	3618	155	1867	8991	219	461
	2015	42073	14288	965	3929	4339	2993	140	865	995	63	27785	6053	523	469	1199	3986	913	3446	150	1879	8507	213	449
Guinea-Bissau	2000	9267	2848	208	892	790	446	211	139	130	31	6419	1316	118	63	266	676	157	1306	807	256	1239	157	57
	2001	8668	2821	207	876	787	464	186	142	128	31	5847	1359	122	66	276	704	164	1291	314	270	1037	186	57
	2002	8748	2810	207	868	785	485	165	144	126	30	5938	1391	126	68	284	726	171	1264	524	282	830	215	58
	2003	10504	2799	206	866	779	500	147	146	125	30	7705	1411	128	69	289	742	177	1226	2427	293	642	243	57
	2004	9665	2818	207	872	781	516	138	148	126	29	6847	1408	129	70	290	744	179	1170	1752	299	478	270	58
	2005	7961	2825	207	860	787	539	123	152	128	28	5136	1384	127	69	286	735	179	1098	228	301	376	295	58
	2006	7658	2826	207	827	798	573	103	159	133	26	4832	1347	124	68	279	717	178	1020	114	300	316	309	59
	2007	7331	2804	204	769	810	611	79	169	138	24	4527	1299	121	66	268	694	176	939	5	296	281	322	58
	2008	7160	2782	201	727	823	625	67	175	144	21	4378	1244	116	64	256	666	170	859	50	291	262	342	58
	2009	6964	2752	198	702	831	614	62	175	150	19	4212	1023	123	68	183	708	182	860	73	312	275	348	56
	2010	6745	2719	196	697	834	585	64	168	156	18	4026	984	119	65	176	682	177	791	48	308	270	349	57
	2011	6579	2686	194	692	830	564	62	164	162	17	3893	946	115	63	169	658	173	727	73	303	264	344	57
	2012	6409	2651	191	684	822	550	58	161	168	16	3758	916	112	62	164	639	170	672	85	300	258	325	56
	2013	6185	2626	188	678	812	546	53	160	173	16	3559	893	110	60	160	629	168	628	26	299	253	279	54
	2014	6028	2608	186	676	803	542	49	158	179	15	3420	875	108	60	157	621	167	590	32	299	246	214	52
	2015	5883	2586	183	673	792	537	46	156	183	15	3297	847	105	58	152	605	165	548	31	294	235	206	50
Kenya	2000	125108	33956	2612	9776	11012	5374	495	2260	2211	217	91152	18716	1823	925	3412	10228	2654	14341	3372	4745	8852	20873	1212
	2001	123779	35326	2705	10114	11443	5617	515	2338	2374	220	88453	18574	1809	926	3383	10114	2653	13661	2401	4788	8660	20215	1270
	2002	121138	36404	2772	10280	11797	5867	518	2411	2543	216	84734	15609	1948	1004	2445	10992	2877	14086	1159	5250	8743	19338	1285
	2003	117689	37413	2828	10422	12145	6065	520	2486	2737	210	80276	15709	1895	983	2520	10620	2813	13127	12	5219	7697	18304	1376
	2004	114783	38215	2867	10507	12399	6253	519	2529	2939	202	76568	15376	1860	971	2455	10361	2779	12297	212	5234	6521	17079	1423
	2005	111088	38793	2886	10552	12533	6434	513	2533	3147	194	72295	14599	1784	937	3485	9863	2684	11238	432	5140	5294	15386	1452
	2006	107511	38812	2857	10432	12441	6603	489	2495	3312	183	68699	14134	1754	925	3333	9679	2656	10531	663	5168	4514	13889	1452
	2007	103446	38748	2821	10272	12320	6762	458	2463	3480	172	64698	13853	1735	917	2153	9524	2642	9920	718	5221	4020	12518	1477
	2008	96776	38304	2751	9981	12042	6876	419	2384	3693	159	58472	13204	1679	889	2032	9177	2564	9091	647	5208	3677	8827	1477
	2009	92297	37827	2691	9781	11802	6835	392	2323	3853	150	54470	12688	1613	855	1951	8750	2477	8315	633	5109	3495	7093	1489
	2010	88067	37309	2638	9637	11596	6641	376	2272	4005	144	50758	12119	1538	816	1858	8274	2368	7574	245	4968	3426	6070	1503
	2011	84260	36539	2579	9472	11368	6247	367	2213	4155	139	47721	9348	1606	853	1224	8739	2480	7555	486	5290	3766	4893	1483
	2012	81042	35759	2518	9272	11158	5862	354	2186	4275	134	45283	9123	1523	809	1227	8168	2361	6864	402	5097	3802	4376	1531
	2013	78558	35103	2464	9109	10964	5568	338	2171	4359	130	43455	8890	1444	767	1320	7729	2245	6251	171	4888	3802	4472	1476
	2014	76416	34427	2404	8926	10718	5379	319	2138	4417	125	41989	8500	1408	748	1252	7558	2196	5698	254	4820	3840	4292	1423
	2015	74429	33741	2342	8757	10447	5231	301	2093	4450	120	40688	8242	1368	727	1213	7353	2140	5325	248	4730	3804	4159	1379
Lesotho	2000	6934	2224	167	688	692	319	49	155	131	22	4710	920	95	46	164	528	145	726	66	225	0	1739	56
	2001	6889	2135	159	672	662	302	45	149	124	21	4754	943	98	47	166	549	150	730	5	229	0	1781	57
	2002	6835	2034	150	647	626	292	40	143	116	20	4801	955	99	48	167	565	153	723	8	230	0	1800	54
	2003	6744	1935	141	620	590	286	36	136	109	18	4809	955	100	48	166	574	154	708	20	228	0	1805	52
	2004	6624	1858	133	598	562	280	33	131	104	17	4766	951	99	47	166	579	154	688	10	226	0	1793	51
	2005	6563	1814	129	587	546	277	30	128	101	16	4749	951	100	47	166	586	155	671	20	226	0	1777	50
	2006	6519	1796	127	582	539	276	29	128	100	15	4723	956	101	48	167	597	157	656	24	227	0	1740	49
	2007	6351	1817	127	588	544	284	28	128	104	15	4534	958	101	48	168	605	159	632	28	232	0	1555	48
	2008	6295	1866	130	603	558	293	28	131	109	15	4429	969	103	48	169	616	162	616	9	237	0	1452	50
	2009	5866	1919	132	619	572	303	28	130	121	14	3947	815	115	54	110	695	181	643	13	275	0	994	51
	2010	5720	1980	136	641	588	310	29	132	131	14	3740	809	115	54	107	703	183	615	65	283	0	754	52
	2011	5601	2022	138	659	597	313	29	134	139	13	3579	809	116	54	105	715	184	595	52	290	0	606	53
	2012	5578	2051	139	674	602	314	29	135	145	14	3527	820	118	55	105	731	188	580	48	297	0	532	53
	2013	5670	2072	139	690	604	314	28	136	147	13	3598	828	119	56	105	745	190	569	51	301	0	579	54
	2014	5629	2073	138	698	600	312	27	136	150	13	3556	827	120	56	104	752	191	550	22	303	0	578	54
	2015	5570	2045	135	697	587	305	25	133	150	12	3525	822	119	56	102	754	191	529	22	304	0	573	53
Liberia	2000	24061	5301	370	1732	1567	830	194	275	263	71	18760	2482	222	123	753	1409	302	2534	3641	477	6294	366	157
	2001	23320	5260	366	1718	1550	872	165	273	253	63	18060	2472	222	123	752	1408	305	2373	3277	494	6084	387	162

U5D[1]: total number of under 5 death; NEO[2]: total number of neonatal death; PNE[3]: pneumonia; PRE[4]: preterm; INT[5]: intrapartum related events; SEP[6]: sepsis/meningitis; TET[7]: tetanus; OTH[8]: other disorders; CON[9]: congenital; DIA[10]: diarrea; POS[11]: total number of post neonatal death; MEN[12]: meningitis; MEA[13]: measles; INJ[14]: injury; MAL[15]: malaria; PER[16]: pertussis

		0-28 days										1-59 months												
	Year	U5D[1]	NEO[2]	PNE[3]	PRE[4]	INT[5]	SEP[6]	TET[7]	OTH[8]	CON[9]	DIA[10]	POS[11]	PNE[3]	PRE[4]	INT[5]	MEN[12]	OTH[8]	CON[9]	DIA[10]	MEA[13]	INJ[14]	MAL[15]	AIDS	PER[16]
	2002	19922	5062	355	1640	1512	845	140	271	245	54	14860	2396	216	120	732	1371	300	2161	912	500	5593	397	161
	2003	18353	4812	337	1565	1443	802	120	261	238	46	13541	2293	207	116	715	1316	289	1942	684	499	4922	400	159
	2004	16878	4587	319	1489	1373	779	101	251	235	39	12291	2216	200	112	684	1271	283	1763	488	503	4210	397	163
	2005	15428	4420	306	1411	1315	792	82	243	237	34	11008	2191	199	112	660	1276	282	1641	7	518	3580	391	152
	2006	14734	4270	292	1312	1263	831	60	242	241	29	10464	2182	198	112	638	1276	285	1542	134	535	3025	380	156
	2007	14176	4161	281	1224	1221	877	42	246	247	24	10015	2195	201	114	620	1293	291	1468	184	557	2569	365	157
	2008	13545	4090	272	1170	1191	897	32	251	256	21	9455	1973	216	123	459	1410	317	1492	29	615	2337	330	155
	2009	13125	4011	264	1137	1159	891	27	249	265	19	9114	1967	217	124	447	1421	322	1417	81	632	2039	297	152
	2010	13748	3938	256	1118	1131	869	25	246	275	17	9810	1987	210	120	466	1366	314	1300	1202	624	1803	258	159
	2011	12846	3893	251	1109	1107	861	23	241	285	16	8953	1813	210	121	379	1371	317	1237	750	637	1739	221	159
	2012	11770	3821	243	1093	1074	847	22	234	292	15	7949	1741	204	118	357	1334	311	1147	123	631	1647	178	159
	2013	11328	3749	236	1076	1041	836	20	227	299	15	7579	1675	195	113	347	1279	300	1043	156	612	1554	155	151
	2014	10842	3692	231	1063	1017	821	19	222	306	14	7150	1547	192	111	323	1268	299	982	76	612	1463	134	143
	2015	10509	3644	226	1053	995	808	18	218	313	13	6865	1513	190	109	316	1254	297	924	73	609	1314	128	138
Madagascar	2000	71115	20421	1561	6530	6433	2846	439	1158	1280	174	50694	14435	1363	436	2941	8025	1323	11336	4055	3639	1885	413	844
	2001	67654	19945	1520	6297	6268	2854	389	1141	1313	163	47709	13943	1316	424	2851	7736	1282	10420	2983	3610	1872	424	848
	2002	64916	19347	1464	6085	6020	2841	342	1106	1333	156	45569	13177	1242	404	2715	7295	1214	9372	3093	3503	2288	428	838
	2003	64317	18687	1400	5869	5722	2846	299	1058	1344	149	45630	12019	1136	370	2488	6630	1116	8136	5567	3281	3663	428	798
	2004	58469	18109	1342	5660	5439	2895	259	1011	1361	142	40360	11839	1119	369	2454	6533	1109	7626	2816	3319	1959	421	795
	2005	52852	17599	1288	5432	5199	2961	223	976	1386	134	35253	11326	1071	358	2341	6245	1071	6951	14	3259	1431	412	775
	2006	50260	17234	1246	5213	5046	3009	194	963	1438	125	33026	10707	1016	340	2198	5876	1023	6259	137	3164	1143	398	766
	2007	48077	16916	1213	5014	4950	2985	171	964	1503	116	31161	10303	979	331	2105	5627	996	5747	207	3119	589	383	775
	2008	46093	16631	1186	4833	4894	2903	153	978	1576	108	29462	9095	1014	344	1650	5802	1039	5666	98	3289	287	367	812
	2009	44848	16427	1166	4709	4848	2828	141	989	1643	103	28421	8298	1009	339	1394	5753	1038	5369	258	3314	499	350	799
	2010	43773	16363	1155	4643	4823	2798	132	999	1713	99	27410	8077	968	325	1391	5477	1003	4924	356	3222	536	335	797
	2011	42709	16283	1142	4587	4777	2790	124	999	1766	96	26426	7784	943	316	1339	5307	982	4598	153	3174	728	312	790
	2012	42125	16199	1129	4539	4734	2768	117	1000	1818	94	25926	7502	904	303	1310	5033	947	4226	322	3073	1217	293	798
	2013	41574	16105	1117	4497	4688	2737	112	998	1865	93	25469	6153	986	331	875	5562	1040	4424	422	3383	1232	279	784
	2014	40700	16155	1115	4501	4684	2726	108	1004	1925	92	24545	6082	962	323	885	5422	1021	3848	117	3326	1537	268	754
	2015	40075	16189	1111	4503	4670	2721	105	1005	1983	91	23886	5854	930	312	853	5233	994	3573	114	3242	1797	261	724
Malawi	2000	82502	16982	1182	5227	4983	3221	216	1182	825	147	65520	12205	973	749	3221	7595	1554	11315	130	2402	12965	11959	454
	2001	77767	16245	1126	5045	4773	3071	181	1142	782	124	61522	11540	926	713	3056	7289	1497	10033	245	2373	11409	11998	443
	2002	71959	15461	1061	4827	4535	2933	151	1097	756	103	56498	9468	912	702	2125	7177	1493	9160	439	2439	10155	11929	499
	2003	66369	14799	1002	4639	4294	2864	128	1038	748	87	51570	8315	858	662	1738	6790	1426	7988	607	2388	8520	11797	480
	2004	61997	14333	955	4515	4106	2821	113	989	759	76	47664	7611	806	622	1559	6384	1361	6988	756	2319	7155	11618	486
	2005	59020	14206	933	4490	4019	2836	103	966	789	69	44814	7059	766	590	1419	6049	1312	6208	877	2256	6415	11370	494
	2006	57239	14250	927	4525	4000	2847	98	959	829	65	42989	6785	756	584	1319	5985	1319	5782	174	2268	6494	11020	503
	2007	56314	14583	940	4664	4066	2893	98	973	885	65	41731	6696	723	561	1362	5688	1283	5246	302	2202	6699	10421	548
	2008	55401	14990	958	4833	4138	2965	98	983	950	65	40411	6436	711	551	1276	5569	1283	4869	422	2187	7085	9457	565
	2009	54458	15296	969	4971	4177	3018	97	987	1011	65	39162	6368	714	554	1243	5585	1310	4631	84	2223	7228	8638	584
	2010	53219	15383	966	5032	4146	3042	93	978	1062	64	37836	6078	688	536	1182	5362	1286	4232	1317	2170	6290	8096	599
	2011	50363	15092	937	4942	4017	2991	85	943	1116	61	35271	6226	721	564	1186	5668	1369	4175	262	2329	5445	6734	593
	2012	46621	14655	901	4783	3854	2906	76	898	1179	57	31966	4733	789	618	574	6264	1524	4231	365	2617	4670	4997	584
	2013	43405	14252	869	4639	3713	2807	69	862	1240	53	29153	4858	770	603	683	6140	1512	3471	484	2610	3682	3808	533
	2014	41189	13885	840	4517	3586	2710	64	833	1285	50	27304	4719	758	593	658	6052	1512	3217	446	2604	3117	3130	499
	2015	40048	13720	825	4474	3517	2651	60	819	1325	49	26328	4596	747	584	640	5956	1514	3014	427	2582	2767	3018	481
Mali	2000	110885	28796	1991	9234	7556	4475	2365	1283	1604	288	82089	15839	1328	721	3312	8192	1602	17214	3414	2696	25927	1269	575
	2001	113269	28297	1984	9094	7535	4625	1989	1294	1485	290	84972	15838	1333	724	3281	8244	1619	16410	6875	2766	26068	1238	576
	2002	104591	27991	1991	8854	7607	4879	1649	1326	1401	285	76600	15560	1315	714	3200	8137	1608	15323	71	2799	26092	1209	571
	2003	102753	27927	2006	8585	7767	5220	1342	1383	1350	273	74826	14820	1259	682	4635	7763	1549	13842	609	2753	25154	1179	582
	2004	100526	28050	2025	8239	7977	5749	1003	1475	1330	252	72476	14476	1236	669	4508	7621	1534	12812	897	2780	24210	1152	581
	2005	97344	28110	2024	7970	8089	6158	777	1546	1313	233	69234	14339	1240	672	4426	7663	1553	12113	126	2869	22535	1129	571
	2006	95872	28205	2017	7806	8153	6467	634	1601	1309	218	67667	14367	1288	702	4319	8003	1628	11917	468	3066	20232	1094	583
	2007	94533	28310	2016	7767	8206	6600	563	1631	1319	209	66223	14407	1398	768	2691	8748	1786	12290	636	3416	18417	1058	606
	2008	92903	28425	2019	7787	8261	6643	516	1656	1340	203	64478	13677	1456	804	3620	9131	1883	12151	251	3629	16197	1054	623
	2009	94653	28612	2029	7879	8322	6646	495	1673	1370	199	66041	14001	1495	827	2449	9406	1948	11882	3400	3804	15153	1029	646
	2010	92768	28640	2025	7963	8316	6592	484	1669	1394	197	64128	13732	1471	814	2400	9260	1926	11159	2774	3809	15091	1031	663
	2011	90369	28416	2000	7963	8226	6515	456	1656	1407	192	61953	11851	1485	821	1961	9374	1950	10777	1512	3911	16574	1045	692
	2012	87750	28138	1974	7942	8120	6422	434	1636	1422	188	59612	10534	1409	781	2518	8867	1863	9772	542	3768	17804	1048	707
	2013	86254	27871	1948	7936	8013	6321	417	1614	1438	184	58383	9837	1354	751	1949	8543	1803	8984	794	3672	18919	1089	689
	2014	83962	27617	1923	7922	7914	6223	399	1595	1461	180	56345	9135	1310	726	1739	8284	1754	8150	465	3602	19378	1135	668
	2015	82710	27439	1903	7914	7838	6163	378	1582	1484	176	55271	8891	1278	709	1691	8107	1723	7622	461	3555	19464	1113	656
Mauritania	2000	11404	4346	289	1790	995	683	159	190	195	44	7058	1877	164	86	321	1087	205	1570	635	465	416	109	124
	2001	12387	4421	292	1812	996	739	151	191	196	44	7966	1945	171	90	333	1145	216	1582	1332	482	432	120	119
	2002	12139	4520	295	1830	1008	803	144	196	199	44	7619	2013	178	94	345	1207	228	1592	777	500	446	129	111
	2003	12433	4619	299	1862	1024	844	139	202	204	44	7814	2071	184	97	355	1252	238	1592	805	515	455	137	114
	2004	12938	4712	303	1902	1045	863	136	209	210	44	8226	2110	189	100	359	1283	247	1573	1117	527	457	143	120
	2005	11930	4773	306	1924	1059	879	131	214	216	44	7157	2133	192	102	360	1308	255	1539	9	536	455	147	122
	2006	12087	4834	309	1931	1074	912	121	221	223	43	7253	2148	195	103	361	1325	262	1498	89	545	451	149	126
	2007	12118	4838	309	1896	1076	957	103	230	228	41	7280	2152	197	104	361	1337	268	1448	142	551	443	149	128
	2008	12087	4846	308	1864	1078	997	88	240	233	38	7241	2140	197	105	359	1338	272	1389	177	554	431	149	130
	2009	12158	4847	306	1842	1076	1021	76	250	239	36	7311	1877	210	112	287	1435	293	1422	347	596	447	149	136
	2010	12669	4820	302	1839	1061	1021	70	250	242	35	7849	1865	210	112	286	1433	295	1363	970	599	431	149	138
	2011	12117	4786	297	1851	1037	1013	67	242	244	35	7331	1815	212	114	272	1460	301	1325	513	611	424	148	136
	2012	11551	4774	293	1868	1017	1014	65	235	247	35	6777	1766	211	113	260	1453	303	1262	102	612	413	146	135
	2013	11455	4733	287	1870	995	1006	62	229	250	34	6722	1741	210	112	255	1449	304	1204	151	613	405	146	133
	2014	11271	4715	284	1872	982	1003	59	227	255	34	6556	1373	227	122	171	1582	332	1236	162	667	435	121	129
	2015	11050	4695	281	1873	971	994	56	225	262	33	6355	1335	222	119	165	1550	327	1157	157	658	423	117	125
Mauritius	2000	322	207	1	124	32	14	0	7	30	0	115	12	6	0	5	27	39	5	0	20	0	0	0
	2001	294	186	2	101	33	11	0	13	26	0	108	16	9	0	6	29	29	6	0	14	0	0	0
	2002	288	181	2	100	22	13	0	6	38	0	107	12	10	1	2	32	25	4	0	21	0	0	0
	2003	297	188	3	89	37	6	0	9	43	0	109	19	4	0	3	37	23	5	0	18	0	0	0
	2004	311	196	3	104	21	12	0	17	39	0	115	21	12	0	4	16	29	12	0	22	0	0	0
	2005	317	201	7	89	17	18	0	17	53	0	116	14	4	1	4	31	35	3	0	22	0	1	0
	2006	313	197	4	92	24	26	0	14	37	0	116	15	6	3	9	35	26	6	0	15	0	0	0
	2007	296	183	0	98	22	14	0	17	32	0	113	20	3	3	1	30	28	6	0	23	0	0	0
	2008	269	161	2	64	35	15	0	15	29	0	108	18	7	4	4	28	31	4	0	10	0	2	0
	2009	241	142	8	61	12	15	0	7	38	0	99	14	5	1	4	33	26	4	0	13	0	0	0
	2010	221	129	8	56	18	4	0	15	28	0	92	13	5	0	4	18	32	2	0	17	0	2	0
	2011	208	124	7	53	10	13	0	9	32	0	84	15	5	0	2	20	26	5	0	12	0	0	0
	2012	201	122	6	50	9	13	0	13	31	0	79	15	1	0	0	31	19	4	0	8	0	0	0
	2013	198	122	7	48	12	13	0	16	25	0	76	18	1	0	1	28	18	6	0	3	0	0	0
	2014	195	122	6	50	11	13	0	13	30	0	73	15	2	0	1	24	19	5	0	7	0	0	0
	2015	192	119	6	49	10	13	0	12	29	0	73	15	2	0	1	24	19	5	0	7	0	0	0
Mozambique	2000	135268	35101	2457	10718	10308	6550	1045	1941	1679	403	100167	14841	1227	808	3968	8447	1752	14217	4852	2954	36692	9595	814
	2001	132029	34027	2379	10595	9944	6341	902	1895	1608	363	98002	14673	1216	808	3877	8373	1761	13336	4738	3009	34566	10807	839
	2002	129106	33065	2307	10546	9597	6082	804	1842	1558	330	96041	14702	1221	820	3836	8414	1795	12693	4762	3102	31915	11914	866
	2003	127630	32423	2254	10485	9352	5963	711	1816	1539	304	95207	14834	1235	838	3823	8547	1843	12179	6045	3216	28883	12887	876
	2004	125047	31884	2208	10236	9182	6009	605	1825	1540	278	93163	15135	1263	867	3849	8759	1915	11829	6182	3367	25398	13686	914
	2005	124465	31333	2159	9822	9030	6177	485	1857	1551	251	93132	15464	1294	897	3882	8999	1994	11518	8288	3526	22038	14288	944
	2006	113658	30934	2119	9481	8906	6336	395	1896	1573	228	82724	15641	1317	918	3877	9155	2062	11111	114	3652	19310	14604	962
	2007	109133	30544	2077	9246	8754	6408	338	1896	1617	207	78589	15624	1319	924	3821	9202	2087	10520	410	3763	17041	12905	973
	2008	105251	30326	2051	9174	8636	6405	309	1884	1670	197	74925	15429	1312	924	3725	9141	2111	9880	477	3817	15406	11731	974
	2009	101191	29991	2016	9103	8476	6345	285	1858	1721	187	71200	13126	1374	974	2561	9680	2232	9842	151	4097	15467	10725	972
	2010	99963	29771	1990	9071	8353	6296	266	1841	1772	181	70192	12834	1346	960	2471	9504	2207	9205	1514	4095	14680	10399	976
	2011	95916	29491	1957	9027	8201	6241	249	1809	1833	174	66425	12474	1319	945	2354	9345	2176	8590	852	4103	13876	9414	977
	2012	90358	29304	1929	8974	8075	6223	235	1766	1936	166	61054	12174	1295	927	2273	9143	2153	7960	209	4134	12899	6896	992

U5D[1]: total number of under 5 death; NEO[2]: total number of neonatal death; PNE[3]: pneumonia; PRE[4]: preterm; INT[5]: intrapartum related events; SEP[6]: sepsis/meningitis; TET[7]: tetanus; OTH[8]: other disorders; CON[9]: congenital; DIA[10]: diarrea; POS[11]: total number of post neonatal death; MEN[12]: meningitis; MEA[13]: measles; INJ[14]: injury; MAL[15]: malaria; PER[16]: pertussis

		0-28 days										1-59 months												
	Year	U5D[1]	NEO[2]	PNE[3]	PRE[4]	INT[5]	SEP[6]	TET[7]	OTH[8]	CON[9]	DIA[10]	POS[11]	PNE[3]	PRE[4]	INT[5]	MEN[12]	OTH[8]	CON[9]	DIA[10]	MEA[13]	INJ[14]	MAL[15]	AIDS	PER[16]
	2013	86794	29132	1902	8928	7957	6202	222	1730	2030	161	57662	10838	1336	957	1803	9525	2240	7783	317	4352	12353	5224	934
	2014	83765	29037	1882	8908	7870	6180	212	1706	2122	157	54728	9969	1354	970	1513	9721	2289	7501	272	4487	11575	4191	887
	2015	82387	28799	1855	8858	7759	6116	201	1687	2169	153	53588	9914	1356	971	1502	9759	2311	7190	268	4532	10813	4103	869
Namibia	2000	4352	1117	73	388	314	148	7	84	97	6	3235	586	58	27	77	374	79	409	4	173	101	1287	61
	2001	4385	1082	70	379	302	143	6	83	94	6	3303	584	58	27	77	375	79	398	3	172	112	1357	62
	2002	4430	1050	68	374	288	140	6	79	90	5	3380	598	59	27	78	387	82	396	51	176	66	1396	63
	2003	4390	1024	65	371	274	141	5	75	87	5	3366	598	60	27	77	389	83	385	39	177	62	1405	64
	2004	4291	1006	63	370	264	141	5	72	86	5	3285	580	59	27	74	381	82	362	4	173	111	1372	61
	2005	4188	997	62	369	257	142	5	70	87	5	3191	599	61	28	75	397	86	361	8	181	58	1276	62
	2006	4026	1001	61	371	255	144	4	69	91	5	3025	596	61	27	74	398	86	345	15	183	61	1118	60
	2007	3808	1020	62	378	257	148	4	67	98	5	2788	607	62	28	75	408	89	335	22	191	15	896	61
	2008	3633	1038	62	385	258	151	4	67	105	5	2595	609	63	28	75	410	91	321	30	196	8	703	62
	2009	3528	1062	63	395	262	154	4	66	113	5	2466	614	63	29	75	416	92	311	62	201	4	536	63
	2010	3419	1095	64	408	267	159	4	66	121	5	2324	531	71	32	53	471	103	332	43	228	2	395	64
	2011	3386	1118	65	418	269	163	4	66	127	5	2268	540	72	33	53	478	106	324	51	234	2	307	66
	2012	3402	1148	66	431	273	168	4	66	134	5	2254	549	74	34	53	494	110	320	59	242	4	248	65
	2013	3312	1170	67	441	274	172	4	65	140	6	2142	550	76	34	52	508	113	314	1	250	5	175	64
	2014	3329	1183	67	448	275	174	4	65	144	6	2146	555	76	35	53	513	115	305	14	254	8	156	63
	2015	3318	1188	67	452	273	174	4	65	147	5	2130	551	76	35	52	512	115	293	14	254	11	155	63
Niger	2000	125279	24846	1891	6775	7654	4087	1220	1228	1770	222	100433	23982	1846	1013	8727	11641	1950	25655	6289	3989	13723	857	762
	2001	137274	24837	1901	6935	7649	4186	1088	1248	1610	220	112437	24541	1889	1041	8946	11980	2004	24920	17211	4203	13996	928	777
	2002	128199	24738	1903	7117	7554	4276	953	1261	1454	220	103461	24577	1894	1047	8979	12051	2020	23621	9102	4349	14053	981	788
	2003	131131	24612	1897	7320	7393	4366	827	1269	1321	220	106519	24278	1875	1039	8877	11946	2011	22031	14461	4453	13724	1019	806
	2004	130203	24478	1884	7488	7199	4491	708	1275	1217	216	105725	23793	1842	1022	8710	11744	1986	20352	16718	4532	13175	1032	820
	2005	110550	24416	1869	7590	7030	4690	593	1287	1150	207	86134	23225	1802	1004	8505	11496	1957	18718	343	4597	12620	1031	837
	2006	107627	24359	1845	7595	6870	4966	479	1302	1109	193	83268	22662	1765	986	8302	11264	1934	17217	549	4659	12073	1014	844
	2007	104321	24322	1815	7524	6730	5284	380	1319	1092	177	79999	22645	1771	985	5426	11325	1946	16221	715	4834	12307	977	846
	2008	100967	24297	1784	7438	6607	5574	308	1329	1095	162	76670	20894	1644	909	7654	10456	1816	14117	900	4629	11892	921	837
	2009	97054	24353	1766	7409	6566	5724	267	1343	1125	153	72701	17723	1721	950	5664	10997	1912	13896	195	4997	12959	846	841
	2010	94444	24403	1750	7414	6534	5803	239	1349	1169	146	70041	17591	1714	946	3721	10960	1916	13040	407	5128	13072	682	864
	2011	92059	24450	1751	7449	6580	5699	225	1369	1234	142	67609	17082	1699	934	3559	10887	1905	12210	221	5221	12416	600	876
	2012	90514	24590	1759	7516	6643	5617	217	1392	1307	140	65924	17051	1686	924	3570	10781	1900	11463	467	5298	11301	579	905
	2013	88842	24814	1792	7607	6821	5377	218	1455	1406	139	64028	16868	1653	906	4553	10619	1874	10681	34	5302	10122	532	884
	2014	88347	25023	1809	7688	6921	5263	214	1503	1487	138	63324	16508	1701	932	4377	11004	1940	10188	136	5544	9706	416	873
	2015	87967	25495	1845	7837	7091	5207	212	1579	1587	138	62472	16447	1702	933	4353	11072	1954	9734	134	5633	9239	410	861
Nigeria	2000	1011815	243837	18900	67565	80626	29692	17256	13323	13672	2803	767978	126690	10760	6220	21417	65138	14085	128456	143877	23919	196205	24299	6911
	2001	986192	244307	19026	67736	81262	30366	15931	13708	13503	2774	741885	129043	10999	6362	21581	66672	14491	125516	113478	24811	195654	26147	7132
	2002	926955	244828	19117	68244	81652	31044	14690	13999	13352	2729	682127	130616	11165	6458	21472	67747	14780	121780	53631	25600	193842	27639	7397
	2003	1023794	245233	19157	68987	81735	31801	13566	14099	13221	2667	778561	129320	11109	6424	31697	67285	14824	115544	152391	25844	187665	28925	7534
	2004	914675	244723	19088	69356	81476	32603	12480	14052	13104	2565	669952	130216	11243	6499	30993	68111	15125	111430	48109	26554	183995	30017	7660
	2005	975525	244299	19010	69509	81291	33506	11492	13981	13071	2441	731226	130347	11324	6544	30294	68485	15388	106736	116845	27151	179500	30840	7771
	2006	858948	242743	18810	69335	80591	34327	10543	13809	13027	2300	616205	133587	11662	6746	19873	70761	15990	104671	9641	28425	176106	30879	7865
	2007	842753	242060	18632	69574	79804	35554	9654	13624	13039	2179	600693	134447	11794	6831	19705	71734	16323	100798	6004	29224	165215	30669	7949
	2008	837850	241224	18450	69790	78713	36876	8811	13442	13056	2085	596626	133570	11772	6832	29633	71874	16453	95872	14364	29639	148660	30181	7777
	2009	811902	241063	18378	70388	78044	37400	8198	13418	13199	2038	570839	135259	11991	6975	29701	73504	16961	92944	2282	30640	133006	29989	7587
	2010	808242	241117	18312	71463	77570	36999	7840	13478	13428	2026	567125	139056	12382	7228	19727	75903	17705	91506	11417	32145	121958	30099	8000
	2011	809090	241207	18219	73146	76836	36270	7667	13390	13646	2034	567883	139528	12495	7321	19590	76246	18103	88010	23218	32875	111907	30099	8491
	2012	781891	240810	18009	74632	75359	36682	7269	13072	13765	2022	541081	134191	12424	7284	28224	75385	18193	83410	6584	33104	104535	28879	8869
	2013	800912	241561	17868	76063	74232	37754	6862	12816	13965	2000	559351	124623	12975	7608	20737	79372	19198	83064	35648	34973	106118	26367	8669
	2014	762534	240955	17669	76434	73141	38506	6412	12650	14189	1953	521579	118221	13030	7640	19008	79960	19481	79578	9583	35511	104774	26316	8476
	2015	750111	240106	17516	76501	72535	38508	6067	12573	14500	1905	510005	116050	12874	7549	18669	79105	19449	75087	9412	35423	102368	25732	8288
Rwanda	2000	61046	14939	1131	3733	4986	2726	340	1026	837	161	46107	11603	1031	578	4327	6313	1413	11051	666	2212	3495	2963	454
	2001	59804	14902	1130	3843	5011	2622	323	1018	811	144	44902	11910	1059	604	2879	6596	1476	10651	454	2378	3471	2955	470
	2002	54834	13847	1046	3632	4660	2435	268	940	751	114	40987	9079	1049	608	2552	6695	1494	9811	683	2481	3249	2884	402
	2003	47646	12374	926	3278	4137	2220	207	830	690	85	35272	7941	949	560	1427	6183	1381	8227	417	2367	2709	2787	325
	2004	40605	11004	813	2919	3641	2031	155	731	652	63	29601	6910	812	488	1269	5364	1212	6510	24	2139	2084	2515	274
	2005	35653	10045	729	2669	3281	1890	121	661	646	48	25608	5947	718	437	1055	4796	1098	5322	80	1990	1668	2254	242
	2006	32077	9613	684	2552	3090	1840	99	630	679	40	22464	5205	649	390	896	4308	1007	4408	134	1863	1374	1985	244
	2007	29283	9456	658	2507	2988	1827	85	618	738	35	19827	4687	591	351	803	3860	933	3690	45	1754	1172	1660	281
	2008	27154	9433	640	2504	2918	1842	75	611	813	31	17721	4245	544	321	717	3481	877	3136	75	1665	1035	1292	333
	2009	25323	9346	618	2493	2828	1837	67	597	878	29	15977	3865	502	295	645	3144	827	2693	87	1579	930	1028	382
	2010	23374	9065	586	2436	2683	1785	59	568	921	27	14309	3002	493	290	400	3051	829	2468	45	1584	896	838	413
	2011	21098	8473	535	2294	2455	1656	50	521	938	24	12625	2569	456	270	308	2797	786	2137	73	1505	821	481	422
	2012	18809	7808	484	2128	2219	1507	42	472	935	21	11001	2163	421	251	223	2560	742	1715	90	1420	750	252	414
	2013	16870	7149	436	1964	1995	1362	36	426	912	18	9721	1919	382	227	192	2331	688	1357	94	1307	673	185	365
	2014	15250	6653	399	1845	1825	1251	31	391	894	16	8597	1708	344	205	170	2103	634	1157	34	1190	600	128	325
	2015	14207	6299	373	1765	1702	1170	28	366	881	15	7908	1566	319	190	155	1951	601	1019	31	1110	549	117	299
Sao Tome and Principe	2000	435	129	9	47	36	19	1	7	9	1	306	63	7	4	11	44	12	49	1	17	90	8	1
	2001	437	129	9	46	35	20	1	7	9	1	308	63	7	4	11	44	12	46	8	18	88	8	1
	2002	432	128	9	45	35	20	1	7	10	1	304	59	6	3	11	42	11	42	13	17	93	8	1
	2003	425	127	9	44	35	21	1	7	10	1	298	56	6	3	10	40	11	38	16	16	92	9	1
	2004	413	126	8	42	35	21	1	7	11	1	287	52	6	3	9	38	10	34	17	16	93	9	1
	2005	399	123	8	40	34	21	1	7	11	0	276	51	5	3	9	37	10	32	16	16	86	9	1
	2006	385	120	8	38	33	21	1	7	12	0	265	52	5	3	9	38	10	31	18	16	76	7	1
	2007	371	117	8	37	32	21	1	6	12	0	254	48	5	3	8	36	10	28	19	15	75	7	1
	2008	346	114	7	36	31	21	1	6	12	0	232	48	5	3	8	36	10	27	0	15	74	5	1
	2009	329	111	7	35	30	21	1	6	12	0	218	45	5	3	8	34	9	24	3	15	66	5	1
	2010	325	109	7	34	29	20	0	6	12	0	216	37	5	3	5	37	10	25	8	16	67	3	1
	2011	322	108	7	34	28	20	0	6	12	0	214	37	5	3	5	37	10	24	12	16	61	3	1
	2012	320	109	7	34	28	20	0	6	13	0	211	37	5	3	5	37	10	23	14	16	57	3	1
	2013	308	109	7	34	28	20	0	6	13	0	199	29	6	3	2	42	11	25	5	18	56	1	1
	2014	303	109	7	34	28	20	0	6	14	0	194	29	6	3	3	42	11	24	4	19	52	1	1
	2015	297	109	7	34	28	20	0	6	14	0	188	28	6	3	2	41	10	22	4	18	50	1	1
Senegal	2000	52347	14833	1016	4999	4119	2778	341	755	705	120	37514	8726	826	419	2485	5130	1147	7698	4112	1981	4341	200	448
	2001	55995	14788	1016	4891	4144	2812	303	776	730	115	41207	8841	840	426	1637	5207	1173	7426	8738	2059	4163	233	462
	2002	61851	14700	1012	4758	4150	2848	259	800	764	109	47151	8602	821	417	1587	5065	1157	6848	16144	2067	3709	261	472
	2003	47007	14580	998	4631	4095	2933	215	809	797	102	32427	8295	795	404	1526	4898	1127	6244	3165	2062	3163	287	462
	2004	41809	14391	972	4501	3981	3033	177	802	830	95	27418	7949	766	389	1458	4715	1095	5648	29	2048	2570	308	443
	2005	39882	14109	940	4350	3845	3085	147	789	865	88	25773	7327	747	378	1313	4570	1074	5175	219	2060	2138	320	452
	2006	37918	13733	904	4166	3700	3083	122	776	902	81	24185	6047	769	389	946	4728	1110	5020	277	2189	1945	321	445
	2007	35699	13360	870	3999	3571	3033	103	764	944	75	22339	5634	732	369	870	4484	1061	4505	92	2145	1701	309	437
	2008	33951	12935	834	3826	3430	2959	88	746	982	70	21016	5377	687	347	843	4172	1001	3999	248	2074	1527	286	453
	2009	32803	12642	808	3714	3328	2884	78	732	1032	67	20161	5084	646	327	801	3897	945	3567	771	2009	1398	248	467
	2010	31292	12403	786	3613	3242	2821	69	722	1088	63	18889	4783	615	312	748	3690	901	3220	679	1966	1296	207	472
	2011	29869	12267	771	3539	3184	2782	62	717	1151	61	17602	4513	588	297	701	3498	863	2920	410	1927	1202	201	481
	2012	29081	12214	759	3488	3146	2770	56	719	1217	59	16867	4351	566	287	678	3343	833	2677	407	1898	1123	209	495
	2013	28523	12176	751	3450	3113	2759	52	719	1276	57	16347	4235	554	281	658	3281	818	2501	392	1894	1073	180	479
	2014	27779	12192	746	3439	3095	2755	49	720	1332	56	15587	3369	586	297	456	3518	868	2531	173	2035	1115	177	463
	2015	27059	12055	732	3395	3037	2712	46	710	1368	55	15004	3255	567	288	440	3414	843	2349	166	2000	1067	171	445
Seychelles	2000	20	13	0	5	2	1	0	1	3	0	7	1	0	0	0	2	2	0	0	2	0	0	0
	2001	21	13	0	5	2	1	0	1	3	0	8	1	0	0	0	2	2	0	0	2	0	0	0
	2002	22	14	0	5	2	2	0	1	4	0	8	1	0	0	0	2	2	0	0	2	0	0	0
	2003	23	15	0	6	2	2	0	2	4	0	8	1	0	0	0	2	2	0	0	2	0	0	0
	2004	24	16	0	6	2	2	0	2	4	0	8	1	0	0	0	3	2	0	0	2	0	0	0
	2005	25	16	0	6	2	2	0	2	4	0	9	1	0	0	0	3	2	0	0	2	0	0	0
	2006	25	16	0	6	2	2	0	3	4	0	9	1	0	0	0	3	2	0	0	2	0	0	0
	2007	25	16	0	5	2	2	0	3	3	0	9	1	0	0	0	3	2	0	0	2	0	0	0

U5D[1]: total number of under 5 death; NEO[2]: total number of neonatal death; PNE[3]: pneumonia; PRE[4]: preterm; INT[5]: intrapartum related events; SEP[6]: sepsis/meningitis; TET[7]: tetanus; OTH[8]: other disorders; CON[9]: congenital; DIA[10]: diarrea; POS[11]: total number of post neonatal death; MEN[12]: meningitis; MEA[13]: measles; INJ[14]: injury; MAL[15]: malaria; PER[16]: pertussis

			0-28 days										1-59 months											
	Year	U5D[1]	NEO[2]	PNE[3]	PRE[4]	INT[5]	SEP[6]	TET[7]	OTH[8]	CON[9]	DIA[10]	POS[11]	PNE[3]	PRE[4]	INT[5]	MEN[12]	OTH[8]	CON[9]	DIA[10]	MEA[13]	INJ[14]	MAL[15]	AIDS	PER[16]
	2008	25	16	0	5	2	3	0	3	3	0	9	1	0	0	0	3	2	0	0	2	0	0	0
	2009	25	16	0	5	2	3	0	3	3	0	9	1	0	0	0	3	2	0	0	2	0	0	0
	2010	24	16	0	5	2	3	0	3	3	0	8	1	0	0	0	3	2	0	0	2	0	0	0
	2011	25	16	0	5	2	3	0	3	3	0	9	1	0	0	0	3	2	0	0	2	0	0	0
	2012	24	16	0	5	2	3	0	3	3	0	8	1	0	0	0	3	2	0	0	2	0	0	0
	2013	24	15	0	5	2	2	0	3	3	0	9	1	0	0	0	3	2	0	0	2	0	0	0
	2014	24	15	0	5	2	2	0	3	3	0	9	1	0	0	0	3	2	0	0	2	0	0	0
	2015	23	14	0	4	1	2	0	3	3	0	9	1	0	0	0	3	2	0	0	2	0	0	0
Sierra Leone	2000	46041	8700	609	2812	2358	1381	423	432	540	145	37341	5310	507	200	1198	2905	580	6108	7273	861	11962	206	230
	2001	42650	8807	609	2813	2359	1538	361	442	541	144	33843	5412	519	205	1218	2998	597	5968	3402	893	12138	257	236
	2002	42983	9037	622	2833	2415	1713	305	469	538	142	33946	5567	536	212	1242	3143	624	5882	2988	935	12284	311	221
	2003	43749	9349	638	2899	2486	1899	268	497	521	141	34400	5763	559	221	1277	3319	657	5827	3043	988	12175	364	209
	2004	41175	9573	649	2949	2550	2019	242	519	505	139	31602	5981	583	231	1312	3483	693	5784	41	1046	11817	412	221
	2005	41267	9669	655	2939	2614	2075	217	542	491	135	31598	6110	598	238	1322	3603	717	5643	308	1093	11295	448	224
	2006	41194	9742	659	2885	2697	2129	189	570	484	129	31452	6194	610	244	1320	3703	741	5455	509	1134	10848	467	226
	2007	39909	9686	653	2765	2744	2181	158	593	473	119	30223	5368	625	252	956	3836	769	5306	125	1191	11092	475	228
	2008	38754	9502	636	2649	2736	2192	138	590	456	106	29252	4956	602	245	832	3726	751	4834	409	1177	11098	407	216
	2009	37028	9266	615	2578	2690	2154	127	567	440	95	27762	4580	569	233	744	3549	718	4317	432	1144	10890	375	210
	2010	45639	8998	593	2551	2619	2067	125	533	426	84	36641	4278	536	222	687	3366	683	3837	11024	1112	10349	341	207
	2011	41661	8692	571	2508	2528	1972	118	506	416	74	32969	3382	545	229	407	3465	702	3685	9035	1170	9870	273	206
	2012	33495	8385	547	2450	2429	1893	107	485	409	66	25110	3161	550	233	335	3533	718	3512	2883	1218	8567	195	205
	2013	28904	8076	523	2377	2325	1827	94	467	405	58	20828	3104	549	232	322	3558	722	3304	176	1248	7257	165	190
	2014	27733	7792	501	2317	2225	1762	84	449	403	52	19941	3271	549	232	385	3586	728	2888	582	1275	6147	121	178
	2015	26466	7615	486	2290	2156	1716	77	437	406	48	18851	3235	549	232	379	3615	735	2740	552	1300	5230	114	169
Somalia	2000	70614	15909	1353	4445	5091	1957	1284	747	849	183	54705	11149	911	530	2732	5116	1125	10887	18497	2194	787	282	495
	2001	71071	16306	1388	4526	5229	1993	1344	767	872	188	54765	11352	926	541	4259	5206	1146	10783	16746	2234	765	303	504
	2002	64627	16512	1409	4548	5334	1953	1410	777	890	190	48115	11829	962	566	4441	5451	1191	10930	8831	2328	771	319	497
	2003	64467	16610	1420	4549	5403	1902	1460	784	901	191	47857	12715	1033	610	3121	5898	1281	11429	7643	2502	803	330	493
	2004	68979	16692	1431	4520	5493	1840	1504	795	916	193	52287	13074	1060	628	3206	6051	1317	11431	11098	2573	993	340	516
	2005	68478	16886	1454	4506	5622	1813	1545	814	938	195	51592	13448	1089	648	3291	6236	1354	11435	9187	2647	1396	346	513
	2006	61068	17297	1495	4535	5830	1811	1607	848	971	199	43771	13898	1124	673	3396	6422	1403	11477	205	2742	1540	348	543
	2007	67755	17719	1541	4565	6046	1839	1638	882	1004	203	50036	14595	1181	709	3561	6772	1479	11663	5447	2898	843	348	540
	2008	68485	18139	1589	4582	6273	1903	1627	924	1037	204	50346	15063	1220	733	3673	6949	1533	11601	5176	3025	435	348	589
	2009	64098	18399	1627	4544	6455	1998	1547	962	1063	203	45699	15308	1245	747	3733	7070	1572	11336	380	3118	258	347	587
	2010	64464	18551	1654	4513	6574	2085	1456	986	1082	200	45913	15374	1253	751	3749	7092	1586	10942	836	3179	215	343	594
	2011	78179	18436	1654	4432	6589	2105	1379	995	1087	194	59743	15248	1245	747	3714	7010	1583	10407	15397	3208	234	342	607
	2012	70871	18254	1646	4356	6562	2072	1348	991	1090	189	52617	14904	1219	732	3622	6824	1557	9759	9410	3190	450	336	616
	2013	74463	17960	1628	4251	6505	2017	1302	986	1091	182	56503	13544	1262	758	3004	7085	1618	9673	14473	3350	816	326	595
	2014	61499	17761	1618	4176	6474	1981	1253	983	1100	176	43738	13232	1237	742	2928	6923	1591	9060	3116	3335	667	329	578
	2015	60537	17692	1619	4137	6482	1965	1212	985	1119	172	42845	13054	1223	734	2883	6832	1579	8581	3054	3347	669	322	567
South Africa	2000	87751	18736	1577	6803	4139	2481	0	2627	1109	0	69015	11273	1577	837	1108	10895	3066	10230	542	4123	170	24980	215
	2001	89021	17857	1503	6483	3944	2364	0	2504	1057	0	71164	11324	1585	832	1114	10920	3084	10013	360	4079	23	27604	223
	2002	88375	16833	1417	6112	3718	2229	0	2361	997	0	71542	10881	1532	797	1067	10486	3000	9373	600	3889	28	29657	231
	2003	85679	15646	1317	5681	3456	2072	0	2194	926	0	70033	10214	1448	745	996	9827	2852	8535	569	3640	43	30924	240
	2004	82361	14778	1244	5365	3264	1957	0	2072	875	0	67583	9683	1361	693	951	9182	2685	7713	696	3423	25	30917	254
	2005	79768	14176	1193	5147	3131	1877	0	1988	839	0	65592	9238	1344	678	876	9004	2669	7289	300	3375	18	30547	254
	2006	78669	14055	1148	5192	3154	1659	0	2129	773	0	64614	9137	1347	677	854	8985	2701	6997	382	3397	25	29852	259
	2007	79501	14325	1282	5257	3046	2078	0	1802	860	0	65176	9441	1394	699	884	9256	2822	6937	426	3523	10	29518	266
	2008	79271	14527	1184	5124	3279	1953	1	2078	908	0	64744	9895	1472	736	920	9771	2994	7008	146	3763	12	27759	268
	2009	76196	14593	1085	4931	3490	1821	1	2320	945	0	61603	10111	1528	762	940	10130	3122	6700	735	3997	12	23299	268
	2010	68233	14544	1171	5181	3235	2160	1	1812	984	0	53689	9350	1571	779	826	10385	3221	5924	3394	4326	24	13620	269
	2011	61178	14037	927	4887	3085	2119	0	1824	1195	0	47141	8547	1568	774	699	10343	3229	5561	1421	4407	15	10316	260
	2012	56310	13224	801	4477	2801	1955	0	1929	1260	0	43086	7727	1520	749	598	9995	3145	5120	818	4313	20	8821	260
	2013	48612	12377	676	4507	2588	1712	0	1703	1191	0	36235	7486	1366	673	623	8981	2842	4457	647	3976	30	4934	219
	2014	44141	11627	702	4074	2484	1694	0	1600	1073	0	32514	6781	1277	629	543	8388	2672	3949	541	3756	32	3748	197
	2015	41930	11141	673	3903	2380	1624	0	1534	1028	0	30789	6432	1216	599	513	7970	2558	3627	513	3587	38	3549	186
South Sudan	2000	51467	16229	1319	3952	5064	1605	2515	742	867	165	35238	8375	690	370	2243	3562	800	7987	1646	1605	6644	753	562
	2001	50887	16188	1334	3983	5116	1641	2343	751	855	166	34699	8506	700	380	1478	3641	819	7687	2284	1682	6112	854	556
	2002	49303	15976	1333	3968	5110	1651	2152	752	846	164	33327	8256	678	372	1422	3526	800	7067	2417	1685	5595	951	556
	2003	47423	15772	1331	3953	5094	1667	1970	751	845	162	31651	7942	652	362	1355	3413	777	6447	2344	1672	5121	1043	523
	2004	44366	15639	1331	3940	5090	1677	1834	751	856	159	28727	7648	628	353	1291	3296	756	5890	886	1659	4688	1132	500
	2005	42818	15609	1341	3963	5109	1718	1686	756	878	158	27209	7215	593	336	1844	3108	721	5276	494	1611	4317	1217	478
	2006	41629	15625	1352	3998	5128	1770	1553	761	907	156	26004	7035	579	331	1777	3036	712	4890	260	1615	4003	1298	468
	2007	40989	15794	1374	4066	5192	1836	1451	772	948	156	25195	6907	571	327	1728	3001	708	4572	211	1626	3727	1372	445
	2008	40640	16005	1397	4146	5269	1896	1362	786	994	155	24635	7040	584	336	1150	3083	733	4446	186	1696	3506	1436	438
	2009	40556	16240	1419	4228	5355	1944	1296	800	1042	156	24316	7023	584	338	1154	3078	739	4243	165	1726	3329	1491	446
	2010	40699	16456	1441	4300	5440	1976	1240	812	1092	156	24243	7022	586	340	1174	3101	748	4064	322	1757	3192	1505	433
	2011	40873	16651	1459	4353	5539	1969	1202	825	1149	155	24222	6998	585	341	1199	3099	752	3881	540	1784	3105	1505	433
	2012	41092	16837	1477	4393	5654	1925	1181	841	1212	155	24255	6703	562	329	1796	2951	729	3565	878	1738	3045	1518	442
	2013	41160	16999	1490	4419	5759	1872	1174	856	1274	155	24161	6779	571	334	1434	3005	746	3460	1153	1786	2965	1493	435
	2014	39932	17036	1492	4426	5803	1835	1135	864	1328	153	22896	6649	562	329	1427	2959	741	3256	380	1781	2899	1488	425
	2015	39487	17034	1489	4431	5814	1817	1094	868	1371	150	22453	6550	556	326	1415	2927	738	3084	371	1779	2830	1459	417
Sudan	2000	114861	38359	2833	13965	10276	5821	941	2030	2109	385	76502	21404	1763	947	5705	12135	2044	16833	6192	5474	2560	155	1288
	2001	116602	38532	2874	13887	10370	5865	938	2033	2174	391	78070	22102	1818	988	3824	12313	2128	16726	8592	5722	2379	181	1297
	2002	116541	38855	2929	13771	10535	5976	916	2071	2262	395	77686	22139	1818	1000	3801	12030	2144	16115	9094	5807	2180	207	1352
	2003	115692	39097	2984	13503	10677	6205	866	2114	2354	393	76595	22171	1821	1012	3775	11824	2169	15519	8816	5892	2035	233	1330
	2004	109406	39453	3045	13278	10849	6439	822	2170	2458	392	69953	22035	1809	1017	3718	11519	2178	14835	3334	5931	1993	257	1326
	2005	106827	39597	3083	12997	10907	6714	773	2194	2539	389	67230	21177	1738	988	5412	10819	2115	13722	1857	5770	2047	278	1308
	2006	104368	39554	3096	12699	10866	6980	730	2194	2604	385	64814	21114	1737	994	5343	10571	2136	13165	979	5824	1333	297	1320
	2007	102379	39600	3120	12427	10851	7249	700	2199	2673	382	62779	20763	1713	986	5212	10213	2128	12472	794	5790	1117	316	1275
	2008	100134	39438	3127	12119	10790	7423	668	2200	2735	377	60696	18127	1917	1109	2622	11323	2408	13386	700	6525	977	336	1269
	2009	97832	39292	3135	11826	10754	7550	637	2212	2808	371	58540	17877	1859	1081	2613	10720	2357	12478	620	6387	920	339	1289
	2010	96459	39241	3153	11596	10724	7691	611	2213	2885	368	57218	17328	1826	1068	2516	10351	2336	11778	1211	6327	893	343	1243
	2011	95706	39085	3160	11364	10670	7779	584	2212	2952	363	56621	16578	1862	1093	2305	10407	2400	11014	2030	6505	834	358	1233
	2012	95658	39014	3176	11197	10642	7838	562	2211	3027	361	56644	16260	1824	1074	3421	9982	2371	9496	3304	6412	892	368	1239
	2013	95222	38882	3190	11022	10616	7831	541	2210	3114	358	56340	15070	1903	1120	2382	10307	2491	9410	4339	6731	1003	375	1209
	2014	90971	38788	3206	10871	10605	7813	519	2214	3206	355	52183	12429	2061	1213	1664	11096	2719	9664	1431	7334	1000	385	1188
	2015	89488	38567	3209	10695	10553	7761	495	2209	3295	350	50921	12190	2034	1197	1637	10788	2702	9154	1395	7285	1003	376	1160
Swaziland	2000	4366	706	50	225	223	99	5	60	41	3	3660	647	78	36	88	420	149	536	0	151	1	1523	31
	2001	4489	686	48	222	214	96	5	60	39	3	3803	665	81	37	91	438	157	541	1	153	2	1607	31
	2002	4539	661	46	216	205	92	4	59	37	3	3878	677	83	38	92	451	162	537	1	155	1	1649	31
	2003	4558	636	43	209	196	88	4	57	36	3	3922	685	85	38	93	459	168	529	0	156	0	1675	32
	2004	4535	616	42	204	188	85	3	56	35	3	3919	685	86	39	92	463	173	512	1	157	1	1677	34
	2005	4484	607	41	202	183	85	3	56	34	3	3877	681	86	39	92	465	175	491	1	157	1	1656	34
	2006	4241	598	40	199	179	86	3	53	35	2	3643	665	85	38	89	457	175	455	1	159	0	1486	34
	2007	4095	596	39	199	176	88	3	53	36	2	3499	649	83	37	87	448	172	423	0	158	0	1406	35
	2008	4033	600	39	201	176	89	3	53	37	2	3433	629	81	36	84	436	169	394	0	156	0	1413	35
	2009	3723	605	39	203	175	92	3	51	40	2	3118	505	85	38	54	462	179	388	1	170	0	1200	36
	2010	3182	604	38	202	172	95	3	46	47	2	2578	479	81	36	51	440	169	337	0	173	0	776	35
	2011	2870	596	37	198	167	95	2	43	51	2	2274	453	77	35	48	419	163	301	0	173	0	569	36
	2012	2652	576	36	192	160	92	2	40	53	2	2076	431	75	34	45	407	159	274	1	173	0	444	35
	2013	2474	554	34	185	152	88	2	38	55	2	1920	411	72	33	42	396	154	251	1	171	0	357	32
	2014	2291	535	32	178	145	85	2	35	56	2	1756	332	74	33	26	411	159	244	1	180	0	265	30
	2015	2221	519	31	174	139	81	2	34	56	1	1702	322	72	33	26	402	155	228	1	176	0	257	29
Togo	2000	23853	6706	494	2098	2010	1155	160	364	367	59	17147	2343	212	117	511	1199	295	1942	2389	563	6418	945	212
	2001	23180	6763	495	2103	2027	1181	150	371	378	58	16417	2381	215	119	523	1213	300	1899	1628	579	6333	998	228
	2002	21652	6828	498	2090	2057	1215	140	380	392	55	14824	2399	218	121	530	1236	305	1842	20	592	6302	1034	226

U5D[1]: total number of under 5 death; NEO[2]: total number of neonatal death; PNE[3]: pneumonia; PRE[4]: preterm; INT[5]: intrapartum related events; SEP[6]: sepsis/meningitis; TET[7]: tetanus; OTH[8]: other disorders; CON[9]: congenital; DIA[10]: diarrea; POS[11]: total number of post neonatal death; MEN[12]: meningitis; MEA[13]: measles; INJ[14]: injury; MAL[15]: malaria; PER[16]: pertussis

						0-28 days											1-59 months							
	Year	U5D[1]	NEO[2]	PNE[3]	PRE[4]	INT[5]	SEP[6]	TET[7]	OTH[8]	CON[9]	DIA[10]	POS[11]	PNE[3]	PRE[4]	INT[5]	MEN[12]	OTH[8]	CON[9]	DIA[10]	MEA[13]	INJ[14]	MAL[15]	AIDS	PER[16]
	2003	21891	6914	503	2047	2097	1291	123	393	409	51	14977	2395	218	121	533	1249	308	1769	205	599	6310	1050	220
	2004	21952	6990	502	2002	2122	1381	107	405	425	46	14962	2386	218	121	534	1245	310	1696	231	604	6338	1052	226
	2005	21775	7026	500	1965	2131	1444	95	411	438	43	14749	2363	216	121	530	1245	310	1617	46	606	6418	1057	220
	2006	21801	7032	497	1948	2135	1461	89	412	450	40	14769	2345	215	120	526	1244	310	1544	100	610	6449	1088	218
	2007	21756	6999	492	1930	2132	1446	84	414	463	38	14757	2348	216	121	526	1251	315	1489	150	619	6373	1130	220
	2008	21708	6949	487	1919	2116	1428	80	410	473	36	14759	2296	225	127	492	1311	330	1487	251	650	6287	1080	223
	2009	21407	6929	483	1919	2102	1422	77	407	484	35	14478	2142	242	138	407	1433	358	1543	158	710	6132	987	229
	2010	21242	6897	479	1918	2079	1421	74	401	493	34	14345	2245	259	148	421	1556	386	1584	222	766	5654	881	224
	2011	20861	6865	474	1922	2057	1411	72	394	502	33	13996	2414	282	160	449	1718	420	1655	102	842	5117	613	225
	2012	20684	6824	468	1929	2034	1390	71	387	512	32	13860	2584	302	173	481	1857	454	1709	222	913	4520	409	237
	2013	20514	6778	464	1940	2011	1359	70	380	522	32	13736	2668	314	179	496	1951	474	1706	389	958	4012	357	232
	2014	19849	6733	458	1945	1987	1336	69	373	533	31	13116	2513	335	191	439	2105	507	1660	87	1030	3775	248	226
	2015	19512	6694	453	1949	1964	1318	67	369	544	31	12818	2517	337	192	439	2132	513	1606	86	1047	3484	243	221
Uganda	2000	168104	35492	2615	10933	11085	5984	546	2268	1807	252	132612	19640	1583	1223	4580	10640	2590	16731	11939	4238	45595	12531	1322
	2001	161763	35552	2603	10853	11087	6148	495	2288	1843	235	126211	19283	1560	1205	4475	10496	2568	15613	8303	4277	45094	11984	1353
	2002	162408	35468	2577	10702	11018	6316	434	2313	1890	218	126940	18031	1577	1216	3961	10628	2616	14868	12825	4437	43852	11543	1385
	2003	154500	35378	2547	10567	10911	6476	380	2336	1956	204	119122	17090	1565	1203	5513	10525	2614	13863	9731	4525	39851	11229	1416
	2004	139261	35191	2512	10418	10773	6562	334	2358	2042	192	104070	16851	1635	1252	3406	11055	2750	13594	29	4863	36180	11011	1445
	2005	133428	34977	2477	10272	10642	6576	300	2376	2151	183	98451	16764	1653	1262	3343	11161	2808	12891	88	5050	30978	10984	1469
	2006	127549	34532	2427	10097	10460	6458	272	2376	2269	173	93017	16110	1598	1221	4827	10752	2742	11734	265	5027	26021	11206	1515
	2007	121515	33839	2359	9806	10196	6344	244	2346	2383	160	87676	15260	1566	1195	4419	10553	2710	10819	484	5059	22895	11227	1490
	2008	115541	33055	2281	9497	9914	6193	219	2305	2500	148	82486	14937	1537	1172	2835	10315	2686	10010	566	5094	20655	11138	1540
	2009	110271	32354	2208	9208	9631	6112	197	2249	2612	137	77917	14141	1502	1146	2608	10096	2651	9240	629	5091	18087	11214	1513
	2010	105431	31615	2137	8973	9352	5957	183	2176	2709	129	73816	13944	1497	1142	2549	10059	2662	8739	143	5179	15051	11302	1549
	2011	100125	30936	2066	8756	9055	5853	170	2080	2834	122	69189	13818	1507	1147	2506	10147	2698	8314	286	5333	11989	9883	1561
	2012	93935	30363	2003	8561	8801	5727	161	1992	3002	116	63572	13902	1514	1147	2543	10150	2728	7863	407	5488	9365	6849	1616
	2013	90205	29963	1959	8426	8614	5630	151	1946	3124	113	60242	13674	1499	1136	2827	10148	2726	7406	148	5526	7611	6002	1537
	2014	86880	29841	1936	8373	8518	5566	145	1922	3270	110	57039	11997	1548	1172	2328	10633	2839	7280	106	5789	6941	4950	1456
	2015	85291	29738	1916	8351	8430	5514	139	1907	3371	109	55553	11791	1531	1160	2285	10566	2833	6892	103	5777	6375	4821	1418
United Republic of Tanzania	2000	173227	42852	3276	11890	13896	7692	723	2672	2402	300	130375	27879	2211	1626	6283	15502	3328	23227	3520	6437	21993	16987	1382
	2001	167206	42639	3241	11791	13805	7710	656	2670	2492	274	124567	26933	2133	1598	6047	15028	3270	21267	3314	6421	19980	17199	1378
	2002	161756	42625	3217	11722	13792	7737	598	2685	2624	249	119131	25740	2036	1553	8795	14372	3180	19253	1718	6339	17530	17215	1401
	2003	156356	42632	3189	11651	13752	7796	541	2695	2783	226	113724	25977	2055	1596	5759	14625	3277	18405	1252	6614	15722	17034	1408
	2004	150635	42609	3155	11552	13704	7826	491	2706	2969	206	108026	25329	2003	1586	5552	14292	3266	17002	963	6667	13233	16694	1439
	2005	144612	42290	3095	11375	13543	7797	443	2697	3152	188	102322	24600	1945	1570	5322	13866	3244	15659	804	6687	11069	16040	1516
	2006	138036	41627	3012	11110	13269	7672	399	2666	3328	171	96409	23751	1898	1526	5069	13380	3206	14344	388	6662	9290	15332	1563
	2007	130760	40576	2891	10703	12838	7544	348	2617	3481	154	90184	22424	1809	1452	4724	12538	3101	12852	1242	6489	7776	14107	1670
	2008	123662	39760	2787	10361	12441	7514	305	2566	3645	140	83902	21296	1740	1383	4435	11863	3009	11597	1269	6349	6629	12613	1720
	2009	117550	38966	2680	9993	12022	7576	262	2516	3788	128	78584	17423	1859	1474	2867	12685	3257	11690	1115	6907	6421	11101	1784
	2010	111200	38421	2597	9777	11657	7625	236	2435	3975	119	72779	16647	1826	1454	2654	12447	3251	10841	930	6933	5805	8224	1768
	2011	106181	38373	2551	9663	11480	7736	216	2407	4205	114	67808	16164	1786	1420	2572	12045	3225	10009	968	6891	5254	5665	1810
	2012	103423	38401	2525	9639	11374	7760	203	2394	4395	111	65022	15919	1791	1416	2498	12032	3273	9517	347	6967	4960	4502	1801
	2013	101993	38608	2514	9672	11342	7798	193	2412	4570	108	63385	13062	1975	1562	1551	13480	3661	8807	460	7730	5237	4106	1756
	2014	99367	38684	2494	9692	11259	7783	185	2398	4767	106	60683	12134	1997	1579	1244	13700	3755	8322	439	7888	5122	2822	1681
	2015	98180	38575	2468	9689	11144	7727	177	2388	4878	105	59605	11925	1980	1566	1219	13569	3777	7896	429	7844	4977	2771	1651
Zambia	2000	75031	15455	1129	4393	4885	2883	229	1033	789	115	59576	11268	757	841	2069	6591	1457	10694	3615	2306	11259	8287	433
	2001	71518	15465	1122	4319	4879	2985	196	1068	790	105	56053	10959	737	822	1997	6428	1426	9853	2127	2315	10470	8476	444
	2002	69113	15244	1098	4203	4788	3021	164	1084	793	93	53869	10447	705	788	1888	6129	1377	8830	3279	2301	9051	8620	455
	2003	62498	14841	1060	4054	4635	2996	137	1077	801	81	47657	9910	672	751	1777	5815	1324	7854	715	2283	7374	8717	465
	2004	58084	14510	1027	3939	4504	2964	118	1062	825	71	43574	8078	701	783	1149	6068	1395	7646	6	2468	6159	8649	473
	2005	55218	14216	996	3843	4388	2922	104	1043	856	63	41002	7839	682	767	1114	5902	1374	7006	101	2487	4689	8557	486
	2006	53094	14078	978	3799	4323	2902	97	1025	896	59	39016	7689	672	757	1088	5802	1371	6514	222	2516	3716	8172	497
	2007	51031	14098	972	3798	4306	2914	93	1010	949	56	36933	7608	668	753	1069	5749	1375	6118	218	2560	3131	7176	509
	2008	49564	14168	971	3809	4307	2935	91	1003	999	54	35396	7443	674	759	1003	5791	1404	5853	87	2623	2836	6417	505
	2009	47673	14265	972	3849	4310	2940	90	984	1067	53	33408	7222	674	759	929	5785	1417	5534	154	2678	2712	5042	504
	2010	47838	14218	964	3867	4281	2874	92	957	1130	53	33620	7260	659	739	959	5588	1397	5115	2213	2667	2651	3831	542
	2011	46254	14089	957	3892	4245	2742	95	932	1174	53	32165	7102	648	720	930	5414	1380	4768	1736	2638	2663	3598	569
	2012	43577	13754	931	3834	4142	2587	93	904	1212	51	29823	6869	626	692	900	5161	1344	4365	743	2582	2669	3268	602
	2013	41048	13530	912	3778	4062	2491	90	884	1262	50	27518	6543	602	665	851	4962	1305	3984	7	2523	2660	2846	569
	2014	39737	13276	890	3697	3966	2426	84	865	1300	48	26461	5288	646	713	541	5374	1414	3642	122	2741	2892	2544	545
	2015	38990	13188	880	3669	3920	2398	80	857	1338	47	25802	5162	634	701	529	5275	1404	3421	121	2715	2826	2481	532
Zimbabwe	2000	41487	8249	590	2750	2500	1101	63	665	537	42	33238	4524	529	205	797	3113	837	3567	124	1158	1392	16922	70
	2001	41956	8516	610	2824	2591	1139	65	686	558	43	33440	4688	547	214	836	3230	867	3593	230	1200	1377	16586	73
	2002	42372	8809	631	2905	2690	1183	68	707	581	44	33563	4916	572	225	893	3390	909	3659	333	1262	1395	15931	77
	2003	42343	9138	655	3000	2797	1235	72	723	610	46	33205	5212	605	240	969	3597	964	3754	137	1344	1346	14958	79
	2004	42835	9516	682	3116	2912	1303	76	738	641	48	33319	5540	642	257	1056	3827	1024	3863	249	1435	1349	13995	82
	2005	43489	9991	715	3273	3045	1397	83	749	678	51	33498	5863	677	274	1146	4061	1084	3956	374	1526	1484	12971	82
	2006	43777	10515	750	3447	3182	1513	90	757	722	54	33262	6193	715	291	1236	4303	1151	4031	455	1626	1395	11785	81
	2007	43733	11098	789	3640	3336	1636	98	767	773	58	32635	6227	720	294	1263	4335	1165	3910	128	1650	2166	10701	78
	2008	43902	11679	827	3834	3483	1762	107	776	830	61	32223	5911	850	354	1004	5162	1383	4466	236	1982	1221	9570	83
	2009	44159	12244	864	4047	3622	1861	117	781	887	64	31915	6176	883	371	1060	5368	1445	4464	366	2083	909	8705	86
	2010	43977	12668	890	4238	3707	1923	127	772	944	67	31309	5970	899	383	965	5476	1487	4357	524	2154	1217	7798	80
	2011	43158	12865	900	4373	3730	1913	134	751	996	68	30293	6059	929	396	959	5640	1548	4288	491	2258	874	6772	80
	2012	40389	12992	901	4449	3726	1905	136	720	1085	68	27397	5685	943	405	839	5732	1583	4108	398	2374	706	4544	80
	2013	38979	13052	900	4499	3719	1881	136	708	1142	68	25927	4563	993	427	484	6040	1676	4125	110	2547	1302	3586	74
	2014	38529	12970	890	4489	3678	1853	131	699	1164	66	25559	4756	1016	437	528	6166	1722	3768	158	2627	908	3398	75
	2015	38087	12836	876	4464	3622	1820	126	690	1172	65	25251	4722	1012	435	523	6131	1723	3614	158	2632	870	3357	74
Western Asia	2000	190947	91060	4217	33900	17812	10720	1204	8527	14219	460	99887	24677	3439	1374	3121	24698	12596	14889	3981	9136	751	27	1199
	2001	179966	88101	4115	32209	17318	10706	1093	8329	13891	439	91865	23132	3394	1366	2982	23414	11927	13557	1333	8770	770	28	1191
	2002	172072	85305	4027	30705	16880	10626	971	8128	13545	423	86767	21752	3412	1357	2854	21987	11226	12433	1298	8340	858	30	1220
	2003	165897	82282	3947	29365	16405	10299	930	7796	13126	415	83615	20773	3314	1316	2753	20790	10614	11552	2353	8132	741	32	1243
	2004	160612	79545	3867	28359	15955	9838	900	7443	12770	412	81067	19911	3219	1284	2646	19755	10071	10766	3707	7948	500	34	1226
	2005	153496	77469	3804	27827	15552	9300	884	7125	12562	414	76027	18192	3224	1283	2248	19293	9733	10469	1820	8027	464	34	1241
	2006	148997	76012	3747	27436	15258	8904	846	6903	12509	410	72985	16889	3184	1268	1971	18542	9364	9915	2056	7943	562	36	1256
	2007	143291	74844	3700	27061	15016	8612	805	6729	12521	400	68447	16187	3132	1253	1874	17765	9051	9201	62	7798	846	38	1240
	2008	139891	73813	3649	26499	14811	8515	734	6608	12612	383	66078	15712	3096	1250	1815	17217	8827	8638	91	7713	440	40	1239
	2009	137331	72616	3587	25857	14555	8477	663	6487	12626	364	64715	15301	3091	1253	1773	16732	8640	8122	695	7644	178	40	1247
	2010	133136	71396	3439	25379	14017	8303	594	6241	13079	344	61740	14266	3018	1153	1679	15500	8956	7502	237	7745	390	46	1248
	2011	130058	69866	3377	24311	13572	8189	542	6158	13390	327	60192	13027	2887	1137	1460	15371	8911	7398	651	7721	296	41	1292
	2012	127008	68230	3231	24146	13172	7970	496	6224	12678	313	58778	11809	3017	1165	1272	15332	8220	6998	706	8511	370	42	1335
	2013	122395	66475	3181	24001	12743	7682	462	5555	12548	302	55920	11287	3021	1151	1098	14573	8026	6328	148	8635	339	42	1273
	2014	117869	64379	3078	23332	12291	7387	431	5317	12250	291	53490	10754	2818	1094	1064	14317	7805	6000	198	7923	255	44	1217
	2015	113547	62320	2973	22652	11862	7110	403	5105	11934	280	51227	10448	2735	1068	1027	13871	7555	5687	193	7193	234	42	1175
Bahrain	2000	164	60	0	21	7	2	0	4	26	0	104	3	11	2	1	22	42	1	0	22	0	0	0
	2001	158	54	0	22	5	1	0	2	24	0	104	6	14	2	0	21	41	0	0	19	0	0	0
	2002	156	48	0	21	4	1	0	2	22	0	108	7	18	2	2	19	47	0	0	13	0	1	0
	2003	156	43	0	21	2	1	0	1	17	0	113	7	28	2	1	22	42	0	0	10	0	1	0
	2004	159	39	0	18	4	1	0	2	14	0	120	6	27	4	2	28	41	1	0	10	0	1	0
	2005	164	35	0	19	2	1	0	1	10	0	129	6	32	5	2	33	40	2	0	10	0	0	0
	2006	162	32	0	15	3	2	0	2	10	0	130	6	24	5	4	31	46	2	0	11	0	0	0
	2007	161	29	0	12	3	2	0	2	10	0	132	8	23	7	4	30	47	2	0	11	0	0	0
	2008	158	27	0	8	3	3	0	2	11	0	131	8	17	11	3	35	44	1	0	13	0	0	0
	2009	155	26	0	7	3	3	0	2	11	0	129	10	15	12	1	40	38	1	0	12	0	0	0
	2010	151	25	0	6	2	3	0	1	13	0	126	7	12	8	1	47	39	0	0	12	0	0	0
	2011	152	25	0	7	1	2	0	2	13	0	127	6	18	4	3	45	41	0	0	10	0	1	0
	2012	150	25	0	10	1	1	0	2	11	0	125	3	23	4	2	41	43	1	0	9	0	1	0
	2013	148	26	0	12	0	0	0	3	10	0	122	3	27	4	2	35	41	1	0	7	0	1	0

U5D[1]: total number of under 5 death; NEO[2]: total number of neonatal death; PNE[3]: pneumonia; PRE[4]: preterm; INT[5]: intrapartum related events; SEP[6]: sepsis/meningitis; TET[7]: tetanus; OTH[8]: other disorders; CON[9]: congenital; DIA[10]: diarrea; POS[11]: total number of post neonatal death; MEN[12]: meningitis; MEA[13]: measles; INJ[14]: injury; MAL[15]: malaria; PER[16]: pertussis

			0-28 days										1-59 months											
	Year	U5D[1]	NEO[2]	PNE[3]	PRE[4]	INT[5]	SEP[6]	TET[7]	OTH[8]	CON[9]	DIA[10]	POS[11]	PNE[3]	PRE[4]	INT[5]	MEN[12]	OTH[8]	CON[9]	DIA[10]	MEA[13]	INJ[14]	MAL[15]	AIDS	PER[16]
	2014	142	24	0	11	1	1	0	2	10	0	118	4	23	5	1	35	41	1	0	7	0	0	0
	2015	135	24	0	9	1	1	0	2	11	0	111	3	20	3	2	36	38	1	0	8	0	1	0
Iraq	2000	37398	20579	1269	6559	5087	3309	170	1435	2617	133	16819	5099	476	336	576	3628	898	3294	532	1803	0	0	176
	2001	37323	20608	1257	6446	5066	3437	149	1470	2658	125	16715	5070	481	340	574	3630	932	3168	533	1804	0	0	183
	2002	37266	20495	1239	6340	5007	3494	134	1483	2678	119	16771	5024	483	341	575	3621	956	3037	751	1797	0	0	187
	2003	36428	20350	1225	6341	4943	3429	130	1464	2701	116	16078	4988	484	341	576	3618	972	2920	196	1793	0	0	191
	2004	36555	20226	1218	6458	4888	3246	136	1417	2746	117	16329	4964	487	344	569	3622	998	2811	545	1794	0	0	195
	2005	36148	20306	1224	6712	4880	3022	151	1370	2826	121	15842	4975	494	347	565	3645	1027	2726	49	1808	0	0	206
	2006	36620	20781	1252	7036	4969	2909	164	1366	2961	124	15839	4982	501	352	558	3661	1064	2641	41	1821	0	0	219
	2007	37373	21322	1280	7319	5071	2874	171	1380	3102	126	16051	5057	515	360	559	3736	1112	2594	33	1859	0	0	226
	2008	38272	21935	1313	7513	5203	2940	168	1418	3254	125	16337	5155	530	369	567	3846	1159	2558	32	1906	0	0	214
	2009	39680	22508	1340	7623	5324	3076	159	1469	3395	123	17172	5247	547	380	572	3944	1217	2518	588	1951	0	0	208
	2010	39727	22959	1357	7616	5421	3255	142	1531	3519	117	16768	5296	557	384	571	3998	1249	2457	60	1983	0	0	214
	2011	39839	23210	1362	7620	5457	3348	132	1565	3613	113	16629	5265	560	384	559	3993	1273	2361	38	1984	0	0	211
	2012	39851	23254	1357	7685	5434	3294	130	1554	3689	111	16597	4953	590	405	483	4186	1362	2205	100	2087	0	0	224
	2013	39574	23091	1343	7808	5346	3110	135	1498	3739	111	16483	4659	618	424	412	4368	1447	2091	59	2180	0	0	224
	2014	39102	22866	1324	7863	5242	2964	135	1450	3778	110	16236	4595	607	417	409	4266	1443	2091	49	2139	0	0	220
	2015	38682	22482	1294	7820	5109	2840	131	1404	3778	107	16200	4584	611	419	406	4270	1475	2017	48	2150	0	0	220
Jordan	2000	4103	2481	114	1002	396	170	61	272	466	0	1622	368	86	22	29	310	261	197	0	323	0	0	27
	2001	3967	2417	107	970	381	158	67	271	462	0	1550	321	105	26	33	324	255	162	0	299	0	0	26
	2002	3845	2342	101	937	365	174	43	268	454	0	1503	297	120	30	32	312	256	142	0	290	0	0	25
	2003	3740	2265	96	903	348	151	57	263	447	0	1475	284	119	30	31	307	256	133	0	290	0	0	25
	2004	3674	2228	93	888	337	156	43	263	448	0	1446	272	116	30	29	303	255	126	0	289	0	0	25
	2005	3660	2208	91	880	329	165	26	265	451	0	1452	268	118	30	29	302	261	120	0	296	0	0	27
	2006	3642	2200	90	877	323	171	14	268	457	0	1442	261	121	31	28	299	265	112	0	297	0	0	27
	2007	3662	2201	88	876	319	167	13	273	465	0	1461	260	125	32	28	301	274	108	0	304	0	0	29
	2008	3719	2247	89	895	321	169	13	279	482	0	1472	258	128	33	27	303	280	106	0	307	0	0	30
	2009	3773	2274	89	908	319	172	13	278	495	0	1499	260	133	34	27	307	289	106	0	311	0	0	32
	2010	3788	2288	90	919	312	175	13	273	507	0	1500	257	136	35	26	305	293	104	0	309	0	0	34
	2011	3826	2307	91	935	301	179	13	266	520	0	1519	258	141	37	26	309	301	104	0	310	0	0	34
	2012	3845	2312	93	945	290	182	13	258	531	0	1533	257	145	38	25	311	307	104	0	310	0	0	35
	2013	3829	2294	92	940	281	181	13	251	536	0	1535	254	148	39	25	311	312	103	0	308	0	0	35
	2014	3786	2266	91	928	274	177	13	245	537	0	1520	248	149	40	24	308	312	101	0	304	0	0	35
	2015	3670	2184	88	893	262	169	12	236	524	0	1486	239	149	39	22	301	309	97	0	295	0	0	34
Kuwait	2000	573	298	4	119	14	9	0	11	141	0	275	30	16	8	4	58	118	3	0	38	0	0	0
	2001	567	293	3	108	22	13	0	13	135	0	274	29	23	3	8	42	122	4	0	44	0	0	0
	2002	542	274	4	120	9	4	0	12	126	0	268	28	23	6	1	66	112	3	0	29	0	0	0
	2003	514	254	5	99	9	7	0	7	127	0	260	24	19	7	6	56	111	4	0	33	0	0	0
	2004	502	248	1	93	16	5	0	8	124	0	254	22	20	5	4	61	111	0	0	31	0	0	0
	2005	518	260	2	99	18	8	0	8	126	0	258	21	19	9	6	50	113	5	0	35	0	0	0
	2006	552	279	1	111	19	4	0	9	134	0	273	18	32	8	1	51	134	1	0	27	0	0	0
	2007	611	309	3	143	19	3	0	6	134	0	302	32	43	7	3	52	128	5	0	32	0	0	0
	2008	678	333	3	135	15	11	0	3	165	0	345	23	52	6	0	68	159	3	0	33	0	0	0
	2009	736	348	3	147	6	13	0	9	171	0	388	35	79	7	5	69	151	3	0	38	0	0	0
	2010	763	335	1	123	14	13	0	10	174	0	428	40	81	12	5	79	157	2	0	53	0	0	0
	2011	753	313	4	136	10	6	0	7	150	0	440	66	76	11	11	76	145	4	0	52	0	0	0
	2012	725	282	2	132	9	4	0	10	124	0	443	60	78	6	8	92	150	3	0	46	0	0	0
	2013	677	261	1	131	9	2	0	13	105	0	416	59	63	0	7	102	146	2	0	36	0	0	0
	2014	634	240	2	113	8	3	0	9	106	0	394	56	65	5	8	82	134	3	0	40	0	0	0
	2015	598	222	2	104	7	3	0	8	98	0	376	54	62	5	8	78	128	3	0	39	0	0	0
Lebanon	2000	1226	720	37	286	113	57	0	59	168	0	506	94	15	4	8	116	102	51	9	100	0	1	6
	2001	1149	678	35	266	104	53	0	58	161	0	471	84	15	4	7	117	96	50	0	91	0	1	6
	2002	1096	641	33	248	97	50	0	58	155	0	455	78	16	4	7	114	94	51	0	85	0	1	6
	2003	1046	613	30	235	91	48	0	58	150	0	433	66	22	6	7	113	92	45	2	72	0	1	7
	2004	975	572	27	217	84	45	0	57	142	0	403	59	23	6	7	103	88	40	3	66	0	1	7
	2005	881	513	23	193	74	39	0	54	129	0	368	52	24	7	6	90	83	34	5	59	0	1	7
	2006	772	443	18	166	64	33	0	50	113	0	329	45	25	7	5	78	77	27	6	51	0	1	6
	2007	656	375	14	140	53	27	0	44	97	0	281	37	24	7	4	64	68	21	7	43	0	1	6
	2008	556	315	11	117	44	22	0	39	82	0	241	30	23	7	3	53	60	16	6	36	0	1	6
	2009	504	284	9	106	40	19	0	36	75	0	220	26	23	7	3	48	55	14	6	32	0	1	5
	2010	506	289	8	107	40	19	0	38	77	0	217	25	25	7	3	47	55	13	6	31	0	1	5
	2011	556	318	9	118	44	20	0	42	85	0	238	26	30	8	3	51	61	13	6	33	0	1	6
	2012	644	376	10	139	52	24	0	51	101	0	268	29	35	9	3	60	69	14	5	37	0	1	6
	2013	741	439	11	162	60	28	0	59	119	0	302	31	40	11	3	67	79	16	7	41	0	0	7
	2014	816	482	12	178	66	30	0	66	131	0	334	34	45	12	3	74	88	17	7	44	0	0	7
	2015	835	491	12	181	66	31	0	67	134	0	344	34	47	13	3	76	92	18	7	45	0	0	8
Oman	2000	918	412	5	149	71	19	7	59	104	0	506	97	17	6	8	134	128	20	0	83	0	4	9
	2001	828	370	4	132	63	15	6	54	95	0	458	84	17	6	7	122	119	17	0	75	0	4	9
	2002	761	340	4	121	57	13	6	51	88	0	421	67	25	8	7	112	113	14	0	62	0	4	9
	2003	710	315	4	112	52	12	6	47	82	0	395	61	25	8	7	104	108	12	0	57	0	4	9
	2004	680	306	3	109	50	11	6	46	81	0	374	56	26	8	6	97	103	12	0	53	0	4	9
	2005	667	302	3	108	48	11	5	45	80	0	365	54	27	8	6	93	102	11	0	51	0	4	9
	2006	658	301	2	109	47	12	5	46	80	0	357	52	29	8	5	89	100	11	0	49	0	4	10
	2007	671	308	2	112	48	13	4	47	82	0	363	52	31	8	5	90	101	11	0	50	0	4	10
	2008	696	321	2	117	49	13	5	50	85	0	375	52	33	9	5	94	105	11	0	53	0	4	10
	2009	734	339	2	124	51	14	5	52	90	0	395	51	35	9	5	101	110	11	0	57	0	3	11
	2010	799	370	2	137	56	15	5	56	99	0	429	53	39	10	6	113	119	11	0	64	0	3	12
	2011	823	383	2	142	58	16	5	57	103	0	440	52	40	10	6	119	122	11	0	67	0	2	12
	2012	865	401	2	150	60	17	5	58	108	0	464	53	43	11	6	127	127	11	0	72	0	2	13
	2013	914	419	3	157	63	18	6	60	113	0	495	56	45	11	6	136	135	12	0	77	0	2	14
	2014	932	430	3	161	65	18	6	62	116	0	502	56	46	11	6	138	137	12	0	78	0	2	14
	2015	935	422	3	158	63	18	6	60	114	0	513	58	47	12	6	141	140	12	0	81	0	2	14
Qatar	2000	148	78	0	30	12	4	0	11	21	0	70	7	7	2	1	20	17	1	0	14	0	0	1
	2001	141	73	0	28	11	3	0	10	20	0	68	7	7	2	1	19	16	1	0	14	0	0	1
	2002	134	70	0	27	10	3	0	9	19	0	64	6	7	2	1	18	15	1	0	13	0	0	1
	2003	131	67	0	26	10	3	0	9	19	0	64	6	7	2	1	18	15	1	0	13	0	0	1
	2004	133	67	0	27	10	3	0	9	19	0	66	6	8	2	1	18	16	1	0	14	0	0	1
	2005	141	70	0	28	10	3	0	9	20	0	71	6	8	2	1	19	17	1	0	15	0	0	1
	2006	137	68	0	27	10	3	0	9	19	0	69	6	8	2	1	18	17	1	1	15	0	0	1
	2007	138	69	0	28	10	3	0	9	20	0	69	6	8	2	1	17	17	1	1	15	0	0	1
	2008	145	72	0	29	10	3	0	9	21	0	73	6	9	2	1	19	18	1	1	16	0	0	1
	2009	151	75	0	31	10	3	0	9	22	0	76	6	9	2	1	20	19	1	1	16	0	0	1
	2010	159	79	0	33	10	4	0	9	23	0	80	6	10	2	1	21	20	1	1	16	0	0	1
	2011	178	89	0	37	11	4	0	10	26	0	89	7	11	3	1	23	22	1	1	18	0	0	2
	2012	197	100	1	41	13	5	0	11	30	0	97	7	12	3	1	26	24	1	1	19	0	0	2
	2013	214	106	1	44	13	5	0	12	31	0	108	8	14	4	1	29	27	1	0	21	0	0	2
	2014	224	111	1	46	14	5	0	13	33	0	113	8	15	4	1	30	29	1	0	22	0	0	2
	2015	226	109	1	45	14	5	0	13	33	0	117	8	16	4	1	31	30	1	0	23	0	0	2
Saudi Arabia	2000	13110	7086	97	2684	1282	514	0	944	1564	0	6024	1187	315	79	108	1511	1240	346	18	1117	0	0	103
	2001	12468	6721	97	2516	1201	484	0	919	1504	0	5747	1093	310	77	100	1460	1199	315	6	1088	0	0	99
	2002	11935	6469	97	2399	1146	461	0	905	1461	0	5466	908	435	109	114	1421	1164	261	4	954	0	0	97
	2003	11487	6179	96	2301	1069	442	0	855	1415	0	5308	854	431	108	108	1381	1138	244	5	943	0	0	96
	2004	11112	5991	95	2251	1014	431	0	812	1388	0	5121	797	426	106	101	1333	1106	225	7	924	0	0	93
	2005	10952	5919	96	2244	981	428	0	785	1385	0	5033	761	425	106	97	1320	1092	211	2	925	0	0	93
	2006	10866	5932	95	2269	963	429	0	771	1404	0	4934	722	424	106	93	1298	1077	196	3	921	0	0	94
	2007	10868	5936	95	2291	944	430	0	755	1421	0	4932	697	422	105	90	1301	1085	184	10	940	0	0	97
	2008	10837	5949	93	2316	927	429	0	741	1444	0	4888	661	420	105	87	1311	1087	169	0	948	0	0	100

U5D[1]: total number of under 5 death; NEO[2]: total number of neonatal death; PNE[3]: pneumonia; PRE[4]: preterm; INT[5]: intrapartum related events; SEP[6]: sepsis/meningitis; TET[7]: tetanus; OTH[8]: other disorders; CON[9]: congenital; DIA[10]: diarrea; POS[11]: total number of post neonatal death; MEN[12]: meningitis; MEA[13]: measles; INJ[14]: injury; MAL[15]: malaria; PER[16]: pertussis

			0-28 days									1-59 months												
	Year	U5D[1]	NEO[2]	PNE[3]	PRE[4]	INT[5]	SEP[6]	TET[7]	OTH[8]	CON[9]	DIA[10]	POS[11]	PNE[3]	PRE[4]	INT[5]	MEN[12]	OTH[8]	CON[9]	DIA[10]	MEA[13]	INJ[14]	MAL[15]	AIDS	PER[16]
	2009	10818	5929	91	2330	906	427	0	720	1456	0	4889	632	414	103	85	1329	1093	157	1	971	0	0	104
	2010	10644	5850	87	2321	877	420	0	691	1454	0	4794	588	400	100	81	1325	1079	143	1	972	0	0	105
	2011	10440	5709	83	2284	839	406	0	660	1438	0	4731	554	389	97	77	1331	1069	133	1	977	0	0	103
	2012	10151	5519	77	2226	795	389	0	625	1406	0	4632	520	378	94	73	1322	1051	123	0	970	0	0	101
	2013	9795	5356	72	2171	758	372	0	602	1381	0	4439	484	363	91	68	1275	1014	113	1	934	0	0	97
	2014	9405	5112	66	2078	715	350	0	573	1331	0	4293	457	355	89	63	1237	989	106	1	903	0	0	94
	2015	9002	4866	60	1980	674	328	0	546	1278	0	4136	431	348	87	59	1193	962	99	1	866	0	0	90
Syrian Arab Republic	2000	12162	6520	360	2565	1218	437	68	369	1503	0	5642	1389	170	108	72	1213	1010	1157	0	424	0	1	98
	2001	11543	6246	337	2426	1153	418	67	384	1462	0	5297	1148	224	143	82	1268	977	976	0	379	0	1	98
	2002	10816	5863	308	2246	1068	395	66	392	1388	0	4953	1043	212	138	75	1187	937	891	0	376	0	1	94
	2003	10030	5455	279	2070	974	379	61	391	1300	0	4575	932	198	130	68	1097	886	804	0	368	0	2	91
	2004	9359	5069	252	1904	889	354	63	390	1217	0	4290	844	188	124	63	1030	849	739	0	361	0	2	90
	2005	8947	4854	237	1805	837	343	58	401	1174	0	4093	780	182	120	58	984	826	694	0	356	0	2	91
	2006	8707	4763	228	1756	807	337	54	419	1161	0	3944	732	180	119	55	946	813	656	0	349	0	2	93
	2007	8691	4768	225	1748	795	331	55	443	1170	0	3923	714	184	123	53	937	826	637	1	351	0	2	97
	2008	8638	4774	221	1743	783	330	49	466	1181	0	3864	689	188	125	50	916	833	612	0	346	0	2	103
	2009	8504	4671	214	1700	754	317	49	475	1162	0	3833	670	192	128	48	903	836	606	0	342	0	2	106
	2010	8078	4456	202	1618	709	293	50	468	1116	0	3622	604	177	118	43	857	778	626	0	311	0	2	107
	2011	7832	4239	192	1535	665	271	50	459	1067	0	3593	531	146	100	37	824	694	724	0	425	0	2	111
	2012	7999	3893	175	1406	603	243	46	433	986	0	4106	440	99	72	29	748	574	858	0	1158	0	2	126
	2013	7188	3549	160	1278	544	218	42	404	903	0	3639	354	75	54	23	666	470	895	0	988	0	2	112
	2014	6333	3205	144	1151	487	193	38	374	818	0	3128	304	64	46	19	602	412	878	0	698	0	2	103
	2015	5515	2966	133	1062	448	175	35	353	759	0	2549	294	63	46	18	587	408	874	0	156	0	2	101
Turkey	2000	55723	28392	565	12253	3567	2188	0	3671	6149	0	27331	3924	1116	455	390	11442	7634	539	0	1827	0	4	0
	2001	51562	26547	524	11386	3280	2060	0	3448	5849	0	25015	3467	1050	428	356	10497	7078	456	0	1679	0	4	0
	2002	47335	24687	484	10521	2999	1929	0	3220	5534	0	22648	3029	979	399	320	9520	6489	383	0	1525	0	3	0
	2003	43147	22516	438	9531	2689	1772	0	2949	5137	0	20631	2662	920	375	290	8682	5982	324	0	1393	0	3	0
	2004	39252	20453	395	8598	2401	1620	0	2690	4750	0	18799	2339	867	354	262	7915	5513	274	0	1271	0	3	0
	2005	36060	18703	358	7805	2158	1491	0	2469	4422	0	17357	2082	831	339	240	7307	5145	235	0	1174	0	3	0
	2006	33110	17255	327	7146	1957	1384	0	2286	4155	0	15855	1833	789	322	217	6670	4748	200	0	1072	0	3	0
	2007	30629	16041	302	6591	1787	1294	0	2132	3934	0	14588	1624	756	309	198	6128	4410	172	0	985	2	3	0
	2008	28465	14927	278	6083	1634	1211	0	1991	3730	0	13538	1451	733	299	181	5672	4128	149	0	912	10	3	0
	2009	26598	13900	257	5617	1495	1134	0	1859	3539	0	12698	1310	721	294	168	5308	3907	131	0	853	2	3	0
	2010	24984	13195	174	5495	1144	896	0	1630	3855	0	11789	856	716	218	147	4387	4294	75	0	1090	0	8	0
	2011	23691	12381	181	4702	963	825	0	1605	4106	0	11310	736	607	222	126	4289	4302	144	0	881	0	3	0
	2012	22468	11871	125	4838	922	774	0	1791	3420	0	10597	558	758	265	143	4186	3642	204	0	840	0	2	0
	2013	21428	11307	165	4919	871	745	0	1269	3339	0	10121	770	785	259	115	3377	3499	222	3	1091	0	0	0
	2014	20135	10593	155	4608	816	698	0	1188	3128	0	9542	617	645	223	114	3524	3402	171	1	843	0	1	0
	2015	18937	9980	146	4342	768	658	0	1120	2947	0	8957	579	605	209	107	3308	3194	161	1	791	0	1	0
United Arab Emirates	2000	563	282	2	108	41	12	0	42	77	0	281	29	34	10	5	76	71	5	0	47	0	0	5
	2001	544	273	1	106	39	12	0	39	75	0	271	27	33	9	4	73	68	4	0	47	0	0	5
	2002	523	266	1	104	38	12	0	36	74	0	257	25	31	9	4	69	65	4	0	45	0	0	5
	2003	514	260	1	103	37	12	0	34	73	0	254	24	31	9	4	68	64	4	0	45	0	0	5
	2004	536	271	1	109	38	12	0	34	77	0	265	24	32	9	4	72	67	4	0	48	0	0	5
	2005	583	299	1	121	42	13	0	36	86	0	284	25	34	10	4	77	72	4	0	51	0	0	6
	2006	615	317	1	129	44	14	0	36	92	0	298	26	36	10	4	80	76	5	0	54	0	0	7
	2007	674	341	2	140	47	15	0	38	100	0	333	29	40	12	4	90	85	5	0	60	0	0	8
	2008	731	375	1	154	52	16	0	42	110	0	356	30	44	13	5	94	92	6	0	63	0	0	10
	2009	770	399	1	164	55	17	0	44	118	0	371	31	47	14	5	96	97	6	0	64	0	0	11
	2010	773	396	2	163	54	17	0	43	118	0	377	31	49	15	4	96	100	7	0	64	0	0	12
	2011	797	403	2	165	55	17	0	44	121	0	394	32	52	16	5	99	105	7	0	65	0	0	13
	2012	780	403	2	165	54	17	0	43	121	0	377	30	51	16	4	92	102	7	0	61	0	0	14
	2013	741	382	2	156	51	16	0	41	116	0	359	28	50	16	4	87	99	6	0	57	0	0	13
	2014	702	356	2	144	47	15	0	39	109	0	346	26	49	15	3	84	96	6	0	54	0	0	13
	2015	661	339	2	137	45	14	0	37	104	0	322	24	47	15	3	78	90	5	0	49	0	0	12
Yemen	2000	64859	24152	1764	8125	6004	4000	898	1650	1382	328	40707	12449	1175	344	1918	6169	1077	9277	3422	3337	750	17	773
	2001	59716	23821	1748	7803	5993	4051	804	1661	1447	314	35895	11797	1115	326	1809	5842	1024	8403	795	3232	770	18	765
	2002	57663	23810	1755	7622	6081	4088	722	1693	1546	303	33853	11240	1065	309	1715	5530	977	7647	543	3151	858	20	796
	2003	57993	23965	1772	7622	6180	4042	676	1715	1658	298	34028	10864	1030	299	1653	5324	948	7062	2151	3116	741	22	818
	2004	57675	24075	1781	7688	6223	3954	652	1716	1767	295	33600	10521	999	291	1598	5173	922	6533	3152	3087	500	24	801
	2005	54774	24000	1768	7812	6173	3775	644	1681	1853	293	30774	9161	1031	299	1234	5372	955	6425	1763	3245	464	24	801
	2006	53156	23641	1731	7794	6052	3607	609	1640	1923	285	29515	8206	1017	298	999	5320	946	6062	2006	3275	562	26	798
	2007	49157	23145	1689	7662	5920	3453	562	1600	1986	274	26012	7672	960	282	924	5018	897	5461	10	3149	844	28	767
	2008	46996	22538	1637	7390	5770	3368	499	1570	2046	258	24458	7348	919	272	885	4806	863	5006	52	3081	429	30	765
	2009	44908	21863	1580	7100	5593	3283	438	1535	2092	242	23045	7023	877	261	852	4565	827	4569	100	2996	176	31	770
	2010	42764	21154	1515	6841	5379	3195	383	1489	2125	226	21610	6504	816	243	792	4226	773	4064	169	2842	390	33	757
	2011	41171	20489	1452	6630	5168	3095	342	1439	2149	214	20682	5496	816	246	608	4212	776	3896	605	2899	296	33	800
	2012	39333	19794	1386	6408	4938	3021	302	1386	2151	202	19539	4898	805	242	494	4142	767	3468	600	2903	370	35	814
	2013	37147	19245	1332	6223	4746	2988	267	1343	2156	191	17902	4581	792	238	432	4119	757	2867	78	2895	339	37	769
	2014	35658	18694	1278	6051	4558	2933	240	1297	2154	181	16964	4349	753	226	411	3937	722	2613	141	2791	255	38	729
	2015	34351	18235	1234	5921	4405	2870	219	1259	2155	174	16116	4140	718	216	391	3772	690	2399	135	2692	234	36	693

U5D[1]: total number of under 5 death; NEO[2]: total number of neonatal death; PNE[3]: pneumonia; PRE[4]: preterm; INT[5]: intrapartum related events; SEP[6]: sepsis/meningitis; TET[7]: tetanus; OTH[8]: other disorders; CON[9]: congenital; DIA[10]: diarrea; POS[11]: total number of post neonatal death; MEN[12]: meningitis; MEA[13]: measles; INJ[14]: injury; MAL[15]: malaria; PER[16]: pertussis

Webappendix 7. Global CSMFs in 2000-2015

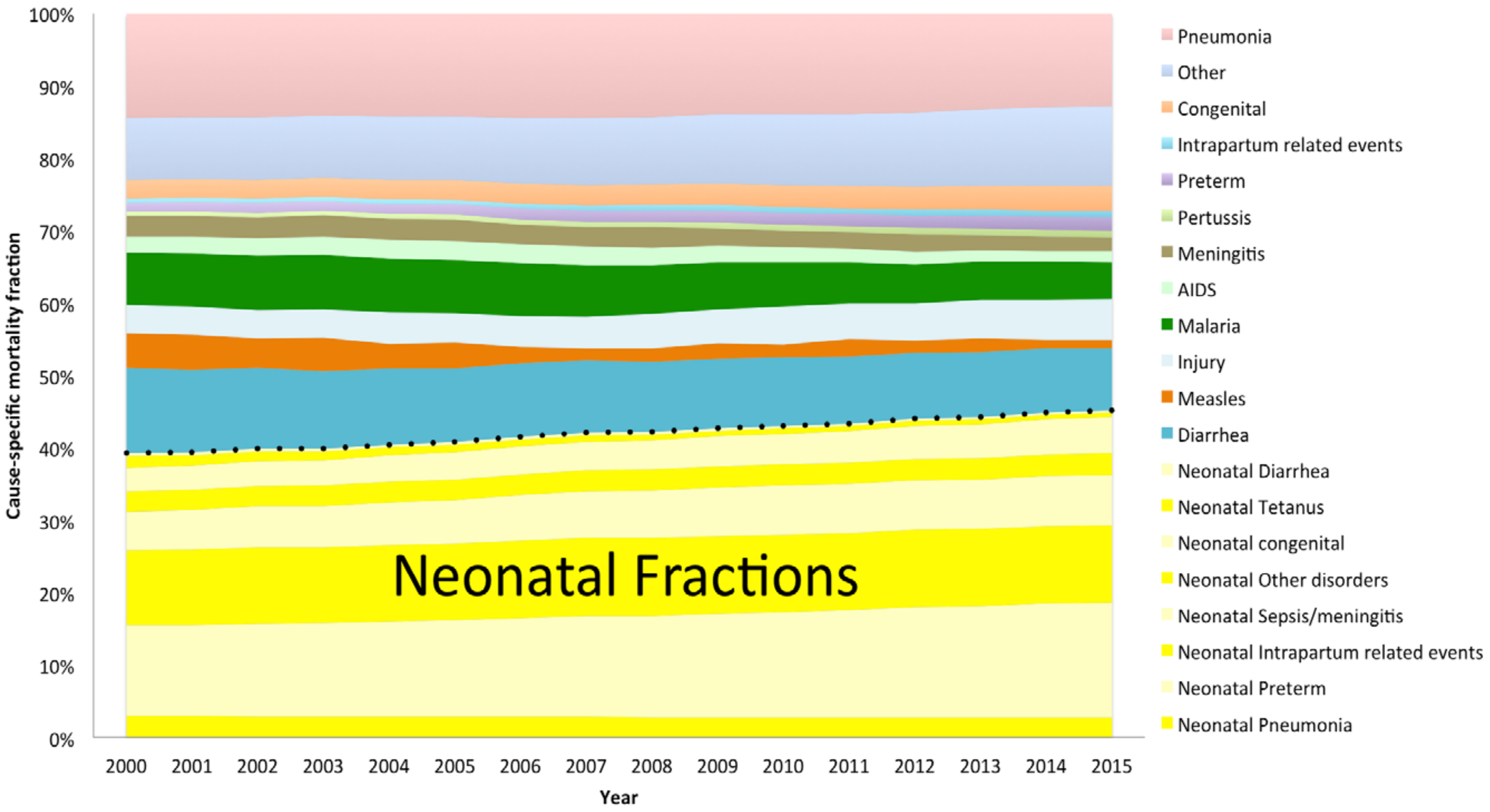

Webappendix 8. Global cause-specific average annual rate of reduction in 2000-2015

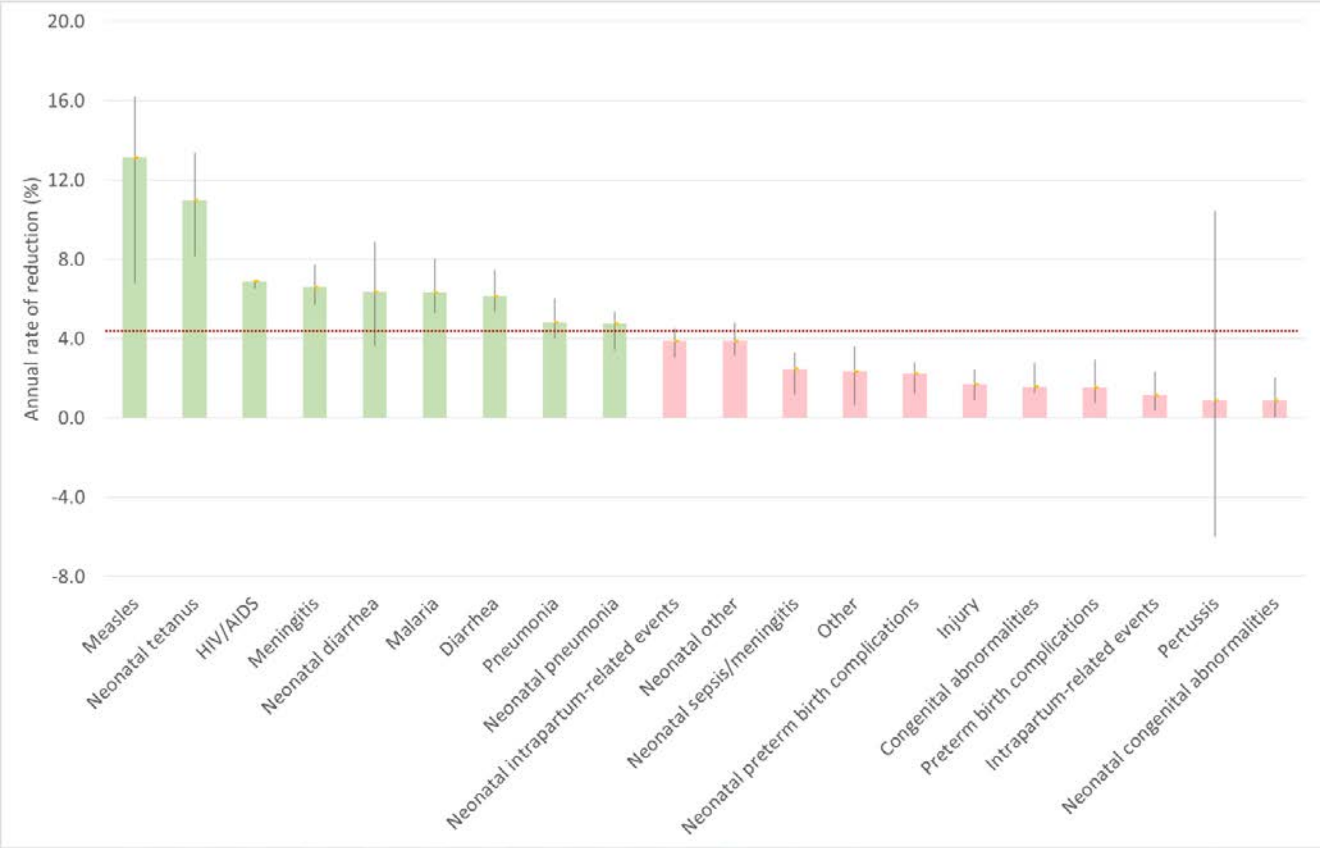

Green bars represent causes that have been declining at an average annual rate of reduction (ARR) of at least 4.4% since 2000; Red bars represent the remaining causes; The dotted line represents an ARR of 4.4%.

Webappendix 9. Trends in cause-specific mortality rates in neonates and children aged 1–59 months by the MDG regions, 2000–2015

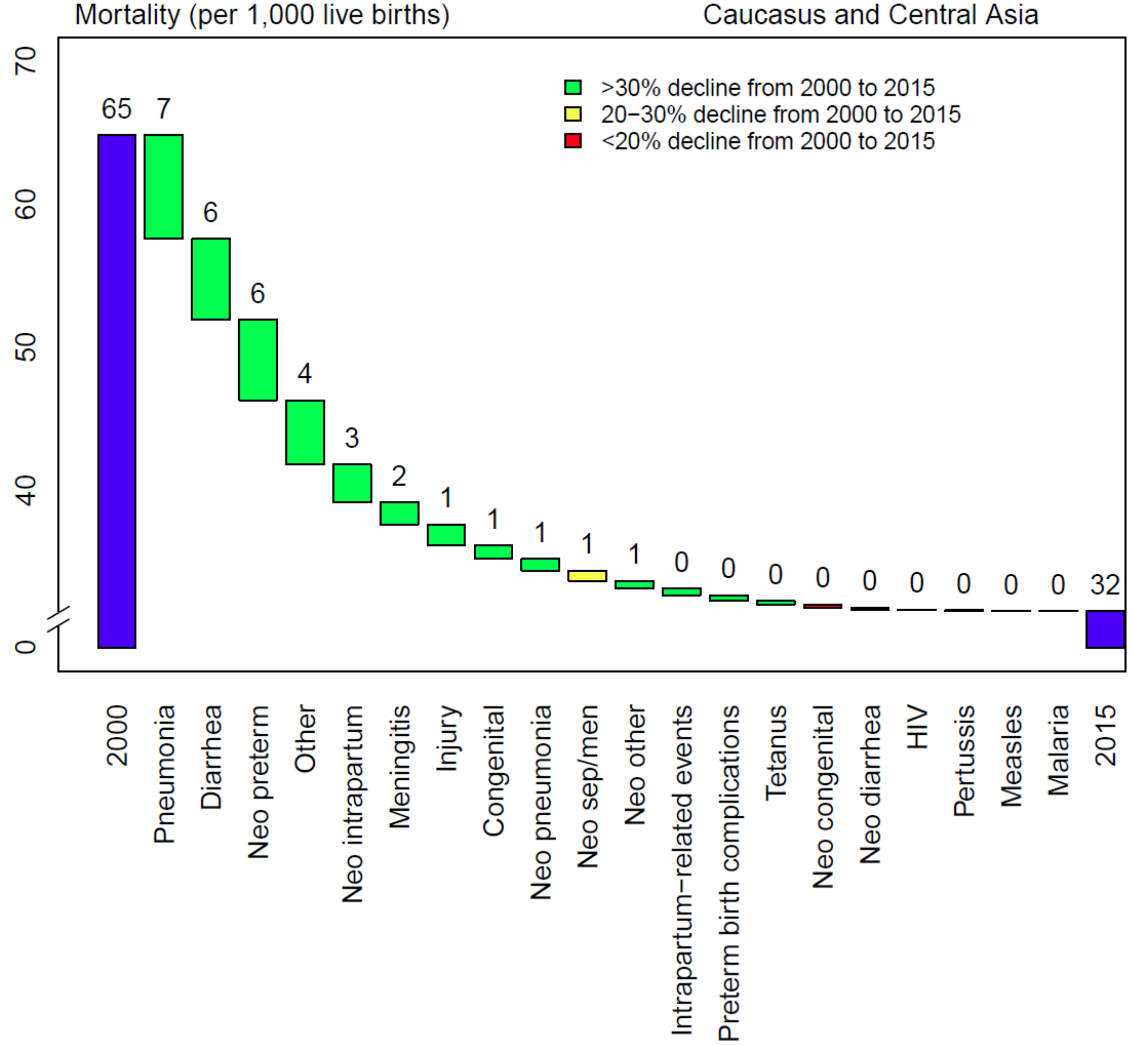

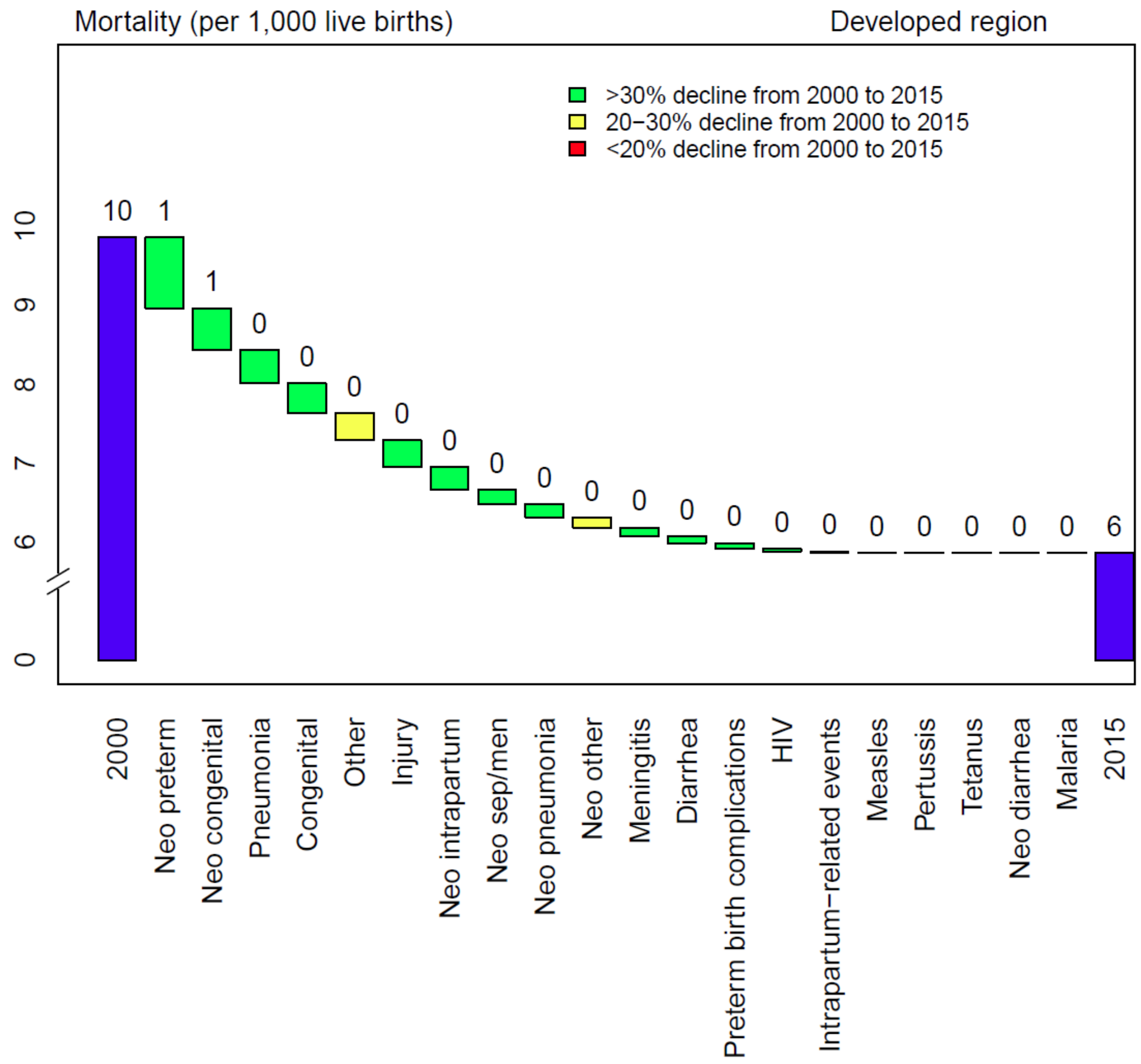

Mortality (per 1,000 live births)
Developed region
>30% decline from 2000 to 2015
20−30% decline from 2000 to 2015
<20% decline from 2000 to 2015
0
6
7
8
9
10
10
1
1
0
0
0
0
0
0
0
0
0
0
0
0
0
0
0
0
0
0
6
2000
Neo preterm
Neo congenital
Pneumonia
Congenital
Other
Injury
Neo intrapartum
Neo sep/men
Neo pneumonia
Neo other
Meningitis
Diarrhea
Preterm birth complications
HIV
Intrapartum−related events
Measles
Pertussis
Tetanus
Neo diarrhea
Malaria
2015

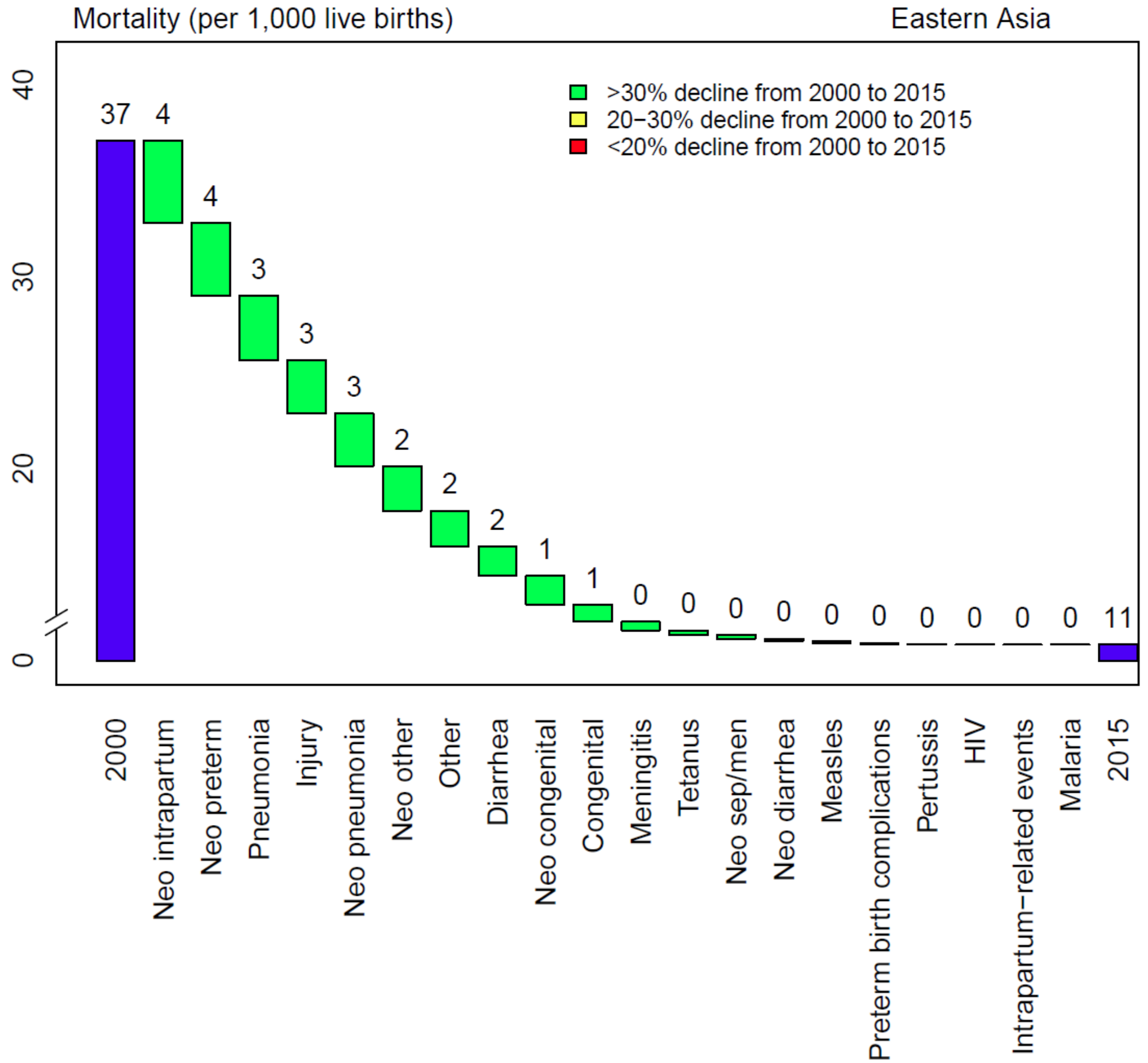

Mortality (per 1,000 live births)
Eastern Asia
>30% decline from 2000 to 2015
20–30% decline from 2000 to 2015
<20% decline from 2000 to 2015
0
20
30
40
37
4
4
3
3
3
2
2
2
1
1
0
0
0
0
0
0
0
0
0
0
11
2000
Neo intrapartum
Neo preterm
Pneumonia
Injury
Neo pneumonia
Neo other
Other
Diarrhea
Neo congenital
Congenital
Meningitis
Tetanus
Neo sep/men
Neo diarrhea
Measles
Preterm birth complications
Pertussis
HIV
Intrapartum-related events
Malaria
2015

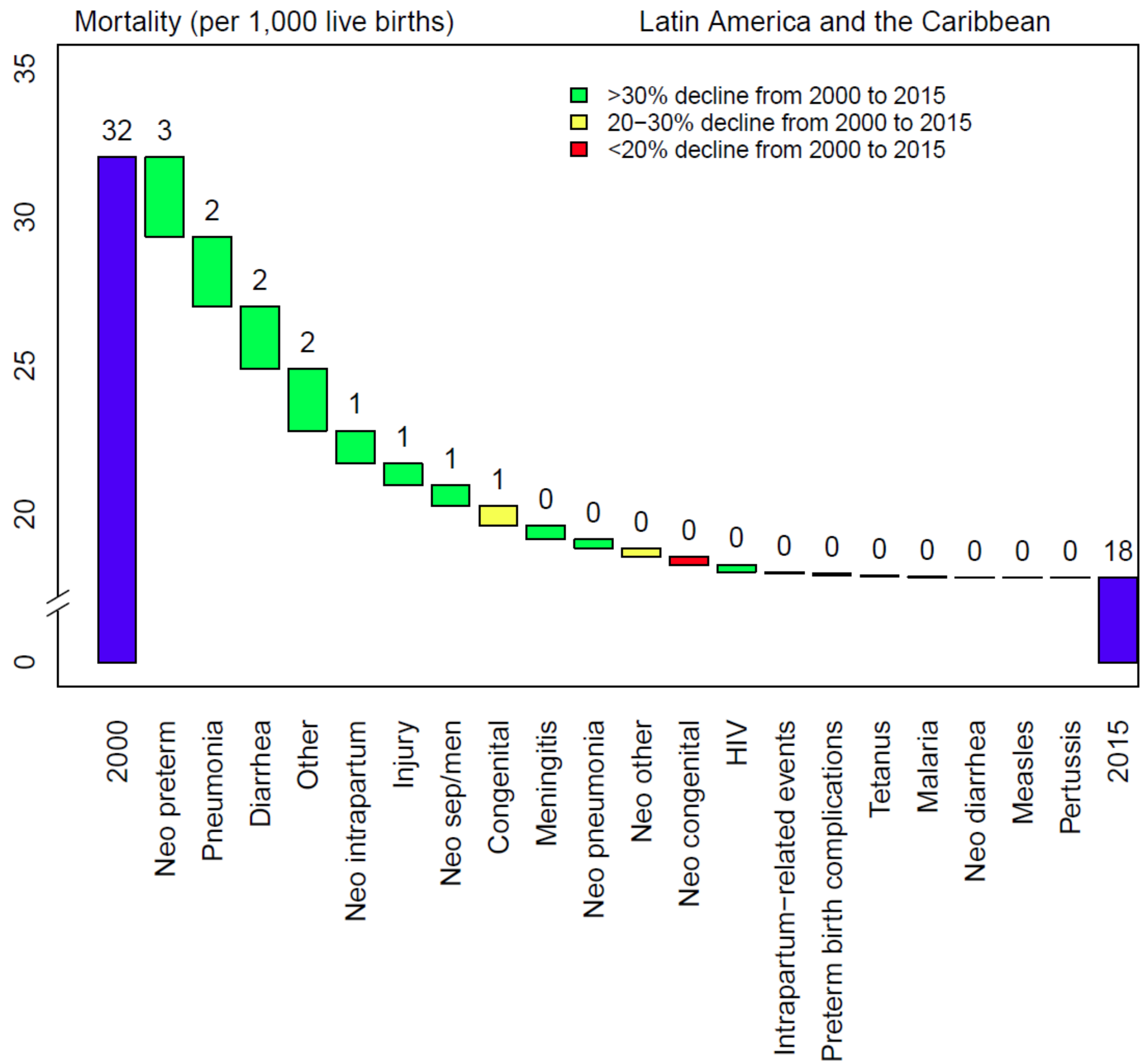
Mortality (per 1,000 live births)
Latin America and the Caribbean
>30% decline from 2000 to 2015
20–30% decline from 2000 to 2015
<20% decline from 2000 to 2015
35
30
25
20
0
32
3
2
2
2
1
1
1
1
0
0
0
0
0
0
0
0
0
0
0
0
18
2000
Neo preterm
Pneumonia
Diarrhea
Other
Neo intrapartum
Injury
Neo sep/men
Congenital
Meningitis
Neo pneumonia
Neo other
Neo congenital
HIV
Intrapartum−related events
Preterm birth complications
Tetanus
Malaria
Neo diarrhea
Measles
Pertussis
2015

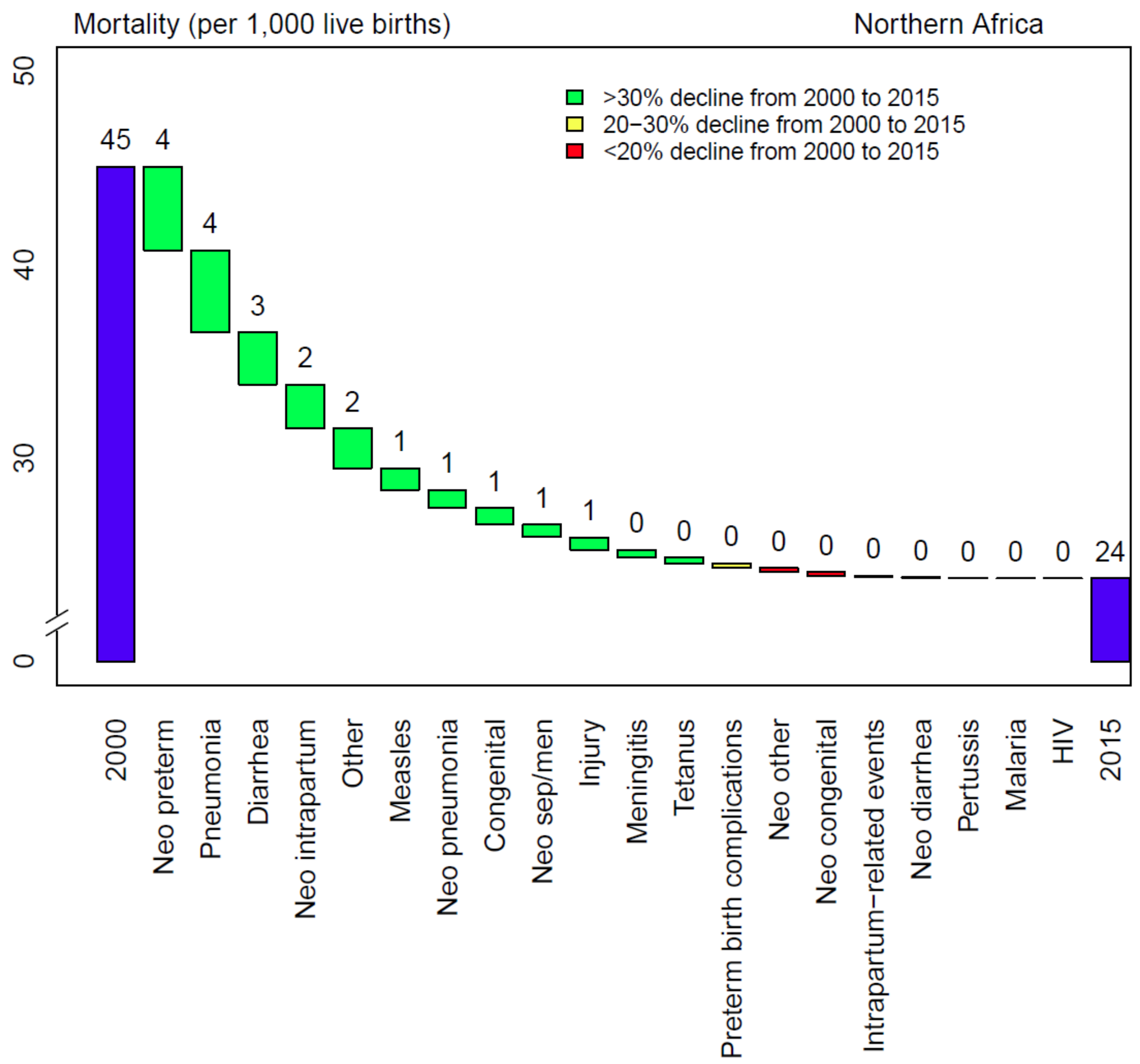
Mortality (per 1,000 live births)
Northern Africa
>30% decline from 2000 to 2015
20–30% decline from 2000 to 2015
<20% decline from 2000 to 2015
50
40
30
0
45
4
4
3
2
2
1
1
1
1
1
0
0
0
0
0
0
0
0
0
0
24
2000
Neo preterm
Pneumonia
Diarrhea
Neo intrapartum
Other
Measles
Neo pneumonia
Congenital
Neo sep/men
Injury
Meningitis
Tetanus
Preterm birth complications
Neo other
Neo congenital
Intrapartum−related events
Neo diarrhea
Pertussis
Malaria
HIV
2015

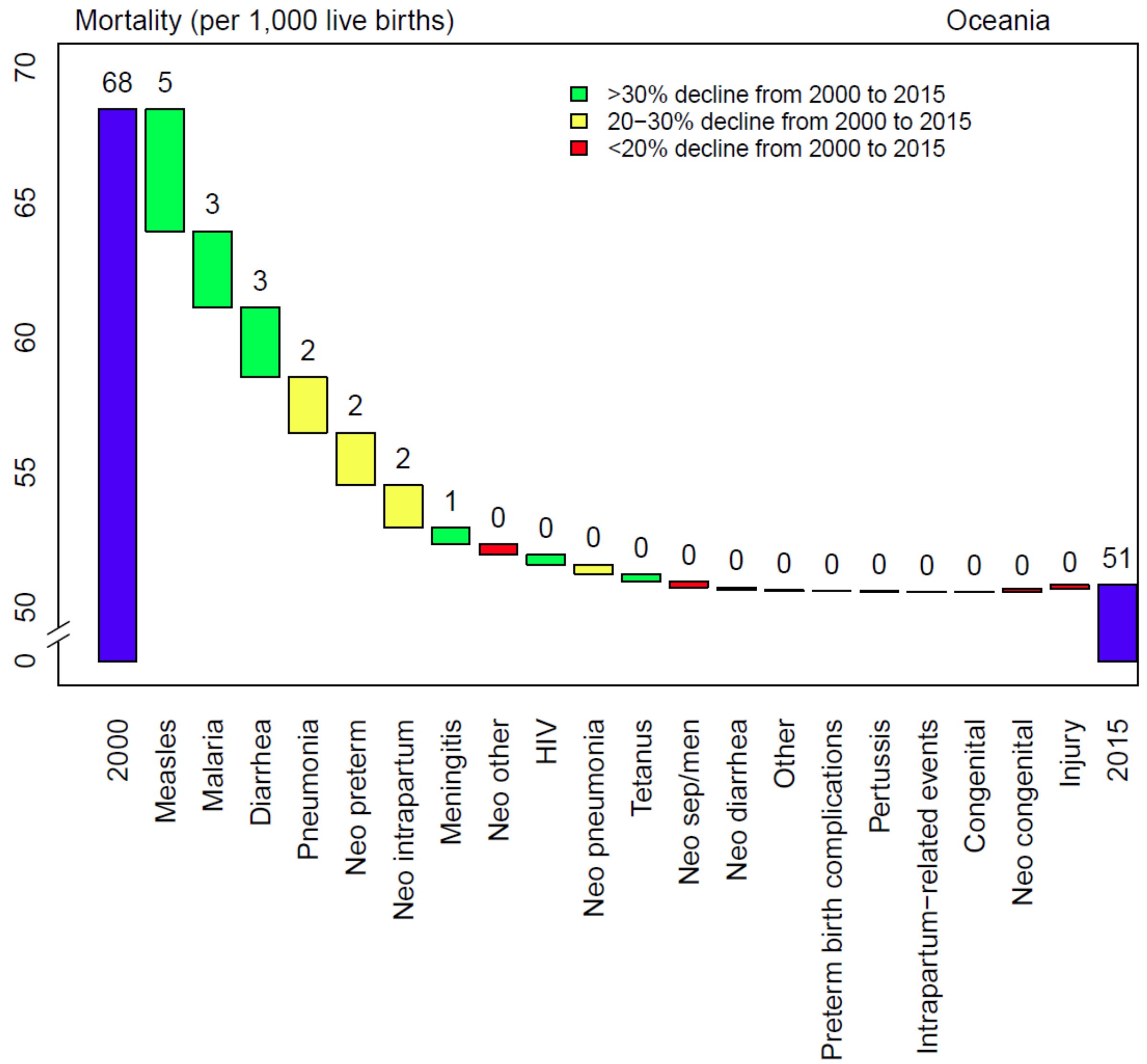
Mortality (per 1,000 live births)
Oceania
>30% decline from 2000 to 2015
20–30% decline from 2000 to 2015
<20% decline from 2000 to 2015
70
65
60
55
50
0
68
5
3
3
2
2
2
1
0
0
0
0
0
0
0
0
0
0
0
0
51
2000
Measles
Malaria
Diarrhea
Pneumonia
Neo preterm
Neo intrapartum
Meningitis
Neo other
HIV
Neo pneumonia
Tetanus
Neo sep/men
Neo diarrhea
Other
Preterm birth complications
Pertussis
Intrapartum−related events
Congenital
Neo congenital
Injury
2015

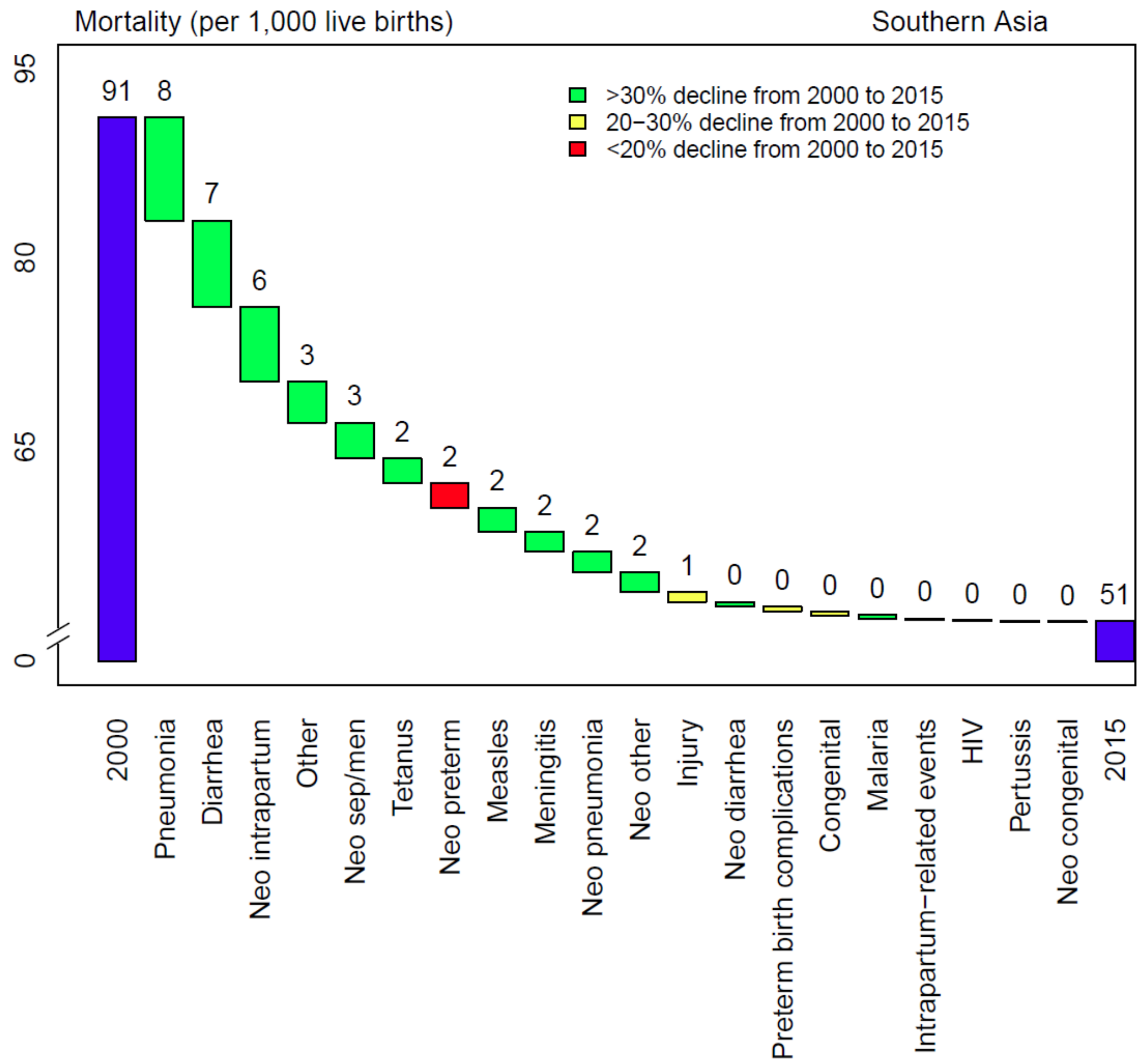

Mortality (per 1,000 live births)
Southern Asia
95
80
65
0
91
8
7
6
3
3
2
2
2
2
2
2
1
0
0
0
0
0
0
0
0
51
>30% decline from 2000 to 2015
20–30% decline from 2000 to 2015
<20% decline from 2000 to 2015
2000
Pneumonia
Diarrhea
Neo intrapartum
Other
Neo sep/men
Tetanus
Neo preterm
Measles
Meningitis
Neo pneumonia
Neo other
Injury
Neo diarrhea
Preterm birth complications
Congenital
Malaria
Intrapartum–related events
HIV
Pertussis
Neo congenital
2015

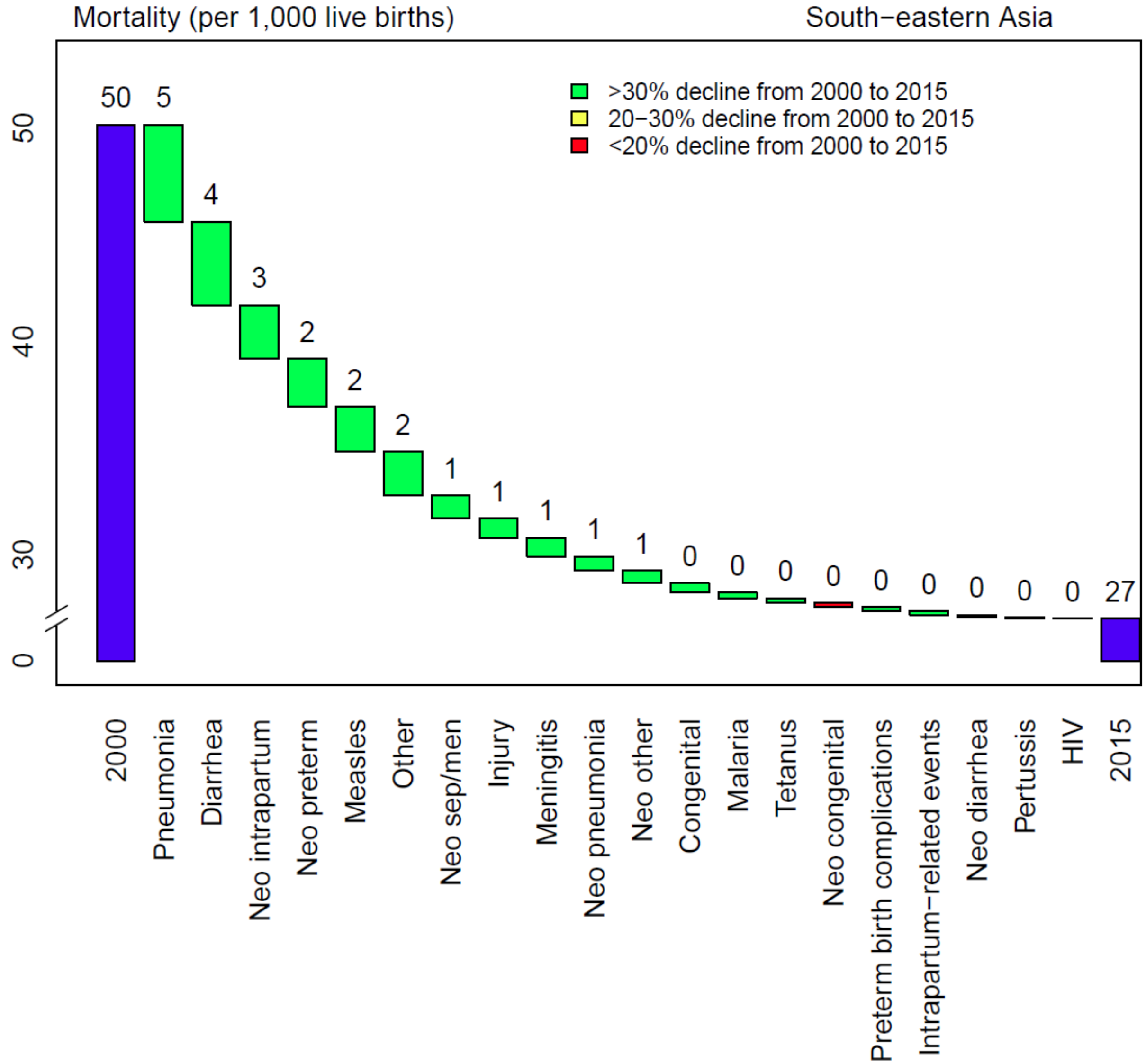

Mortality (per 1,000 live births)
South−eastern Asia
>30% decline from 2000 to 2015
20−30% decline from 2000 to 2015
<20% decline from 2000 to 2015
50
40
30
0
50
5
4
3
2
2
2
1
1
1
1
1
0
0
0
0
0
0
0
0
0
27
2000
Pneumonia
Diarrhea
Neo intrapartum
Neo preterm
Measles
Other
Neo sep/men
Injury
Meningitis
Neo pneumonia
Neo other
Congenital
Malaria
Tetanus
Neo congenital
Preterm birth complications
Intrapartum−related events
Neo diarrhea
Pertussis
HIV
2015

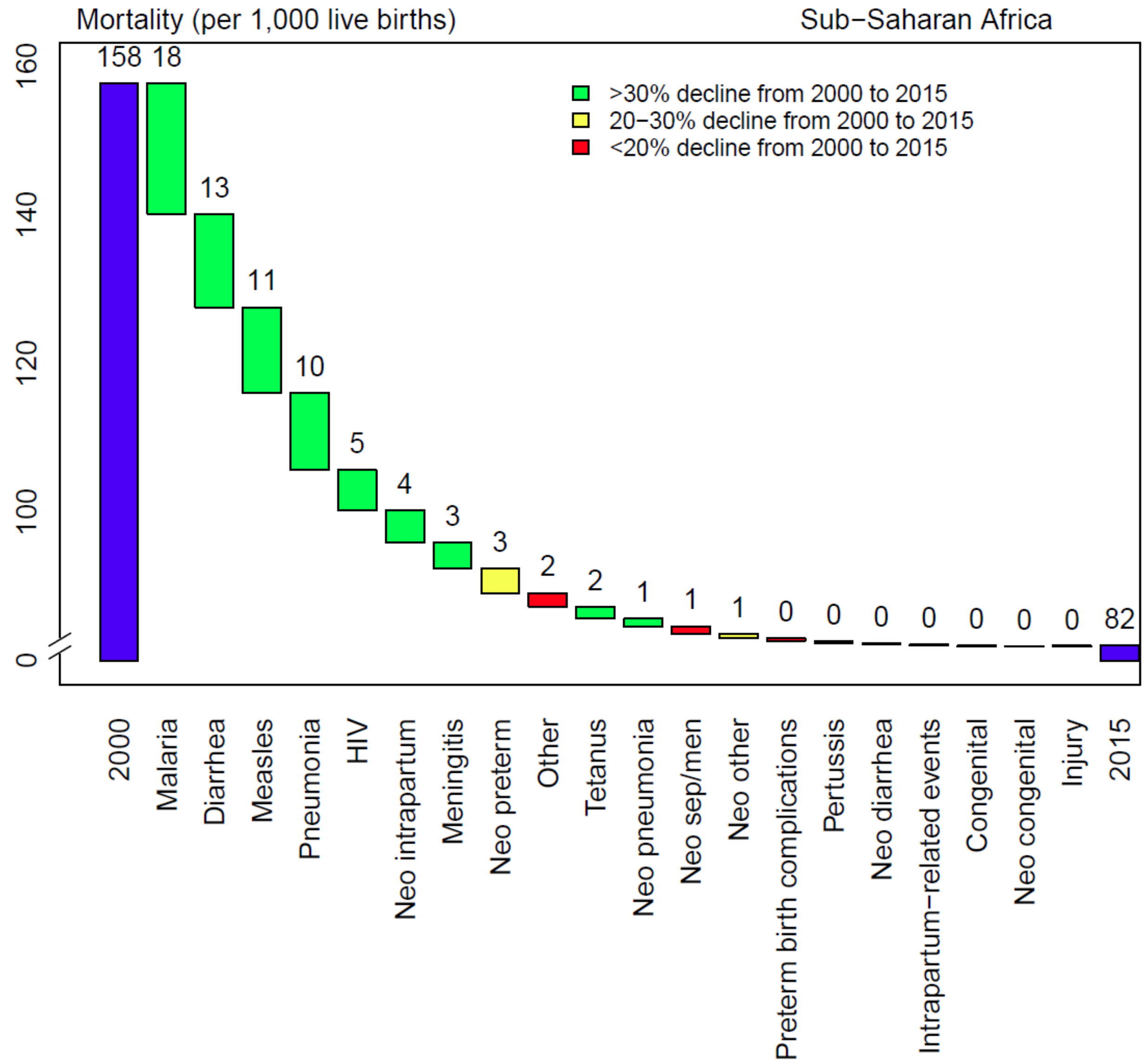

Mortality (per 1,000 live births)
Sub−Saharan Africa
>30% decline from 2000 to 2015
20−30% decline from 2000 to 2015
<20% decline from 2000 to 2015
0
100
120
140
160
158
18
13
11
10
5
4
3
3
2
2
1
1
1
0
0
0
0
0
0
0
82
2000
Malaria
Diarrhea
Measles
Pneumonia
HIV
Neo intrapartum
Meningitis
Neo preterm
Other
Tetanus
Neo pneumonia
Neo sep/men
Neo other
Preterm birth complications
Pertussis
Neo diarrhea
Intrapartum−related events
Congenital
Neo congenital
Injury
2015

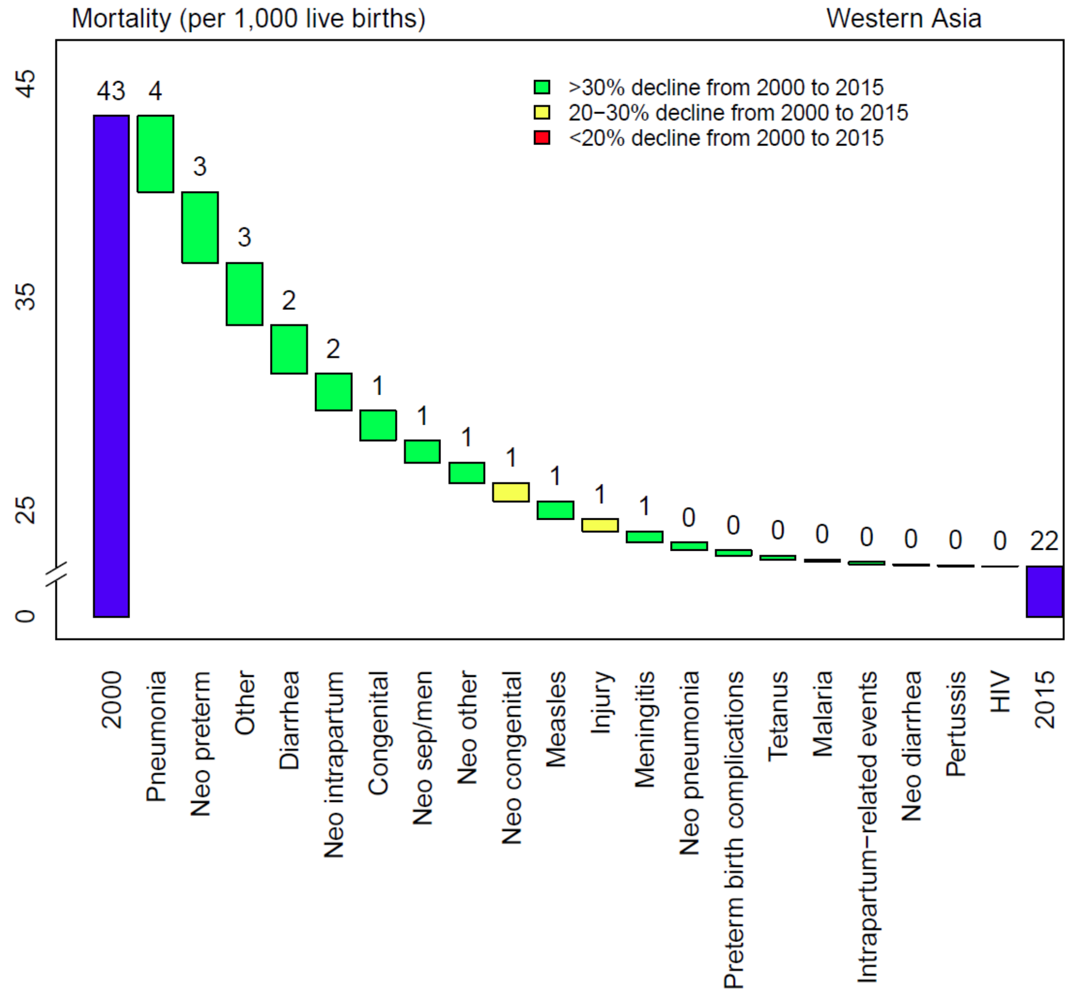

*Neo means neonatal, sep/men means sepsis/meningitis. Causes not specified as neonatal deaths refers to those of 1-59 months.

Webappendix 10. CSMFs by the MDG regions, 2000-2015

Caucasus and Central Asia

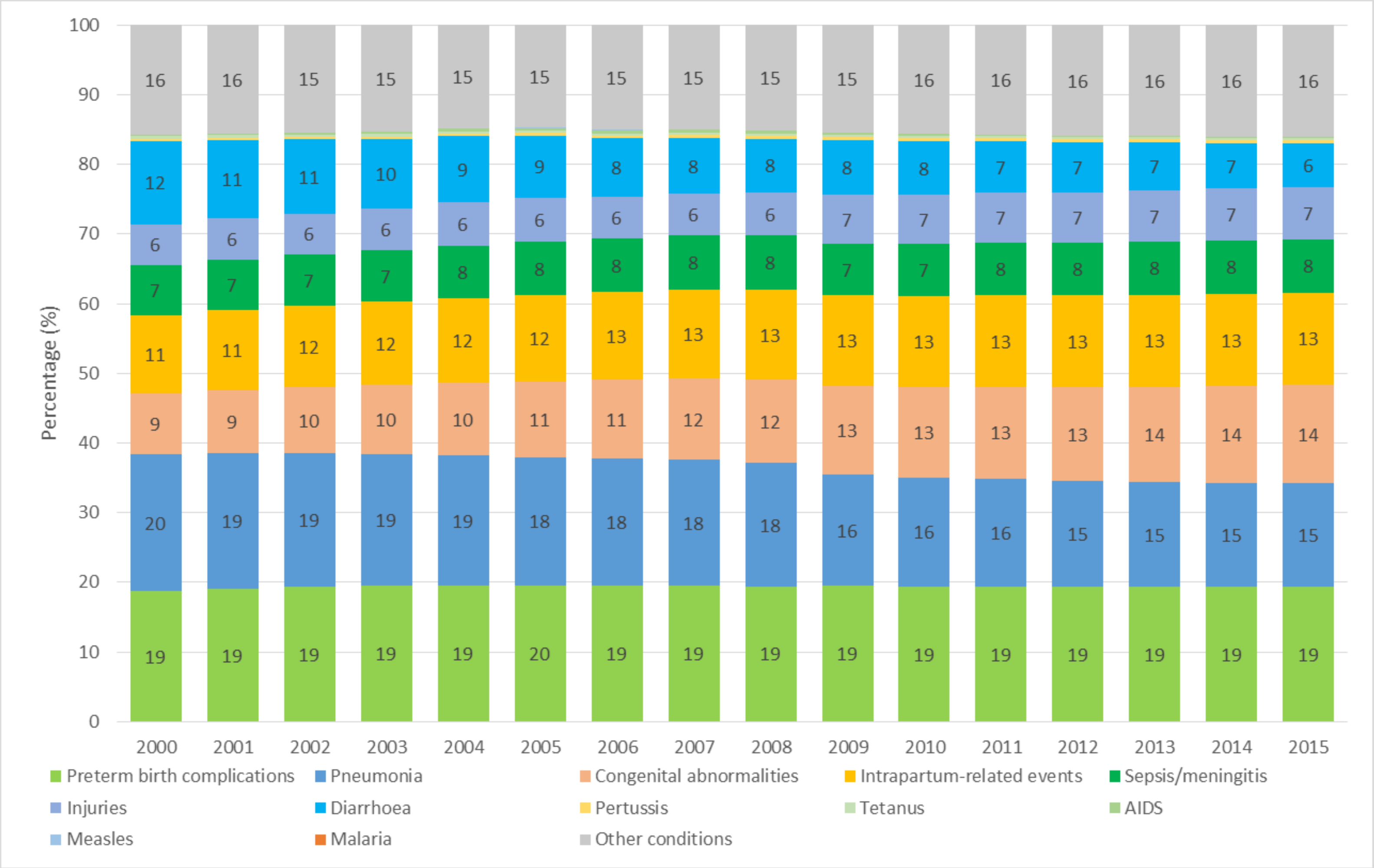

Developed regions

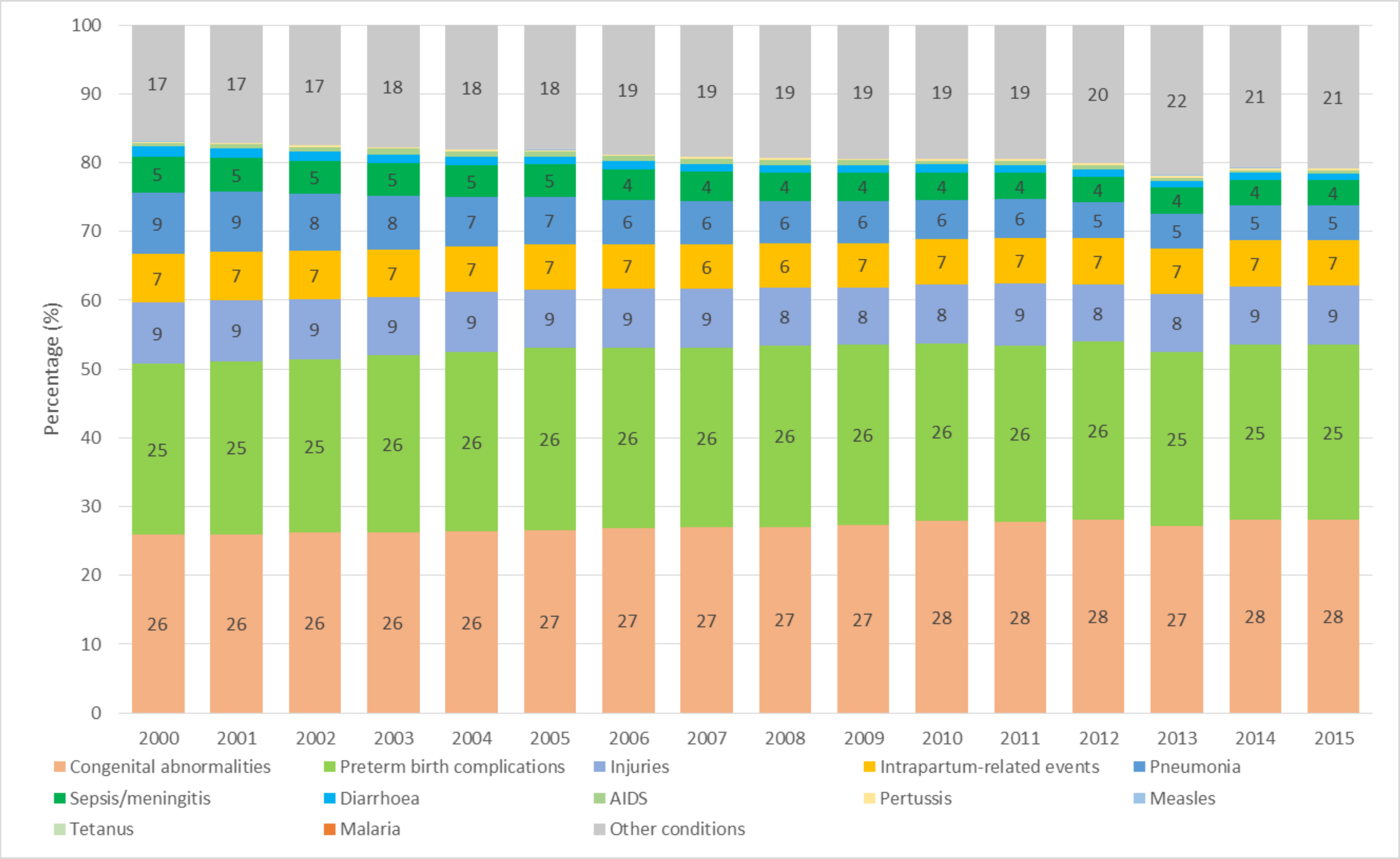

Eastern Asia

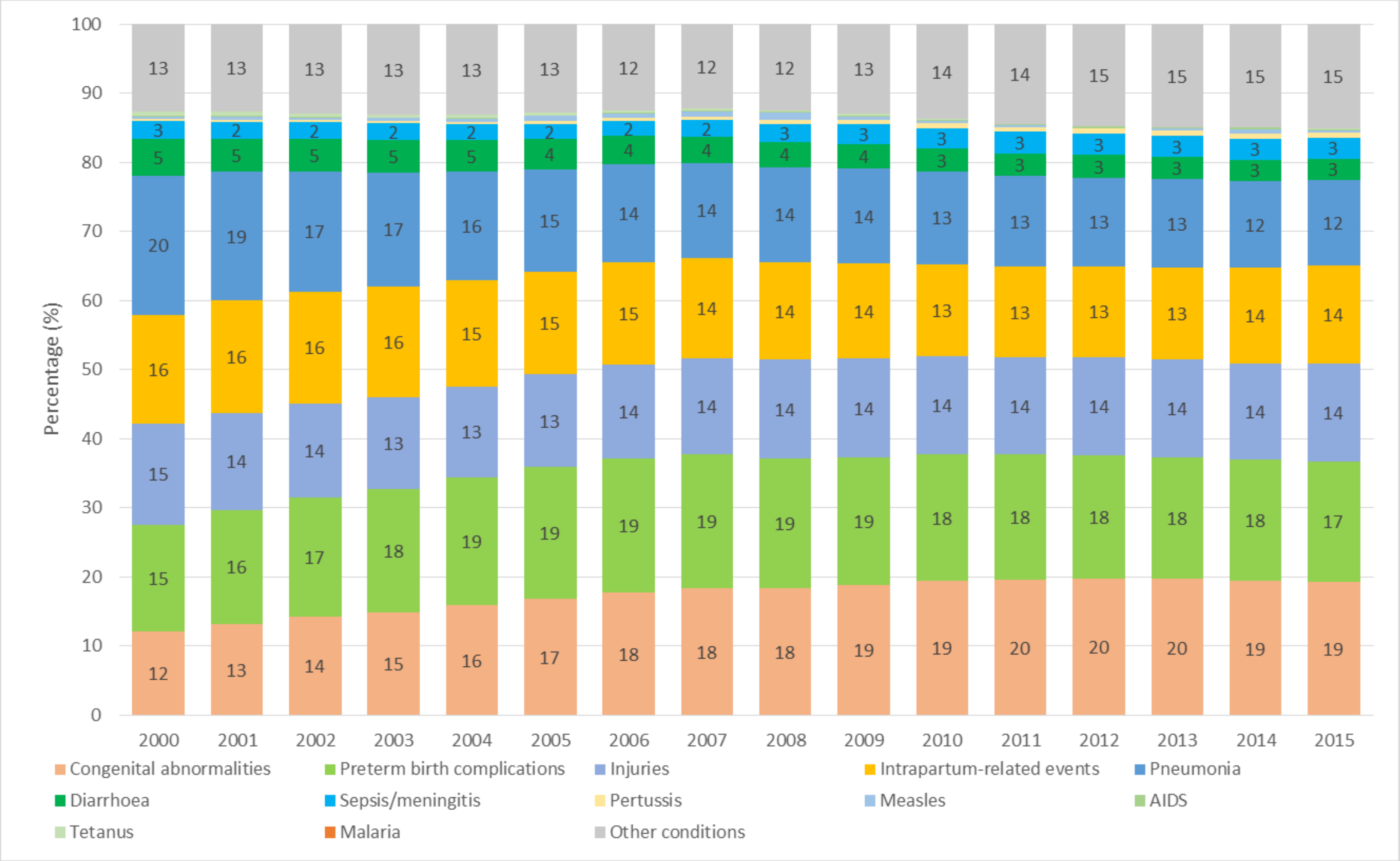

Latin America and the Caribbean

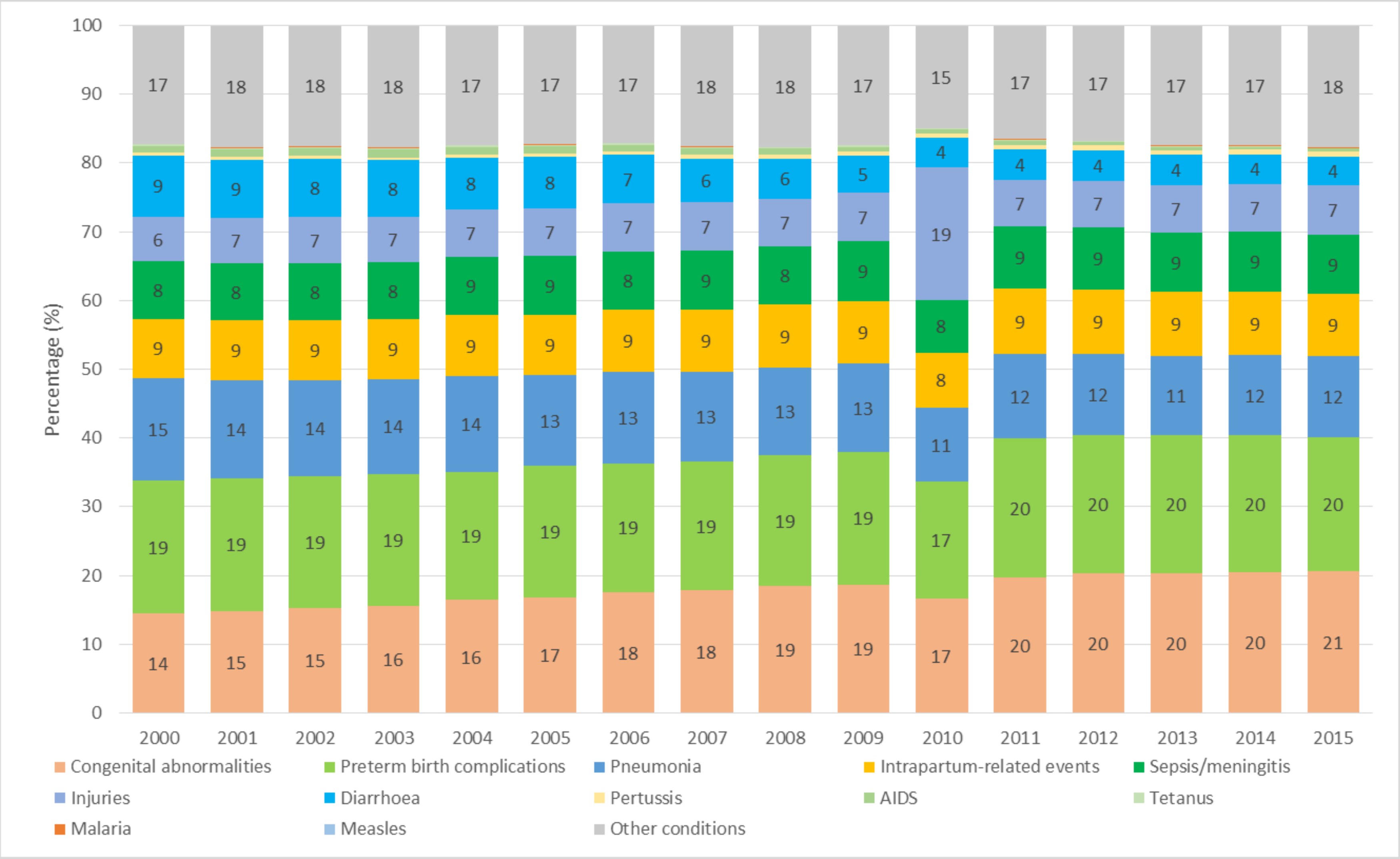

Northern Africa

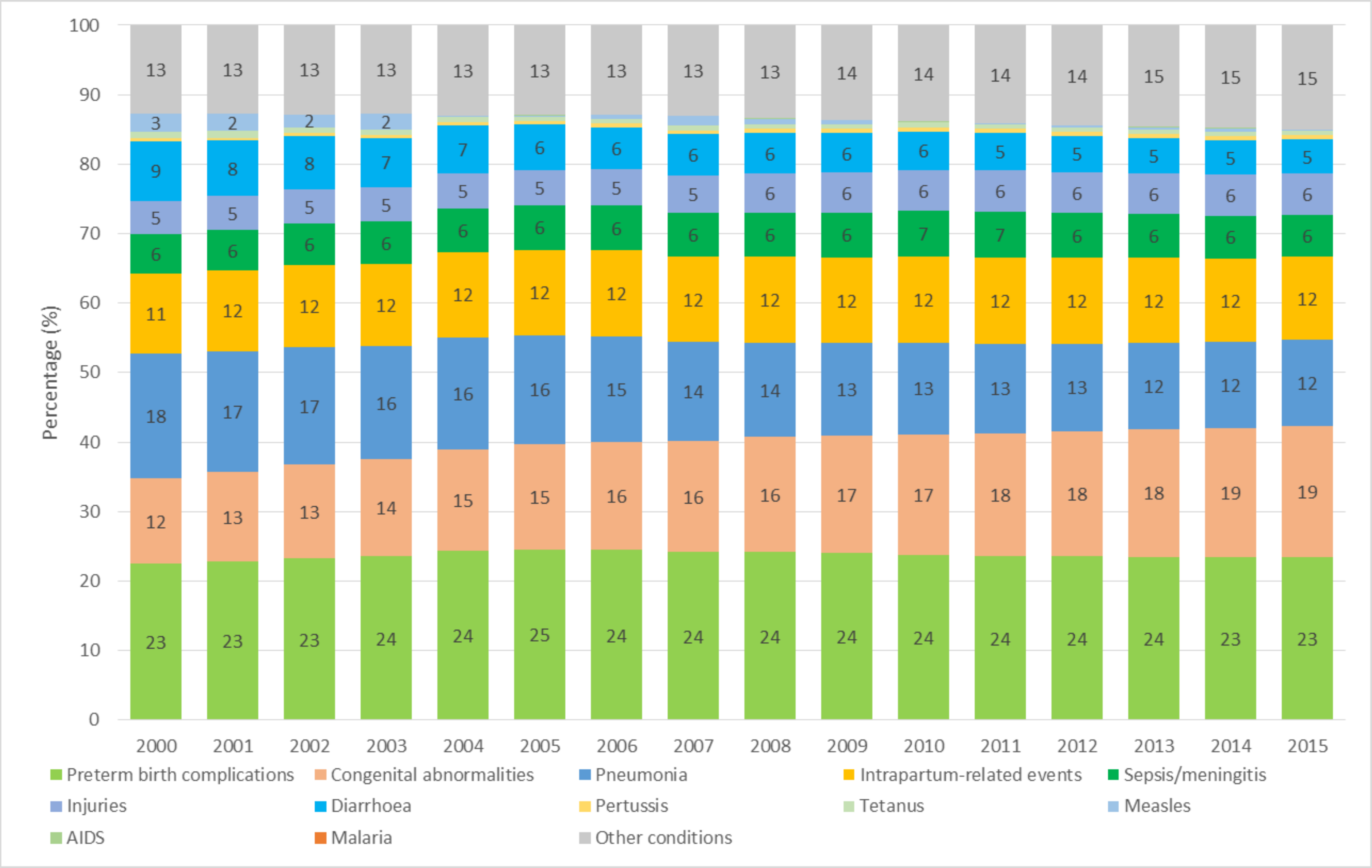

Oceania

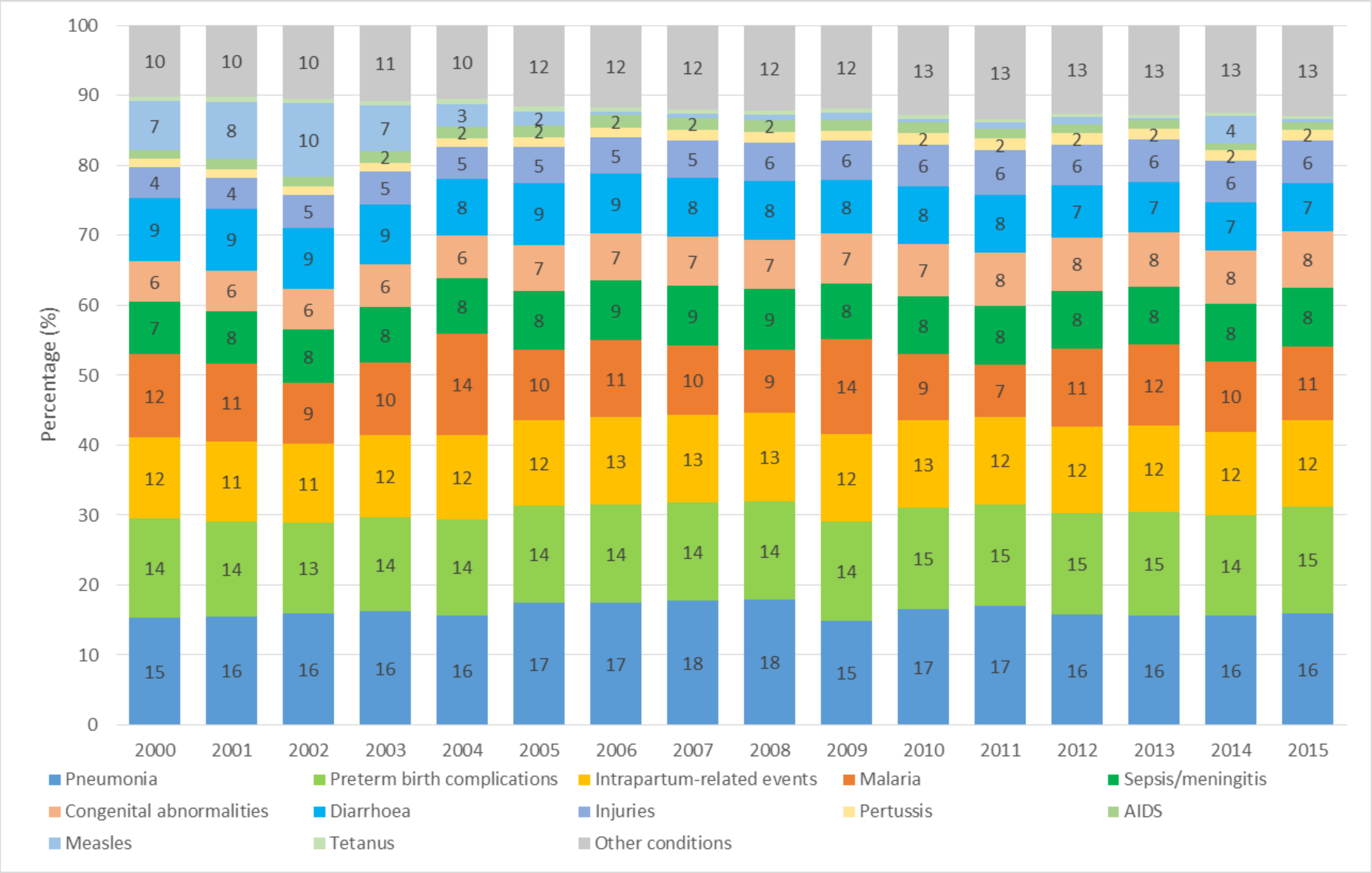

Percentage (%)
Pneumonia
Preterm birth complications
Intrapartum-related events
Malaria
Sepsis/meningitis
Congenital abnormalities
Diarrhoea
Injuries
Pertussis
AIDS
Measles
Tetanus
Other conditions

South-eastern Asia

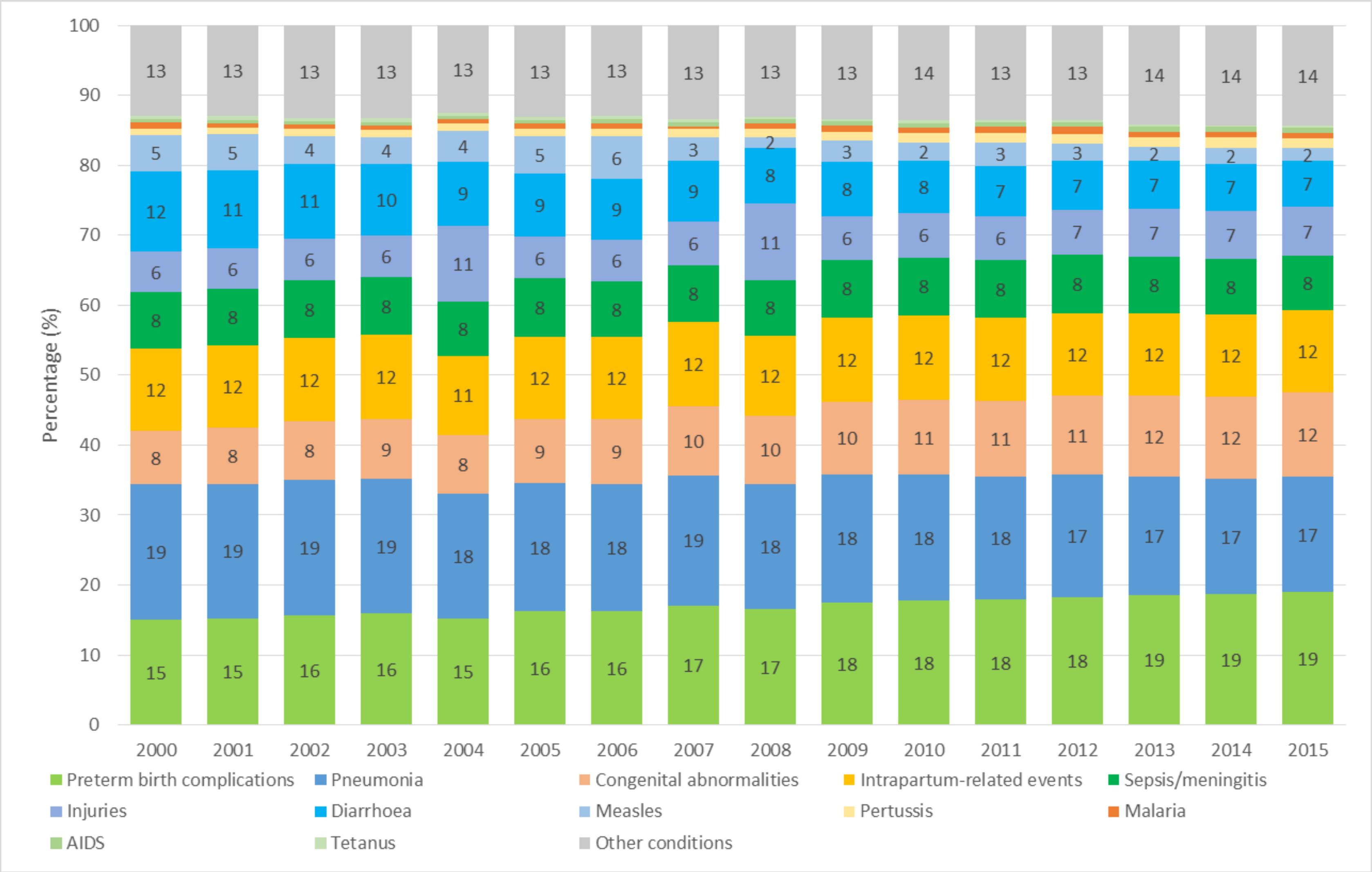

Southern Asia

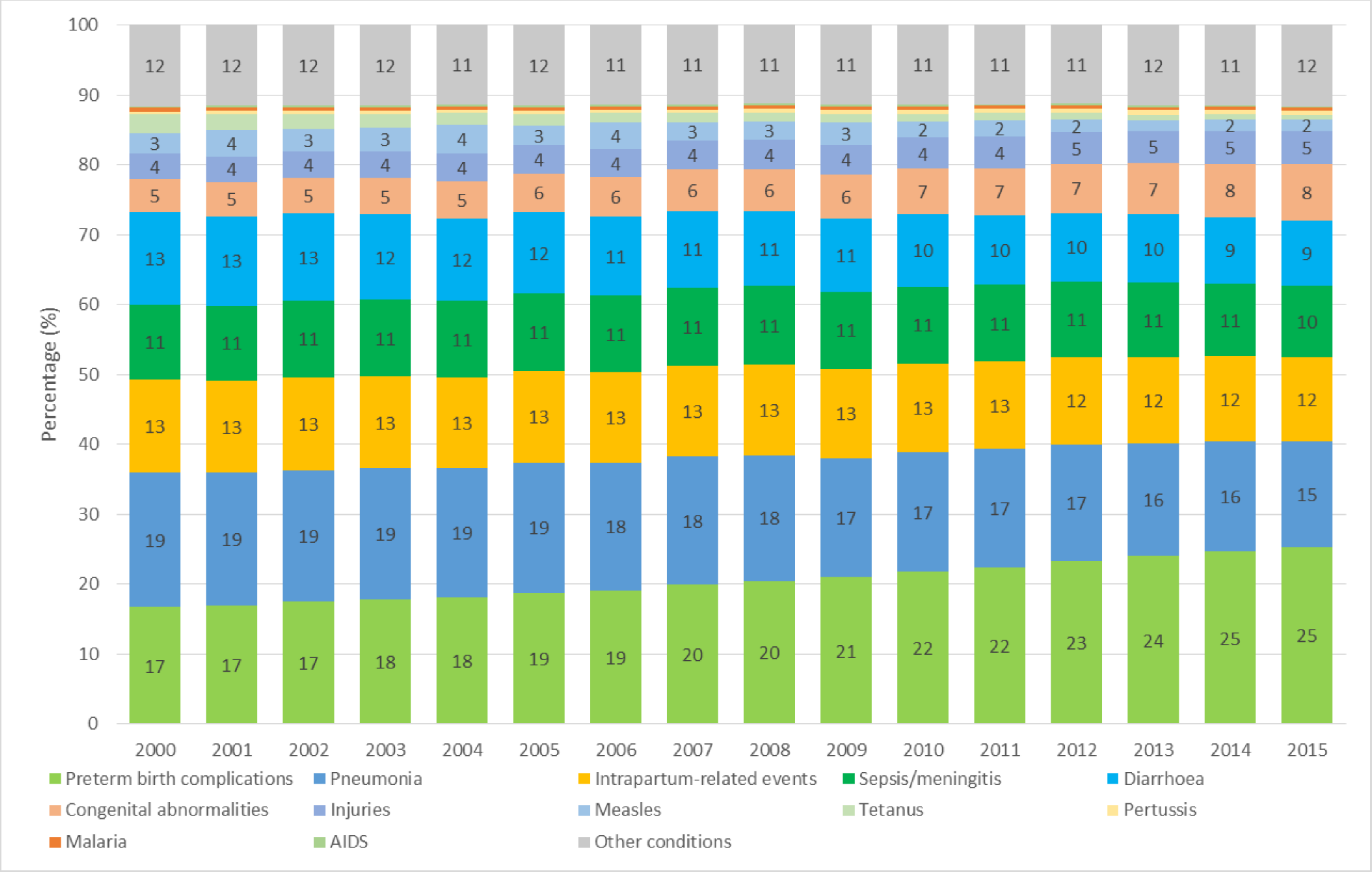

Sub-Saharan Africa

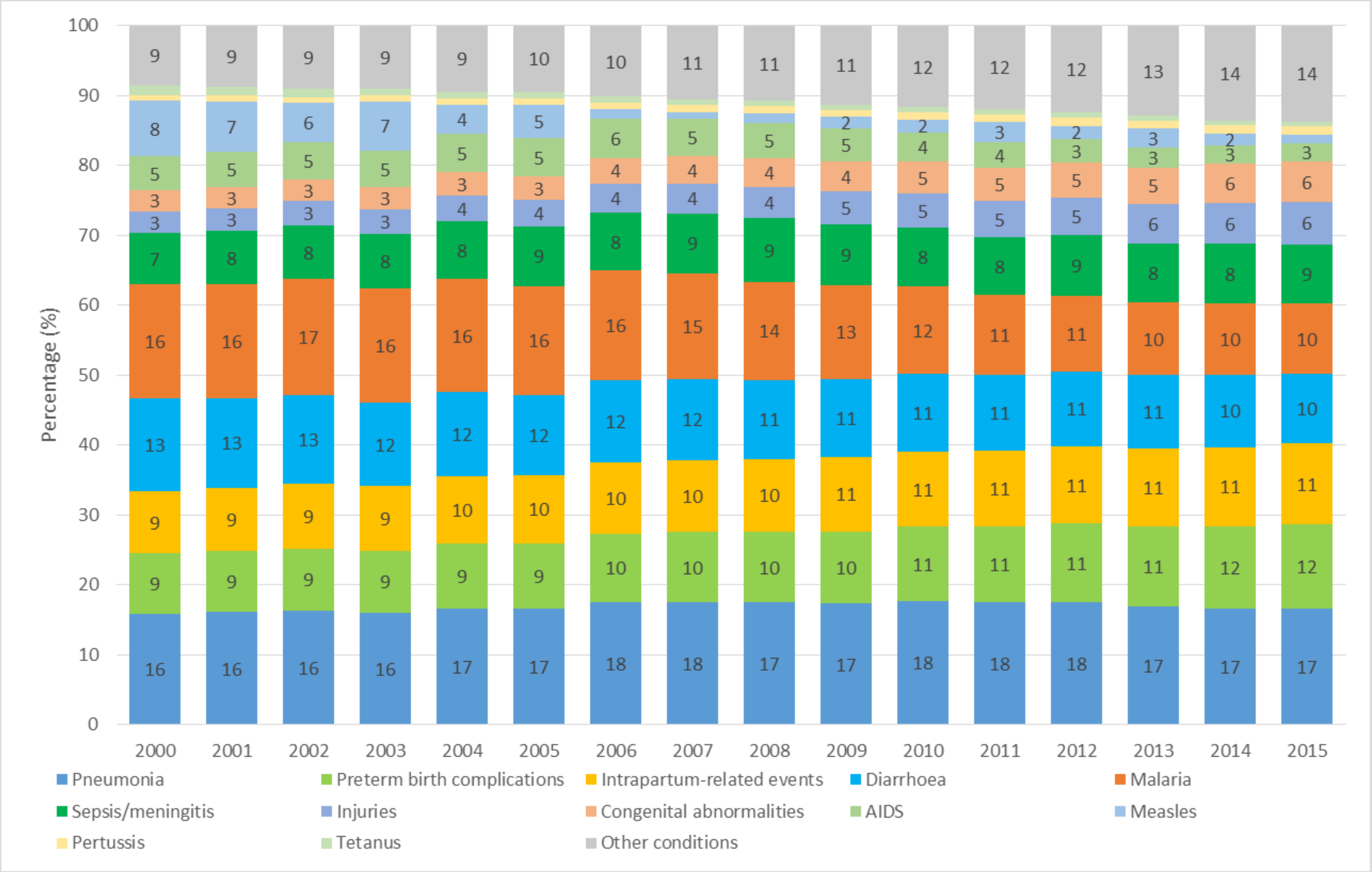

Western Asia

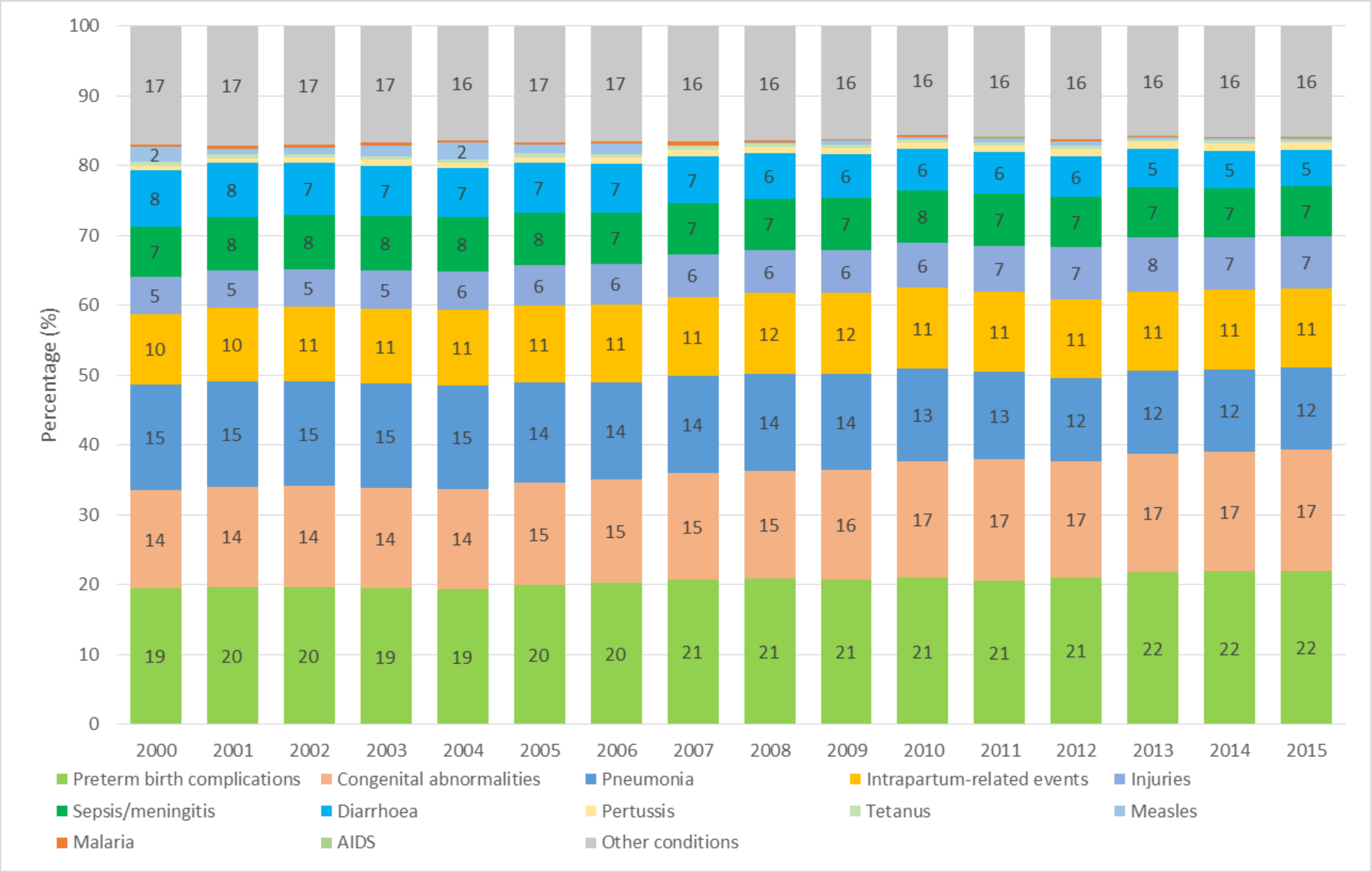

*CSMFs are not labeled if less than 2%

Reference

1. Liu L, Johnson HL, Cousens S, et al. Global, regional, and national causes of child mortality: an updated systematic analysis for 2010 with time trends since 2000. *The Lancet* 2012; **379**(9832): 2151-61.
2. Liu L, Oza S, Hogan D, et al. Global, regional, and national causes of child mortality in 2000–13, with projections to inform post-2015 priorities: an updated systematic analysis. *The Lancet* 2015; **385**(9966): 430-40.
3. LLC EP. DistillerSR - Systematic Review and Literature Review Software. 2015. https://distillercer.com/products/distillersr-systematic-review-software/ (accessed 01/01/2015).
4. Streatfield PK, Khan WA, Bhuiya A, et al. Cause-specific childhood mortality in Africa and Asia: evidence from INDEPTH health and demographic surveillance system sites. *Glob Health Action* 2014; **7**: 25363.
5. Kalter HD, Roubanatou AM, Koffi A, Black RE. Direct estimates of national neonatal and child cause–specific mortality proportions in Niger by expert algorithm and physician–coded analysis of verbal autopsy interviews. *Journal of global health* 2015; **5**(1).
6. India Ministry of Home Affairs. Report on Causes of Death 2004-2006. 2015. http://www.cghr.org/projects/million-death-study-project/ (accessed 06.22 2015).
7. INDEPTH. INDEPTH Network. http://www.indepth-network.org/ (accessed January 26, 2015).
8. Bhatt S, Weiss DJ, Cameron E, et al. The effect of malaria control on Plasmodium falciparum in Africa between 2000 and 2015. *Nature* 2015; **526**(7572): 207-+.
9. Guerra CA, Gikandi PW, Tatem AJ, et al. The limits and intensity of Plasmodium falciparum transmission: implications for malaria control and elimination worldwide. *PLoS Med* 2008; **5**(2): e38. doi:10.1371/journal.pmed.0050038
10. World Health Organization. World Malaria Report 2014. *Geneva: WHO* 2014.
11. Gething PW, Patil AP, Smith DL, et al. A new world malaria map: Plasmodium falciparum endemicity in 2010. *Malar J* 2011; **10**(378): 1475-2875.
12. Johnson HL, Deloria-Knoll M, Levine OS, et al. Systematic evaluation of serotypes causing invasive pneumococcal disease among children under five: the pneumococcal global serotype project. *PLoS Med* 2010; **7**(10).
13. Watt JP, Wolfson LJ, O'Brien KL, et al. Burden of disease caused by Haemophilus influenzae type b in children younger than 5 years: global estimates. *Lancet* 2009; **374**(9693): 903-11.
14. Davis S, Feikin D, Johnson HL. The effect of Haemophilus influenzae type B and pneumococcal conjugate vaccines on childhood meningitis mortality: a systematic review. *BMC Public Health* 2013; **13 Suppl 3**: S21.
15. O'Brien KL, Wolfson LJ, Watt JP, et al. Burden of disease caused by Streptococcus pneumoniae in children younger than 5 years: global estimates. *Lancet* 2009; **374**(9693): 893-902.
16. Lanata CF, Fischer-Walker CL, Olascoaga AC, Torres CX, Aryee MJ, Black RE. Global causes of diarrheal disease mortality in children< 5 years of age: a systematic review. *Plos one* 2013; **8**(9): e72788. doi: 10.1371/journal.pone.0072788

17. WUNEIC. WHO/UNICEF Estimates of National Immunization Coverage (WUENIC). Geneva: World Health Organization. 2016
18. Griffiths U, Clark A, Gessner B, et al. Dose-specific efficacy of Haemophilus influenzae type b conjugate vaccines: a systematic review and meta-analysis of controlled clinical trials. *Epidemiology and infection* 2012; **140**(08): 1343-55.
19. Lucero MG, Dulalia VE, Nillos LT, et al. Pneumococcal conjugate vaccines for preventing vaccine-type invasive pneumococcal disease and X-ray defined pneumonia in children less than two years of age. *Cochrane Database Syst Rev* 2009; (4): CD004977. doi: 10.1002/14651858.CD004977.pub2
20. Lamberti LM, Ashraf S, Walker CLF, Black RE. A systematic review of the effect of rotavirus vaccination on diarrhea outcomes among children under-five. *Pediatric Infectious Disease Journal* 2016; **9**. doi: 10.1097/INF.0000000000001232
21. Black RE, Cousens S, Johnson HL, et al. Global, regional, and national causes of child mortality in 2008: a systematic analysis. *The lancet* 2010; **375**(9730): 1969-87.
22. Mosley WH, Chen LC. An analytical framework for the study of child survival in developing countries. *Population and development review* 1984; **10**: 25-45.
